# Proteomic analysis of heat stress resistance of cucumber leaves when grafted onto Momordica rootstock

**DOI:** 10.1038/s41438-018-0060-z

**Published:** 2018-10-01

**Authors:** Ye Xu, Yinghui Yuan, Nanshan Du, Yu Wang, Sheng Shu, Jin Sun, Shirong Guo

**Affiliations:** 1 Key Laboratory of Southern Vegetable Crop Genetic Improvement in Ministry of Agriculture, College of Horticulture, Nanjing Agricultural University, Nanjing, China; 2 Department of Horticulture, Henan Agricultural University, Zhengzhou, China; 3 Suqian Academy of Protected Horticulture, Nanjing Agricultural University, Suqian, China

## Abstract

Various biotic and abiotic stresses threaten the cultivation of future agricultural crops. Among these stresses, heat stress is a major abiotic stress that substantially reduces agricultural productivity. Many strategies to enhance heat stress tolerance of crops have been developed, among which is grafting. Here, we show that Momordica-grafted cucumber scions have intrinsically enhanced chlorophyll content, leaf area, and net photosynthetic rate under heat stress compared to plants grafted onto cucumber rootstock. To investigate the mechanisms by which Momordica rootstock enhanced cucumber scions heat stress tolerance, comparative proteomic analysis of cucumber leaves in response to rootstock-grafting and/or heat stress was conducted. Seventy-seven differentially accumulated proteins involved in diverse biological processes were identified by two-dimensional electrophoresis (2-DE) in conjunction with matrix-assisted laser desorption/ionization time-of-flight/time-of-flight mass spectrometry (MALDI-TOF/TOF MS). The following four main categories of proteins were involved: photosynthesis (42.8%), energy and metabolism (18.2%), defense response (14.3%), and protein and nucleic acid biosynthesis (11.7%). Proteomic analysis revealed that scions grafted onto Momordica rootstocks upregulated more proteins involved in photosynthesis compared to scions grafted onto cucumber rootstocks under heat stress and indicated enhanced photosynthetic capacity when seedlings were exposed to heat stress. Furthermore, the expression of photosynthesis-related genes in plants grafted onto Momordica rootstocks significantly increased in response to heat stress. In addition, increased high-temperature tolerance of plants grafted onto Momordica rootstock was associated with the accumulation of ribulose-1,5-bisphosphate carboxylase/oxygenase (Rubisco) and oxygen-evolving enhancer protein 1 (OEE1). Taken together, the data indicated that Momordica rootstock might alleviate growth inhibition caused by heat stress by improving photosynthesis, providing valuable insight into enhancing heat stress tolerance in the global warming epoch.

## Heat-tolerant cucumbers: The graft of life

Photosynthesis-related genes and proteins are triggered into action when cucumber is grafted onto another member of the Cucurbitaceae family, helping it to tolerate heat. Shirong Guo of China’s Nanjing Agricultural University and colleagues analysed the genes and proteins of a cucumber plant grafted onto Momordica, a climbing plant from the same family, when it was exposed to high temperatures. Heat stress negatively affects photosynthesis in plants. The team found that cucumber grafted onto Momordica adapted better to heat stress than cucumber grafted onto cucumber. They found this was due to the expression of key enzymes and genes related to photosynthesis. The findings provide insight into the molecular mechanisms involved in enhancing heat stress tolerance in plants by grafting.

## Introduction

Plant physiological processes are negatively affected by heat stress, and therefore, a crucial constraint for crop growth and productivity worldwide^1, 2^. The temperature in summer in the southern region of China usually exceeds 40 °C. High temperature induces leaf wilting, inhibits shoot and root growth, and results in decreased dry matter accumulation^3^. Photosynthesis is sensitive to temperature^4^. Thus, the enzymes for energy distribution and carbon metabolism, especially Rubisco, are significantly affected by heat stress^5^.

Heat-tolerant varieties of crops have higher photosynthetic efficiency than heat-sensitive varieties when exposed to high temperature^6–8^. Photosynthesis is the physiological process that is sensitive to temperature^5^. Therefore, heat stress always disturbs the expression levels of proteins in plants, especially proteins related to photosynthesis. Grafting is a technique that can reduce or eliminate losses in production triggered by soil-borne pathogens, salinization, heat stress or heavy metal uptake in many plants^9, 10^. Grafting-mediated salt tolerance is likely caused by higher photosynthetic ability, carbon assimilation rate, and antioxidant enzyme capacity^11^. Rootstock genotypes influence the adaptive capacity of shoots for heat stress in various plants, and non-grafted plants are influenced more from stress than grafted plants^12^. Signals originating in root-originated signals, such as ABA, can alter miRNAs in shoots, which play a vital role in the regulation of stress response genes under heat stress^13^. Moreover, luffa rootstock promoted the production of hydrogen peroxide (H_2_O_2_), which acted as a second messenger to induce the accumulation of heat shock protein 70 (HSP70), and increased tolerance to heat stress^14^. Root-zone and aerial heat changed the balance of light absorption and utilization in self-grafted plants, and lead to the accumulation of reactive oxygen species (ROS), which contributed to the damage to photosynthetic apparatus^13^. However, plants grafted onto luffa rootstocks attenuated the photosynthetic inhibition and oxidative stress caused by heat stress by promoting the accumulation of HSP70 and elevating antioxidant activity. These results suggested that grafting is an effective method for increasing plant stress tolerance. Nevertheless, there is little information on the mechanism by which rootstocks can elevate stress tolerance using a proteomic approach.

Cucumber (*Cucumis sativus* L.) is an economically important species in protected cultivation that is sensitive to heat stress. Momordica (*Momordica charantia* L.) that is chilling-sensitive, but heat-tolerant originated in India and widely cultivates in the tropics and subtropics^15^. Most cucumber genotypes are highly compatible with other species within the cucurbit family, such as Momordica. Luffa and pumpkin have been used as rootstock and this is the first time we employed Momordica for grafting cucumber. In the present study, we adopted a proteomics-based methodology (2-DE accompanied by MALDI-TOF/TOF-MS) to investigate the effect of Momordica, and to identify differentially accumulated proteins influenced by rootstock and heat stress. We observed that grafting cucumber onto Momordica rootstock increased heat tolerance, which was associated with higher chlorophyll content, leaf area, and photosynthesis. Seventy-seven differentially accumulated proteins were identified in response to rootstock-grafting and/or heat stress, which were grouped into categories according to biological processes. Proteins of the four main groups were involved in photosynthesis (42.8%), energy and metabolism (18.2%), defense response (14.3%), and protein and nucleic acid biosynthesis (11.7%). Moreover, the expression of photosynthesis-related genes and the accumulation of Rubisco and OEE1 in plants grafted onto Momordica rootstock under heat stress were higher than those in plants grafted onto cucumber rootstock. Taken together, Momordica rootstock might alleviate growth inhibition caused by heat stress through improvement of photosynthesis.

## Materials and methods

### Plant materials and treatments

Cucumber (*Cucumis sativus* L., cv. Jinyou No.35, Cs) was used as the scion and Momordica (*Momordica charantia* L., cv. Changlv, Mc) was used as the rootstock. Cleft grafting was used in this study and self-grafted plants were included as controls. Seeds of rootstock were sown in 15-cell polystyrene trays filled with commercial organic substrate (2:2:1 [v/v/v] vinegar waste compost: peat: vermiculite; Peilei, Zhenjiang, China), and seeds of scion were sown in 72-cell trays when the rootstock seedlings had just emerged.

Cleft grafting was performed when the cotyledons of the scions and the second true leaves of rootstock had fully expanded. The seedlings were arranged in a completely randomized design with three replicates per treatment. Grafted plants were transferred to a small plastic arched shed and maintained at a temperature above 25 °C and a relative humidity between 85% and 100% for 7 days until the graft union had completely healed. After full expansion of the third true leaves, grafted plants of similar size were transferred to a growth chamber with a photosynthetic photon flux density (PPFD) of 300 μmol m^−2^ s^−1^, relative humidity of 70–75% and 12 h photoperiod for 7 days until the expansion of the fourth true leaves were completed. The seedlings were treated as follows: (1) self-grafted plants treated with 28 °C/18 °C (day/night), Cs-28 °C; (2) Momordica-grafted plants treated with 28 °C/18 °C (day/night), Mc-28 °C; (3) self-grafted plants treated with 42 °C/32 °C (day/night), Cs-42 °C; (4) Momordica-grafted plants treated with 42 °C/32 °C (day/night), Mc-42 °C. Leaf samples after treatment for 7 days were flash-frozen in liquid nitrogen and stored at −80 °C before gene expression and protein analysis.

### Plant growth, chlorophyll content, and net photosynthetic rate analysis

After 7 days, plants were washed with sterile distilled water, and dried with bibulous paper to measure the fresh weights (FW). Plant materials were incubated at 105 °C for 15 min, placed at 70 °C for 72 h and then weighted to determine the dry weights (DW). Fifteen plants were measured for each treatment. The area of the fully expanded cucumber leaves was estimated using an Expression 1680 scanner (Epson, Sydney, Australia) and analyzed with WinRHIZO (Regent Instruments Ltd, Ontario, Canada).

Chlorophyll content in cucumber leaves was extracted with a mixture of acetone, ethanol, and water (4.5: 4.5: 1, V:V:V) and analyzed according to the method of Arnon^16^. The fourth leaves were used for net photosynthetic rate (Pn) analysis with a portable photosynthesis system (LI-6400, LI-COR Inc., Lincoln, USA), at a temperature of 25 °C, 85% relative humidity, a cuvette air flow rate of 500 mL min^−1^, and an ambient CO_2_ concentration of 380 μmol mol^−1^. A PPFD of 600 μmol m^−2^ s^−1^ was provided by a mixture of red and blue light-emitting diodes.

### Total RNA isolation and gene expression analysis

Total RNA was extracted from cucumber leaves according to the manufacturer’s instructions using an RNA simple Total RNA Kit (Tiangen, China). One microgram of total RNA was used to reverse transcribe to a cDNA template using the ReverTra Ace qPCR RT Kit (Toyobo, Japan).

Quantitative real-time polymerase chain reaction (qRT-PCR) assays were performed using SYBR Green PCR Master Mix (Takara, Japan) in a StepOnePlus^TM^ Real-Time PCR System (Applied Biosystems, USA). The PCR conditions consisted of denaturation at 95 °C for 3 min, followed by 40 cycles of denaturation at 95 °C for 15 s, annealing at 58 °C for 15 s and extension at 72 °C for 30 s. The cucumber actin gene was used as an internal control. Gene-specific primers were designed according to the cDNA sequences as described in Table S1. Relative gene expression was calculated by the 2^−△△Ct^ method^17^.

### Protein extraction

Proteins were extracted using a trichloroacetic acid (TCA) acetone precipitation method with modifications^18^. Leaf samples (1 g) were ground in liquid nitrogen and homogenized in 4 °C extraction buffer containing 8 M urea, 65 mM dithiothreitol (DTT) and 4% (w/v) 3-[(3-cholanidopropyl) dimethylammonio]-1-propanesulfonic acid (CHAPS), and 40 mM Tris for 10 min. The mixture was centrifuged at 25,000 × *g* for 25 min at 4 °C, and the proteins in the supernatant precipitated overnight by the addition of 8 volumes of ice-cold acetone containing 10% (w/v) trichloroacetic acid and 0.07% (v/v) β-mercaptoethanol. Protein samples were precipitated overnight at −20 °C and centrifuged at 20,000 × *g* for 25 min. The pellets were washed three times with ice-cold acetone containing 0.07% (v/v) β-mercaptoethanol and stored at −20 °C for 1 h. Finally, the pellets were air-dried at room temperature and dissolved in rehydration buffer (8 M urea, 1 M thiourea, 2% w/v CHAPS buffer). The concentrations of proteins were determined using a Bio-Rad protein assay kit (USA) and equal amounts of proteins were subjected to 2-DE.

### 2-DE

Isoelectric focusing (IEF) was implemented with pH 4–7, 18 cm immobilized pH gradient (IPG) linear gradient strips (GE Healthcare, USA). The dried protein pellets were rehydrated in rehydration buffer including 7 M urea, 2 M thiourea, 4% 3-[(3-cholanidopropyl) dimethylammonio]-1-propanesulfonic acid (w/v), 40 mM DL-Dithiothreitol (DTT), 0.5% (v/v) IPG buffer 4–7, and 0.01% (w/v) bromophenol blue. IPG strips containing 800 μg protein were rehydrated for 12–16 h at 25 °C. IPG strips were run on an Ettan IPGphor 3 (GE Healthcare, Milwaukee, WI, USA) as follows: 100 V for 1 h, followed by 200 V for 1 h, 500 V for 1 h, 1000 V for 1 h, 4000 V for 1 h, a gradient of 10,000 V for 1 h, and then 10,000 V rapid focus, achieving a total of 75,000 V h. The electric current during IEF did not exceed 50 mA per strip. After IEF, the strips were equilibrated in 5 ml DTT buffer containing 6 M urea, 30% (v/v) glycerol, 2% sodium dodecyl sulfate (SDS), 1% (w/v) DTT, and 50 mM Tris (hydroxymethyl) aminomethane-HCl (Tris-HCl) (pH 8.8) for 15 min followed by iodoacetamide buffer solution containing 2.5% (w/v) iodoacetamide (instead of DTT) for 15 min. Strips were loaded onto a 12.5% SDS-polyacrylamide gel electrophoresis (SDS-PAGE) gel and sealed with 1% molten agarose containing bromophenol blue. Proteins were separated using a Hoefer SE600 Ruby Standard Vertical System (GE Healthcare). Electrophoresis was performed at 15 W per gel until the bromophenol blue dye reached the bottom of the gel. The gels were stained with Coomassie Brilliant Blue (CBB) R-250 for 12 h and de-stained with methanol: acetic acid: deionized water = 1:1:8, v/v until a clear background gel was achieved.

### Image and data analysis

De-stained 2D gels were scanned using an Image Scanner III (GE Healthcare). The digitized images were analyzed with Image master 2D Platinumv5.0 (GE Healthcare, USA). The concentration of each protein spot was determined by the percentage volume (vol. %), and normalized as the ratio of the volume of a single spot to the entire set of spots in the gel. Spots in three replicates were considered for mass spectrometry when there were significant (Duncan’s multiple range test at the *P* *<* 0.05 level) and reproducible changes (a fold change ≥1.5).

### Protein identification

Differentially accumulated proteins were excised from gels and in-gel protein digestion was performed. Mass spectrometry (MS) and tandem mass spectrometry (MS/MS) spectra were obtained using an ABI 5800 proteomics analyzer MALDI-TOF/TOF system (Applied Biosystems, Foster City, CA, USA) operating in a result-dependent acquisition mode. Peptide mass maps were acquired in positive ion reflector mode (20 kV accelerating voltage) with 1000 laser shots per spectrum. Monoisotopic peak masses were automatically determined over a mass range of 800–4000 Da. Averaged MS/MS spectra were obtained in positive ion mode with a collision energy of 2 kV. Monoisotopic peak masses were automatically determined with the signal-to-noise ratio minimum set to 50. The MS and MS/MS spectral data were used to search NCBI (http://www.ncbi.nlm.nih.gov/), a cucumber genomics database (http://cucumber.genomics.org.cn) and a Momordica database (downloaded from NCBI) with the software MASCOT version 2.2 (Matrix Science, London, UK) using the following parameters: trypsin cleavage, one missed cleavage allowed; carbamidomethyl set as a fixed modification; oxidation of methionine allowed as a variable modification; peptide mass tolerance within 100 ppm; fragment tolerance set to ±0.4 Da; and minimum ion score confidence interval for MS/MS data set to 95%. The better organisms from which 77 protein spots were identified were filtered using these parameters: protein score, ion score and the peptide count with ion score step by step.

### Functional classification

Identified proteins of various biological process categories were classified according to Gene Ontology (http://www.geneontology.org) and UniProtKB (http://www.uniprot.org/). Hierarchical clustering of protein expression patterns was performed by Cluster software version 3.0 on log2-transformed spot abundance ratios of heat stress and/or Momordica-grafted combinations compared to the control. A heat map was visualized with Java Treeview.

### Western blot analysis

Cucumber leaves were ground in liquid nitrogen and homogenized in extraction buffer (30 mM Tris-HCl (pH 8.7), 1 mM MgCl_2_, 0.7 M saccharose, 1 mM ethylenediaminetetraacetic acid (EDTA), 1 mM DTT, 1 mM phenylmethanesulfonyl fluoride (PMSF) and 1 mM ascorbic acid to extract proteins. Protein concentrations were measured using a Bio-Rad protein assay kit (USA), denatured at 95 °C for 5 min and stored at −20 °C for further analysis. The denatured protein extracts (10 μg) were separated using a 12% SDS-PAGE for western blotting, and the proteins on the SDS–PAGE gel were transferred to a 0.45 μm poly vinylidene fluoride (PVDF) membrane. The membrane was blocked with 5% non-fat dry milk for 1 h, washed with TBST buffer (including Tris-HCl, NaCl, and tween 20) three times, and incubated with a mouse anti-Rubisco large subunit monoclonal antibody, a rabbit anti-OEE1 monoclonal antibody or a rabbit anti-actin antibody for 2 h. The membrane was washed with TBST buffer and incubated at room temperature for 1 h with Goat Anti-Rat IgG HRP-conjugate antibody or Goat Anti-rabbit IgG HRP-conjugate antibody. Finally, the membrane was washed with TBST three times and developed using diaminobenzidine (DAB) and H_2_O_2_.

### Statistical analysis

At least three independent replicates were used for each determination. All data were statistically analyzed using Duncan’s multiple range test (*P* < 0.05) SPSS 20.0 for Windows.

## Results

### Plant growth, chlorophyll content, and net photosynthetic rate analysis

**Fig. 1:**
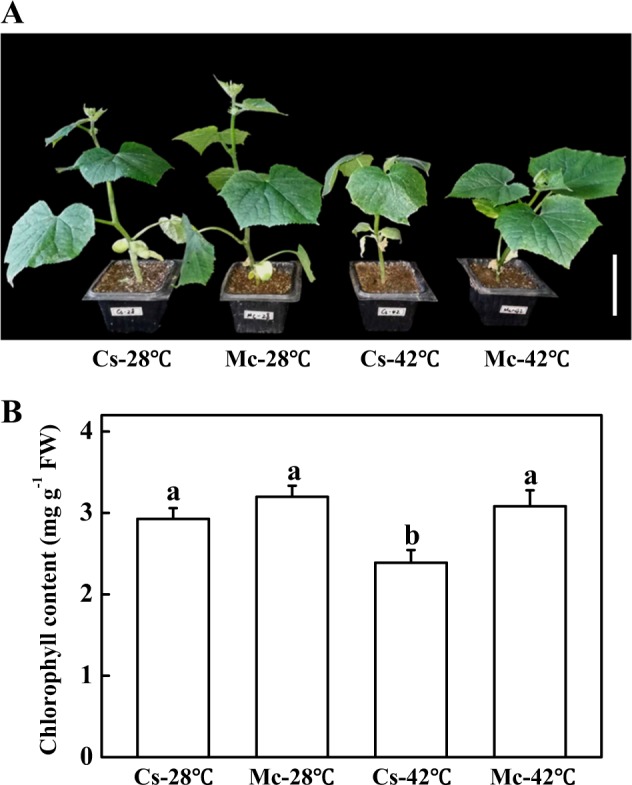
The effects of heat stress on plant growth. (**a**) and chlorophyll content (**b**) of self-grafted and rootstock-grafted cucumber scions. All data are presented as means of three biological replicates (±SE). Means with same letter did not significantly differ at *P* < 0.05 according to Duncan multiple range test. Three independent experiments were performed with similar results. Bar: 10 cm

To investigate the role of Momordica rootstock responses to heat stress, we compared the tolerance to heat stress of cucumber plants grafted onto cucumber rootstock to plants grafted onto Momordica rootstock. The growth of plants was significantly inhibited after heat stress treatment for 7 days (Fig. 1a). The leaves of the scions grafted onto cucumber rootstock were small and chlorotic after heat stress treatment for 7 days. However, the plants grafted onto Momordica rootstock were stronger compared to the plants grafted onto cucumber rootstock (Fig. 1a). There was no significant difference of the growth and biomass between treatments under control temperature (Fig. 1a, Table 1). Fresh weight, dry weight, and leaf area of plants grafted onto cucumber rootstock were 0.85-fold, 0.81-fold, and 0.76-fold change of plants grafted onto Momordica rootstock respectively after 7 days of heat stress (Table 1). The chlorophyll content in all treatments was similar to control plants, except for Cs-42 °C (Fig. 1b).

**Table 1:** Effects of heat shock on plant growth of self-grafted cucumber seedlings and rootstock-grafted cucumber seedlings

Treatment	Fresh weight g/plant	Dry weight g/plant	Leaf area cm^2^/plant
Cs-28 °C	15.64 ± 0.60a	1.48 ± 0.03a	85.73 ± 6.51a
Mc-28 °C	15.43 ± 0.90a	1.45 ± 0.02a	84.88 ± 3.83a
Cs-42 °C	11.53 ± 0.59c	1.09 ± 0.04c	53.83 ± 4.43c
Mc-42 °C	13.54 ± 0.38b	1.34 ± 0.04b	71.27 ± 3.15b

Data are means ± SE. The letters “a”, “b”, “c”, and “d” indicate significant differences between treatments (*P* < 0.05)

**Fig. 2:**
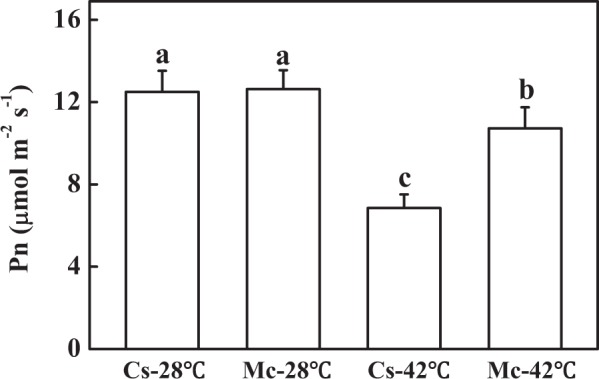
The effects of heat stress on net photosynthesis rate (Pn) of self-grafted and rootstock-grafted cucumber scions. All data are presented as means of three biological replicates (±SE). Means with same letter are not significantly differ at *P* < 0.05 according to Duncan multiple range test. Three independent experiments were performed with similar results

Although the chlorophyll content of plants grafted onto cucumber rootstock decreased significantly after heat stress for 7 days, heat stress had no effect on the chlorophyll content of Momordica-grafted plants (Fig. 1b). We also measured the net photosynthetic rate (Pn) of self-grafted plants and Momordica-grafted plants to compare heat tolerance. There was no significant difference of Pn between self-grafted plants and Momordica-grafted plants under control temperature (Fig. 2). The Pn of self-grafted plants was 0.64-fold of Momordica-grafted plants under heat stress (Fig. 2). Thus, self-grafted plants were more sensitive to heat stress than Momordica-grafted plants.

### Functional classification and clustering analysis of differentially accumulated proteins

**Fig. 3:**
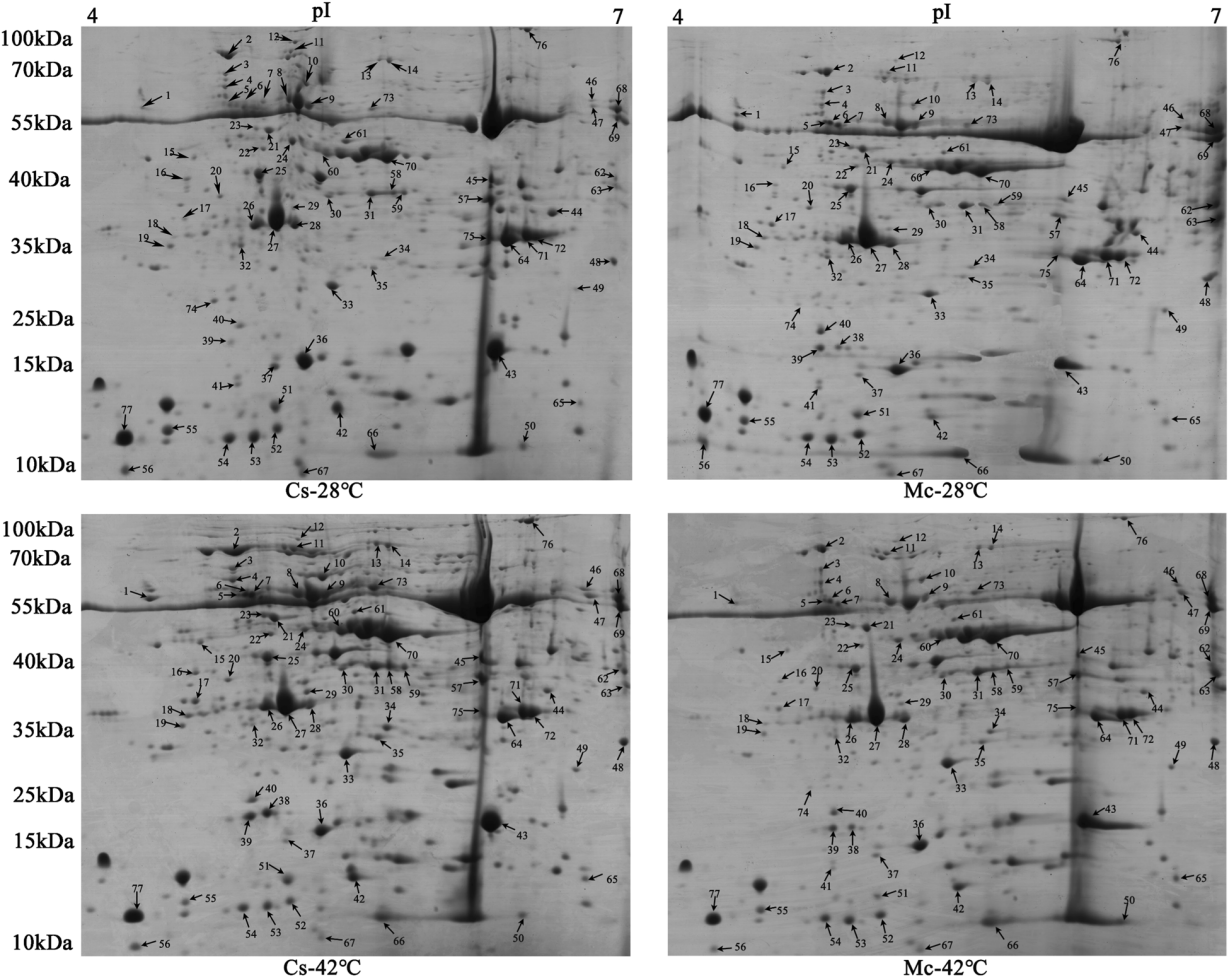
Representative 2-DE gel images of total protein extractions from leaf samples under heat stress for 7 days. An equal amount (800 μg) of total proteins were separated by IEF/SDS-PAGE, stained with Coomassie Brilliant Blue (R-250) and loaded onto each 18-cm gel strip (pH 4–7, linear). The pI and molecular mass standards are indicated at top and left side of each gel image. Spot numbers indicate 77 differentially accumulated proteins annotated according to numbering in Table 2

We analyzed differentially accumulated proteins between self-grafted and Momordica-grafted plants. The protein patterns in 2-DE images from the four treatments were similar (Fig. 3). In comparison of these images, at least 600 protein spots were visualized on each gel, and 77 differently accumulated protein spots were identified by 2-DE coupled to MALDI-TOF/TOF-MS (Table 2).

**Table 2:** Proteins identified by MALDI-TOF/TOF MS

Spot no.^a^	Protein name	NCBI accession no.	Protein score	Cov^b^ (%)	Peptide count	kDa/pI		Fold change^c^			
						Theoretical	Experimental	Cs-28 °C/Cs-28 °C	Mc-28 °C/Cs-28 °C	Cs-42 °C/Cs-28 °C	Mc-42 °C/Cs-42 °C
Photosynthesis (33)											
4	RuBisCO large subunit-binding protein subunit alpha	gi|449456032	732	63%	30	61.40/5.06	66.01/4.85	1.00	0.87	2.13	0.77
10	Rubisco large subunit-binding protein subunit beta, chloroplastic	gi|449452644	1,120	59%	29	64.75/5.86	66.05/5.33	1.00	0.40	2.00	0.37
15	Peptidyl-prolyl cis-trans isomerase CYP38, chloroplastic	gi|449446650	586	63%	22	48.85/5.02	46.50/4.81	1.00	1.01	1.53	0.72
21	Ribulose bisphosphate carboxylase/oxygenase activase, chloroplastic	gi|449441384	707	54%	19	51.77/5.58	53.25/5.20	1.00	1.37	1.55	0.85
23	Ribulose bisphosphate carboxylase/oxygenase activase, chloroplastic	gi|449441384	761	58%	22	51.77/5.58	54.00/5.14	1.00	2.35	2.37	0.85
25	Sedoheptulose-1,7-bisphosphatase, chloroplastic	gi|229597543	602	61%	25	42.08/5.96	41.50/5.15	1.00	0.93	0.62	0.80
26	Oxygen-evolving enhancer protein 1	gi|700193260	445	63%	17	34.94/6.24	35.33/5.13	1.00	1.98	1.06	1.38
27	Oxygen-evolving enhancer protein 1	gi|700193260	600	56%	16	34.94/6.24	35.75/5.24	1.00	1.94	0.96	1.29
28	Oxygen-evolving enhancer protein 1	gi|700193260	971	63%	18	34.94/6.24	35.50/5.36	1.00	1.02	0.56	1.79
35	Triosephosphate isomerase, chloroplastic	gi|449458564	657	58%	19	32.72/7.01	30.75/5.73	1.00	0.73	1.54	0.68
36^d^	Ribulose-1,5-bisphosphate carboxylase/oxygenase large subunit, partial (chloroplast)	gi|545698970 AFH05588	302 304	50% 54%	77	20.29/6.58 18.80/5.92	16.00/5.41	1.00	0.74	0.39	1.37
37^d^	Ribulose-1,5-bisphosphate carboxylase/oxygenase large subunit, partial (chloroplast)	gi|545698970 AFH05590	280 282	21% 27%	5 5	20.29/6.58 18.10/5.50	15.50/5.23	1.00	0.47	0.26	1.52
41^d^	Ribulose-1,5-bisphosphate carboxylase/oxygenase large subunit, partial (chloroplast)	gi|108951132	142	19% 41%	7 2	51.13/5.91 53.40/6.07	13.33/4.99	1.00	0.94	0.00	+∞
		CCD31477	140								
43	Oxygen-evolving enhancer protein 2, chloroplastic	gi|449460024	383	57%	15	28.12/8.61	17.00/6.26	1.00	0.88	0.69	0.66
44	Ribulose-1,5-bisphosphate carboxylase/oxygenase large subunit, partial (chloroplast)	gi|111182702	411	50%	19	51.46/6.00	37.25/6.59	1.00	0.70	0.37	1.03
48	Thylakoid lumenal 29 kDa protein, chloroplastic	gi|449436992	538	49%	19	40.25/7.66	30.40/6.94	1.00	3.44	1.32	1.22
51	Oxygen-evolving enhancer protein 2, chloroplastic	gi|449460024	396	61%	13	28.12/8.61	11.50/5.23	1.00	0.89	0.69	0.46
52	Ribulose bisphosphate carboxylase large chain, partial	gi|659133948	358	34%	10	30.15/6.23	9.75/5.24	1.00	1.55	0.44	1.78
53	Ribulose 1,5-bisphosphate carboxylase/oxygenase large Subunit (plastid)	gi|590000423	309	28%	14	52.61/6.00	9.50/5.11	1.00	0.93	0.32	1.67
54	Ribulose bisphosphate carboxylase large chain, partial	gi|659133948	224	35%	11	30.15/6.23	9.25/4.99	1.00	0.94	0.43	1.26
55^d^	Ribulose-1,5-bisphosphate carboxylase/oxygenase large Subunit, partial (chloroplast)	gi|111182716 CCD31477	170 177	19% 56%	10 4	52.20/6.00 5.34/6.07	10.25/4.68	1.00	0.82	0.26	1.69
56	Plastocyanin A, chloroplast	gi|700204935	260	41%	4	17.01/4.92	8.00/4.41	1.00	1.96	0.87	0.46
60	Beta-form rubisco activase	gi|700195391	568	53%	18	48.29/8.19	48.25/5.53	1.00	1.57	1.59	0.99
62	Ribulose-1,5-bisphosphate carboxylase/oxygenase large subunit, partial (chloroplast)	gi|111182702	497	51%	25	51.46/6.00	40.75/6.95	1.00	16.53	1.90	1.16
63	Ribulose-1,5-bisphosphate carboxylase/oxygenase large subunit, partial (chloroplast)	gi|111182702	753	51%	22	51.46/6.00	39.25/6.97	1.00	14.48	1.32	3.62
64	Ribulose-1,5-bisphosphate carboxylase/oxygenase large subunit, partial (chloroplast)	gi|111182702	676	45%	22	51.46/6.00	34.00/6.35	1.00	0.91	0.65	1.07
66	Ribulose bisphosphate carboxylase small chain, chloroplastic	gi|449434620	456	57%	14	20.69/8.24	8.75/5.73	1.00	2.68	1.31	1.27
67	Ribulose bisphosphate carboxylase small chain, chloroplastic	gi|449434620	405	55%	13	20.69/8.24	7.25/5.40	1.00	0.97	0.34	1.96
70	Beta-form rubisco activase	gi|700195391	723	63%	23	48.29/8.19	47.25/5.81	1.00	1.52	1.55	1.05
71	Ribulose-1,5-bisphosphate carboxylase/oxygenase large subunit, partial (chloroplast)	gi|111182702	756	42%	21	51.46/6.00	34.50/6.46	1.00	0.81	0.65	0.99
72	Ribulose-1,5-bisphosphate carboxylase/oxygenase large subunit, partial (chloroplast)	gi|111182702	586	42%	21	51.46/6.00	34.25/6.52	1.00	0.77	0.63	1.31
75	Ribulose-1,5-bisphosphate carboxylase/oxygenase large subunit, partial (chloroplast)	gi|111182702	502	49%	24	51.46/6.00	35.00/6.25	1.00	0.56	0.87	0.79
77	plastocyanin A, chloroplast	gi|700204935	410	39%	3	17.01/4.92	9.5/4.45	1.00	0.88	0.65	0.77
Energy and metabolism (14)											
8	ATP synthase CF1 beta subunit (plastid)	gi|590000422	904	69%	28	53.81/5.11	59.20/5.19	1.00	1.52	1.17	1.11
9	ATP synthase subunit beta, mitochondrial-like	gi|449465916	1,020	73%	30	60.07/5.90	58.98/5.34	1.00	1.17	1.34	0.51
13	Transketolase, chloroplastic	gi|351735634	197	45%	27	80.57/6.00	96.50/5.75	1.00	2.18	2.00	0.69
14	Transketolase, chloroplastic	gi|351735634	453	54%	32	80.57/6.00	95.17/5.81	1.00	1.67	1.40	0.61
18^d^	ACT domain-containing protein ACR11	gi|449439743 XP_022153437	221 213	35% 21%	9 8	31.96/5.53 31.97/5.71	35.00/4.72	1.00	2.42	3.05	0.38
31	Fructose-bisphosphate aldolase 1, chloroplastic	gi|449464838	455	64%	21	42.87/6.38	39.50/5.72	1.00	1.56	1.30	1.44
45	Fructose-bisphosphate aldolase, cytoplasmic isozyme 1	gi|449444016	708	62%	19	43.08/6.19	42.00/6.26	1.00	0.42	1.48	0.42
50	Nucleoside diphosphate kinase	gi|659074723	308	42%	7	16.40/6.30	8.40/6.41	1.00	1.06	0.56	3.22
57	Malate dehydrogenase, mitochondrial	gi|700198438	478	54%	13	36.18/8.52	38.44/6.25	1.00	0.33	0.87	0.71
58	Fructose-bisphosphate aldolase 1, chloroplastic	gi|449464838	254	44%	13	42.87/6.38	39.75/5.81	1.00	1.19	2.43	0.54
59	Fructose-bisphosphate aldolase 1, chloroplastic	gi|449464838	340	60%	19	42.87/6.38	39.75/5.88	1.00	0.54	1.21	0.86
61	*S*-adenosylmethionine synthase 2	gi|449472806	764	80%	24	43.20/5.35	53.5/5.61	1.00	0.48	0.54	0.69
65	Nucleoside diphosphate kinase	gi|700198251	152	43%	9	25.96/9.18	11.75/6.75	1.00	1.56	1.05	1.52
73	Enolase isoform X1	gi|449451102	421	58%	18	47.71/5.48	62.50/5.72	1.00	1.48	3.20	0.54
Defense response (11)											
5	Abscisic stress ripening-like protein	gi|700190659	487	66%	19	31.72/5.02	59.02/4.87	1.00	2.02	0.95	0.72
6	Abscisic stress ripening-like protein	gi|700190659	621	67%	21	31.72/5.02	59.11/4.89	1.00	11.55	5.03	1.83
7	Abscisic stress ripening-like protein	gi|700190659	832	72%	21	31.72/5.02	58.00/4.93	1.00	5.86	5.43	0.62
16	Peroxidase	gi|700198939	589	52%	11	34.28/4.94	40.25/4.78	1.00	0.62	1.11	0.40
17	Chromoplast-specific carotenoid-associated protein, chromoplast	gi|449434000	682	64%	15	35.22/5.05	36.75/4.77	1.00	2.40	2.90	0.58
34^d^	Ascorbate peroxidase	gi|525507192 AGJ72851	662 652	71% 72%	15 14	27.38/5.43 27.41/5.43	32.50/5.78	1.00	1.30	3.03	0.61
40^d^	2-Cys peroxiredoxin BAS1, chloroplastic	gi|659084460 XP_022144676	442 442	38% 40%	10 10	30.03/8.32 29.55/7.66	20.75/5.04	1.00	2.30	1.44	0.96
46	Leghemoglobin reductase	gi|449459772	514	56%	23	53.57/7.68	64.00/6.79	1.00	1.55	3.02	0.93
49	Peptide methionine sulfoxide reductase A1-like	gi|778690317	333	35%	8	29.89/8.96	26.25/6.73	1.00	2.09	1.38	1.13
68	Catalase isozyme 3	gi|700192329	585	52%	24	57.01/6.84	59.00/6.94	1.00	6.25	2.07	1.13
69	Catalase isozyme 1	gi|778697155	638	62%	25	57.05/6.80	57.29/6.96	1.00	4.57	2.58	1.30
Protein and nucleic acid biosynthesis (9)											
3	Protein disulfide-isomerase	gi|700192511	879	72%	35	57.05/4.88	72.07/4.86	1.00	1.68	2.54	0.54
12	Elongation factor G-2, chloroplastic	gi|449459756	372	46%	29	85.63/5.42	108.25/5.34	1.00	0.32	0.50	1.19
19	Glycine-rich RNA-binding protein blt801	gi|778656094	484	53%	11	28.46/5.07	33.75/4.69	1.00	0.95	0.61	0.78
22	30S ribosomal protein S1, chloroplastic	gi|449459770	851	51%	21	45.30/5.34	48.00/5.17	1.00	0.95	1.54	0.60
24^d^	Actin-7	gi|449459238 XP_022132857	454 454	63% 63%	18 18	41.68/5.31 41.68/5.31	49.25/5.34	1.00	0.98	0.61	1.24
30	Protease Do-like 1, chloroplastic isoform X1	gi|449450105	739	50%	18	46.81/7.13	39.50/5.54	1.00	1.56	1.25	1.15
47	Serine hydroxymethyltransferase	gi|700193477	702	61%	30	57.84/8.12	61.5/6.80	1.00	0.89	3.09	0.97
74	29 kDa ribonucleoprotein, chloroplastic	gi|449440111	173	47%	9	30.48/5.84	24.33/4.88	1.00	0.36	0.00	+ ∞
76	Glycine dehydrogenase, mitochondrial	gi|449450349	656	47%	35	113.29/6.62	123.25/6.49	1.00	0.54	1.54	0.43
Molecular chaperone (7)											
1	Calreticulin	gi|449454026	418	55%	25	48.36/4.45	62.21/4.40	1.00	2.66	5.13	0.25
2^d^	HSP70, chloroplast	gi|700206320 XP_022154567	784 774	51% 50%	35 34	75.35/5.18 75.18/5.26	99.01/4.88	1.00	0.98	1.77	0.40
11	Luminal-binding protein 5	gi|659074058	776	45%	29	73.43/5.10	84.00/5.31	1.00	0.98	3.25	0.57
33	20 kDa chaperonin, chloroplastic	gi|449452602	611	65%	17	26.87/7.85	27.50/5.55	1.00	1.05	1.69	0.97
38	Small heat shock protein, chloroplastic-like isoform X1	gi|778675414	506	51%	10	20.84/5.03	18.67/5.13	0.00	+ ∞	+ ∞	0.11
39	Small heat shock protein, chloroplastic-like isoform X2	gi|659095978	492	48%	11	23.82/6.36	18.75/5.03	1.00	3.65	4.75	0.62
42	Cytosolic class II low molecular weight heat shock protein	gi|700200202	347	67%	7	17.48/5.54	11.50/5.58	1.00	0.40	0.72	0.85
Unknown protein (3)											
20	Uncharacterized protein LOC103496938	gi|449442405	534	74%	22	35.16/4.79	38.75/4.95	1.00	1.72	1.41	0.65
29	Uncharacterized protein LOC101217229	gi|449463844	578	59%	17	37.81/6.22	37.25/5.35	1.00	1.20	1.90	0.56
32	Fruit protein pKIWI502	gi|449434568	525	53%	12	32.52/6.08	33.50/5.05	1.00	0.96	0.38	1.57

^a^Spot number corresponding with 2-DE gel as shown in Fig. 3

^b^Percentage of sequence coverage by matched peptides

^c^The values higher than 1.5 or lower than 0.67 indicate significant changes, with each value representing the mean value of three biological replicates

^d^According to the comparison with the two databases (cucumber and momordica), these protein spots might come from momordica in a great extent. The two results of one protein spot were listed

**Fig. 4:**
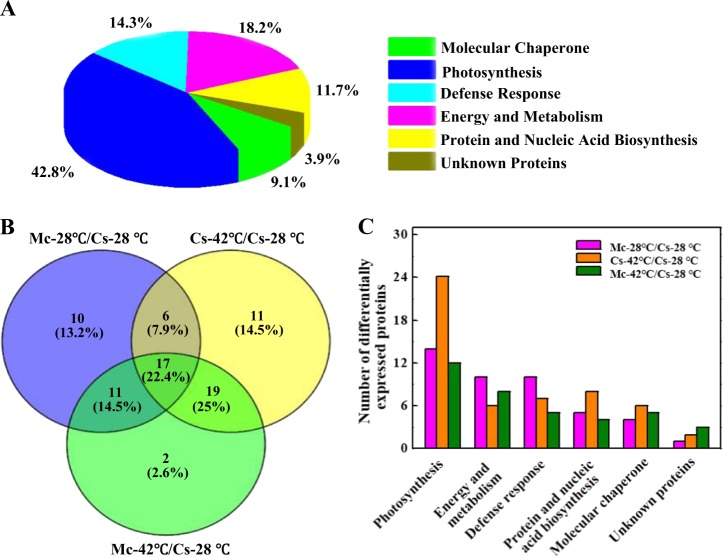
Distribution of differentially accumulated proteins by Momordica rootstock and/or heat stress in cucumber leaves. **a** Functional classification and distribution of all 77 differentially accumulated proteins. **b** Venn diagram showing number of overlapping proteins differentially regulated by Momordica rootstock and/or heat stress compared to control. **c** Functional protein distribution in compared groups (changes ≥1.5-fold or ≤0.67-fold)

In total, 77 differentially accumulated protein from Momordica rootstock and/or heat stress were well matched in NCBI the viridiplantae database (V.2010.12.10, 184045 sequences). These proteins were grouped into six categories in terms of their biological functions according to Gene Ontology and UniProt Protein Knowledgebase (Fig. 4a). The identified proteins were sorted into photosynthesis (42.8%), energy and metabolism (18.2%), protein and nucleic acid biosynthesis (11.7%), defense response (14.3%), molecular chaperone (9.1%), and unknown proteins (3.9%) (Fig. 4a). Among the 77 differentially accumulated protein spots, 53 proteins were significantly regulated by heat stress compared to Cs−28 °C, and 44 proteins were differentially accumulated by rootstock under control temperature. There were 36 proteins that were influenced by Momordica rootstock compared to self-grafted plants under heat stress, and 37 proteins differentially regulated by heat stress when grafted onto Momordica rootstock (Fig. 4b). Photosynthetic proteins were most enriched in heat stress and/or Momordica rootstock, followed by identical percentages changes in energy and metabolism, defense response, and protein and nucleic acid biosynthesis proteins. In the comparison of Mc-42 °C/Mc-28 °C, the number of differentially accumulated proteins of four main categories was less than that in comparison of Cs-42 °C/Cs-28 °C (Fig. 4c).

**Fig. 5:**
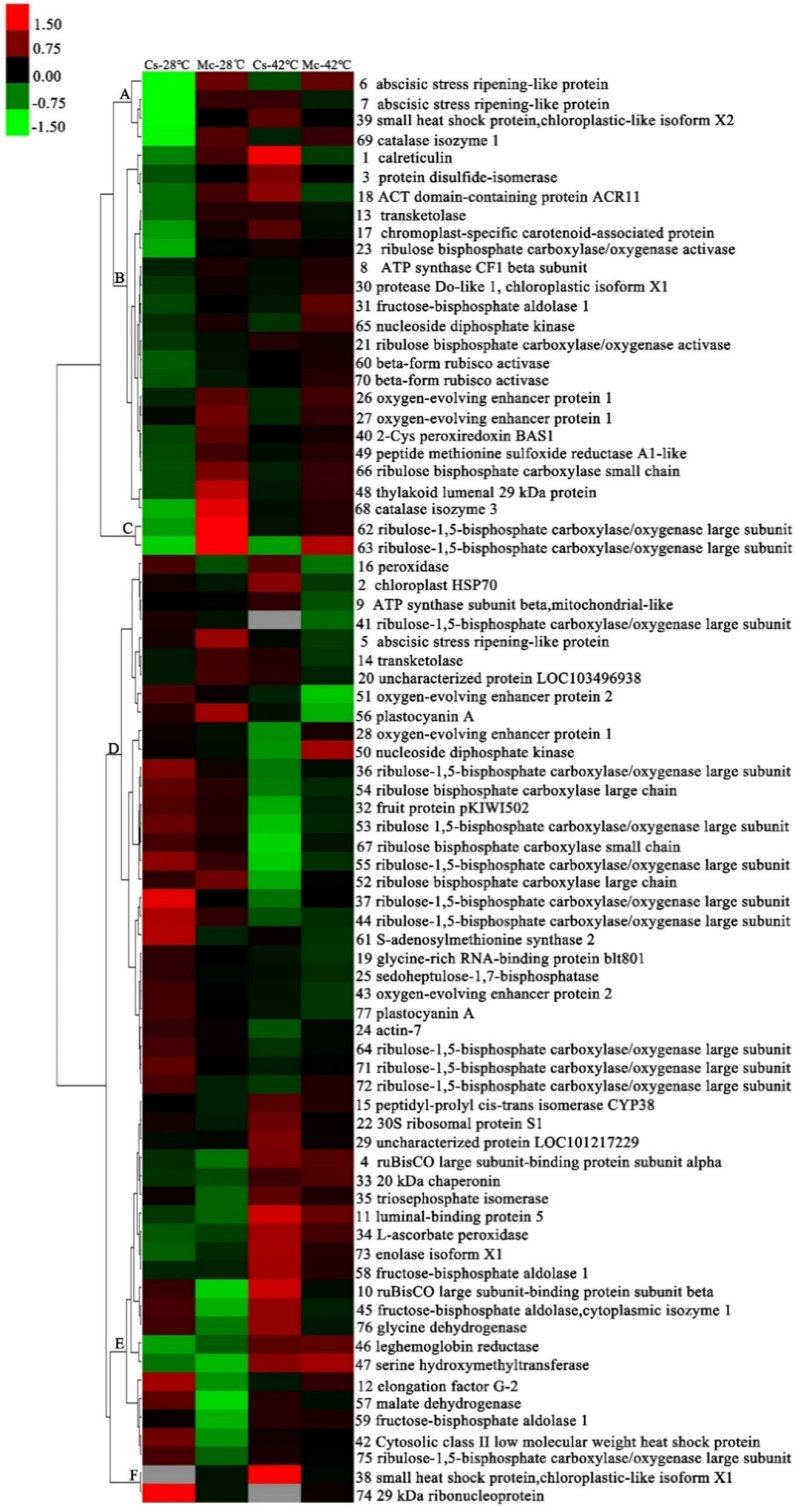
Hierarchical clustering analysis of differentially accumulated proteins responding to Momordica rootstock and/or high temperature. Fold changes of protein abundance among four treatments were log2 transformed and delivered to Cluster and Treeview software. Each row represents individual protein spots and spot numbers, and protein names are labeled at right of corresponding heat maps. Red and green show higher and lower expression levels, respectively

For a comprehensive view of the differentially accumulated proteins induced by heat stress and Momordica rootstock, hierarchical clustering was performed, and proteins that were similarly accumulated were grouped together (Fig. 5). Cluster A consisted of four protein spots (spots 6, 7, 39, and 69) that were upregulated by Momordica rootstock and heat stress, but two were recovered by grafting onto Momordica rootstock, whereas the other two protein spots were upregulated compared to Cs-42 °C. Cluster B included 20 protein spots, and most significantly accumulated in the other three treatments compared to Cs-28 °C. These protein spots were involved in energy and metabolism, photosynthesis and defense response. Cluster C contained two photosynthesis-related protein spots, which were upregulated by Momordica rootstock regardless of temperature treatment. Cluster D contained 29 protein spots that were primarily related to photosynthesis, and most of them were downregulated by heat stress. However, the majority of photosynthesis-related protein spots were upregulated in Mc-42 °C compared to Cs-42 °C. Cluster E included 20 protein spots that were upregulated by heat stress treatment, and the majority were downregulated when grafted onto Momordica rootstock. Cluster F consisted of two protein spots that were not significantly regulated by heat stress in plants grafted onto Momordica rootstock.

### Expression analysis of several photosynthesis-related genes

**Fig. 6:**
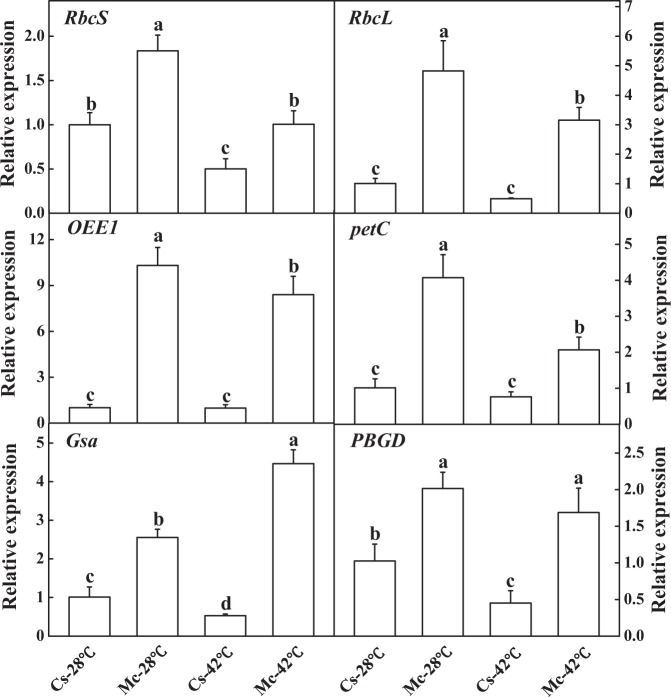
Effects of Momordica rootstock and/or heat stress treatment on transcripts of RbcS, RbcL, OEE1, Gsa, petC, and PBGD in leaves of cucumber scions. Each bar represents a mean ± SE of three independent experiments. Means followed by different letters indicate significant differences between treatments (*P* < 0.05) according to Duncan’s multiple range test

The abundance of photosynthesis and photosynthetic pigment metabolism progress-related proteins were altered by the treatments. To verify these results, we analyzed the expression pattern of three photosynthesis-related genes (*RbcS, RbcL*, and *OEE1*) and three genes related to photosynthetic pigment metabolism (*petC*, *Gsa*, and *PBGD*). Momordica rootstock upregulated all genes compared to self-grafted plants under control temperature (Fig. 6). The transcription of these genes in the plants grafted onto cucumber rootstock was inhibited after heat stress treatment for 7 days, but they were dramatically upregulated in Momordica-grafted plants.

### Validation of differentially accumulated proteins

**Fig. 7:**
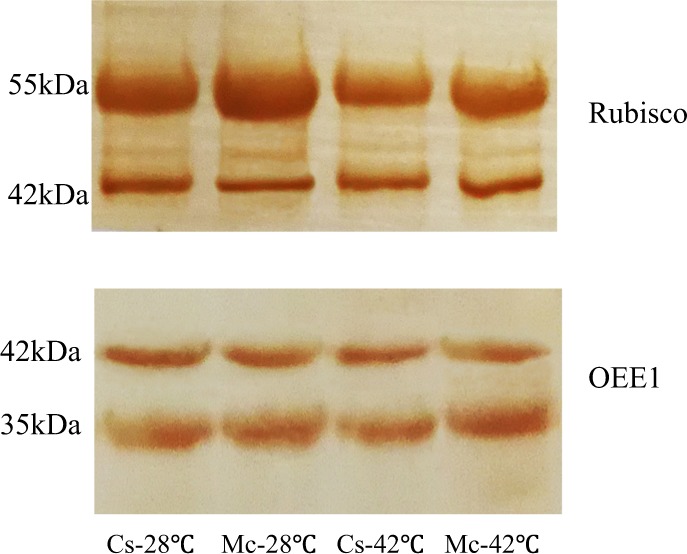
Western blotting analysis of Rubisco large subunit and OEE1 expression level in leaves of cucumber scions after heat treatment for 7 days. Western blotting was performed three times with three independent biological samples, and similar results were obtained

As shown in Table 2, among the half of photosynthesis-related differentially accumulated proteins that were significantly regulated by heat stress and/or Momordica rootstock were Rubisco large subunit and OEE1. This indicated that Rubisco large subunit and OEE1 were vital plant proteins for coping with heat stress. To further confirm this result, we used western blotting to analyze the abundance of Rubisco large subunit and OEE1. The results agreed with the 2-DE data. The abundance of Rubisco large subunit and OEE1 in the self-grafted plants decreased under heat stress compared to Cs-28 °C (Fig. 7). However, the abundance of these proteins was maintained by Momordica rootstock under heat stress (Fig. 7).

## Discussion

**Fig. 8:**
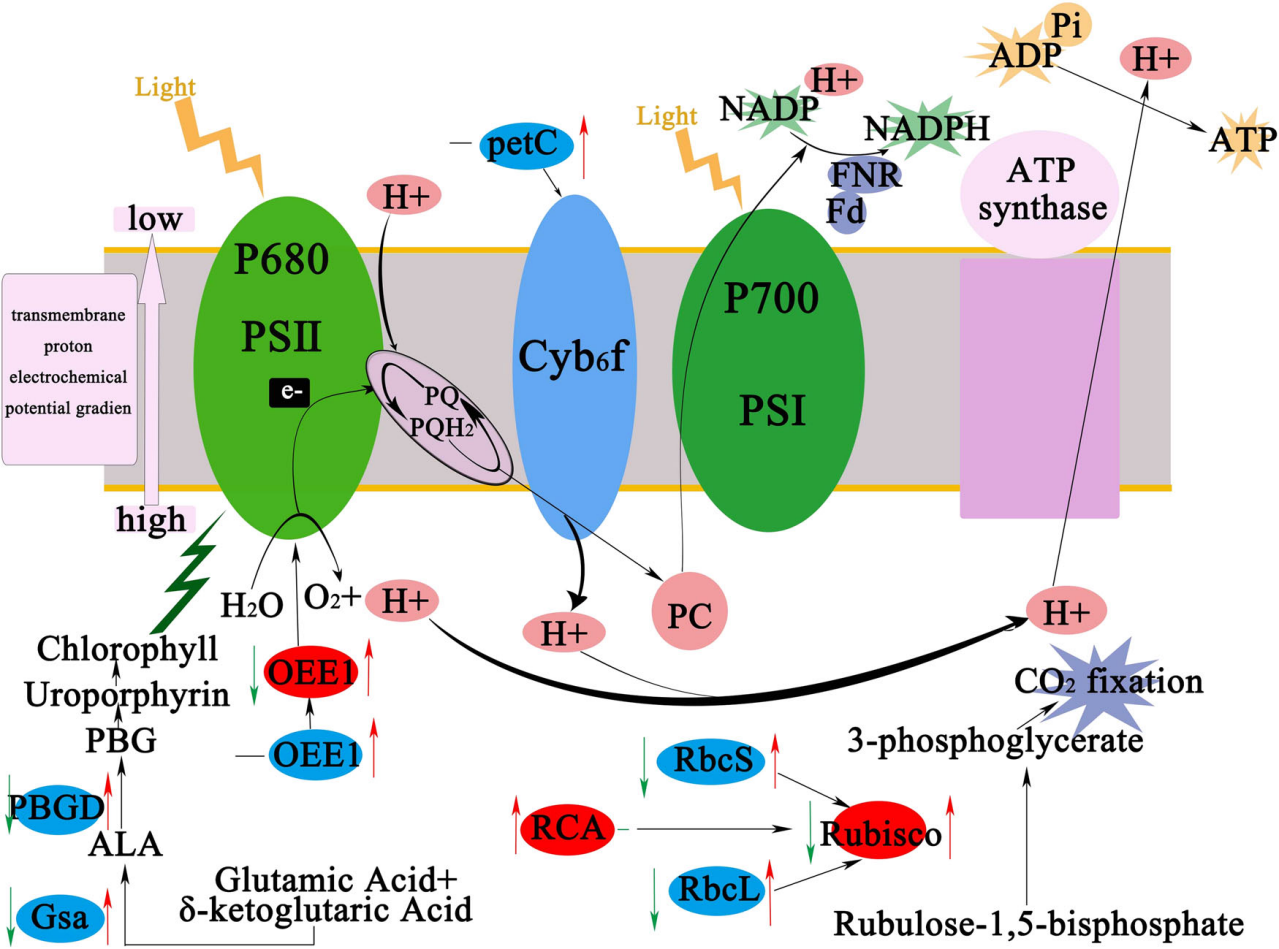
Schematic presentation of effects of heat stress and Momordica rootstock on photosynthesis metabolism in cucumber leaves. Changes in protein (marked in red ellipses) and gene expression (marked in blue ellipses) were integrated. Arrows at left of ellipses indicate changes induced by heat stress and arrows at right indicate changes induced by Momordica rootstock under heat stress. Red or green arrows show upregulation or downregulation, respectively, while black short lines indicate no change. OEE1 oxygen-evolving enhancer protein 1, Rubisco ribulose-l,5-bisphosphate carboxylase/oxygenase, RCA ribulose-l,5-bisphosphate carboxylase/oxygenase activase, petC cytochrome b6-f complex iron–sulfur subunit, PBGD porphobilinogen deaminase, Gsa glutamate-1-semialdehyde 2,1-aminomutase, RbcS rubisco small subunit, RbcL rubisco large subunit

The cucumber scions grafted onto Momordica rootstock showed accumulation of proteins related to photosynthesis, and enhanced photosynthetic capacity under heat stress, which alleviated growth inhibition (Fig. 8). These results suggested that Momordica rootstock elevates resistance to heat stress. The smaller number of differentially accumulated proteins in Mc-42 °C/Mc-28 °C compared to Cs-42 °C/Cs-28 °C indicated that Momordica grafted plants were not as adversely affected by heat stress. The proteins that regulated metabolic processes by Momordica rootstock and heat stress are discussed below.

### Proteins related to photosynthesis

Photosynthesis is considered to be the most significant physiological process because of its regulation of plant biomass accumulation^19^. As photosynthesis is sensitive to temperatures, the expression of photosynthesis-related proteins is worthy of investigation.

The Calvin cycle, which is light-independent and utilizes energy to convert CO_2_ and H_2_O into organic compounds, is essential to photosynthesis^20, 21^. In this study, the majority of Calvin-cycle-related proteins, including the Rubisco large subunit, RCA and sedoheptulose-1,7-bisphosphatase, were altered by Momordica rootstock grafting and/or heat stress. Indeed, the abundance of Rubisco large subunits of self-grafted plants was downregulated under heat stress, but they were upregulated when cucumber scions were grafted onto Momordica rootstock. Rubisco in the passivation state has no activity and it can be activated by Rubisco activase (RCA). In this study, RCA was upregulated by heat stress. The expression level of RCA had no positive correlation with its activity^22, 23^, which might account for the lower content of RCA in Mc-42 °C compared to Cs-42 °C. The activity of sedoheptulose-1,7-bisphosphatase is related to the rate of carbon assimilation^24, 25^, and the different levels of SBPase in the two plant types under heat stress indicated that the Calvin cycle was affected by the genotype of the rootstock. Photosynthesis in plants is a complicated process that is co-regulated by many factors, such as photosynthetic apparatus^26^. The regulatory role of Momordica rootstock on photosynthetic rate when combined with photosynthetic apparatus, light reaction and Calvin cycle requires further study.

Oxygen-evolving enhancer protein, a part of the oxygen evolving complex of PSII, is involved in the light reaction of PSII ^27, 28^. The expression of OEE1 (spots 26, 27, and 28) in self-grafted plants decreased under heat stress, but increased when cucumber scions were grafted onto Momordica rootstock. These results indicated that Momordica rootstock played a vital role in maintaining the stability of PSII, and is consistent with a previous proteomics study^29^. However, the expression of OEE2 was different from OEE1, and the relationship between OEE1 and OEE2 is not clear yet. Triosephosphate isomerase (spot 35) is able to catalyze the conversion of propylene phosphate isomers and D-3-phosphoric acid, which can supply energy for growth^30, 31^. In this study, it was upregulated by heat stress, which suggested that plants needed more energy to keep growing under heat stress.

### Proteins involved in energy and metabolism

Adequate adenosine triphosphate (ATP) is essential for plant responses to abiotic stress^32^. ATP synthase (spots 8 and 9) was upregulated when self-grafted plants were grown under heat stress, and suggested that a greater energy requirement for the degradation and biosynthesis of proteins^33^. Interestingly, ATP synthase was also up-regulated in plants grafting onto Momordica rootstock under control temperature, which indicated that the process of ATP biosynthesis was active. Nucleoside diphosphate kinase (NDPK) (spots 50 and 65) transfers phosphate groups of high energy between ATP and NDP, and provides energy for growth and development. In this study, NDPK was downregulated in self-grafted plants under heat stress, whereas it was upregulated in Momordica-grafted plants. This observation might indicate that self-grafted plants had severe damage, whereas plants grafted onto Momordica rootstock, grew more normally under heat stress and benefitted from NDPK, which provided sufficient energy. ACR11 (spot 18), which can control Fd-GOGAT levels, is a member of the ACT domain-containing protein family^34^. The variation trend of ACR11 was similar to that of ATP synthase, and suggesting that Fd-GOGAT levels were not influenced significantly by heat stress in Momordica-grafted plants.

Fructose-bisphosphate aldolase (FBA) and malate dehydrogenase (MDH) are involved in the tricarboxylic acid cycle^35, 36^. The general expression pattern of FBA (spots 31, 45, 58, and 59) was upregulated by heat stress in self-grafted plants, but downregulated in Momordica-grafted plants. Transketolase (TK) (spots 13, 14) participates in the pentose phosphate pathway^37^. The accumulation level of TK in self-grafted plants increased under heat stress compared to control, whereas it was down-regulated in plants grafted onto Momordica rootstock under heat stress, and indicated a relatively stable pentose phosphate pathway in Momordica-grafted plants. Enolase (ENO) (spot 73) is one of the most important enzymes related to glycolysis and it catalyzes the dehydration of 2-phosphoglycerate into phosphoenolpyruvate^38^. The expression of ENO was similar to TK. Salt stress induces the expression of ENO protein and attempts to generate more energy to cope with stress^39^, and suggests that ENO is involved in multiple stress responses.

### Proteins involved in defense response

ROS metabolism is a general response to various stresses^40^. Heat stress induces the accumulation of ROS, which damages cellular membranes and functional components. Therefore, plants have developed an antioxidant system to regulate ROS level. In this study, nine antioxidant-related protein spots were identified.

Abscisic stress ripening-like protein (ASR) (spots 5, 6, and 7), peroxidase (POD) (spot 16), 2-Cys peroxiredoxin (spot 40), catalase (CAT) (spots 68 and 69), and ascorbate peroxidase (APX) (spot 34) regulate ROS level when plants are under stress^41–43^. Peptide methionine sulfoxide reductase A1-like (MsrA) (spot 49) plays an important role in repairing oxidative damage^44^. Most of these proteins were induced by heat stress, whereas they were expressed equally in Momordica-grafted plants compared to self-grafted plants. These results indicated that ROS induced by heat stress in Momordica-grafted plants might act as a signaling molecule to activate other resistance pathways.

### Proteins related to protein and nucleic acid biosynthesis

In general, proteins were affected by abiotic stress^45^. Elongation factor G-2 (spot 12), which is involved in the initiation and elongation stage of mRNA translation and protein synthesis, was downregulated in self-grafted plants under heat stress^46^. The expression pattern of glycine-rich RNA-binding protein (spot 19) was similar to elongation factor G-2, which indicated that protein synthesis was inhibited by heat stress in self-grafted plants.

Actin-7 (spot 24) is not only a major cytoskeletal component in all eukaryotic cells, but also a nuclear protein that plays a role in gene transcription^47^. In the present study, it was downregulated in self-grafted plants when plants were under heat stress, which illustrated that heat stress disturbed the cellular homeostasis in self-grafted plants. Chloroplast gene expression is regulated at both the transcriptional and post-transcriptional level^48^. A number of chloroplast ribonucleoproteins (cpRNPs) are likely to be involved in post-transcriptional RNA modification processes, which are important steps in the regulation of chloroplast gene expression^49^. Ribonucleoprotein (spot 74) was not present in self-grafted plants under heat stress. The lack of accumulation suggests that the biosynthesis capacity of chloroplast proteins in self-grafted plants was weaker than Momordica-grafted plants under heat stress, which impacted the photosynthesis process. Serine hydroxymethyl transferase (SHMT) (spot 47), a key enzyme in the synthesis of serine, catalyzes the conversion of glycine to serine. In addition, glycine dehydrogenase (spot 76) participates in glycine, serine and threonine metabolism. Both SHMT and glycine dehydrogenase were induced by heat stress. Protease is involved in recognizing and removing abnormal proteins, and it is induced in response to stress conditions^45^. Protease Do-like 1 (spot 30) plays a precise role in proteolysis of specific proteins, and accelerated the degradation of misfolded/damaged proteins under many types of stress^50^, was upregulated by Momordica rootstock under optimal temperature. This phenomenon might be caused by grafting cucumbers onto another species, rather than grafting onto their own roots.

### Proteins related to molecular chaperone

Molecular chaperones prevent the formation of misfolded protein structures when cells are under normal conditions and they are exposed to stress, such as high temperature^51, 52^. Heat shock proteins (HSPs) are representative proteins that are induced when cells suffer heat stress^53^. In this study, HSP70 (spot 2) was highly upregulated under heat stress. Furthermore, small HSPs (sHSPs) prevent protein aggregation during abiotic stress, especially heat stress^54^. The sHSPs (spots 38 and 39) were upregulated by heat stress. These results indicated that HSPs bound to denatured and unfold proteins to refold them under heat stress. Moreover, calreticulin (spot 1), a major endoplasmic reticulum Ca^2+^ binding chaperone, plays an essential role in regulating intracellular Ca^2+^ homeostasis^55^. In our study, calreticulin was upregulated in Cs-42 °C, which suggested that Ca^2+^ homeostasis was affected in self-grafted plants under heat stress, but it was reduced in plants grafted onto Momordica rootstock.

### Proteins of cucumber leaves from Momordica organism

Proteins produced in rootstock are transported into scions in grafted plants^56^. PGIP protein in the wild-type scion tissue grafted onto PGIP-expressing genetically engineered rootstock caused the reduction of pathogen damage in scion tissues^57^. Nine protein spots may be derived from the Momordica rootstock. Among these protein spots, 4 spots (36, 37, 41, 55) were related to photosynthesis. Except for faint up-regulation of 36 spots, the other three accumulated significantly in Mc-42 °C. This observation indicated that these proteins were transferred to scion from Momordica rootstock under heat stress and enhanced heat stress tolerance by intensifying photosynthesis capacity.

The accumulation of some protein spots decreased in Momordica-grafted plants under heat stress. It is likely that proteins moved from Momordica rootstock to cucumber scions and affected proteins in cucumber leaves, which inhibited the accumulation of cucumber proteins. The accumulation of these proteins in Momordica rootstock-grafted plants was lower, because Momordica rootstock was less influenced by heat stress than cucumber. Thus, detection of the accumulation of these proteins was lower in Mc-42 °C compared to Cs-42 °C. In future research, we will focus on how the proteins in Momordica are transferred into cucumber leaves, and what is the mechanism of movement.

### Evaluation of current work and a hypothetical working process

Photosynthesis, which is an important process for energy production, is sensitive to a variety of environmental stresses, such as heat, cold, drought, and heavy metal^58^. Grafting with stress-tolerant rootstocks can alleviate stress-induced reduction of photosynthesis in scions. Sensitivity of photosynthesis to heat stress is decreased when tomato was grafted onto heat-tolerant rootstock^12^. The proteomics analyses revealed the accumulation of key enzymes included in biological processes that played important role in enhancement of salt tolerance of bottle gourd rootstock-grafted plants^59^. Similarly, our research focused on the proteomics of the positive roles of Momordica rootstock in the response of cucumber leaves to heat stress, especially the enzymes related to photosynthesis. Improved plant growth with increased biomass accumulation in cucumber plants grafted onto rootstock with specific tolerance has been reported^60–62^. In our study, biomass was increased by Momordica rootstock compared to self-grafted plants under heat stress. There is a subset of genes that were influenced by apple rootstock^63^. In our study, we found that grafting onto Momordica rootstock induced significant transcriptional changes in some photosynthesis related genes under normal temperature, and it significantly upregulated the transcripts of the genes under heat stress. The high temperature adopted in this study simulated the temperature found in Southern of China during the summer, which made our research practical and realistic. Also, the technique of grafting onto heat-resistant rootstocks applies to other species to enhance heat stress tolerance.

The number of protein spots was not much enough because of the limitation of gel. In the future, we will use isobaric tags for relative and absolute quantitation (iTRAQ) to better elucidate the mechanism of heat stress resistance induced by Momordica rootstock. As for the dual source of cucumber and Momordica, it may provide new angle of research to explore the mechanism of heat tolerance mediated by grafting.

## Conclusion

In summary, there was qualitative and quantitative modification of key proteins in cucumber induced by Momordica rootstock which promoted photosynthesis and growth of cucumber scions under heat stress. Momordica rootstock-grafted plants exhibited the ability to adapt to heat stress with higher photosynthesis and more accumulation of biomass than self-grafted plants after heat stress for 7 days. The heat stress tolerance of Momordica rootstock grafted plants was attributed to the expression of key enzymes related to photosynthesis primarily. Momordica rootstock enhanced the capacity of photosynthesis in the scion, which stabilized other processes, such as acid biosynthesis, defense response and expressed thermal tolerance. We concluded that through comparative proteomics, this study provides comprehensive insights to better understand the mechanism by which Momordica rootstock confers tolerance to elevated temperatures.

## Electronic supplementary material

(cucumber database) detailed match information for each gel spot

(momordica database) detailed match information for each gel spot

Primers used for qRT-PCR assays

2-DE and Western Blot

Dataset 1

Dataset 2

Dataset 3

Dataset 4

Dataset 5
