# Supplementary material for: Proteomic analysis of heat stress resistance of cucumber leaves when grafted onto Momordica rootstock
Source: Hortic Res. 2018 Oct 1;5:53. doi: 10.1038/s41438-018-0060-z (PMC6165847; doi:10.1038/s41438-018-0060-z)
Supplement: Supplementary file 1 — (cucumber database) detailed match information for each gel spot [file 41438_2018_60_MOESM1_ESM.pdf]

detailed match information for each gel spot（cucumis database）

Cucumis Database

spot No.	Protein Name	Species	Accession No.	Protein MW	Protein PI	Pep. Count	Protein Score	Protein Score	Total Ion Score	Total Ion C. I. %
1	hypothetical protein Csa_2G033940 [Cucumis sativus]		gi|700205871	48356.4	4.45	25	418	100	266	100
	**Protein Group**									
	PREDICTED: calreticulin [Cucumis sativus]		gi|449454026	48356.4	4.4499998092 6514					

Peptide Information

Calc. Mass	Obsrv. Mass	± da	± ppm	Start Seq.	End Seq.	Sequence	Ion Score	C. I. %	Modification	Rank	Result Type
826.4094	826.403	-0.0064	-8	26	31	VFFEER					Mascot
894.4203	894.4174	-0.0029	-3	362	369	AAFEEAEK					Mascot
896.3785	896.3801	0.0016	2	32	38	FDDGWEK					Mascot
942.4064	942.3993	-0.0071	-8	62	69	WHGDANDK					Mascot
974.4578	974.4266	-0.0312	-32	220	227	KPADWDDK					Mascot
974.5153	974.5093	-0.006	-6	117	125	LLSGDVDQK					Mascot
1016.4176	1016.4252	0.0076	7	108	116	LDCGGGYMK			Carbamidomethyl (C)[3], Oxidation (M)[8]		Mascot
1033.495	1033.4666	-0.0284	-27	347	355	QVAEDTWGK					Mascot
1052.4796	1052.4784	-0.0012	-1	32	39	FDDGWEKR					Mascot
1068.4956	1068.4968	0.0012	1	70	78	GIQTSEDYR					Mascot
1068.4956	1068.4968	0.0012	1	70	78	GIQTSEDYR	24	22.754			Mascot
1163.5402	1163.5522	0.012	10	297	306	APMIDNPDFK			Oxidation (M)[3]		Mascot
1222.5991	1222.5804	-0.0187	-15	307	316	DDPELYVFPK					Mascot

1275.6328	1275.717	0.0842	66	347	357	QVAEDTWGKNK					Mascot
1309.7151	1309.6929	-0.0222	-17	93	103	DNILVFQFSVK					Mascot
1566.7072	1566.689	-0.0182	-12	48	61	DESLAGEWNYTPGK					Mascot
1665.7795	1665.7731	-0.0064	-4	79	92	FYAISAEYPEFSNK					Mascot
1665.7795	1665.7731	-0.0064	-4	79	92	FYAISAEYPEFSNK	80	100			Mascot
1691.8639	1691.8263	-0.0376	-22	197	211	QTGSLYSDWSLLPPK					Mascot
1722.8082	1722.8199	0.0117	7	47	61	RDESLAGEWNYTPGK					Mascot
1722.8082	1722.8199	0.0117	7	47	61	RDESLAGEWNYTPGK	7	0			Mascot

1819.9589	1819.9305	-0.0284	-16	197	212	QTGSLYSDWSLLPPKK				Mascot
1997.9703	1997.9635	-0.0068	-3	329	346	SGTLFDNVLITDDPEYAK				Mascot
1997.9703	1997.9635	-0.0068	-3	329	346	SGTLFDNVLITDDPEYAK	109	100		Mascot
2044.9498	2044.9451	-0.0047	-2	220	236	KPADWDDKEFIPDPEDK				Mascot
2343.0308	2343.0522	0.0214	9	127	147	FGGETPYSIMFGPDICGYSTK			Carbamidomethyl (C)[16], Oxidation (M)[10]	Mascot
2367.1213	2367.1245	0.0032	1	297	316	APMIDNPDFKDDPELYVFPK			Oxidation (M)[3]	Mascot
2367.1213	2367.1245	0.0032	1	297	316	APMIDNPDFKDDPELYVFPK	46	99.393	Oxidation (M)[3]	Mascot
2471.1257	2471.146	0.0203	8	127	148	FGGETPYSIMFGPDICGYSTKK			Carbamidomethyl (C)[16], Oxidation (M)[10]	Mascot
3779.8838	3779.9805	0.0967	26	165	196	EVPCETDQLTHVYTFIIRPDATYSILIDNVEK			Carbamidomethyl (C)[4]	Mascot

2									
	Chloroplast HSP70 [Cucumis sativus]	gi|700206320	75350	5.18	35	784	100	585	100
	**Protein Group**								
	chloroplast HSP70 [Cucumis sativus]	gi|124245039	75350	5.17999982833862					
	heat shock protein 70 [Cucumis sativus]	gi|1143427	75366	5.15000009536743					
	stromal 70 kDa heat shock-related protein, chloroplastic [Cucumis sativus]	gi|821595433	75366	5.15000009536743					
	unnamed protein product [Cucumis sativus]	gi|257307177	75366	5.15000009536743					
	unnamed protein product [Cucumis sativus]	gi|257344387	75366	5.15000009536743					
	unnamed protein product [Cucumis sativus]	gi|219773901	75366	5.15000009536743					

unnamed protein product [Cucumis sativus]	gi|219764942	75366	5.1500<br>000953<br>6743

Peptide Information

Calc. Mass	Obsrv. Mass	± da	± ppm	Start Seq.	End Seq.	Sequence	Ion Score	C. I. %	Modification	Rank	Result Type
869.5091	869.5036	-0.0055	-6	532	539	GVPQIEVK					Mascot
870.5407	870.5431	0.0024	3	229	236	IAGLEVLR					Mascot
884.5676	884.5381	-0.0295	-33	118	125	LVGQIAKR					Mascot
887.4655	887.4686	0.0031	3	170	177	LECPAIGK			Carbamidomethyl (C)[3]		Mascot
902.4829	902.4713	-0.0116	-13	311	318	DEGIDLLK					Mascot
915.4894	915.486	-0.0034	-4	382	389	TPVENSLR					Mascot
935.5309	935.5272	-0.0037	-4	523	531	LDGIPPAPR					Mascot
970.5316	970.5325	0.0009	1	359	366	HIETTITR					Mascot
996.6088	996.6006	-0.0082	-8	410	418	IPAVQELVK					Mascot
1066.578	1066.5394	-0.0386	-36	104	113	TTPSVVAYTK					Mascot
1125.5609	1125.5648	0.0039	3	579	588	MVSEADKFAK					Mascot
1138.6467	1138.6162	-0.0305	-27	616	626	ELGDKVPGPVK					Mascot

1141.5558	1141.5576	0.0018	2	579	588	MVSEADKFAK			Oxidation (M)[1]		Mascot
1156.6685	1156.6686	0.0001	0	380	389	LKTPVENSLR					Mascot
1162.5951	1162.5848	-0.0103	-9	638	649	EAISGGSTEAIK					Mascot
1170.6589	1170.6273	-0.0316	-27	114	124	NGDRLVGQIAK					Mascot
1176.6412	1176.6232	-0.018	-15	300	309	IVDWLAANFK					Mascot
1197.5304	1197.6011	0.0707	59	145	154	KMSEVDEESK			Oxidation (M)[2]		Mascot
1364.7057	1364.7321	0.0264	19	192	203	LVDDASKFLNDK					Mascot
1373.7272	1373.7292	0.002	1	397	409	DIDEVILVGGSTR					Mascot
1373.7272	1373.7292	0.002	1	397	409	DIDEVILVGGSTR	131	100			Mascot
1381.6594	1381.6488	-0.0106	-8	601	612	NQADSVVYQTEK					Mascot
1387.754	1387.7264	-0.0276	-20	321	332	QALQRLTETAEK					Mascot
1396.6414	1396.6608	0.0194	14	369	379	FEELCSDLLDR			Carbamidomethyl (C)[5]		Mascot
1461.7697	1461.7676	-0.0021	-1	178	190	QFAAEEISAQVLR					Mascot
1461.7697	1461.7676	-0.0021	-1	178	190	QFAAEEISAQVLR	129	100			Mascot
1551.681	1551.7307	0.0497	32	693	707	GPEGDVIDADFSDSK					Mascot
1556.7816	1556.7899	0.0083	5	24	37	SNNVSSRSVFFGQK					Mascot

1566.7911	1566.7914	0.0003	0	207	220	AVVTVPAYFNDSQR					Mascot
1566.7911	1566.7914	0.0003	0	207	220	AVVTVPAYFNDSQR	87	100			Mascot
1579.8115	1579.7915	-0.02	-13	126	139	QAVVNPENTFFSVK					Mascot
1579.8115	1579.7915	-0.02	-13	126	139	QAVVNPENTFFSVK	46	99.352			Mascot
1589.8646	1589.767	-0.0976	-61	178	191	QFAAEEISAQVLRK					Mascot
1595.7734	1595.7772	0.0038	2	367	379	AKFEELCSDLLDR			Carbamidomethyl (C)[7]		Mascot
1595.7734	1595.7772	0.0038	2	367	379	AKFEELCSDLLDR	29	70.603	Carbamidomethyl (C)[7]		Mascot
1723.8901	1723.8685	-0.0216	-13	237	252	IINEPTAASLAYGFEK					Mascot
1723.8901	1723.8685	-0.0216	-13	237	252	IINEPTAASLAYGFEK	97	100			Mascot
1735.9126	1735.9193	0.0067	4	126	140	QAVVNPENTFFSVKR					Mascot
1931.9557	1931.9467	-0.009	-5	561	578	QDITITGASTLPSDEVER					Mascot
2060.0505	2060.0542	0.0037	2	560	578	KQDITITGASTLPSDEVER					Mascot
2060.0505	2060.0542	0.0037	2	560	578	KQDITITGASTLPSDEVER	53	99.88			Mascot
2437.1841	2437.2068	0.0227	9	487	509	SEVFSTAADGQTSVEINV LQGER					Mascot
2437.1841	2437.2068	0.0227	9	487	509	SEVFSTAADGQTSVEINV LQGER	13	0			Mascot
3056.5681	3056.6094	0.0413	14	73	103	VVGIDLGTTNSAVAAMEG GKPTIVTNAEGQR					Mascot
3072.563	3072.5989	0.0359	12	73	103	VVGIDLGTTNSAVAAMEG GKPTIVTNAEGQR			Oxidation (M)[16]		Mascot

3

Protein disulfide-isomerase [Cucumis sativus]	gi|700192511	57045.6	4.88		35	879	100	639	100

Protein Group

PREDICTED: protein disulfide-isomerase [Cucumis sativus]	gi|449464162	57045.6	4.8800 001144 4092

Peptide Information

Calc. Mass	Obsrv. Mass	± da	± ppm	Start Seq.	End Seq.	Sequence	Ion Score	C. I. %	Modification	Rank	Result Type
866.4254	866.4174	-0.008	-9	291	297	EVAEQYK					Mascot
914.44	914.3812	-0.0588	-64	1	8	MASSSFIR			Oxidation (M)[1]		Mascot
931.4156	931.3969	-0.0187	-20	263	270	FFSSSNDK					Mascot
936.4672	936.4448	-0.0224	-24	67	74	SLAPEYEK					Mascot

965.4686	965.4527	-0.0159	-16	461	468	LVEYNGDR			Mascot
987.5105	987.4581	-0.0524	-53	142	151	QSGPASAEIK			Mascot
1021.4796	1021.521	0.0414	41	499	507	ESKQDSEAK			Mascot
1033.4545	1033.4541	-0.0004	0	91	99	VDANEESNR			Mascot
1037.5011	1037.4669	-0.0342	-33	122	130	SSQDYKGPR			Mascot
1047.5317	1047.4857	-0.046	-44	152	161	SAEDASNLIK			Mascot
1087.5743	1087.5681	-0.0062	-6	205	215	GETSVSGPVVR			Mascot
1106.5841	1106.5704	-0.0137	-12	100	108	ELATQFEIR			Mascot
1106.5841	1106.5704	-0.0137	-12	100	108	ELATQFEIR	59	99.97	Mascot
1115.6056	1115.6006	-0.005	-4	141	151	KQSGPASAEIK			Mascot
1150.6508	1150.6235	-0.0273	-24	162	171	DVYIVGIFPK			Mascot
1150.6508	1150.6235	-0.0273	-24	162	171	DVYIVGIFPK	82	100	Mascot
1170.5426	1170.5253	-0.0173	-15	172	181	LSGDEFNNFK			Mascot
1170.5426	1170.5253	-0.0173	-15	172	181	LSGDEFNNFK	61	99.981	Mascot
1185.5898	1185.5758	-0.014	-12	289	297	YREVAEQYK			Mascot
1267.7257	1267.6875	-0.0382	-30	321	331	EDQVPVLLVQK			Mascot
1363.6852	1363.673	-0.0122	-9	471	481	EDIINFIETNR			Mascot
1382.6223	1382.5966	-0.0257	-19	189	200	TDYDFGHTLDAK			Mascot
1382.6223	1382.5966	-0.0257	-19	189	200	TDYDFGHTLDAK	57	99.952	Mascot
1434.7839	1434.7418	-0.0421	-29	378	390	VVVADSIQDVVYK	52	99.817	Mascot

1439.7013	1439.6694	-0.0319	-22	365	377	SEPIPESNNEPVK			Mascot
1516.7795	1516.7483	-0.0312	-21	340	352	FNVEADQIAPWVK			Mascot
1566.8962	1566.7706	-0.1256	-80	201	215	LLPRGETSVSGPVVR			Mascot
1578.8123	1578.7986	-0.0137	-9	469	481	SKEDIINFIETNR			Mascot
1578.8123	1578.7986	-0.0137	-9	469	481	SKEDIINFIETNR	99	100	Mascot
1606.8071	1606.8245	0.0174	11	471	483	EDIINFIETNRDK			Mascot
1651.8075	1651.7915	-0.016	-10	187	200	LRTDYDFGHTLDAK			Mascot
1659.8418	1659.8221	-0.0197	-12	216	228	LFKPFDEQFVDFK			Mascot
1659.8418	1659.8221	-0.0197	-12	216	228	LFKPFDEQFVDFK	52	99.833	Mascot
1674.8545	1674.8112	-0.0433	-26	484	498	TAEDTKPKDTESKPK			Mascot
1821.8728	1821.8781	0.0053	3	271	286	AMFFLNYTTEAADSLK			Mascot
1913.9491	1913.8822	-0.0669	-35	33	49	EFVLTLDNSNFSDVVSK			Mascot
2331.1965	2331.1677	-0.0288	-12	410	431	LAPTLDEVAVSYESDPDV VIAK			Mascot

2446.2136	2446.1914	-0.0222	-9	298	320	GEISFLIGDSESSQAALN YFGLK					Mascot
2459.2915	2459.2671	-0.0244	-10	409	431	KLAPTLDEVAVSYESDPD VVIAK					Mascot
2709.2832	2709.3083	0.0251	9	432	455	FDATANDIAVGDFEVQGY PTLYFR					Mascot
2709.2832	2709.3083	0.0251	9	432	455	FDATANDIAVGDFEVQGY PTLYFR	176	100			Mascot
2825.3628	2825.3733	0.0105	4	238	262	FIESSSIPTVTEFNNDPSN HVYLSK					Mascot

4

hypothetical protein Csa_6G147530 [Cucumis sativus]	gi|700191656	61399.9	5.06	30	732	100	555	100

Protein Group

PREDICTED: ruBisCO large subunit-binding protein subunit alpha [Cucumis sativus]	gi|449456032	61399.9	5.0599 999427 7954

Peptide Information

Calc. Mass	Obsrv. Mass	± da	± ppm	Start Seq.	End Seq.	Sequence	Ion Score	C. I. %	Modification	Rank	Result Type
816.4825	816.4581	-0.0244	-30	262	268	VLITDQK					Mascot
857.5091	857.5034	-0.0057	-7	482	489	LGADIIQK					Mascot
898.572	898.5627	-0.0093	-10	311	319	GILNVAAIK					Mascot
940.5713	940.5544	-0.0169	-18	274	281	DIIPILEK					Mascot
989.4786	989.4758	-0.0028	-3	179	187	AIEGEDDIK					Mascot
1028.5735	1028.5629	-0.0106	-10	253	261	LIAELENAR					Mascot
1028.5735	1028.5629	-0.0106	-10	253	261	LIAELENAR	30	75.115			Mascot
1043.5845	1043.5795	-0.005	-5	91	100	VVNDGVTIAR					Mascot
1052.5007	1052.4911	-0.0096	-9	48	56	EIAFDQSSR					Mascot
1052.5007	1052.4911	-0.0096	-9	48	56	EIAFDQSSR	24	18.355			Mascot
1076.5106	1076.4817	-0.0289	-27	473	481	DKLEDAEEK					Mascot
1181.7001	1181.6901	-0.01	-8	66	77	LANAVGLTLGPR					Mascot
1181.7001	1181.6901	-0.01	-8	66	77	LANAVGLTLGPR	44	99.202			Mascot
1204.6208	1204.6017	-0.0191	-16	80	90	NVVLDEFGSPK					Mascot
1258.689	1258.663	-0.026	-21	165	175	TVQGLIEELEK					Mascot
1290.6172	1290.6205	0.0033	3	423	434	VGAATETELEDR					Mascot

1386.7839	1386.744	-0.0399	-29	165	176	TVQGLIEELEKK				Mascot
1409.7019	1409.6786	-0.0233	-17	44	56	ASAKEIAFDQSSR				Mascot
1418.7122	1418.6953	-0.0169	-12	423	435	VGAATETELEDRK				Mascot
1479.7479	1479.7203	-0.0276	-19	240	252	GYISPQFVTNPEK				Mascot
1479.7479	1479.7203	-0.0276	-19	240	252	GYISPQFVTNPEK	86	100		Mascot
1499.6748	1499.6497	-0.0251	-17	394	406	ELAETDSVYDTEK				Mascot
1517.7847	1517.7446	-0.0401	-26	529	542	YENLVEAGVIDPAK				Mascot
1582.9164	1582.8813	-0.0351	-22	144	159	LGLLNVTSGANPVSLK				Mascot

1582.9164	1582.8813	-0.0351	-22	144	159	LGLLNVTSGANPVSLK	37	95.461		Mascot
1636.7773	1636.7859	0.0086	5	123	139	TNDSAGDGTTTASVLAR				Mascot
1671.9164	1671.9009	-0.0155	-9	161	175	GIDKTVQGLIEELEK				Mascot
1739.0175	1739.0044	-0.0131	-8	144	160	LGLLNVTSGANPVSLKR				Mascot
1780.9261	1780.8928	-0.0333	-19	101	117	AIELPDPMENAGAALIR				Mascot
1796.9211	1796.8903	-0.0308	-17	101	117	AIELPDPMENAGAALIR			Oxidation (M)[8]	Mascot
1796.9211	1796.8903	-0.0308	-17	101	117	AIELPDPMENAGAALIR	96	100	Oxidation (M)[8]	Mascot
1918.9717	1918.9612	-0.0105	-5	370	387	DTTTIIADAASKDELQAR				Mascot
1918.9717	1918.9612	-0.0105	-5	370	387	DTTTIIADAASKDELQAR	127	100		Mascot
2245.1743	2245.1235	-0.0508	-23	188	210	AVASISAGNDELIGLMIAD<br>AIGK			Oxidation (M)[16]	Mascot
2250.2957	2250.2429	-0.0528	-23	287	308	APLLIIAEDVTGEALATLV<br>VNK				Mascot
2278.2654	2278.2341	-0.0313	-14	490	512	ALVAPASLIAQNAGIEGE<br>VVVEK				Mascot
2278.2654	2278.2341	-0.0313	-14	490	512	ALVAPASLIAQNAGIEGE<br>VVVEK	79	100		Mascot
2916.6194	2916.625	0.0056	2	443	472	NATFAAIEEGIVPGGGAA<br>LVHLSTLVPAIK				Mascot
3042.4097	3042.4182	0.0085	3	211	239	VGPDGVLSIESSSSFETT<br>VDVEEGMAIDR			Oxidation (M)[25]	Mascot
3042.4097	3042.4182	0.0085	3	211	239	VGPDGVLSIESSSSFETT<br>VDVEEGMAIDR	32	87.296	Oxidation (M)[25]	Mascot

5

Abscisic stress ripening-like protein [Cucumis sativus]	gi|700190659	31723.4	5.02	19	487	100	359	100

Protein Group

PREDICTED: glycine-rich cell wall structural protein 1.8-like [Cucumis sativus]	gi|449441220	31723.4	5.0199 999809 2651

Peptide Information

Calc. Mass	Obsrv. Mass	± da	± ppm	Start Seq.	End Seq.	Sequence	Ion Score	C. I. %	Modification	Rank	Result Type
853.3937	853.3473	-0.0464	-54	77	83	YGELEDK					Mascot
966.4023	966.3778	-0.0245	-25	237	244	EDSEHGHR					Mascot
1073.4535	1073.4254	-0.0281	-26	110	118	VDEYGDGYR					Mascot
1073.4535	1073.4254	-0.0281	-26	110	118	VDEYGDGYR	18	0			Mascot
1201.5485	1201.5221	-0.0264	-22	110	119	VDEYGDGYRK					Mascot
1231.5563	1231.5337	-0.0226	-18	237	246	EDSEHGHRHK					Mascot
1290.5485	1290.5155	-0.033	-26	152	162	KEDEYDGGYSK					Mascot
1309.6271	1309.5863	-0.0408	-31	98	109	VATEYGGQLEDK					Mascot
1317.5343	1317.5059	-0.0284	-22	139	151	GGEYGGGYGESER					Mascot
1317.5343	1317.5059	-0.0284	-22	139	151	GGEYGGGYGESER	35	94.192			Mascot
1364.5699	1364.5662	-0.0037	-3	269	279	EKEEAEEEDEK					Mascot
1401.6282	1401.6086	-0.0196	-14	84	97	AGEYGGGYGLSENK					Mascot
1401.6282	1401.6086	-0.0196	-14	84	97	AGEYGGGYGLSENK	89	100			Mascot
1431.636	1431.5999	-0.0361	-25	233	244	HEAKEDSEHGHR					Mascot
1445.6292	1445.5958	-0.0334	-23	139	152	GGEYGGGYGESERK					Mascot
1489.6442	1489.6053	-0.0389	-26	124	138	VTAEYGGGYGESGDK					Mascot
1538.687	1538.6412	-0.0458	-30	55	69	VAAEYGGGYGESHNK					Mascot
1538.687	1538.6412	-0.0458	-30	55	69	VAAEYGGGYGESHNK	106	100			Mascot
2128.0935	2128.0623	-0.0312	-15	212	232	HLEHLGELGAAGAGAFA LHEK					Mascot
2364.0627	2364.0364	-0.0263	-11	98	118	VATEYGGQLEDKVDEYG DGYR					Mascot
2364.0627	2364.0364	-0.0263	-11	98	118	VATEYGGQLEDKVDEYG DGYR	78	100			Mascot
2546.2898	2546.2344	-0.0554	-22	245	268	HKLEEEIAAVAAVGAGGF AFHEHR					Mascot
2787.2117	2787.1816	-0.0301	-11	29	54	TSTYSEDLSEDKLDAYGS					Mascot

AYGGSENK

2788.1606	2788.1655	0.0049	2	124	151	VTAEYGGGYGESGDKG GEYGGGYGESER					Mascot
2788.1606	2788.1655	0.0049	2	124	151	VTAEYGGGYGESGDKG GEYGGGYGESER	36	95.136			Mascot

6

Abscisic stress ripening-like protein [Cucumis sativus]	gi|700190659	31723.4	5.02	21	621	100	471	100

Protein Group

PREDICTED: glycine-rich cell wall structural protein 1.8-like [Cucumis sativus]	gi|449441220	31723.4	5.0199 999809 2651

Peptide Information

Calc. Mass	Obsrv. Mass	± da	± ppm	Start Seq.	End Seq.	Sequence	Ion Score	C. I. %	Modification	Rank	Result Type
818.442	818.4029	-0.0391	-48	284	289	KHHHIF					Mascot
853.3937	853.3635	-0.0302	-35	77	83	YGELEDK					Mascot
966.4023	966.3837	-0.0186	-19	237	244	EDSEHGHR					Mascot
1073.4535	1073.4365	-0.017	-16	110	118	VDEYGDGYR					Mascot
1073.4535	1073.4365	-0.017	-16	110	118	VDEYGDGYR	18	0			Mascot
1107.4324	1107.5198	0.0874	79	271	279	EEAEEEDEK					Mascot
1201.5485	1201.5343	-0.0142	-12	110	119	VDEYGDGYRK					Mascot
1231.5563	1231.5505	-0.0058	-5	237	246	EDSEHGHRHK					Mascot
1290.5485	1290.5293	-0.0192	-15	152	162	KEDEYDGGYSK					Mascot
1309.6271	1309.5907	-0.0364	-28	98	109	VATEYGGQLEDK					Mascot
1317.5343	1317.5154	-0.0189	-14	139	151	GGEYGGGYGESER					Mascot
1317.5343	1317.5154	-0.0189	-14	139	151	GGEYGGGYGESER	49	99.731			Mascot
1401.6282	1401.616	-0.0122	-9	84	97	AGEYGGGYGLSENK					Mascot
1401.6282	1401.616	-0.0122	-9	84	97	AGEYGGGYGLSENK	87	100			Mascot
1431.636	1431.6147	-0.0213	-15	233	244	HEAKEDSEHGHR					Mascot
1445.6292	1445.608	-0.0212	-15	139	152	GGEYGGGYGESERK					Mascot
1489.6442	1489.6094	-0.0348	-23	124	138	VTAEYGGGYGESGDK					Mascot
1489.6442	1489.6094	-0.0348	-23	124	138	VTAEYGGGYGESGDK	60	99.98			Mascot

1538.687	1538.6478	-0.0392	-25	55	69	VAAEYGGGYGESHNK					Mascot
1538.687	1538.6478	-0.0392	-25	55	69	VAAEYGGGYGESHNK	120	100			Mascot
2128.0935	2128.0623	-0.0312	-15	212	232	HLEHLGELGAAGAGAFA LHEK					Mascot
2128.0935	2128.0623	-0.0312	-15	212	232	HLEHLGELGAAGAGAFA LHEK	13	0			Mascot
2281.136	2281.1082	-0.0278	-12	247	268	LEEEIAAVAAVGAGGFAF HEHR					Mascot

2364.0627	2364.0469	-0.0158	-7	98	118	VATEYGGQLEDKVDEYG DGYR					Mascot
2364.0627	2364.0469	-0.0158	-7	98	118	VATEYGGQLEDKVDEYG DGYR	78	100			Mascot
2546.2898	2546.2815	-0.0083	-3	245	268	HKLEEEIAAVAAVGAGGF AFHEHR					Mascot
2546.2898	2546.2815	-0.0083	-3	245	268	HKLEEEIAAVAAVGAGGF AFHEHR	48	99.676			Mascot
2787.2117	2787.1577	-0.054	-19	29	54	TSTYSEDLSEDKLDAYGS AYGGSENK					Mascot
2788.1606	2788.1616	0.001	0	124	151	VTAEYGGGYGESGDKG GEYGGGYGESER					Mascot

7

Abscisic stress ripening-like protein [Cucumis sativus]	gi|700190659	31723.4	5.02	21	832	100	681	100

Protein Group

PREDICTED: glycine-rich cell wall structural protein 1.8-like [Cucumis sativus]	gi|449441220	31723.4	5.0199 999809 2651

Peptide Information

Calc. Mass	Obsrv. Mass	± da	± ppm	Start Seq.	End Seq.	Sequence	Ion Score	C. I. %	Modification	Rank	Result Type
818.442	818.4151	-0.0269	-33	284	289	KHHHIF					Mascot
966.4023	966.3855	-0.0168	-17	237	244	EDSEHGHR					Mascot
1073.4535	1073.4274	-0.0261	-24	110	118	VDEYGDGYR					Mascot

1201.5485	1201.5145	-0.034	-28	110	119	VDEYGDGYRK				Mascot
1231.5563	1231.5532	-0.0031	-3	237	246	EDSEHGHRHK				Mascot
1290.5485	1290.5283	-0.0202	-16	152	162	KEDEYDGGYSK				Mascot
1309.6271	1309.5952	-0.0319	-24	98	109	VATEYGGQLEDK				Mascot
1317.5343	1317.5116	-0.0227	-17	139	151	GGEYGGGYGESER				Mascot
1317.5343	1317.5116	-0.0227	-17	139	151	GGEYGGGYGESER	69	99.998		Mascot
1364.5699	1364.5654	-0.0045	-3	269	279	EKEEAEEEDEK				Mascot
1401.6282	1401.6211	-0.0071	-5	84	97	AGEYGGGYGLSENK				Mascot
1401.6282	1401.6211	-0.0071	-5	84	97	AGEYGGGYGLSENK	79	100		Mascot
1431.636	1431.5978	-0.0382	-27	233	244	HEAKEDSEHGHR				Mascot
1445.6292	1445.6139	-0.0153	-11	139	152	GGEYGGGYGESERK				Mascot
1489.6442	1489.5973	-0.0469	-31	124	138	VTAEYGGGYGESGDK				Mascot
1538.687	1538.6447	-0.0423	-27	55	69	VAAEYGGGYGESHNK				Mascot
1538.687	1538.6447	-0.0423	-27	55	69	VAAEYGGGYGESHNK	135	100		Mascot
2070.8232	2070.7444	-0.0788	-38	167	186	SGEYGGGMEEEGGEYG<br>GVYK			Oxidation (M)[8]	Mascot
2128.0935	2128.0469	-0.0466	-22	212	232	HLEHLGELGAAGAGAFA<br>LHEK				Mascot
2128.0935	2128.0469	-0.0466	-22	212	232	HLEHLGELGAAGAGAFA<br>LHEK	13	0		Mascot
2281.136	2281.1035	-0.0325	-14	247	268	LEEEIAAVAAVGAGGFAF<br>HEHR				Mascot
2364.0627	2364.0225	-0.0402	-17	98	118	VATEYGGQLEDKVDEYG<br>DGYR				Mascot
2364.0627	2364.0225	-0.0402	-17	98	118	VATEYGGQLEDKVDEYG<br>DGYR	128	100		Mascot

2546.2898	2546.2615	-0.0283	-11	245	268	HKLEEEIAAVAAVGAGGF<br>AFHEHR				Mascot
2546.2898	2546.2615	-0.0283	-11	245	268	HKLEEEIAAVAAVGAGGF<br>AFHEHR	182	100		Mascot
2787.2117	2787.1558	-0.0559	-20	29	54	TSTYSEDLSEDKLDAYGS<br>AYGGSENK				Mascot
2788.1606	2788.1428	-0.0178	-6	124	151	VTAEYGGGYGESGDKG<br>GEYGGGYGESER				Mascot

2788.1606	2788.1428	-0.0178	-6	124	151	VTAEYGGGYGESGDKG GEYGGGYGESER	76	100	Mascot

8

ATP synthase CF1 beta subunit (plastid) [Cucumis hystrix]	gi|590000422	53814	5.11	28	904	100	725	100	

Protein Group

ATP synthase CF1 beta subunit (chloroplast) [Cucumis sativus]	gi|115432811	53814	5.1100 001335 144
ATP synthase CF1 beta subunit (plastid) [Cucumis hystrix]	gi|586598693	53814	5.1100 001335 144
ATPase beta subunit (chloroplast) [Cucumis sativus]	gi|74027108	53814	5.1100 001335 144
RecName: Full=ATP synthase subunit beta, chloroplastic; AltName: Full=ATP synthase F1 sector subunit beta; AltName: Full=F-ATPase subunit beta	gi|158518590	53814	5.1100 001335 144

Peptide Information

Calc. Mass	Obsrv. Mass	± da	± ppm	Start Seq.	End Seq.	Sequence	Ion Score	C. I. %	Modification	Rank	Result Type
811.4421	811.4156	-0.0265	-33	128	134	TTSPIHR					Mascot
922.4299	922.3916	-0.0383	-42	88	95	GMEVVDTR			Oxidation (M)[2]		Mascot
975.5622	975.5359	-0.0263	-27	168	178	IGLFGGAGVGK					Mascot
1007.5771	1007.5505	-0.0266	-26	146	154	LSIFETGIK					Mascot
1021.5676	1021.5101	-0.0575	-56	448	456	YVGLAETIR					Mascot
1190.6416	1190.6094	-0.0322	-27	135	145	SAPAFIQLDTK					Mascot
1201.7052	1201.6562	-0.049	-41	155	164	VVDLLAPYRR					Mascot
1208.594	1208.5601	-0.0339	-28	76	87	AVAMSATDGLTR			Oxidation (M)[4]		Mascot
1261.5076	1261.4552	-0.0524	-42	208	217	EGNDLYMEMK			Oxidation (M)[7,9]		Mascot
1265.6042	1265.5576	-0.0466	-37	487	497	ATNLEMESNLK			Oxidation (M)[6]		Mascot
1275.713	1275.6945	-0.0185	-15	40	50	MPNIYNALIVK					Mascot
1291.7079	1291.6473	-0.0606	-47	40	50	MPNIYNALIVK			Oxidation (M)[1]		Mascot
1294.7478	1294.7219	-0.0259	-20	96	109	APLSVPVGGATLGR					Mascot

1294.7478	1294.7219	-0.0259	-20	96	109	APLSVPVGGATLGR	86	100		Mascot
1328.6707	1328.6409	-0.0298	-22	192	205	AHGGVSVFGGVGER				Mascot
1328.6707	1328.6409	-0.0298	-22	192	205	AHGGVSVFGGVGER	109	100		Mascot
1431.6863	1431.6595	-0.0268	-19	379	390	IVGEEHYETAQR				Mascot

1431.6863	1431.6595	-0.0268	-19	379	390	IVGEEHYETAQR	54	99.915		Mascot
1433.7748	1433.7208	-0.054	-38	278	291	FVQAGSEVSALLGR				Mascot
1471.7614	1471.7289	-0.0325	-22	249	261	VGLTALTMAEYFR				Mascot
1487.7563	1487.7051	-0.0512	-34	249	261	VGLTALTMAEYFR			Oxidation (M)[8]	Mascot
1487.7563	1487.7051	-0.0512	-34	249	261	VGLTALTMAEYFR	29	69.006	Oxidation (M)[8]	Mascot
1502.6614	1502.7126	0.0512	34	206	217	TREGNDLYMEMK			Oxidation (M)[9]	Mascot
1518.6564	1518.6138	-0.0426	-28	206	217	TREGNDLYMEMK			Oxidation (M)[9,11]	Mascot
1552.7125	1552.6703	-0.0422	-27	218	231	ESGVINEENFAESK				Mascot
1601.8104	1601.7762	-0.0342	-21	232	246	VALVYGQMNEPPGAR				Mascot
1617.8054	1617.7458	-0.0596	-37	232	246	VALVYGQMNEPPGAR			Oxidation (M)[8]	Mascot
1617.8054	1617.7458	-0.0596	-37	232	246	VALVYGQMNEPPGAR	45	99.314	Oxidation (M)[8]	Mascot
1735.0154	1734.9369	-0.0785	-45	23	39	IAQIIGPVLDVAFPPGK				Mascot
1949.9968	1949.9683	-0.0285	-15	262	277	DVNEQDVLLFIDNIFR				Mascot
1969.0389	1968.9929	-0.046	-23	110	127	IFNVLGEPIDNLGPVDTR				Mascot
1969.0389	1968.9929	-0.046	-23	110	127	IFNVLGEPIDNLGPVDTR	155	100		Mascot
1983.0658	1983.0117	-0.0541	-27	128	145	TTSPIHRSAPAFIQLDTK				Mascot
2061.0322	2060.9807	-0.0515	-25	360	378	GIYPAVDPLDSTSTMLQP<br>R				Mascot
2077.0271	2076.9563	-0.0708	-34	360	378	GIYPAVDPLDSTSTMLQP<br>R			Oxidation (M)[15]	Mascot
2077.0271	2076.9563	-0.0708	-34	360	378	GIYPAVDPLDSTSTMLQP<br>R	37	94.938	Oxidation (M)[15]	Mascot
2171.1079	2171.0229	-0.085	-39	400	418	ELQDIIAILGLDELSEEDR				Mascot
2282.0791	2282.0476	-0.0315	-14	292	312	MPSAVGYQPTLSTEMGS<br>LQER				Mascot
2289.3079	2289.1628	-0.1451	-63	18	39	NNLGRIAQIIGPVLDVAFP<br>PGK				Mascot
2298.074	2298.0217	-0.0523	-23	292	312	MPSAVGYQPTLSTEMGS<br>LQER			Oxidation (M)[1]	Mascot
2314.0691	2313.9998	-0.0693	-30	292	312	MPSAVGYQPTLSTEMGS			Oxidation (M)[1,15]	Mascot

Calc. Mass	Obsrv. Mass	± da	± ppm	Start Seq.	End Seq.	Sequence	Ion Score	C. I. %	Modification	Rank	Result Type
						LQER					
2314.0691	2313.9998	-0.0693	-30	292	312	MPSAVGYQPTLSTEMGS LQER	12	0	Oxidation (M)[1,15]		Mascot
2572.2419	2572.2068	-0.0351	-14	51	73	GQDISGQEINVTCEVQQL LGNNR			Carbamidomethyl (C)[13]		Mascot
2572.2419	2572.2068	-0.0351	-14	51	73	GQDISGQEINVTCEVQQL LGNNR	200	100	Carbamidomethyl (C)[13]		Mascot

9

hypothetical protein Csa_5G576620 [Cucumis sativus]	gi|700196343	60068.4	5.9	30	1,020	100	831	100

Protein Group

PREDICTED: ATP synthase subunit beta, mitochondrial-like [Cucumis sativus]	gi|449465916	60068.4	5.9000000953 6743

Peptide Information

Calc. Mass	Obsrv. Mass	± da	± ppm	Start Seq.	End Seq.	Sequence	Ion Score	C. I. %	Modification	Rank	Result Type
866.4003	866.3782	-0.0221	-26	271	277	EGNDLYR					Mascot
975.5622	975.5383	-0.0239	-24	231	241	IGLFGGAGVGK					Mascot
1005.5285	1005.5657	0.0372	37	278	286	EMIESGVIK					Mascot
1021.5234	1021.488	-0.0354	-35	278	286	EMIESGVIK			Oxidation (M)[2]		Mascot
1151.5957	1151.5656	-0.0301	-26	189	197	TDHYLPIHR					Mascot
1173.6627	1173.635	-0.0277	-24	218	227	VVDLLAPYQR					Mascot
1173.6627	1173.635	-0.0277	-24	218	227	VVDLLAPYQR	64	99.99			Mascot
1278.6359	1278.6239	-0.012	-9	139	150	TIAMDGTEGLVR			Oxidation (M)[4]		Mascot
1278.6359	1278.7483	0.1124	88	139	150	TIAMDGTEGLVR			Oxidation (M)[4]		Mascot
1339.7468	1339.7053	-0.0415	-31	173	184	IINVIGEPIDEK					Mascot
1380.7006	1380.6578	-0.0428	-31	517	529	ESITSFQGVLDGK					Mascot
1390.6863	1390.6575	-0.0288	-21	255	268	AHGGFSVFAGVGER					Mascot
1390.6863	1390.6575	-0.0288	-21	255	268	AHGGFSVFAGVGER	124	100			Mascot
1399.7693	1399.7395	-0.0298	-21	313	325	VGLTGLTVAEHFR					Mascot
1399.7693	1399.7395	-0.0298	-21	313	325	VGLTGLTVAEHFR	102	100			Mascot
1409.8112	1409.7817	-0.0295	-21	154	167	VLNTGSPITVPVGR					Mascot
1409.8112	1409.7817	-0.0295	-21	154	167	VLNTGSPITVPVGR	71	99.998			Mascot
1457.8396	1457.7526	-0.087	-60	242	254	TVLIMELINNVAK					Mascot

1473.8346	1473.7496	-0.085	-58	242	254	TVLIMELINNVAK			Oxidation (M)[5]	Mascot
1492.7755	1492.7417	-0.0338	-23	342	355	FTQANSEVSALLGR				Mascot
1492.7755	1492.7417	-0.0338	-23	342	355	FTQANSEVSALLGR	35	92.37		Mascot
1565.9122	1565.8624	-0.0498	-32	153	167	RVLNTGSPITVPVGR				Mascot
1621.7462	1621.7523	0.0061	4	296	310	CALVYGQMNEPPGAR			Oxidation (M)[8]	Mascot
1678.7676	1678.718	-0.0496	-30	296	310	CALVYGQMNEPPGAR			Carbamidomethyl (C)[1], Oxidation (M)[8]	Mascot

1678.7676	1678.718	-0.0496	-30	296	310	CALVYGQMNEPPGAR			Carbamidomethyl (C)[1], Oxidation (M)[8]	Mascot
1707.921	1707.8774	-0.0436	-26	124	138	LVLEVAQHLGENMVR				Mascot
1723.916	1723.8597	-0.0563	-33	124	138	LVLEVAQHLGENMVR			Oxidation (M)[13]	Mascot
1723.916	1723.8597	-0.0563	-33	124	138	LVLEVAQHLGENMVR			Oxidation (M)[13]	Mascot
1752.9742	1752.8862	-0.088	-50	173	188	IINVIGEPIDEKGDLK				Mascot
1853.8964	1853.824	-0.0724	-39	438	453	MLSPHILGEDHYNTAR				Mascot
1864.944	1864.9055	-0.0385	-21	326	341	DAEGQDVLLFIDNIFR				Mascot
1864.944	1864.9055	-0.0385	-21	326	341	DAEGQDVLLFIDNIFR	117	100		Mascot
1868.9059	1868.9335	0.0276	15	271	286	EGNDLYREMIESGVIK			Oxidation (M)[9]	Mascot
2013.054	2012.9679	-0.0861	-43	512	529	YVELKESITSFQGVLDGK				Mascot
2061.0498	2060.9963	-0.0535	-26	419	437	QISELGIYPAVDPLDSTSR				Mascot
2172.1548	2172.0808	-0.074	-34	198	217	EAPAFVEQATEQQILVTG IK				Mascot
2186.1453	2186.1045	-0.0408	-19	356	376	IPSAVGYQPTLATDLGGL QER				Mascot
2186.1453	2186.1045	-0.0408	-19	356	376	IPSAVGYQPTLATDLGGL QER	167	100		Mascot
2235.2019	2235.1631	-0.0388	-17	104	123	FDEGLPPILTALEVLDHSI R				Mascot
2433.1531	2433.083	-0.0701	-29	530	550	YDDLPEQSFYMIGGIEEVI AK			Oxidation (M)[11]	Mascot
2591.3135	2591.271	-0.0425	-16	79	103	ITDEFTGAGSIGQVCQVI GAVVDVR			Carbamidomethyl (C)[15]	Mascot
2591.3135	2591.271	-0.0425	-16	79	103	ITDEFTGAGSIGQVCQVI GAVVDVR	153	100	Carbamidomethyl (C)[15]	Mascot
2688.376	2688.3176	-0.0584	-22	464	487	NLQDIIAILGMDELSEDDK LTVAR			Oxidation (M)[11]	Mascot
3714.8862	3714.8528	-0.0334	-9	383	418	GSITSVQAIYVPADDLTD				Mascot

3842.9812	3842.9358	-0.0454	-12	382	418	PAPATTFAHLDATTVLSR KGSITSVQAIYVPADDLT DPAPATTFAHLDATTVLS R					Mascot

10

hypothetical protein Csa_1G599450 [Cucumis sativus]	gi|700211255	64746.1	5.86		29	1,120	100	955	100

Protein Group

PREDICTED: ruBisCO large subunit-binding protein subunit beta, chloroplastic [Cucumis sativus]	gi|449452644	64746.1	5.8600 001335 144

Peptide Information

Calc. Mass	Obsrv. Mass	± da	± ppm	Start Seq.	End Seq.	Sequence	Ion Score	C. I. %	Modification	Rank	Result Type
953.4686	953.436	-0.0326	-34	533	540	VLSSDNYR					Mascot
959.5156	959.4839	-0.0317	-33	235	243	GVVTLEEGR					Mascot
1087.6106	1087.5814	-0.0292	-27	234	243	KGVVTLEEGR					Mascot
1140.4691	1140.4241	-0.045	-39	493	501	ETFENDEEK					Mascot
1143.481	1143.4425	-0.0385	-34	273	281	MAVEYENCK			Carbamidomethyl (C)[8]		Mascot
1159.4758	1159.418	-0.0578	-50	273	281	MAVEYENCK			Carbamidomethyl (C)[8], Oxidation (M)[1]		Mascot
1182.7094	1182.6649	-0.0445	-38	86	97	LADLVGVTLGPK					Mascot
1229.6848	1229.6051	-0.0797	-65	456	466	LRVEDALNATK					Mascot
1245.5715	1245.507	-0.0645	-52	567	577	CCLEHAASVAK			Carbamidomethyl (C)[1,2]		Mascot
1280.7686	1280.724	-0.0446	-35	169	181	VVAAGANPVLITR					Mascot
1280.7686	1280.724	-0.0446	-35	169	181	VVAAGANPVLITR	76	100			Mascot
1284.7158	1284.672	-0.0438	-34	294	304	DLINILEDAIR					Mascot
1358.6699	1358.6171	-0.0528	-39	66	77	ELHFNQDGSAIK					Mascot
1358.6699	1358.6171	-0.0528	-39	66	77	ELHFNQDGSAIK	66	99.994			Mascot
1450.7061	1450.6481	-0.058	-40	413	424	NLIEVAEQDYEK					Mascot
1450.7061	1450.6481	-0.058	-40	413	424	NLIEVAEQDYEK	75	100			Mascot
1486.7649	1486.7024	-0.0625	-42	66	78	ELHFNQDGSAIKK					Mascot
1505.7159	1505.6472	-0.0687	-46	260	272	GYISPYFVTDSEK					Mascot
1505.7159	1505.6472	-0.0687	-46	260	272	GYISPYFVTDSEK	96	100			Mascot
1540.775	1540.7169	-0.0581	-38	578	590	TFLMSDCVVVEIK			Carbamidomethyl (C)[7]		Mascot
1541.7694	1541.6893	-0.0801	-52	122	135	EVELEDPVENIGAK					Mascot
1556.7699	1556.7042	-0.0657	-42	578	590	TFLMSDCVVVEIK			Carbamidomethyl (C)[7], Oxidation (M)[4]		Mascot

Calc. Mass	Obsrv. Mass	± da	± ppm	Start	End	Sequence	Ion	C. I. %	Modification	Rank	Result Type
1643.8785	1643.8136	-0.0649	-39	467	483	AAVEEGIVVGGGCTLLR					Mascot
1666.7806	1666.7211	-0.0595	-36	488	501	VDAIKETFENDEEK					Mascot

Calc. Mass	Obsrv. Mass	± da	± ppm	Start	End	Sequence	Ion	C. I. %	Modification	Rank	Result Type
1700.9	1700.8472	-0.0528	-31	467	483	AAVEEGIVVGGGCTLLR			Carbamidomethyl (C)[13]		Mascot
1700.9	1700.8472	-0.0528	-31	467	483	AAVEEGIVVGGGCTLLR	126	100	Carbamidomethyl (C)[13]		Mascot
1822.8705	1822.7875	-0.083	-46	493	508	ETFENDEEKVGADIVK					Mascot
1834.991	1834.9377	-0.0533	-29	348	364	SQYLDDIAILTGGTVIR	163	100			Mascot
1864.9247	1864.8589	-0.0658	-35	390	407	DTTTIVGDGSTQEAVSKR					Mascot
1890.8691	1890.825	-0.0441	-23	244	259	SADNFLYVVEGMQFDR					Mascot
1890.8691	1890.825	-0.0441	-23	244	259	SADNFLYVVEGMQFDR	58	99.964			Mascot
1900.0386	1899.9551	-0.0835	-44	434	452	LSGGVAVIQVGAQTETEL K					Mascot
1906.864	1906.7986	-0.0654	-34	244	259	SADNFLYVVEGMQFDR			Oxidation (M)[12]		Mascot
1906.864	1906.7986	-0.0654	-34	244	259	SADNFLYVVEGMQFDR	70	99.998	Oxidation (M)[12]		Mascot
1963.0859	1963.0248	-0.0611	-31	347	364	KSQYLDDIAILTGGTVIR					Mascot
1963.0859	1963.0248	-0.0611	-31	347	364	KSQYLDDIAILTGGTVIR	87	100			Mascot
2157.1763	2157.0811	-0.0952	-44	434	454	LSGGVAVIQVGAQTETEL KEK					Mascot
2430.1826	2430.1772	-0.0054	-2	1	24	MASTFTAMSSIGTLAAPG SRVMDK					Mascot
2430.2722	2430.1772	-0.095	-39	144	168	TNDLAGDGTTTSVVLAQ GLIAEGVK	198	100			Mascot
2448.1387	2448.0569	-0.0818	-33	541	563	YGYNAATGNYEDLMAAG IIDPTK					Mascot
2464.1338	2464.0354	-0.0984	-40	541	563	YGYNAATGNYEDLMAAG IIDPTK			Oxidation (M)[14]		Mascot
3219.4417	3219.3423	-0.0994	-31	200	230	EVEDSELADVAAVSAGN NHEVGNMIAEAMSK			Oxidation (M)[24,29]		Mascot

11

hypothetical protein Csa_5G149330 [Cucumis sativus]	gi|700194836	73384	5.1	30	786	100	626	100

Peptide Information

Calc. Mass	Obsrv. Mass	± da	± ppm	Start	End	Sequence	Ion	C. I. %	Modification	Rank	Result Type

				Seq.	Seq.		Score			
943.4957	943.4883	-0.0074	-8	269	275	IMEYFIK				Mascot
959.4907	959.4833	-0.0074	-8	269	275	IMEYFIK			Oxidation (M)[2]	Mascot
965.4938	965.4792	-0.0146	-15	162	170	ETAEAFLGK				Mascot
989.4897	989.4858	-0.0039	-4	542	549	LSQEEIDR				Mascot
998.5013	998.4965	-0.0048	-5	89	97	NQAAVNPER				Mascot
1025.5487	1025.5452	-0.0035	-3	305	313	ALSSQHQVR				Mascot
1047.5834	1047.5679	-0.0155	-15	130	138	DGKPYIQVK				Mascot
1169.6637	1169.6622	-0.0015	-1	193	204	DAGIIAGLNVAR				Mascot
1183.647	1183.6432	-0.0038	-3	491	501	FDLTGIPPAPR				Mascot
1183.647	1183.6432	-0.0038	-3	491	501	FDLTGIPPAPR	74	99.999		Mascot
1224.6293	1224.6139	-0.0154	-13	160	170	MKETAEAFLGK				Mascot
1226.5688	1226.5751	0.0063	5	384	393	DYFEGKEPNK				Mascot
1240.6243	1240.6134	-0.0109	-9	160	170	MKETAEAFLGK			Oxidation (M)[1]	Mascot
1296.6219	1296.6161	-0.0058	-4	334	343	FEELNNDLFR				Mascot
1296.6219	1296.6161	-0.0058	-4	334	343	FEELNNDLFR	85	100		Mascot
1324.5903	1324.5901	-0.0002	0	553	563	EAEEFAEEDKK				Mascot

1379.6147	1379.6064	-0.0083	-6	572	582	NSLETYTYNMK			Oxidation (M)[10]	Mascot
1467.7843	1467.7874	0.0031	2	378	389	VQQLLKDYFEGK				Mascot
1467.7843	1467.7874	0.0031	2	378	389	VQQLLKDYFEGK				Mascot
1500.8018	1500.7975	-0.0043	-3	361	374	NQIDEIVLVGGSTR				Mascot
1500.8018	1500.7975	-0.0043	-3	361	374	NQIDEIVLVGGSTR	120	100		Mascot
1508.738	1508.7322	-0.0058	-4	69	81	ITPSWVAFTDSER				Mascot
1508.738	1508.7322	-0.0058	-4	69	81	ITPSWVAFTDSER	36	94.003		Mascot
1523.7601	1523.7483	-0.0118	-8	332	343	ARFEELNNDLFR				Mascot
1523.7601	1523.7483	-0.0118	-8	332	343	ARFEELNNDLFR	20	0		Mascot
1536.7513	1536.7419	-0.0094	-6	55	68	NGHVEIIANDQGNR				Mascot
1564.8291	1564.7528	-0.0763	-49	146	159	VFSPEEISAMILTK				Mascot
1580.8241	1580.7814	-0.0427	-27	146	159	VFSPEEISAMILTK			Oxidation (M)[10]	Mascot
1595.8905	1595.7754	-0.1151	-72	487	501	NLGKFDLTGIPPAPR				Mascot
1659.8953	1659.8585	-0.0368	-22	205	220	IINEPTAAAIAYGLDK				Mascot
1665.8231	1665.8118	-0.0113	-7	174	188	DAVVTVPAYFNDAQR				Mascot
1665.8231	1665.8118	-0.0113	-7	174	188	DAVVTVPAYFNDAQR	44	98.972		Mascot
1680.9027	1680.8186	-0.0841	-50	82	97	LIGEAAKNQAAVNPER				Mascot

Calc. Mass	Obsrv. Mass	± da	± ppm	Start Seq.	End Seq.	Sequence	Ion Score	C. I. %	Modification	Rank	Result Type
1787.9901	1787.9526	-0.0375	-21	205	221	IINEPTAAAIAYGLDKK					Mascot
1861.9478	1861.937	-0.0108	-6	631	646	EVEAVCNPIITAVYQR			Carbamidomethyl (C)[6]		Mascot
1907.0022	1906.9874	-0.0148	-8	172	188	IKDAVVTVPAYFNDAQR					Mascot
1907.0022	1906.9874	-0.0148	-8	172	188	IKDAVVTVPAYFNDAQR	95	100			Mascot
2054.9917	2054.98	-0.0117	-6	314	331	VEIESLFDGTDFSEPLTR					Mascot
2054.9917	2054.98	-0.0117	-6	314	331	VEIESLFDGTDFSEPLTR	144	100			Mascot
2103.1267	2103.1199	-0.0068	-3	629	646	LKEVEAVCNPIITAVYQR			Carbamidomethyl (C)[8]		Mascot
2103.1267	2103.1199	-0.0068	-3	629	646	LKEVEAVCNPIITAVYQR	9	0	Carbamidomethyl (C)[8]		Mascot
2427.0471	2427.0098	-0.0373	-15	609	628	DALEWLDDNQSAEKEDYEEK					Mascot
2691.2898	2691.2937	0.0039	1	457	479	SQVFTTYQDQQTTVSIQVFEGER					Mascot

12

hypothetical protein Csa_5G160160 [Cucumis sativus] gi|700195036 90329.2 5.49 30 376 100 244 100

Peptide Information

Calc. Mass	Obsrv. Mass	± da	± ppm	Start Seq.	End Seq.	Sequence	Ion Score	C. I. %	Modification	Rank	Result Type
824.4413	824.4523	0.011	13	261	267	LGANFFR					Mascot
877.4521	877.4442	-0.0079	-9	722	728	MLEPIMK			Oxidation (M)[1]		Mascot
885.4828	885.4999	0.0171	19	151	157	ILYYTGR					Mascot
904.5138	904.5259	0.0121	13	658	665	EYIPGVVK					Mascot
978.4891	978.5006	0.0115	12	125	132	SVPLEDYR					Mascot
1086.5361	1086.5156	-0.0205	-19	477	485	LLEMHANSR			Oxidation (M)[4]		Mascot
1134.5902	1134.6261	0.0359	32	124	132	RSVPLEDYR					Mascot
1255.6464	1255.6274	-0.019	-15	133	144	NIGIMAHIDAGK			Oxidation (M)[5]		Mascot
1291.6675	1291.6538	-0.0137	-11	37	48	EGKTIMAAESVR					Mascot
1310.8042	1310.8031	-0.0011	-1	490	503	VALAGDIVALAGLK					Mascot
1416.723	1416.7332	0.0102	7	593	605	VEANVGAPQVNYR					Mascot
1416.723	1416.7332	0.0102	7	593	605	VEANVGAPQVNYR	45	99.208			Mascot
1433.6809	1433.6921	0.0112	8	552	563	LAQEDPSFHFSR					Mascot
1433.6809	1433.6921	0.0112	8	552	563	LAQEDPSFHFSR	9	0			Mascot
1455.7261	1455.6698	-0.0563	-39	457	470	LSAGSYVMNSNKGK					Mascot
1463.7238	1463.7335	0.0097	7	621	634	QSGGQGQFADITVR					Mascot
1463.7238	1463.7335	0.0097	7	621	634	QSGGQGQFADITVR	65	99.993			Mascot
1468.7278	1468.7352	0.0074	5	411	423	GTDPENPELIVER					Mascot

1482.7112	1482.7159	0.0047	3	424	437	VASDDEPFSGLAFK				Mascot
1491.6461	1491.7291	0.083	56	635	647	FEPMEAGSGYEFK				Mascot
1507.641	1507.6467	0.0057	4	635	647	FEPMEAGSGYEFK			Oxidation (M)[4]	Mascot
1568.8142	1568.7601	-0.0541	-34	438	451	IMSDPFVGSLTFVR				Mascot
1573.8446	1573.8484	0.0038	2	749	763	RGQINSFGDKPGGLK				Mascot
1573.8446	1573.8484	0.0038	2	749	763	RGQINSFGDKPGGLK				Mascot
1584.8091	1584.8	-0.0091	-6	438	451	IMSDPFVGSLTFVR			Oxidation (M)[2]	Mascot
1584.8091	1584.8	-0.0091	-6	438	451	IMSDPFVGSLTFVR	8	0	Oxidation (M)[2]	Mascot
1591.8187	1591.821	0.0023	1	620	634	KQSGGQGQFADITVR				Mascot

1669.8441	1669.9102	0.0661	40	369	385	GAISACFVPVLCGSAFK				Mascot
1708.9017	1708.8622	-0.0395	-23	798	812	FDVVPQHIQNELAAK				Mascot
1820.929	1820.9363	0.0073	4	590	605	EFKVEANVGAPQVNYR				Mascot
2068.071	2068.0823	0.0113	5	198	215	INIIDTPGHVDFTLEVER				Mascot
2068.071	2068.0823	0.0113	5	198	215	INIIDTPGHVDFTLEVER	17	0		Mascot
2092.0203	2091.9946	-0.0257	-12	666	685	GLEECMSNGVLAGFPVVDVR				Mascot
2167.1467	2167.1304	-0.0163	-8	764	782	VVDALVPLAEMFQYVSTLR			Oxidation (M)[11]	Mascot
2178.1038	2178.1162	0.0124	6	729	748	VEVVTPEEHLGDVIGDLNSR				Mascot
2178.1038	2178.1162	0.0124	6	729	748	VEVVTPEEHLGDVIGDLNSR	76	100		Mascot
2229.0347	2229.0515	0.0168	8	316	333	FQYEDIPEDLVDLAQDYR				Mascot
2447.2566	2447.2744	0.0178	7	686	708	AVLVDGTYHDVDSSVLAFQLAAR				Mascot
2447.2566	2447.2744	0.0178	7	686	708	AVLVDGTYHDVDSSVLAFQLAAR	25	18.165		Mascot
2668.3135	2668.3228	0.0093	3	564	586	DEEINQTVIEGMGELHLEIIVDR			Oxidation (M)[12]	Mascot

13										
	hypothetical protein Csa_3G126240 [Cucumis sativus]	gi|700201476	80569.6	6	27	197	100	76	100	

Protein Group

chloroplast transketolase [Cucumis sativus]	gi|351735634	80569.6	6
transketolase [Cucumis sativus]	gi|525507124	80569.6	6

Peptide Information

Calc. Mass	Obsrv. Mass	± da	± ppm	Start Seq.	End Seq.	Sequence	Ion Score	C. I. %	Modification	Rank	Result Type
805.4202	805.4151	-0.0051	-6	714	722	FGASAPAGK					Mascot
904.3869	904.3965	0.0096	11	469	475	MYGDFQK			Oxidation (M)[1]		Mascot
996.4686	996.4788	0.0102	10	129	135	NPYWFNR					Mascot
996.4686	996.4788	0.0102	10	129	135	NPYWFNR	15	0			Mascot
1005.5615	1005.5681	0.0066	7	91	99	FLAIDAVEK					Mascot
1046.5775	1046.5778	0.0003	0	591	599	RPSIMALSR			Oxidation (M)[5]		Mascot
1049.515	1049.5117	-0.0033	-3	399	407	YPEEAAELK					Mascot
1138.5123	1138.5247	0.0124	11	296	305	NGNTGYDEIR					Mascot
1171.6317	1171.6422	0.0105	9	677	687	ESVLPEAVTAR					Mascot
1171.6317	1171.6422	0.0105	9	677	687	ESVLPEAVTAR	7	0			Mascot
1177.6099	1177.6104	0.0005	0	398	407	KYPEEAAELK					Mascot
1193.5433	1193.558	0.0147	12	425	435	YTPESPGDATR					Mascot
1193.5433	1193.558	0.0147	12	425	435	YTPESPGDATR	33	89.557			Mascot
1221.6475	1221.6342	-0.0133	-11	323	334	VTTTIGFGSPNK					Mascot
1359.705	1359.6959	-0.0091	-7	436	447	NLSQQCLNALAK			Carbamidomethyl (C)[6]		Mascot
1361.6808	1361.6808	0	0	335	348	ANSYSVHGSALGAK					Mascot
1432.7795	1432.7849	0.0054	4	726	739	EFGLTVEAVVAAAR					Mascot
1476.8057	1476.7838	-0.0219	-15	602	615	LPHLPGTSIEGVEK					Mascot
1534.8435	1534.7654	-0.0781	-51	2	16	ASTSSLTLSQALLSR					Mascot
1685.7919	1685.792	0.0001	0	377	391	HTPLGADFEAEWNAK					Mascot
1689.9534	1689.7874	-0.166	-98	85	99	SINTIRFLAIDAVEK					Mascot
1732.9592	1732.9436	-0.0156	-9	600	615	QKLPHLPGTSIEGVEK					Mascot

1732.9592	1732.9436	-0.0156	-9	600	615	QKLPHLPGTSIEGVEK	20	0			Mascot
1796.9429	1796.9114	-0.0315	-18	408	424	SIITGELPAGWEDALPK					Mascot
1837.0219	1837.027	0.0051	3	723	739	IYKEFGLTVEAVVAAAR					Mascot
2107.1104	2107.0928	-0.0176	-8	448	468	VLPGFLGGSADLASSNMTLLK			Oxidation (M)[17]		Mascot
2142.0291	2142.0383	0.0092	4	355	372	SNLGWPYEPFHVPEDVQK					Mascot

2167.1387	2167.0024	-0.1363	-63	1	20	MASTSSLTLSQALLSREISR			Oxidation (M)[1]		Mascot
2299.0845	2299.0981	0.0136	6	563	583	AMPNILMFRPADGNETAGAYK			Oxidation (M)[2,7]		Mascot
3023.448	3023.4927	0.0447	15	257	283	LIALYDDNHISIDGDTEIAFTESVDTR					Mascot
3471.7754	3471.845	0.0696	20	176	210	TPGHPENFETPGVEVTTGPLGQGIANAVGLALAEK					Mascot

14

hypothetical protein Csa_3G126240 [Cucumis sativus]	gi|700201476	80569.6	6	32	453	100	285	100

Protein Group

chloroplast transketolase [Cucumis sativus]	gi|351735634	80569.6	6
transketolase [Cucumis sativus]	gi|525507124	80569.6	6

Peptide Information

Calc. Mass	Obsrv. Mass	± da	± ppm	Start Seq.	End Seq.	Sequence	Ion Score	C. I. %	Modification	Rank	Result Type
888.392	888.4042	0.0122	14	469	475	MYGDFQK					Mascot
904.3869	904.393	0.0061	7	469	475	MYGDFQK			Oxidation (M)[1]		Mascot
994.5541	994.4639	-0.0902	-91	48	56	LTGSAHRPR					Mascot
996.4686	996.4788	0.0102	10	129	135	NPYWFNR					Mascot
996.4686	996.4788	0.0102	10	129	135	NPYWFNR	56	99.934			Mascot
1005.5615	1005.5667	0.0052	5	91	99	FLAIDAVEK					Mascot
1046.5775	1046.5789	0.0014	1	591	599	RPSIMALSR			Oxidation (M)[5]		Mascot
1049.515	1049.524	0.009	9	399	407	YPEEAAELK					Mascot
1138.5123	1138.5271	0.0148	13	296	305	NGNTGYDEIR					Mascot
1158.6776	1158.6503	-0.0273	-24	590	599	KRPSIMALSR					Mascot
1171.6317	1171.6437	0.012	10	677	687	ESVLPEAVTAR					Mascot
1174.6725	1174.6653	-0.0072	-6	590	599	KRPSIMALSR			Oxidation (M)[6]		Mascot
1177.6099	1177.6205	0.0106	9	398	407	KYPEEAAELK					Mascot
1193.5433	1193.5576	0.0143	12	425	435	YTPESPGDATR					Mascot
1193.5433	1193.5576	0.0143	12	425	435	YTPESPGDATR	46	99.362			Mascot
1221.6475	1221.6403	-0.0072	-6	323	334	VTTTIGFGSPNK					Mascot
1338.6689	1338.6716	0.0027	2	688	700	VSIEAGSTFGWGK					Mascot
1359.705	1359.7084	0.0034	3	436	447	NLSQQCLNALAK			Carbamidomethyl (C)[6]		Mascot

1361.6808	1361.6869	0.0061	4	335	348	ANSYSVHGSALGAK				Mascot
1432.7795	1432.7889	0.0094	7	726	739	EFGLTVEAVVAAAR				Mascot
1432.7795	1432.7889	0.0094	7	726	739	EFGLTVEAVVAAAR	27	53.034		Mascot
1470.7893	1470.7758	-0.0135	-9	284	295	FEGLGWHVIWVK				Mascot

1476.8057	1476.7897	-0.016	-11	602	615	LPHLPGTSIEGVEK				Mascot
1534.8435	1534.7814	-0.0621	-40	2	16	ASTSSLTLSQALLSR				Mascot
1685.7919	1685.7974	0.0055	3	377	391	HTPLGADFEAEWNAK				Mascot
1685.7919	1685.7974	0.0055	3	377	391	HTPLGADFEAEWNAK	95	100		Mascot
1689.9534	1689.7983	-0.1551	-92	85	99	SINTIRFLAIDAVEK				Mascot
1732.9592	1732.9419	-0.0173	-10	600	615	QKLPHLPGTSIEGVEK				Mascot
1796.9429	1796.9279	-0.015	-8	408	424	SIITGELPAGWEDALPK				Mascot
1837.0219	1837.0209	-0.001	-1	723	739	IYKEFGLTVEAVVAAAR				Mascot
2107.1104	2107.1028	-0.0076	-4	448	468	VLPGFLGGSADLASSNM TLLK			Oxidation (M)[17]	Mascot
2134.9968	2135.0251	0.0283	13	659	676	VVSFVSWELFDDQSDAY K				Mascot
2142.0291	2142.0459	0.0168	8	355	372	SNLGWPYEPFHVPEDVQ K				Mascot
2142.0291	2142.0459	0.0168	8	355	372	SNLGWPYEPFHVPEDVQ K	61	99.982		Mascot
2167.1387	2167.0054	-0.1333	-62	1	20	MASTSSLTLSQALLSREI SR			Oxidation (M)[1]	Mascot
2299.0845	2299.0908	0.0063	3	563	583	AMPNILMFRPADGNETA GAYK			Oxidation (M)[2,7]	Mascot
2714.2195	2714.2446	0.0251	9	100	124	ANSGHPGLPMGCAPMG HILYDEVMK			Carbamidomethyl (C)[12], Oxidation (M)[10,15]	Mascot
2730.2144	2730.22	0.0056	2	100	124	ANSGHPGLPMGCAPMG HILYDEVMK			Carbamidomethyl (C)[12], Oxidation (M)[10,15,24]	Mascot
3023.448	3023.5063	0.0583	19	257	283	LIALYDDNHISIDGDTEIAF TESVDTR				Mascot
3471.7754	3471.8469	0.0715	21	176	210	TPGHPENFETPGVEVTT GPLGQGIANAVGLALAEK				Mascot

hypothetical protein Csa_3G836450 [Cucumis sativus]	gi|700204528	48849.9	5.02		22	586	100	459	100

Protein Group

PREDICTED: peptidyl-prolyl cis-trans isomerase CYP38, chloroplastic [Cucumis sativus]	gi|449446650	48849.9	5.0199999809 2651

Peptide Information

Calc. Mass	Obsrv. Mass	± da	± ppm	Start Seq.	End Seq.	Sequence	Ion Score	C. I. %	Modification	Rank	Result Type
856.4886	856.5408	0.0522	61	207	214	DAVAPKQK					Mascot
870.4791	870.5587	0.0796	91	205	212	NRDAVAPK					Mascot
877.4811	877.4879	0.0068	8	249	256	AAVDMKVK			Oxidation (M)[5]		Mascot
933.504	933.5109	0.0069	7	119	126	YALPIDNK					Mascot
967.5207	967.5202	-0.0005	-1	181	189	EHGVEVLGK					Mascot
1095.6157	1095.621	0.0053	5	180	189	KEHGVEVLGK					Mascot
1144.6572	1144.6666	0.0094	8	169	179	NLIISGLAESK					Mascot
1273.7263	1273.6639	-0.0624	-49	119	129	YALPIDNKAIR					Mascot
1295.5837	1295.6027	0.019	15	291	300	HFYDGMEIQR					Mascot
1295.5837	1295.6027	0.019	15	291	300	HFYDGMEIQR	54	99.915			Mascot
1311.5787	1311.5917	0.013	10	291	300	HFYDGMEIQR			Oxidation (M)[6]		Mascot
1311.5787	1311.5917	0.013	10	291	300	HFYDGMEIQR	49	99.734	Oxidation (M)[6]		Mascot
1344.708	1344.7214	0.0134	10	328	339	TVPLEIMVEGEK					Mascot
1360.7029	1360.7097	0.0068	5	328	339	TVPLEIMVEGEK			Oxidation (M)[7]		Mascot
1426.697	1426.7179	0.0209	15	359	371	LPFNAFGTMAMAR					Mascot
1427.6849	1427.7252	0.0403	28	30	42	LWNGACSNPVGPR			Carbamidomethyl (C)[6]		Mascot
1442.6919	1442.707	0.0151	10	359	371	LPFNAFGTMAMAR			Oxidation (M)[9]		Mascot
1442.6919	1442.707	0.0151	10	359	371	LPFNAFGTMAMAR			Oxidation (M)[9]		Mascot
1458.6869	1458.6981	0.0112	8	359	371	LPFNAFGTMAMAR			Oxidation (M)[9,11]		Mascot
1458.6869	1458.6981	0.0112	8	359	371	LPFNAFGTMAMAR			Oxidation (M)[9,11]		Mascot
1614.8585	1614.8674	0.0089	6	130	143	EVQKPLEDISESLK					Mascot
1614.8585	1614.8674	0.0089	6	130	143	EVQKPLEDISESLK	66	99.994			Mascot
1631.7871	1631.8109	0.0238	15	389	403	ESELTPSNSNILDGR					Mascot
1631.7871	1631.8109	0.0238	15	389	403	ESELTPSNSNILDGR	92	100			Mascot

1729.8684	1729.8837	0.0153	9	340	354	APFYGETLEELGLYK					Mascot
1729.8684	1729.8837	0.0153	9	340	354	APFYGETLEELGLYK	121	100			Mascot

1755.9124	1755.9352	0.0228	13	190	204	LEVGLDELQQIVEDR					Mascot
1755.9124	1755.9352	0.0228	13	190	204	LEVGLDELQQIVEDR	128	100			Mascot
1890.9122	1890.9188	0.0066	3	1	17	MAAIVSCKFCSSLTTSK			Carbamidomethyl (C)[7,10]		Mascot
2013.9553	2013.9854	0.0301	15	372	388	EEFDNNSASSQVFWLLK					Mascot
2138.033	2138.0557	0.0227	11	404	421	YTVFGYITENEDFLADLK					Mascot
2463.2437	2463.281	0.0373	15	422	444	VGDVIESMQVVSGLDNLVNPSYK					Mascot
2479.2385	2479.2585	0.02	8	422	444	VGDVIESMQVVSGLDNLVNPSYK			Oxidation (M)[8]		Mascot
2563.1836	2563.2151	0.0315	12	301	325	ADGFVVQTGDPEGPAEGFIDPSTEK					Mascot
3066.3813	3066.4451	0.0638	21	215	240	ELLNYVGSVEEDMVDGFPYEVPEEYK			Oxidation (M)[13]		Mascot

16

Peroxidase [Cucumis sativus]	gi|700198939	34275.7	4.94	11	589	100	538	100
**Protein Group**								
peroxidase 2-like precursor [Cucumis sativus]	gi|525506991	34275.7	4.94000005722046					
peroxidase [Cucumis sativus]	gi|167533	34275.7	4.94000005722046					
unnamed protein product [Cucumis sativus]	gi|257656815	34275.7	4.94000005722046					
unnamed protein product [Cucumis sativus]	gi|219908445	34275.7	4.94000005722046					

Peptide Information

Calc. Mass	Obsrv. Mass	± da	± ppm	Start Seq.	End Seq.	Sequence	Ion Score	C. I. %	Modification	Rank	Result Type
830.4479	830.4485	0.0006	1	150	157	TANLQGAR					Mascot
834.4316	834.4315	-0.0001	0	308	315	TGTTGEIR					Mascot
863.3538	863.3566	0.0028	3	199	205	CMFFSGR			Oxidation (M)[2]		Mascot

920.3753	920.3669	-0.0084	-9	199	205	CMFFSGR			Carbamidomethyl (C)[1], Oxidation (M)[2]	Mascot
920.3753	920.3669	-0.0084	-9	199	205	CMFFSGR	31	83.049	Carbamidomethyl (C)[1], Oxidation (M)[2]	Mascot
1710.8558	1710.847	-0.0088	-5	250	263	NYYTNLQNNQGLLR				Mascot
1710.8558	1710.847	-0.0088	-5	250	263	NYYTNLQNNQGLLR	116	100		Mascot
1732.9886	1732.849	-0.1396	-81	7	22	VIVSLVLCLMMAVSVR				Mascot
1758.9135	1758.9076	-0.0059	-3	129	145	DAVTLASGQGWTVQLGR				Mascot
1804.9592	1804.9545	-0.0047	-3	160	175	LPSPFESLSNIQGIFR				Mascot
2076.0874	2076.0776	-0.0098	-5	158	175	DRLPSPFESLSNIQGIFR				Mascot
2076.0874	2076.0776	-0.0098	-5	158	175	DRLPSPFESLSNIQGIFR	28	71.673		Mascot
2172.0681	2172.0586	-0.0095	-4	176	196	DVGLNDNTDLVALSGAH TFGR				Mascot
2172.0681	2172.0586	-0.0095	-4	176	196	DVGLNDNTDLVALSGAH TFGR	197	100		Mascot
2288.1375	2288.1333	-0.0042	-2	107	128	TAVENVCPGVVSCADILA LGSR			Carbamidomethyl (C)[7,13]	Mascot
2288.1375	2288.1333	-0.0042	-2	107	128	TAVENVCPGVVSCADILA LGSR	165	100	Carbamidomethyl (C)[7,13]	Mascot
3793.8225	3793.8674	0.0449	12	264	300	SDQVLFSTPGASTIATVN SLASSESAFADAFAQSMI			Oxidation (M)[35]	Mascot

R

17	PREDICTED: chromoplast-specific carotenoid-associated protein, chromoplast [Cucumis sativus]	gi|449434000	35215.5	5.05		15	682	100	598	100
	**Protein Group**									
	RecName: Full=Chromoplast-specific carotenoid-associated protein, chromoplastic; Flags: Precursor	gi|62899808	35215.5	5.0500 001907 3486						
	chromoplast-specific carotenoid-associated protein CHRC [Cucumis sativus]	gi|4138857	35215.5	5.0500 001907 3486						
	chromoplast-specific carotenoid-associated protein, CHRC [Cucumis sativus]	gi|1523992	35215.5	5.0500 001907						

hypothetical protein Csa_6G512870 [Cucumis sativus]	gi|700193868	35215.5	3486<br>5.0500<br>001907<br>3486

Peptide Information

Calc. Mass	Obsrv. Mass	± da	± ppm	Start Seq.	End Seq.	Sequence	Ion Score	C. I. %	Modification	Rank	Result Type
824.4261	824.4261	0	0	279	285	FSISNTR					Mascot
970.5568	970.5516	-0.0052	-5	270	278	TISSQPPIK					Mascot
1091.6096	1091.5978	-0.0118	-11	304	314	GDGGSVFVLLK					Mascot
1091.6096	1091.5978	-0.0118	-11	304	314	GDGGSVFVLLK	50	99.751			Mascot
1240.5692	1240.5807	0.0115	9	74	85	YGDDSSVAVAEK					Mascot
1243.5955	1243.5898	-0.0057	-5	102	112	ALVDSFYGTDR					Mascot
1243.5955	1243.5898	-0.0057	-5	102	112	ALVDSFYGTDR	80	100			Mascot
1371.6903	1371.6813	-0.009	-7	101	112	KALVDSFYGTDR					Mascot
1371.6903	1371.6813	-0.009	-7	101	112	KALVDSFYGTDR	18	0			Mascot
1472.8207	1472.791	-0.0297	-20	122	134	AEIVELITQLESK					Mascot
1472.8207	1472.791	-0.0297	-20	122	134	AEIVELITQLESK	75	100			Mascot
1590.7898	1590.775	-0.0148	-9	86	98	EEEKPLEPSEIYK					Mascot
1636.8905	1636.8373	-0.0532	-33	135	150	NPTPAPTEALTLLNGK					Mascot
1762.7766	1762.7513	-0.0253	-14	59	73	AVLNDDEWGEDKDEK					Mascot
1852.9327	1852.9211	-0.0116	-6	286	300	VESWLLTTYLDEDLR					Mascot
1852.9327	1852.9211	-0.0116	-6	286	300	VESWLLTTYLDEDLR	128	100			Mascot
2338.1926	2338.1506	-0.042	-18	248	269	IDFTPFNGIISSLQDTASN					Mascot

**Project 1\Sample project201(**	2338.1506	**58**	Mascot

						VAK					
2812.3411	2812.3293	-0.0118	-4	74	98	YGDDSSVAVAEKEEEKP LEPSEIYK					Mascot
2812.3411	2812.3293	-0.0118	-4	74	98	YGDDSSVAVAEKEEEKP LEPSEIYK	151	100			Mascot
2931.4988	2931.4851	-0.0137	-5	221	247	FEEGVIGTPQLTDSIVIPD NVDFLGQK					Mascot
2931.4988	2931.4851	-0.0137	-5	221	247	FEEGVIGTPQLTDSIVIPD NVDFLGQK	97	100			Mascot
3722.8608	3722.9116	0.0508	14	174	208	VEEISQTIDSENLTVQNS					Mascot

VQFSGPLATTSITTNAK

18

hypothetical protein Csa_3G710190 [Cucumis sativus]	gi|700203507	31959.5	5.53	9	221	100	185	100

Protein Group

PREDICTED: ACT domain-containing protein ACR11 [Cucumis sativus]	gi|449439743	31959.5	5.5300 002098 0835

Peptide Information

Calc. Mass	Obsrv. Mass	± da	± ppm	Start Seq.	End Seq.	Sequence	Ion Score	C. I. %	Modification	Rank	Result Type
808.41	808.4091	-0.0009	-1	257	262	FHVSYR					Mascot
856.5251	856.5237	-0.0014	-2	121	128	NLGLNVVK					Mascot
1037.4899	1037.4879	-0.002	-2	284	292	RPTTEEASF					Mascot
1037.4899	1037.4879	-0.002	-2	284	292	RPTTEEASF	39	97.038			Mascot
1259.7028	1259.6793	-0.0235	-19	109	120	LGALLDTMNALK					Mascot
1269.6686	1269.6626	-0.006	-5	153	163	VDDPELLEAIR					Mascot
1269.6686	1269.6626	-0.006	-5	153	163	VDDPELLEAIR	38	96.707			Mascot
1275.6978	1275.6821	-0.0157	-12	109	120	LGALLDTMNALK			Oxidation (M)[8]		Mascot
1397.7635	1397.7528	-0.0107	-8	152	163	KVDDPELLEAIR					Mascot
1397.7635	1397.7528	-0.0107	-8	152	163	KVDDPELLEAIR	110	100			Mascot
1891.1488	1891.1414	-0.0074	-4	263	279	GKPLIKPLQQVISNSLR					Mascot
1891.1488	1891.1414	-0.0074	-4	263	279	GKPLIKPLQQVISNSLR					Mascot
2101.1904	2101.1851	-0.0053	-3	214	232	SLLYVETADRPGLLVDLVK					Mascot
2101.1904	2101.1851	-0.0053	-3	214	232	SLLYVETADRPGLLVDLVK					Mascot
2235.0637	2235.0425	-0.0212	-9	194	213	QQVDVDIATHINVQDDGPDR					Mascot

19

hypothetical protein Csa_1G013770 [Cucumis sativus]	gi|700208634	28457.9	5.07	11	484	100	426	100

Protein Group

PREDICTED: glycine-rich RNA-binding protein blt801	gi|778656094	28457.9	5.0700

[Cucumis sativus] 001716 6138

Peptide Information

Calc. Mass	Obsrv. Mass	± da	± ppm	Start Seq.	End Seq.	Sequence	Ion Score	C. I. %	Modification	Rank	Result Type
851.4006	851.3969	-0.0037	-4	116	122	VNYANDR					Mascot
936.4785	936.4688	-0.0097	-10	66	73	YGEVIEAR					Mascot
936.4785	936.4688	-0.0097	-10	66	73	YGEVIEAR	59	99.974			Mascot
1044.5797	1044.5616	-0.0181	-17	74	82	VIVDRETGR					Mascot
1155.4437	1155.4725	0.0288	25	270	279	DEDDSNDFAK					Mascot
1361.5717	1361.5546	-0.0171	-13	258	269	ASGEDHFEENAR					Mascot
1361.5717	1361.5546	-0.0171	-13	258	269	ASGEDHFEENAR	65	99.994			Mascot
1512.7693	1512.7351	-0.0342	-23	61	73	EAFTKYGEVIEAR					Mascot
1658.6963	1658.6666	-0.0297	-18	243	257	SEGEQFGSNETNTMK					Mascot
1658.6963	1658.6666	-0.0297	-18	243	257	SEGEQFGSNETNTMK	5	0			Mascot
1674.6912	1674.6547	-0.0365	-22	243	257	SEGEQFGSNETNTMK			Oxidation (M)[14]		Mascot
1674.6912	1674.6547	-0.0365	-22	243	257	SEGEQFGSNETNTMK	64	99.993	Oxidation (M)[14]		Mascot
1727.86	1727.8401	-0.0199	-12	45	60	VFVGGISFSTDDQSLR					Mascot
1727.86	1727.8401	-0.0199	-12	45	60	VFVGGISFSTDDQSLR	164	100			Mascot
2497.9976	2497.9585	-0.0391	-16	258	279	ASGEDHFEENARDEDDS NDFAK					Mascot
2497.9976	2497.9585	-0.0391	-16	258	279	ASGEDHFEENARDEDDS NDFAK	37	95.823			Mascot
2943.3755	2943.3655	-0.01	-3	85	112	GFGFVTYTSSEEASSAIQ ALDGQDLHGR					Mascot
2943.3755	2943.3655	-0.01	-3	85	112	GFGFVTYTSSEEASSAIQ ALDGQDLHGR	37	96.362			Mascot
3957.686	3957.7173	0.0313	8	202	242	GSVGGFGGNDAGYNAA SNFATGNTFGSESNAGF GSSDYFAK					Mascot

20

hypothetical protein Csa_2G151040 [Cucumis sativus]	gi|700206394	35161.4	4.79	22	534	100	381	100

Protein Group

PREDICTED: uncharacterized protein LOC101217348	gi|449442405	35161.4	4.7899

[Cucumis sativus] 999618

5303

Peptide Information

Calc. Mass	Obsrv. Mass	± da	± ppm	Start Seq.	End Seq.	Sequence	Ion Score	C. I. %	Modification	Rank	Result Type
813.508	813.4987	-0.0093	-11	164	170	LTLPLEK					Mascot
877.4778	877.4775	-0.0003	0	128	134	LVFDDIR					Mascot
935.4833	935.4739	-0.0094	-10	209	216	NDFELGLK					Mascot
1073.5262	1073.5055	-0.0207	-19	36	44	DFIHDIGEK					Mascot
1235.6155	1235.5973	-0.0182	-15	232	242	ADLSEFTPIDK					Mascot
1235.6155	1235.5973	-0.0182	-15	232	242	ADLSEFTPIDK	58	99.962			Mascot
1300.6896	1300.6708	-0.0188	-14	34	44	VKDFIHDIGEK					Mascot
1310.6199	1310.5938	-0.0261	-20	258	268	DFGSALWDMIR					Mascot
1326.6147	1326.6022	-0.0125	-9	258	268	DFGSALWDMIR			Oxidation (M)[9]		Mascot
1328.7573	1328.7382	-0.0191	-14	152	163	VDLIVDVPVFGR					Mascot
1419.7478	1419.7271	-0.0207	-15	205	216	LENKNDFELGLK					Mascot
1419.7478	1419.7271	-0.0207	-15	205	216	LENKNDFELGLK	53	99.869			Mascot
1443.705	1443.6846	-0.0204	-14	278	290	GNIHVDTPFGQMK					Mascot
1459.7	1459.6672	-0.0328	-22	278	290	GNIHVDTPFGQMK			Oxidation (M)[12]		Mascot
1459.7	1459.6672	-0.0328	-22	278	290	GNIHVDTPFGQMK	76	100	Oxidation (M)[12]		Mascot
1533.8312	1533.8125	-0.0187	-12	192	204	FSFEETVAILHLK					Mascot
1569.9364	1569.9191	-0.0173	-11	150	163	IKVDLIVDVPVFGR					Mascot
1719.9429	1719.9231	-0.0198	-12	243	257	NGISYVDLPITFRPK					Mascot
1745.9221	1745.8865	-0.0356	-20	135	149	NTYHDIKPGSIIPYK					Mascot
1745.9221	1745.8865	-0.0356	-20	135	149	NTYHDIKPGSIIPYK	81	100			Mascot
1763.8269	1763.8165	-0.0104	-6	217	231	DLDCEVWLSDVSIGR			Carbamidomethyl (C)[4]		Mascot
1763.8269	1763.8165	-0.0104	-6	217	231	DLDCEVWLSDVSIGR	7	0	Carbamidomethyl (C)[4]		Mascot
1813.9583	1813.9158	-0.0425	-23	171	186	TGEIPIPYKPDVDIEK					Mascot
1813.9583	1813.9158	-0.0425	-23	171	186	TGEIPIPYKPDVDIEK	106	100			Mascot

2142.1553	2142.1213	-0.034	-16	103	123	LIAGLIPDAGTIHAHGEET VK					Mascot
2164.0857	2164.1196	0.0339	16	271	290	GTGYTIKGNIHVDTPFGQ MK					Mascot
2270.2502	2270.2141	-0.0361	-16	102	123	KLIAGLIPDAGTIHAHGEE					Mascot

Calc. Mass	Obsrv. Mass	± da	± ppm	Start Seq.	End Seq.	Sequence	Ion Score	C. I. %	Modification	Rank	Result Type
						TVK					
2336.2134	2336.1943	-0.0191	-8	81	101	NPNPVPIPLVDIDYLIESD GR					Mascot
2464.3083	2464.2886	-0.0197	-8	81	102	NPNPVPIPLVDIDYLIESD GRK					Mascot
2738.4612	2738.4387	-0.0225	-8	45	70	IEETIGFGKPTADVSGIHI PSISLEK					Mascot

21

hypothetical protein Csa_6G008680 [Cucumis sativus]	gi|700190533	51768.1	5.58		19	707	100	614	100

Protein Group

PREDICTED: ribulose bisphosphate carboxylase/oxygenase activase, chloroplastic [Cucumis sativus]	gi|449441384	51768.1	5.5799999237 0605

Peptide Information

Calc. Mass	Obsrv. Mass	± da	± ppm	Start Seq.	End Seq.	Sequence	Ion Score	C. I. %	Modification	Rank	Result Type
895.4156	895.3989	-0.0167	-19	352	358	VYDDEVR					Mascot
940.4675	940.4474	-0.0201	-21	303	309	FYWAPTR					Mascot
940.4675	940.4474	-0.0201	-21	303	309	FYWAPTR	46	99.413			Mascot
1008.5295	1008.5089	-0.0206	-20	313	321	IGVCSGIFR			Carbamidomethyl (C)[4]		Mascot
1008.5295	1008.5089	-0.0206	-20	313	321	IGVCSGIFR	26	44.235	Carbamidomethyl (C)[4]		Mascot
1104.6234	1104.5455	-0.0779	-71	148	156	NFMKLPNIK					Mascot
1129.5525	1129.5233	-0.0292	-26	378	387	EGPPTFEQPK					Mascot
1152.714	1152.6809	-0.0331	-29	157	167	VPLILGIWGGK					Mascot
1228.6031	1228.573	-0.0301	-24	172	181	SFQCELVFAK			Carbamidomethyl (C)[4]		Mascot
1228.6031	1228.573	-0.0301	-24	172	181	SFQCELVFAK	63	99.988	Carbamidomethyl (C)[4]		Mascot
1257.6686	1257.6365	-0.0321	-26	322	332	SDNIPKEDIVK					Mascot
1566.7404	1566.7745	0.0341	22	222	236	MCALFINDLDAGAGR					Mascot
1623.7618	1623.7255	-0.0363	-22	222	236	MCALFINDLDAGAGR			Carbamidomethyl (C)[2]		Mascot
1623.7618	1623.7255	-0.0363	-22	222	236	MCALFINDLDAGAGR	115	100	Carbamidomethyl (C)[2]		Mascot
1639.7567	1639.714	-0.0427	-26	222	236	MCALFINDLDAGAGR			Carbamidomethyl (C)[2], Oxidation (M)[1]		Mascot
1639.7567	1639.714	-0.0427	-26	222	236	MCALFINDLDAGAGR	61	99.983	Carbamidomethyl (C)[2], Oxidation (M)[1]		Mascot
1679.7872	1679.749	-0.0382	-23	73	87	GLAFDVSDDQQDITR					Mascot
1679.7872	1679.749	-0.0382	-23	73	87	GLAFDVSDDQQDITR	100	100			Mascot

1864.9036	1864.9233	0.0197	11	73	89	GLAFDVSDDQQDITRGK					Mascot
1882.9698	1882.9327	-0.0371	-20	333	349	LVDTFPGQSIDFFGALR					Mascot
1882.9698	1882.9327	-0.0371	-20	333	349	LVDTFPGQSIDFFGALR	142	100			Mascot
1906.9579	1906.905	-0.0529	-28	393	408	LLEYGNMLVQEQENVK					Mascot
1922.9529	1922.8799	-0.073	-38	393	408	LLEYGNMLVQEQENVK			Oxidation (M)[7]		Mascot
2063.0591	2063.0146	-0.0445	-22	393	409	LLEYGNMLVQEQENVKR					Mascot

2079.054	2079.0002	-0.0538	-26	393	409	LLEYGNMLVQEQENVKR			Oxidation (M)[7]		Mascot
2089.1692	2089.1274	-0.0418	-20	278	296	VPIIVTGNDFSTLYAPLIR					Mascot
2089.1692	2089.1274	-0.0418	-20	278	296	VPIIVTGNDFSTLYAPLIR	125	100			Mascot
2304.0669	2303.917	-0.1499	-65	182	204	MGINPIMMSAGELESGN AGEPAK					Mascot
2320.0618	2319.9756	-0.0862	-37	182	204	MGINPIMMSAGELESGN AGEPAK			Oxidation (M)[1]		Mascot
2336.0566	2335.9861	-0.0705	-30	182	204	MGINPIMMSAGELESGN AGEPAK			Oxidation (M)[1,7]		Mascot
2352.0515	2351.981	-0.0705	-30	182	204	MGINPIMMSAGELESGN AGEPAK			Oxidation (M)[1,7,8]		Mascot
2369.0544	2368.9678	-0.0866	-37	121	140	QYNFDNNVDGFYIAPAF MDK					Mascot
2385.0493	2384.9692	-0.0801	-34	121	140	QYNFDNNVDGFYIAPAF MDK			Oxidation (M)[18]		Mascot
2784.2893	2784.2424	-0.0469	-17	439	464	AAQQVSLPIPEGCTDPNA DNFDPTAR			Carbamidomethyl (C)[13]		Mascot
3239.5679	3239.522	-0.0459	-14	90	120	GMVDTLFQAPSGAGTHD PVLSSYEYLSAGLR					Mascot
3255.5627	3255.5149	-0.0478	-15	90	120	GMVDTLFQAPSGAGTHD PVLSSYEYLSAGLR			Oxidation (M)[2]		Mascot

22

PREDICTED: 30S ribosomal protein S1, chloroplastic [Cucumis sativus]	gi|449459770	45304.5	5.34	21	851	100	731	100	

Peptide Information

Calc. Mass	Obsrv. Mass	± da	± ppm	Start	End	Sequence	Ion	C. I. %	Modification	Rank	Result Type

				Seq.	Seq.		Score			
942.5366	942.5162	-0.0204	-22	363	371	IAQAEALAR				Mascot
970.5139	970.4961	-0.0178	-18	315	322	VMILSHDR				Mascot
986.5087	986.4674	-0.0413	-42	315	322	VMILSHDR			Oxidation (M)[2]	Mascot
1055.6208	1055.6024	-0.0184	-17	200	210	GGVVAVVEGLR				Mascot
1055.6208	1055.6024	-0.0184	-17	200	210	GGVVAVVEGLR	59	99.967		Mascot
1082.4935	1082.4761	-0.0174	-16	352	360	AEEMAQTFR				Mascot
1098.4884	1098.4653	-0.0231	-21	352	360	AEEMAQTFR			Oxidation (M)[4]	Mascot
1128.5718	1128.558	-0.0138	-12	334	343	LEPTPGDMIR				Mascot
1144.5668	1144.5376	-0.0292	-26	334	343	LEPTPGDMIR			Oxidation (M)[8]	Mascot
1171.5266	1171.5063	-0.0203	-17	61	69	EVFEEAYER				Mascot
1171.5266	1171.5063	-0.0203	-17	61	69	EVFEEAYER	29	69.133		Mascot
1174.5264	1174.5096	-0.0168	-14	92	101	YDFDSELGTK				Mascot
1210.6467	1210.6102	-0.0365	-30	211	221	GFVPFSQISTK				Mascot
1225.5994	1225.5635	-0.0359	-29	2	12	ASMAQQFTGLR			Oxidation (M)[3]	Mascot
1237.5696	1237.549	-0.0206	-17	236	245	FVEVDEEQSR				Mascot
1237.5696	1237.549	-0.0206	-17	236	245	FVEVDEEQSR	45	99.286		Mascot
1256.6667	1256.6422	-0.0245	-19	333	343	KLEPTPGDMIR				Mascot
1272.6617	1272.6329	-0.0288	-23	333	343	KLEPTPGDMIR			Oxidation (M)[9]	Mascot
1280.627	1280.605	-0.022	-17	170	179	SIQYDLAWER				Mascot
1280.627	1280.605	-0.022	-17	170	179	SIQYDLAWER	65	99.993		Mascot
1310.6852	1310.661	-0.0242	-18	138	149	HVEEAGVFPGLR				Mascot
1310.6852	1310.661	-0.0242	-18	138	149	HVEEAGVFPGLR	78	100		Mascot
1372.6348	1372.6204	-0.0144	-10	1	12	MASMAQQFTGLR			Oxidation (M)[1,4]	Mascot
1412.7056	1412.6808	-0.0248	-18	59	69	LKEVFEEAYER				Mascot
1412.7056	1412.6808	-0.0248	-18	59	69	LKEVFEEAYER	95	100		Mascot
1551.8643	1551.8333	-0.031	-20	136	149	IKHVEEAGVFPGLR				Mascot

1556.853	1556.806	-0.047	-30	300	314	ISDIATVLQPGDSLK				Mascot
1587.7948	1587.7474	-0.0474	-30	122	135	SSAYLPLQEACIHR				Mascot
1644.8163	1644.7843	-0.032	-19	122	135	SSAYLPLQEACIHR			Carbamidomethyl (C)[11]	Mascot
1644.8163	1644.7843	-0.032	-19	122	135	SSAYLPLQEACIHR	93	100	Carbamidomethyl (C)[11]	Mascot
1895.9169	1895.8527	-0.0642	-34	104	121	GTVFCTDNNGALVDITAK			Carbamidomethyl (C)[5]	Mascot
2188.092	2188.0374	-0.0546	-25	72	91	NAPVEGISFTLEDFHAAL EK				Mascot

2188.092	2188.0374	-0.0546	-25	72	91	NAPVEGISFTLEDFHAAL EK	145	100			Mascot
2278.145	2278.1016	-0.0434	-19	150	169	EEFVIIGENESDDSLILSL R					Mascot
2278.145	2278.1016	-0.0434	-19	150	169	EEFVIIGENESDDSLILSL R	122	100			Mascot

23

hypothetical protein Csa_6G008680 [Cucumis sativus]	gi|700190533	51768.1	5.58	22	761	100	645	100

Protein Group

PREDICTED: ribulose bisphosphate carboxylase/oxygenase activase, chloroplastic [Cucumis sativus]	gi|449441384	51768.1	5.5799 999237 0605

Peptide Information

Calc. Mass	Obsrv. Mass	± da	± ppm	Start Seq.	End Seq.	Sequence	Ion Score	C. I. %	Modification	Rank	Result Type
895.4156	895.3979	-0.0177	-20	352	358	VYDDEVR					Mascot
940.4675	940.4453	-0.0222	-24	303	309	FYWAPTR					Mascot
940.4675	940.4453	-0.0222	-24	303	309	FYWAPTR	26	42.908			Mascot
1008.5295	1008.5054	-0.0241	-24	313	321	IGVCSGIFR			Carbamidomethyl (C)[4]		Mascot
1008.5295	1008.5054	-0.0241	-24	313	321	IGVCSGIFR	32	84.276	Carbamidomethyl (C)[4]		Mascot
1129.5525	1129.5183	-0.0342	-30	378	387	EGPPTFEQPK					Mascot
1152.714	1152.6183	-0.0957	-83	157	167	VPLILGIWGGK					Mascot
1228.6031	1228.5701	-0.033	-27	172	181	SFQCELVFAK			Carbamidomethyl (C)[4]		Mascot
1228.6031	1228.5701	-0.033	-27	172	181	SFQCELVFAK	66	99.995	Carbamidomethyl (C)[4]		Mascot
1257.6686	1257.6338	-0.0348	-28	322	332	SDNIPKEDIVK					Mascot
1328.6456	1328.6256	-0.02	-15	300	309	MEKFYWAPTR					Mascot
1340.6382	1340.6047	-0.0335	-25	303	312	FYWAPTREDR					Mascot
1566.7404	1566.7496	0.0092	6	222	236	MCALFINDLDAGAGR					Mascot
1605.8418	1605.7063	-0.1355	-84	313	327	IGVCSGIFRSDNIPK					Mascot
1623.7618	1623.7167	-0.0451	-28	222	236	MCALFINDLDAGAGR			Carbamidomethyl (C)[2]		Mascot
1623.7618	1623.7167	-0.0451	-28	222	236	MCALFINDLDAGAGR	118	100	Carbamidomethyl (C)[2]		Mascot
1639.7567	1639.7001	-0.0566	-35	222	236	MCALFINDLDAGAGR			Carbamidomethyl (C)[2], Oxidation (M)[1]		Mascot
1679.7872	1679.7413	-0.0459	-27	73	87	GLAFDVSDDQQDITR					Mascot
1679.7872	1679.7413	-0.0459	-27	73	87	GLAFDVSDDQQDITR	102	100			Mascot

1864.9036	1864.9194	0.0158	8	73	89	GLAFDVSDDQQDITRGK				Mascot
1882.9698	1882.9253	-0.0445	-24	333	349	LVDTFPGQSIDFFGALR				Mascot
1882.9698	1882.9253	-0.0445	-24	333	349	LVDTFPGQSIDFFGALR	138	100		Mascot
1906.9579	1906.8899	-0.068	-36	393	408	LLEYGNMLVQEQENVK				Mascot
1922.9529	1922.8811	-0.0718	-37	393	408	LLEYGNMLVQEQENVK			Oxidation (M)[7]	Mascot

2063.0591	2063.0088	-0.0503	-24	393	409	LLEYGNMLVQEQENVKR				Mascot
2063.0591	2063.0088	-0.0503	-24	393	409	LLEYGNMLVQEQENVKR	28	66.996		Mascot
2079.054	2078.9939	-0.0601	-29	393	409	LLEYGNMLVQEQENVKR			Oxidation (M)[7]	Mascot
2089.1692	2089.1199	-0.0493	-24	278	296	VPIIVTGNDFSTLYAPLIR				Mascot
2089.1692	2089.1199	-0.0493	-24	278	296	VPIIVTGNDFSTLYAPLIR	136	100		Mascot
2304.0669	2303.9775	-0.0894	-39	182	204	MGINPIMMSAGELESGN AGEPAK				Mascot
2320.0618	2319.9578	-0.104	-45	182	204	MGINPIMMSAGELESGN AGEPAK			Oxidation (M)[1]	Mascot
2334.1824	2334.0547	-0.1277	-55	410	431	VQLADKYLSEAALGDAN EDAIK				Mascot
2352.0515	2352.001	-0.0505	-21	182	204	MGINPIMMSAGELESGN AGEPAK			Oxidation (M)[1,7,8]	Mascot
2369.0544	2368.9727	-0.0817	-34	121	140	QYNFDNNVDGFYIAPAF MDK				Mascot
2385.0493	2384.9661	-0.0832	-35	121	140	QYNFDNNVDGFYIAPAF MDK			Oxidation (M)[18]	Mascot
2784.2893	2784.2322	-0.0571	-21	439	464	AAQQVSLPIPEGCTDPNA DNFDPTAR			Carbamidomethyl (C)[13]	Mascot
3255.5627	3255.5032	-0.0595	-18	90	120	GMVDTLFQAPSGAGTHD PVLSSYEYLSAGLR			Oxidation (M)[2]	Mascot

24											
	PREDICTED: actin-7 [Cucumis sativus]	gi|449459238	41682.9	5.31	18	454	100	352	100
	**Protein Group**								
	PREDICTED: actin-7 [Cucumis melo]	gi|659121974	41682.9	5.3099999427 7954					

PREDICTED: actin-7 [Cucumis melo]	gi|659080849	41682.9	5.3099999427 7954
PREDICTED: actin-7 [Cucumis sativus]	gi|778717846	41682.9	5.3099999427 7954
actin [Cucumis sativus]	gi|700207016	41682.9	5.3099999427 7954
hypothetical protein Csa_6G484600 [Cucumis sativus]	gi|700193178	41682.9	5.3099999427 7954

Peptide Information

Calc. Mass	Obsrv. Mass	± da	± ppm	Start Seq.	End Seq.	Sequence	Ion Score	C. I. %	Modification	Rank	Result Type
890.4764	890.5403	0.0639	72	209	215	EIVRDMK					Mascot
976.4483	976.4277	-0.0206	-21	21	30	AGFAGDDAPR					Mascot
1144.5602	1144.5256	-0.0346	-30	42	52	HTGVMVGMGQK					Mascot
1160.5552	1160.525	-0.0302	-26	42	52	HTGVMVGMGQK			Oxidation (M)[5]		Mascot
1176.5354	1176.5128	-0.0226	-19	199	208	GYMFTTTAER					Mascot
1176.5354	1176.5128	-0.0226	-19	199	208	GYMFTTTAER	46	99.446			Mascot
1182.5273	1182.5116	-0.0157	-13	53	63	DAYVGDEAQSK					Mascot
1192.5304	1192.5005	-0.0299	-25	199	208	GYMFTTTAER			Oxidation (M)[3]		Mascot
1192.5304	1192.5005	-0.0299	-25	199	208	GYMFTTTAER			Oxidation (M)[3]		Mascot
1198.7056	1198.6801	-0.0255	-21	31	41	AVFPSIVGRPR					Mascot
1198.7056	1198.6801	-0.0255	-21	31	41	AVFPSIVGRPR					Mascot
1445.6655	1445.6359	-0.0296	-20	362	374	GEYDESGPSIVHR					Mascot
1515.7491	1515.7161	-0.033	-22	87	97	IWHHTFYNELR					Mascot
1515.7491	1515.7161	-0.033	-22	87	97	IWHHTFYNELR	71	99.998			Mascot
1531.8148	1531.7096	-0.1052	-69	180	193	LDLAGRDLTDALMK					Mascot
1547.8098	1547.7078	-0.102	-66	180	193	LDLAGRDLTDALMK			Oxidation (M)[13]		Mascot

1547.8098	1547.7078	-0.102	-66	180	193	LDLAGRDLTDALMK			Oxidation (M)[13]		Mascot
1774.897	1774.8544	-0.0426	-24	241	256	NYELPDGQVITIGAER					Mascot
1774.897	1774.8544	-0.0426	-24	241	256	NYELPDGQVITIGAER	143	100			Mascot

1788.8949	1788.8665	-0.0284	-16	194	208	ILTERGYMFTTTAER					Mascot
1788.8949	1788.8665	-0.0284	-16	194	208	ILTERGYMFTTTAER					Mascot
1855.9324	1855.8711	-0.0613	-33	218	233	LAYVALDYEQELETAK					Mascot
1932.8796	1932.8229	-0.0567	-29	71	86	YPIEHGIVSNWDDMEK					Mascot
1948.8746	1948.8154	-0.0592	-30	71	86	YPIEHGIVSNWDDMEK			Oxidation (M)[14]		Mascot
1954.0645	1954.0081	-0.0564	-29	98	115	VAPEEHPVLLTEAPLNPK					Mascot
1954.0645	1954.0081	-0.0564	-29	98	115	VAPEEHPVLLTEAPLNPK	92	100			Mascot
1975.91	1975.9998	0.0898	45	2	20	ADAEDIQPLVCDNGTGM VK					Mascot
2199.0752	2199.0149	-0.0603	-27	294	314	DLYGNIVLSGGSTMFPGI ADR			Oxidation (M)[14]		Mascot
2327.1702	2327.1077	-0.0625	-27	293	314	KDLYGNIVLSGGSTMFPG IADR			Oxidation (M)[15]		Mascot
3151.6423	3151.5981	-0.0442	-14	150	179	TTGIVLDSGDGVSHTVPI YEGYALPHAILR					Mascot

25

hypothetical protein Csa_5G198220 [Cucumis sativus]	gi|700195455	42075.4	5.96	25	602	100	434	100

Protein Group

sedoheptulose-1,7-bisphosphatase [Cucumis sativus]	gi|229597543	42075.4	5.9600 000381 4697
sedoheptulose-1,7-bisphosphatase, chloroplastic-like [Cucumis sativus]	gi|525507240	42075.4	5.9600 000381 4697

Peptide Information

Calc. Mass	Obsrv. Mass	± da	± ppm	Start Seq.	End Seq.	Sequence	Ion Score	C. I. %	Modification	Rank	Result Type
809.4515	809.4296	-0.0219	-27	279	284	EKYTLR					Mascot
841.3661	841.3557	-0.0104	-12	140	145	YSHFCK			Carbamidomethyl (C)[5]		Mascot
853.4414	853.4294	-0.012	-14	355	362	TQVAYGSK					Mascot
861.5192	861.5109	-0.0083	-10	133	139	LLFEALR					Mascot
869.3748	869.3666	-0.0082	-9	332	340	AGGYSSDGR					Mascot
903.5046	903.4917	-0.0129	-14	254	261	LFSPGNLR					Mascot
903.5046	903.4917	-0.0129	-14	254	261	LFSPGNLR	45	99.115			Mascot
908.5451	908.5264	-0.0187	-21	217	224	TTYVLALK					Mascot

912.5189	912.5021	-0.0168	-18	272	278	LINYYVK				Mascot
963.4629	963.4214	-0.0415	-43	245	253	ETTEIGEGK				Mascot
987.5105	987.4944	-0.0161	-16	347	354	VIENLDER				Mascot
987.5105	987.4944	-0.0161	-16	347	354	VIENLDER	36	93.33		Mascot
1122.5646	1122.5355	-0.0291	-26	90	99	LLTCMGEALR			Oxidation (M)[5]	Mascot
1163.5912	1163.5724	-0.0188	-16	90	99	LLTCMGEALR			Carbamidomethyl (C)[4]	Mascot
1163.5912	1163.5724	-0.0188	-16	90	99	LLTCMGEALR	6	0	Carbamidomethyl (C)[4]	Mascot
1184.4857	1184.5093	0.0236	20	1	10	METGIACCAR			Carbamidomethyl (C)[7,8], Oxidation (M)[1]	Mascot
1185.5059	1185.483	-0.0229	-19	262	271	ATFDNPDYDK				Mascot
1188.5532	1188.5321	-0.0211	-18	368	377	FEETLYGSSR				Mascot
1188.5532	1188.5321	-0.0211	-18	368	377	FEETLYGSSR	40	97.545		Mascot
1251.658	1251.627	-0.031	-25	302	313	GIFTNVTSPSTK				Mascot
1394.7638	1394.6627	-0.1011	-72	34	46	SLKASSLFGESLR				Mascot
1478.7057	1478.6755	-0.0302	-20	202	216	GSDQVAAAMGVYGPR	127	100		Mascot

1494.7006	1494.661	-0.0396	-26	202	216	GSDQVAAAMGVYGPR			Oxidation (M)[9]	Mascot
1494.7006	1494.661	-0.0396	-26	202	216	GSDQVAAAMGVYGPR	90	100	Oxidation (M)[9]	Mascot
1574.9193	1574.866	-0.0533	-34	318	331	LLFEVAPLGFLVEK				Mascot
1602.8818	1602.7698	-0.112	-70	86	99	GLIRLLTCMGEALR			Carbamidomethyl (C)[8]	Mascot
1633.8618	1633.7942	-0.0676	-41	285	299	YTGGMVPDVNQIIVK				Mascot
1649.8568	1649.8019	-0.0549	-33	285	299	YTGGMVPDVNQIIVK			Oxidation (M)[5]	Mascot
1649.8568	1649.8019	-0.0549	-33	285	299	YTGGMVPDVNQIIVK	81	100	Oxidation (M)[5]	Mascot
1717.8433	1717.7953	-0.048	-28	225	239	DFPGTHEFLLLDEGK				Mascot
1717.8433	1717.7953	-0.048	-28	225	239	DFPGTHEFLLLDEGK	105	100		Mascot
1739.9183	1739.7874	-0.1309	-75	90	104	LLTCMGEALRTISFK			Carbamidomethyl (C)[4]	Mascot
1755.9132	1755.7645	-0.1487	-85	90	104	LLTCMGEALRTISFK			Carbamidomethyl (C)[4], Oxidation (M)[5]	Mascot
1828.9076	1828.8649	-0.0427	-23	363	377	EEIIRFEETLYGSSR				Mascot
2079.0071	2078.9524	-0.0547	-26	262	278	ATFDNPDYDKLINYYVK				Mascot
2079.0071	2078.9524	-0.0547	-26	262	278	ATFDNPDYDKLINYYVK				Mascot
2717.1853	2717.1184	-0.0669	-25	107	132	TASCGGTACVNSFGDEQ LAVDMLADK			Carbamidomethyl (C)[4,9]	Mascot
2733.1802	2733.1086	-0.0716	-26	107	132	TASCGGTACVNSFGDEQ LAVDMLADK			Carbamidomethyl (C)[4,9], Oxidation (M)[22]	Mascot

26									
Oxygen-evolving enhancer protein 1 [Cucumis sativus]	gi|700193260	34938.8	6.24		17	445	100	346	100
**Protein Group**									
PREDICTED: oxygen-evolving enhancer protein 1, chloroplastic [Cucumis sativus]	gi|449448384	34938.8		6.2399997711816					

Peptide Information

Calc. Mass	Obsrv. Mass	± da	± ppm	Start Seq.	End Seq.	Sequence	Ion Score	C. I. %	Modification	Rank	Result Type
850.4305	850.4222	-0.0083	-10	237	244	GSSFLDPK					Mascot
964.5866	964.5781	-0.0085	-9	208	215	VPFLFTIK					Mascot
1080.5573	1080.5433	-0.014	-13	91	99	LTFDEIQSK					Mascot
1145.5586	1145.5464	-0.0122	-11	152	161	NAPPEFQNTK					Mascot
1235.6227	1235.5857	-0.037	-30	272	283	ENIKNASSSTGK					Mascot
1236.6583	1236.6439	-0.0144	-12	90	99	RLTFDEIQSK					Mascot
1236.6583	1236.6439	-0.0144	-12	90	99	RLTFDEIQSK	9	0			Mascot
1328.6555	1328.645	-0.0105	-8	135	145	FCLEPTSFTVK			Carbamidomethyl (C)[2]		Mascot
1374.6747	1374.6587	-0.016	-12	264	275	GDEEELAKENIK					Mascot
1456.7505	1456.7344	-0.0161	-11	134	145	KFCLEPTSFTVK			Carbamidomethyl (C)[3]		Mascot
1562.7559	1562.7378	-0.0181	-12	247	263	GGSTGYDNAVALPAGGR					Mascot
1562.7559	1562.7378	-0.0181	-12	247	263	GGSTGYDNAVALPAGGR	93	100			Mascot
1760.8813	1760.8611	-0.0202	-11	191	207	DGIDYAAVTVQLPGGER					Mascot
1760.8813	1760.8611	-0.0202	-11	191	207	DGIDYAAVTVQLPGGER	131	100			Mascot
2119.0999	2118.9541	-0.1458	-69	1	21	MAASVQAAAATLMQPSKLASR			Oxidation (M)[1]		Mascot
2142.9761	2142.949	-0.0271	-13	106	126	GTGTANQCPTIDGGVDSFAFK			Carbamidomethyl (C)[8]		Mascot
2142.9761	2142.949	-0.0271	-13	106	126	GTGTANQCPTIDGGVDSFAFK	35	93.695	Carbamidomethyl (C)[8]		Mascot
2254.1125	2254.0854	-0.0271	-12	166	186	LTYTLDEIEGPFEVGADGSIK					Mascot
2284.1609	2284.1311	-0.0298	-13	216	236	QLVASGKPESFSGDFLVPSYR					Mascot
2284.1609	2284.1311	-0.0298	-13	216	236	QLVASGKPESFSGDFLVPSYR	79	100			Mascot
2434.1482	2434.1172	-0.031	-13	247	271	GGSTGYDNAVALPAGGR					Mascot

2604.304	2604.2874	-0.0166	-6	291	315	GDEEELAK SKPETGEVIGVFESIQPS DTDLGAK					Mascot

27

Oxygen-evolving enhancer protein 1 [Cucumis sativus]	gi|700193260	34938.8	6.24		16	600	100	511	100
**Protein Group**									
PREDICTED: oxygen-evolving enhancer protein 1, chloroplastic [Cucumis sativus]	gi|449448384	34938.8		6.2399997711 1816					

Peptide Information

Calc. Mass	Obsrv. Mass	± da	± ppm	Start Seq.	End Seq.	Sequence	Ion Score	C. I. %	Modification	Rank	Result Type
964.5866	964.571	-0.0156	-16	208	215	VPFLFTIK					Mascot
964.5866	964.571	-0.0156	-16	208	215	VPFLFTIK	64	99.991			Mascot
1080.5573	1080.5437	-0.0136	-13	91	99	LTFDEIQSK					Mascot
1145.5586	1145.5375	-0.0211	-18	152	161	NAPPEFQNTK					Mascot
1236.6583	1236.6438	-0.0145	-12	90	99	RLTFDEIQSK					Mascot
1236.6583	1236.6438	-0.0145	-12	90	99	RLTFDEIQSK	11	0			Mascot
1279.6318	1279.5789	-0.0529	-41	322	332	IQGVWYAQLDS					Mascot
1328.6555	1328.6343	-0.0212	-16	135	145	FCLEPTSFTVK			Carbamidomethyl (C)[2]		Mascot
1374.6747	1374.6544	-0.0203	-15	264	275	GDEEELAKENIK					Mascot
1456.7505	1456.7207	-0.0298	-20	134	145	KFCLEPTSFTVK			Carbamidomethyl (C)[3]		Mascot
1562.7559	1562.734	-0.0219	-14	247	263	GGSTGYDNAVALPAGGR					Mascot
1562.7559	1562.734	-0.0219	-14	247	263	GGSTGYDNAVALPAGGR	53	99.874			Mascot
1760.8813	1760.8563	-0.025	-14	191	207	DGIDYAAVTVQLPGGER					Mascot
1760.8813	1760.8563	-0.025	-14	191	207	DGIDYAAVTVQLPGGER	151	100			Mascot
2142.9761	2142.9294	-0.0467	-22	106	126	GTGTANQCPTIDGGVDS FAFK			Carbamidomethyl (C)[8]		Mascot
2142.9761	2142.9294	-0.0467	-22	106	126	GTGTANQCPTIDGGVDS FAFK	45	99.289	Carbamidomethyl (C)[8]		Mascot
2254.1125	2254.0757	-0.0368	-16	166	186	LTYTLDEIEGPFEVGADG SIK					Mascot

Calc. Mass	Obsrv. Mass	± da	± ppm	Start Seq.	End Seq.	Sequence	Ion Score	C. I. %	Modification	Rank	Result Type
2254.1125	2254.0757	-0.0368	-16	166	186	LTYTLDEIEGPFEVGADG SIK	20	0			Mascot
2284.1609	2284.1318	-0.0291	-13	216	236	QLVASGKPESFSGDFLV PSYR					Mascot
2284.1609	2284.1318	-0.0291	-13	216	236	QLVASGKPESFSGDFLV PSYR	167	100			Mascot
2294.1299	2294.0752	-0.0547	-24	187	207	FEEKDGIDYAAVTVQLPG GER					Mascot
2434.1482	2434.1172	-0.031	-13	247	271	GGSTGYDNAVALPAGGR GDEEELAK					Mascot

Calc. Mass	Obsrv. Mass	± da	± ppm	Start Seq.	End Seq.	Sequence	Ion Score	C. I. %	Modification	Rank	Result Type
2604.304	2604.2563	-0.0477	-18	291	315	SKPETGEVIGVFESIQPS DTDLGAK					Mascot

28

Oxygen-evolving enhancer protein 1 [Cucumis sativus] gi|700193260 34938.8 6.24 18 971 100 860 100

Protein Group

PREDICTED: oxygen-evolving enhancer protein 1, chloroplastic [Cucumis sativus] gi|449448384 34938.8 6.2399997711816

Peptide Information

Calc. Mass	Obsrv. Mass	± da	± ppm	Start Seq.	End Seq.	Sequence	Ion Score	C. I. %	Modification	Rank	Result Type
850.4305	850.427	-0.0035	-4	237	244	GSSFLDPK					Mascot
964.5866	964.5657	-0.0209	-22	208	215	VPFLFTIK					Mascot
964.5866	964.5657	-0.0209	-22	208	215	VPFLFTIK	58	99.963			Mascot
1080.5573	1080.5377	-0.0196	-18	91	99	LTFDEIQSK					Mascot
1145.5586	1145.5375	-0.0211	-18	152	161	NAPPEFQNTK					Mascot
1145.5586	1145.5375	-0.0211	-18	152	161	NAPPEFQNTK	50	99.737			Mascot
1235.6227	1235.6096	-0.0131	-11	272	283	ENIKNASSSTGK					Mascot
1236.6583	1236.6378	-0.0205	-17	90	99	RLTFDEIQSK					Mascot
1236.6583	1236.6378	-0.0205	-17	90	99	RLTFDEIQSK	35	92.88			Mascot
1328.6555	1328.627	-0.0285	-21	135	145	FCLEPTSFTVK			Carbamidomethyl (C)[2]		Mascot

1374.6747	1374.6431	-0.0316	-23	264	275	GDEEELAKENIK				Mascot
1456.7505	1456.719	-0.0315	-22	134	145	KFCLEPTSFTVK			Carbamidomethyl (C)[3]	Mascot
1456.7505	1456.719	-0.0315	-22	134	145	KFCLEPTSFTVK	25	21.393	Carbamidomethyl (C)[3]	Mascot
1544.8101	1544.7073	-0.1028	-67	2	17	AASVQAAAATLMQPSK				Mascot
1560.8051	1560.7046	-0.1005	-64	2	17	AASVQAAAATLMQPSK			Oxidation (M)[12]	Mascot
1562.7559	1562.7268	-0.0291	-19	247	263	GGSTGYDNAVALPAGGR				Mascot
1562.7559	1562.7268	-0.0291	-19	247	263	GGSTGYDNAVALPAGGR	166	100		Mascot
1760.8813	1760.8469	-0.0344	-20	191	207	DGIDYAAVTVQLPGGER				Mascot
1760.8813	1760.8469	-0.0344	-20	191	207	DGIDYAAVTVQLPGGER	157	100		Mascot
2142.9761	2142.9214	-0.0547	-26	106	126	GTGTANQCPTIDGGVDS FAFK			Carbamidomethyl (C)[8]	Mascot
2142.9761	2142.9214	-0.0547	-26	106	126	GTGTANQCPTIDGGVDS FAFK	129	100	Carbamidomethyl (C)[8]	Mascot
2254.1125	2254.0601	-0.0524	-23	166	186	LTYTLDEIEGPFEVGADG SIK				Mascot
2254.1125	2254.0601	-0.0524	-23	166	186	LTYTLDEIEGPFEVGADG SIK	97	100		Mascot

2284.1609	2284.124	-0.0369	-16	216	236	QLVASGKPESFSGDFLV PSYR				Mascot
2284.1609	2284.124	-0.0369	-16	216	236	QLVASGKPESFSGDFLV PSYR	144	100		Mascot
2294.1299	2294.0693	-0.0606	-26	187	207	FEEKDGIDYAAVTVQLPG GER				Mascot
2434.1482	2434.0994	-0.0488	-20	247	271	GGSTGYDNAVALPAGGR GDEEELAK				Mascot
2604.304	2604.2439	-0.0601	-23	291	315	SKPETGEVIGVFESIQPS DTDLGAK				Mascot

29

hypothetical protein Csa_4G268040 [Cucumis sativus]	gi|700198862	37807	6.22		17	578	100	491	100
**Protein Group**									
PREDICTED: uncharacterized protein LOC101217229	gi|449463844	37807	6.2199						

[Cucumis sativus] 997901

9165

Peptide Information

Calc. Mass	Obsrv. Mass	± da	± ppm	Start Seq.	End Seq.	Sequence	Ion Score	C. I. %	Modification	Rank	Result Type
800.3971	800.3944	-0.0027	-3	155	160	TMYTIR			Oxidation (M)[2]		Mascot
802.4166	802.4094	-0.0072	-9	220	225	RENDLR					Mascot
881.425	881.4208	-0.0042	-5	236	242	YEEALEK					Mascot
937.499	937.4852	-0.0138	-15	126	134	TGLFTVGDK					Mascot
1030.5164	1030.5042	-0.0122	-12	296	304	TDPDLSNIR					Mascot
1084.611	1084.6017	-0.0093	-9	204	212	EIQLQNALR					Mascot
1084.611	1084.6017	-0.0093	-9	204	212	EIQLQNALR	72	99.998			Mascot
1126.5264	1126.5122	-0.0142	-13	177	186	TDSFGELTEK					Mascot
1204.646	1204.6304	-0.0156	-13	305	314	TLEEFEPLVK					Mascot
1204.646	1204.6304	-0.0156	-13	305	314	TLEEFEPLVK	73	99.999			Mascot
1285.6859	1285.6349	-0.051	-40	294	304	VRTDPDLSNIR					Mascot
1667.7913	1667.7573	-0.034	-20	109	125	DGGTYIDAIAPGGFADK					Mascot
1667.7913	1667.7573	-0.034	-20	109	125	DGGTYIDAIAPGGFADK	109	100			Mascot
1724.849	1724.8094	-0.0396	-23	316	330	FDESFINENAINAIK					Mascot
1724.849	1724.8094	-0.0396	-23	316	330	FDESFINENAINAIK	84	100			Mascot
1880.9138	1880.9214	0.0076	4	107	125	GRDGGTYIDAIAPGGFAD K					Mascot
1880.9501	1880.9214	-0.0287	-15	315	330	RFDESFINENAINAIK	82	100			Mascot
2039.0081	2038.9867	-0.0214	-10	275	293	AGLSALEDALEAGFEDFK R					Mascot
2039.0081	2038.9867	-0.0214	-10	275	293	AGLSALEDALEAGFEDFK R	74	100			Mascot
2138.0918	2138.0703	-0.0215	-10	135	154	VLATSAVFGTEIWPAAEY GR					Mascot
3202.386	3202.363	-0.023	-7	77	103	TDTDSDDPNQQPYEEYE VELEQPYGLK					Mascot
3562.6829	3562.6829	0	0	243	274	FESVLGSKPTPDEASVAS YNVACCYSQLNQLK			Carbamidomethyl (C)[23,24]		Mascot

hypothetical protein Csa_5G524830 [Cucumis sativus]	gi|700196213	46808.8	7.13	18	739	100	649	100

Protein Group

PREDICTED: protease Do-like 1, chloroplastic isoform X1 [Cucumis sativus]	gi|449450105	46808.8	7.1300001144092

Peptide Information

Calc. Mass	Obsrv. Mass	± da	± ppm	Start Seq.	End Seq.	Sequence	Ion Score	C. I. %	Modification	Rank	Result Type
815.4985	815.4949	-0.0036	-4	415	421	VTVEVLR					Mascot
855.541	855.5117	-0.0293	-34	369	376	AGLLPTKR					Mascot
996.6564	996.6498	-0.0066	-7	332	340	VTRPILGIK					Mascot
996.6564	996.6498	-0.0066	-7	332	340	VTRPILGIK					Mascot
1024.5059	1024.4945	-0.0114	-11	396	404	VTNGSDLYR					Mascot
1145.6161	1145.6091	-0.007	-6	114	123	LQTDELATVR					Mascot
1214.7103	1214.6774	-0.0329	-27	411	421	VGDKVTVEVLR					Mascot
1273.7111	1273.703	-0.0081	-6	113	123	KLQTDELATVR					Mascot
1273.7111	1273.703	-0.0081	-6	113	123	KLQTDELATVR	21	0			Mascot
1309.6635	1309.6528	-0.0107	-8	183	194	VTLADQTTFDAK					Mascot
1309.6635	1309.6528	-0.0107	-8	183	194	VTLADQTTFDAK	77	100			Mascot
1336.7358	1336.7122	-0.0236	-18	428	439	IPVILEPKPDES					Mascot
1336.7358	1336.7122	-0.0236	-18	428	439	IPVILEPKPDES	74	100			Mascot
1406.6812	1406.7013	0.0201	14	30	41	SISFHNFSNPTR					Mascot
1423.7441	1423.7336	-0.0105	-7	165	176	DGHIVTNYHVIR					Mascot
1423.7441	1423.7336	-0.0105	-7	165	176	DGHIVTNYHVIR	67	99.995			Mascot
1776.0742	1776.0607	-0.0135	-8	216	232	LRPIPVGISADLLVGQK					Mascot
1776.0742	1776.0607	-0.0135	-8	216	232	LRPIPVGISADLLVGQK	110	100			Mascot
1784.0065	1783.9307	-0.0758	-42	2	19	AAAAAFSLLPSSLSRPPK					Mascot
1915.047	1914.9578	-0.0892	-47	1	19	MAAAAAFSLLPSSLSRPPK					Mascot
2016.0648	2016.0322	-0.0326	-16	195	213	VVGFDQDKDVAVLSIDAPK					Mascot
2016.0648	2016.0322	-0.0326	-16	195	213	VVGFDQDKDVAVLSIDAPK	130	100			Mascot
2064.1125	2064.1011	-0.0114	-6	124	141	LFQENTPSVVYITNLAVR					Mascot

2064.1125	2064.1011	-0.0114	-6	124	141	LFQENTPSVVYITNLAVR	158	100			Mascot
2385.2925	2385.281	-0.0115	-5	233	255	VFAIGNPFGLDHTLTTGVISGLR					Mascot
2385.2925	2385.281	-0.0115	-5	233	255	VFAIGNPFGLDHTLTTGVISGLR	13	0			Mascot
2736.4204	2736.3965	-0.0239	-9	341	368	FAPDQSVEQLGVSGVLVLDAPANGPAGK					Mascot

31

hypothetical protein Csa_2G252020 [Cucumis sativus]	gi|700206734	42867.2	6.38		21	455	100	332	100
**Protein Group**									
PREDICTED: fructose-bisphosphate aldolase 1, chloroplastic [Cucumis sativus]	gi|449464838	42867.2		6.3800001144 4092					

Peptide Information

Calc. Mass	Obsrv. Mass	± da	± ppm	Start Seq.	End Seq.	Sequence	Ion Score	C. I. %	Modification	Rank	Result Type
811.5036	811.5006	-0.003	-4	138	145	QGIVPGIK					Mascot
822.4355	822.437	0.0015	2	238	244	TFEVAQK					Mascot
871.4996	871.4413	-0.0583	-67	26	33	QPSVSVVR					Mascot
873.4577	873.4637	0.006	7	202	209	EAAWGLAR					Mascot
947.4979	947.4932	-0.0047	-5	339	346	ALQNTCLK			Carbamidomethyl (C)[6]		Mascot
1027.5895	1027.5885	-0.001	-1	357	365	IAQDALIQR					Mascot
1027.5895	1027.5885	-0.001	-1	357	365	IAQDALIQR	45	99.254			Mascot
1098.5327	1098.5315	-0.0012	-1	172	181	AAAYYQQGAR					Mascot
1098.5327	1098.5315	-0.0012	-1	172	181	AAAYYQQGAR	56	99.946			Mascot
1116.6372	1116.5669	-0.0703	-63	366	376	AKSNSLAQLGK					Mascot
1141.6827	1141.6732	-0.0095	-8	99	109	TLLVTAPELGK					Mascot
1387.7175	1387.7139	-0.0036	-3	82	94	LASIGLENTEANR					Mascot
1387.7175	1387.7139	-0.0036	-3	82	94	LASIGLENTEANR	55	99.938			Mascot
1452.8422	1452.8348	-0.0074	-5	187	201	TVVSIPNGPSALAVK					Mascot
1466.6614	1466.6731	0.0117	8	67	80	GILAMDESNATCGK			Carbamidomethyl (C)[12]		Mascot
1482.6564	1482.6487	-0.0077	-5	67	80	GILAMDESNATCGK			Carbamidomethyl (C)[12], Oxidation (M)[5]		Mascot
1543.8187	1543.808	-0.0107	-7	81	94	RLASIGLENTEANR					Mascot
1620.8843	1620.8652	-0.0191	-12	280	294	ASPQTVAEYTLSLLK					Mascot

1620.8843	1620.8652	-0.0191	-12	280	294	ASPQTVAEYTLSLLK	37	96.159			Mascot	
1864.0062	1863.9852	-0.021	-11	278	294	DKASPQTVAEYTLSLLK					Mascot	
1965.8019	1965.7769	-0.025	-13	377	393	YTGEGESEEANQDMYVK			Oxidation (M)[14]		Mascot	
2363.1653	2363.1538	-0.0115	-5	110	130	YISGAILFEETLYQSTVDG EK					Mascot	
2427.1721	2427.1748	0.0027	1	149	171	GLVPLPGSNNESWCQGL DGLASR			Carbamidomethyl (C)[14]		Mascot	
2427.1721	2427.1748	0.0027	1	149	171	GLVPLPGSNNESWCQGL	139	100	Carbamidomethyl (C)[14]		Mascot	
**Project 1\Sample project201(**	2427.1748										**175**	Mascot
						DGLASR						
2434.0027	2434.1328	0.1301	53	377	397	YTGEGESEEANQDMYVK GYTY					Mascot	
2442.2446	2442.1697	-0.0749	-31	34	56	CHPTAAPSTLIVRAGSYN DELVK					Mascot	
2499.2661	2499.019	-0.2471	-99	34	56	CHPTAAPSTLIVRAGSYN DELVK			Carbamidomethyl (C)[1]		Mascot	
3019.5371	3019.5486	0.0115	4	210	237	YAAVSQDNGLVPIVEPEIL LDGEHGIDR					Mascot	

32

hypothetical protein Csa_5G611610 [Cucumis sativus]	gi|700196953	32524.8	6.08		12	525	100	458	100
**Protein Group**									
PREDICTED: fruit protein pKIWI502 [Cucumis sativus]	gi|449434568	32524.8		6.0799 999237 0605					

Peptide Information

Calc. Mass	Obsrv. Mass	± da	± ppm	Start Seq.	End Seq.	Sequence	Ion Score	C. I. %	Modification	Rank	Result Type
948.5261	948.5255	-0.0006	-1	102	109	AGQYLQLR					Mascot
948.5261	948.5255	-0.0006	-1	102	109	AGQYLQLR	52	99.877			Mascot
1095.5681	1095.5648	-0.0033	-3	196	206	SLIESGFGASK					Mascot
1183.5266	1183.5258	-0.0008	-1	228	237	FDEWESSGVK					Mascot
1183.5266	1183.5258	-0.0008	-1	228	237	FDEWESSGVK	89	100			Mascot

1213.6324	1213.601	-0.0314	-26	208	217	TDVRLYYGAR					Mascot
1260.6617	1260.6299	-0.0318	-25	155	166	GDVVQLSQVMGK					Mascot
1276.6566	1276.676	0.0194	15	155	166	GDVVQLSQVMGK			Oxidation (M)[10]		Mascot
1388.7567	1388.7371	-0.0196	-14	154	166	KGDVVQLSQVMGK					Mascot
1404.7516	1404.728	-0.0236	-17	154	166	KGDVVQLSQVMGK			Oxidation (M)[11]		Mascot
1475.7775	1475.767	-0.0105	-7	140	153	SVEGSIAELLCGLK	92	100	Carbamidomethyl (C)[11]		Mascot
1947.8542	1947.8538	-0.0004	0	222	237	MAYQDRFDEWESSGVK					Mascot
1947.8542	1947.8538	-0.0004	0	222	237	MAYQDRFDEWESSGVK	17	0			Mascot
1963.8491	1963.8368	-0.0123	-6	222	237	MAYQDRFDEWESSGVK			Oxidation (M)[1]		Mascot
1963.8491	1963.8368	-0.0123	-6	222	237	MAYQDRFDEWESSGVK	25	39.288	Oxidation (M)[1]		Mascot
2852.3738	2852.3669	-0.0069	-2	238	264	VVPVLSQPESDWTGDSGYVQAAFSNAK					Mascot
2852.3738	2852.3669	-0.0069	-2	238	264	VVPVLSQPESDWTGDSGYVQAAFSNAK	90	100			Mascot
3078.5571	3078.5767	0.0196	6	167	195	GFDVDQIAPPQDFPSVFIFATGSGISPIR					Mascot
3078.5571	3078.5767	0.0196	6	167	195	GFDVDQIAPPQDFPSVFIFATGSGISPIR	112	100			Mascot
3552.7449	3552.7307	-0.0142	-4	266	299	AFDPLSTGAVICGQKPMTEEVTSILVADGVSSEK			Carbamidomethyl (C)[12], Oxidation (M)[17]		Mascot
3680.8398	3680.8628	0.023	6	265	299	KAFDPLSTGAVICGQKPMTEEVTSILVADGVSSEK			Carbamidomethyl (C)[13], Oxidation (M)[18]		Mascot

33	PREDICTED: 20 kDa chaperonin, chloroplastic [Cucumis sativus]	gi|449452602	26867.3	7.85	17	611	100	496	100

Protein Group

PREDICTED: 20 kDa chaperonin, chloroplastic [Cucumis sativus]	gi|778663199	26867.3	7.8499999046 3257

Peptide Information

Calc. Mass	Obsrv. Mass	± da	± ppm	Start Seq.	End Seq.	Sequence	Ion Score	C. I. %	Modification	Rank	Result Type
828.3886	828.396	0.0074	9	230	236	YAGNEFK					Mascot
846.4567	846.4637	0.007	8	76	82	IKEAEEK					Mascot

952.5098	952.5049	-0.0049	-5	122	130	TGAQVVYSK				Mascot
970.4952	970.5006	0.0054	6	162	169	DLQPLNDR				Mascot
970.4952	970.5006	0.0054	6	162	169	DLQPLNDR	52	99.847		Mascot
1009.4949	1009.4996	0.0047	5	239	247	DGSDYIALR				Mascot
1009.4949	1009.4996	0.0047	5	239	247	DGSDYIALR	60	99.979		Mascot
1146.6365	1146.6331	-0.0034	-3	181	192	TAGGLLLTEGSK				Mascot
1149.6263	1149.6267	0.0004	0	62	71	YTSIKPLGDR				Mascot
1149.6263	1149.6267	0.0004	0	62	71	YTSIKPLGDR	52	99.862		Mascot
1194.6113	1194.6182	0.0069	6	237	247	GKDGSDYIALR				Mascot
1532.7327	1532.7244	-0.0083	-5	148	161	EDDIVGILETDDAK				Mascot
1620.8778	1620.851	-0.0268	-17	215	229	KPLTVAVGNNVMYSK				Mascot
1636.8727	1636.8571	-0.0156	-10	215	229	KPLTVAVGNNVMYSK			Oxidation (M)[12]	Mascot
1776.9789	1776.9633	-0.0156	-9	214	229	RKPLTVAVGNNVMYSK				Mascot
1792.9739	1792.9581	-0.0158	-9	214	229	RKPLTVAVGNNVMYSK			Oxidation (M)[13]	Mascot
1792.9739	1792.9581	-0.0158	-9	214	229	RKPLTVAVGNNVMYSK			Oxidation (M)[13]	Mascot
1887.0698	1887.0472	-0.0226	-12	54	71	AATVVAPKYTSIKPLGDR				Mascot
1905.9706	1905.9618	-0.0088	-5	131	147	YAGTELEFNGSNHLILK				Mascot
2133.1187	2133.103	-0.0157	-7	193	213	EKPSIGTVIAVGPGHLDE EGK				Mascot
2133.1187	2133.103	-0.0157	-7	193	213	EKPSIGTVIAVGPGHLDE EGK	173	100		Mascot
2289.2197	2289.2192	-0.0005	0	193	214	EKPSIGTVIAVGPGHLDE EGKR				Mascot
2484.21	2484.2114	0.0014	1	148	169	EDDIVGILETDDAKDLQPL				Mascot
**Project 1\Sample project201(**	2484.2114								**190**	Mascot
						NDR				
2637.3843	2637.3962	0.0119	5	83	109	TDGGILLPSTAQTRPQGG EVVAVGEGK				Mascot
2637.3843	2637.3962	0.0119	5	83	109	TDGGILLPSTAQTRPQGG EVVAVGEGK	159	100		Mascot

34	hypothetical protein Csa_1G479610 [Cucumis sativus]	gi|700210559	27377.8	5.43	15	662	100	566	100

Protein Group

L-ascorbate peroxidase, cytosolic-like [Cucumis sativus]	gi|525507192	27377.8	5.4299<br>998283<br>3862
ascorbate peroxidase [Cucumis sativus]	gi|479280230	27375.8	5.4400<br>000572<br>2046
ascorbate peroxidase [Cucumis sativus]	gi|226897527	27391.8	5.4400<br>000572<br>2046
ascorbate peroxidase, partial [Cucumis sativus]	gi|37020723	27343.8	5.4299<br>998283<br>3862
cytosolic ascorbate peroxidase [Cucumis sativus]	gi|1669585	27377.8	5.4299<br>998283<br>3862
unnamed protein product [Cucumis sativus]	gi|257696294	27377.8	5.4299<br>998283<br>3862
unnamed protein product [Cucumis sativus]	gi|219925171	27377.8	5.4299<br>998283<br>3862
unnamed protein product [Cucumis sativus]	gi|219791837	27377.8	5.4299<br>998283<br>3862
unnamed protein product [Cucumis sativus]	gi|219910391	27377.8	5.4299<br>998283<br>3862
unnamed protein product [Cucumis sativus]	gi|257643540	27377.8	5.4299<br>998283<br>3862
unnamed protein product [Cucumis sativus]	gi|257632785	27377.8	5.4299<br>998283<br>3862
unnamed protein product [Cucumis sativus]	gi|257352583	27377.8	5.4299<br>998283<br>3862

Peptide Information

Calc. Mass	Obsrv. Mass	± da	± ppm	Start Seq.	End Seq.	Sequence	Ion Score	C. I. %	Modification	Rank	Result Type
917.4695	917.5082	0.0387	42	31	38	NCAPLMLR					Mascot
923.4404	923.4523	0.0119	13	53	61	TGGPFGTMR					Mascot

Calc. Mass	Obsrv. Mass	± da	± ppm	Start Seq.	End Seq.	Sequence	Ion Score	C. I. %	Modification	Rank	Result Type
923.4404	923.4523	0.0119	13	53	61	TGGPFGTMR					Mascot
939.4353	939.4443	0.009	10	53	61	TGGPFGTMR			Oxidation (M)[8]		Mascot
939.4353	939.4443	0.009	10	53	61	TGGPFGTMR			Oxidation (M)[8]		Mascot
974.491	974.5038	0.0128	13	31	38	NCAPLMLR			Carbamidomethyl (C)[2]		Mascot
990.4859	990.4941	0.0082	8	31	38	NCAPLMLR			Carbamidomethyl (C)[2], Oxidation (M)[6]		Mascot
1073.5837	1073.5892	0.0055	5	199	208	EGLLQLASDK					Mascot
1250.6012	1250.6146	0.0134	11	120	130	EDKPEPPPEGR					Mascot
1250.6012	1250.6146	0.0134	11	120	130	EDKPEPPPEGR	70	99.998			Mascot
1277.6096	1277.6193	0.0097	8	39	49	LAWHSAGTFCK			Carbamidomethyl (C)[10]		Mascot
1287.6467	1287.6566	0.0099	8	188	198	SYFTELLTGEK					Mascot
1287.6467	1287.6566	0.0099	8	188	198	SYFTELLTGEK	60	99.976			Mascot
1309.6859	1309.6185	-0.0674	-51	131	142	LPDATKGSDHLR					Mascot
1401.6355	1401.6403	0.0048	3	4	14	CYPVVSEEYQK			Carbamidomethyl (C)[1]		Mascot
1583.9155	1583.9269	0.0114	7	209	222	ALLSDPVFRPLVEK					Mascot
1583.9155	1583.9269	0.0114	7	209	222	ALLSDPVFRPLVEK	79	100			Mascot
1619.8396	1619.8293	-0.0103	-6	25	38	GFIAEKNCAPLMLR			Carbamidomethyl (C)[8]		Mascot
1636.8402	1636.8525	0.0123	8	64	79	SELAHGANNGLDIAVR					Mascot
1808.8855	1808.8916	0.0061	3	172	187	SGFEGPWTTNPLIFDK					Mascot
1808.8855	1808.8916	0.0061	3	172	187	SGFEGPWTTNPLIFDK	95	100			Mascot
1912.0035	1912.0167	0.0132	7	62	79	FKSELAHGANNGLDIAVR					Mascot
2061.8826	2061.8958	0.0132	6	223	240	YAADEDAFFADYAEAHQK					Mascot
2061.8826	2061.8958	0.0132	6	223	240	YAADEDAFFADYAEAHQK	144	100			Mascot
2552.2451	2552.2847	0.0396	16	143	166	DVFYTMGLSDQDIVALSGGHTLGR					Mascot
2568.24	2568.2671	0.0271	11	143	166	DVFYTMGLSDQDIVALSGGHTLGR			Oxidation (M)[6]		Mascot
2568.24	2568.2671	0.0271	11	143	166	DVFYTMGLSDQDIVALSG	118	100	Oxidation (M)[6]		Mascot

GHTLGR

35

hypothetical protein Csa_2G263900 [Cucumis sativus]	gi|700206776	32718.8	7.01	19	657	100	539	100

Protein Group

PREDICTED: triosephosphate isomerase, chloroplastic [Cucumis sativus]	gi|449458564	32718.8	7.0100002288 8184

Peptide Information

Calc. Mass	Obsrv. Mass	± da	± ppm	Start Seq.	End Seq.	Sequence	Ion Score	C. I. %	Modification	Rank	Result Type
954.4832	954.4999	0.0167	17	57	64	FFVGGNWK					Mascot
1048.5634	1048.5226	-0.0408	-39	245	254	KNVSSEVASK					Mascot
1096.5898	1096.6113	0.0215	20	141	149	WVILGHSER					Mascot
1096.5898	1096.6113	0.0215	20	141	149	WVILGHSER	70	99.997			Mascot
1109.562	1109.6052	0.0432	39	1	11	MAAVSTSLASR			Oxidation (M)[1]		Mascot
1285.6246	1285.6433	0.0187	15	190	199	TFDVCFQQLK			Carbamidomethyl (C)[5]		Mascot
1357.6747	1357.6921	0.0174	13	151	162	HVIGEDDQFIGK					Mascot
1357.6747	1357.6921	0.0174	13	151	162	HVIGEDDQFIGK	81	100			Mascot
1361.7059	1361.7128	0.0069	5	110	121	IEISAQNSWVSK					Mascot
1419.7479	1419.7595	0.0116	8	289	302	GPEFGTIVNSVTAK					Mascot
1419.7479	1419.7595	0.0116	8	289	302	GPEFGTIVNSVTAK	45	99.155			Mascot
1420.7319	1420.7584	0.0265	19	275	288	EDIDGFLVGGASLK					Mascot
1435.7428	1435.7616	0.0188	13	122	135	GGAFTGEISVEQLK					Mascot
1435.7428	1435.7616	0.0188	13	122	135	GGAFTGEISVEQLK	105	100			Mascot
1457.6995	1457.7501	0.0506	35	57	69	FFVGGNWKCNGTK					Mascot
1485.7697	1485.7875	0.0178	12	151	163	HVIGEDDQFIGKK					Mascot
1513.7759	1513.8015	0.0256	17	150	162	RHVIGEDDQFIGK					Mascot
1547.8428	1547.7777	-0.0651	-42	289	303	GPEFGTIVNSVTAKK					Mascot
1548.8268	1548.8469	0.0201	13	274	288	KEDIDGFLVGGASLK					Mascot
1548.8268	1548.8469	0.0201	13	274	288	KEDIDGFLVGGASLK	126	100			Mascot
1562.865	1562.8146	-0.0504	-32	2	16	AAVSTSLASRFSPLR					Mascot
1619.85	1619.8754	0.0254	16	226	240	VATPEQAQEVHAAIR					Mascot
1619.85	1619.8754	0.0254	16	226	240	VATPEQAQEVHAAIR	112	100			Mascot
1695.837	1695.8441	0.0071	4	257	273	IIYGGSVNGSNCAELAK					Mascot

2274.1904	2274.2092	0.0188	8	84	103	LEDDVDVVVAPPFVYIEQ VK					Mascot

36

ribulose-1,5-bisphosphate carboxylase/oxygenase large subunit, partial (chloroplast) [Cucumis sativus]	gi|545698970	20294.4	6.58		7	302	100	266	100

Protein Group

putative ribulose-1,5-bisphosphate carboxylase/oxygenase large subunit, partial (chloroplast) [Cucumis sativus]	gi|125380586	20050.2	6.5900 001525 8789

Peptide Information

Calc. Mass	Obsrv. Mass	± da	± ppm	Start Seq.	End Seq.	Sequence	Ion Score	C. I. %	Modification	Rank	Result Type
805.4818	805.5008	0.019	24	131	137	IPTAYIK					Mascot
1021.5312	1021.5552	0.024	23	24	32	DTDILAAFR					Mascot
1021.5312	1021.5552	0.024	23	24	32	DTDILAAFR	77	100			Mascot
1407.6678	1407.6924	0.0246	17	13	23	LTYYTPEYETK					Mascot
1407.6678	1407.6924	0.0246	17	13	23	LTYYTPEYETK	82	100			Mascot
1465.7546	1465.786	0.0314	21	138	150	TFQGPPHGIQVER					Mascot
1465.7546	1465.786	0.0314	21	138	150	TFQGPPHGIQVER	107	100			Mascot
1502.8512	1502.7584	-0.0928	-62	156	168	YGRPLLGCTIKPK			Carbamidomethyl (C)[8]		Mascot
2410.1814	2410.2292	0.0478	20	13	32	LTYYTPEYETKDTDILAAF R					Mascot
3854.8721	3855.0376	0.1655	43	33	70	VTPQPGVPPEEAGAAVA AESSTGTWTTVWTDGLT SLDR					Mascot

37

ribulose-1,5-bisphosphate carboxylase/oxygenase large subunit, partial (chloroplast) [Cucumis sativus]	gi|545698970	20294.4	6.58		5	280	100	261	100

Protein Group

putative ribulose-1,5-bisphosphate	gi|125380586	20050.2	6.5900

carboxylase/oxygenase large subunit, partial (chloroplast) [Cucumis sativus] 001525 8789

Peptide Information

Calc. Mass	Obsrv. Mass	± da	± ppm	Start Seq.	End Seq.	Sequence	Ion Score	C. I. %	Modification	Rank	Result Type
805.4818	805.5041	0.0223	28	131	137	IPTAYIK					Mascot
1021.5312	1021.5586	0.0274	27	24	32	DTDILAAFR					Mascot
1021.5312	1021.5586	0.0274	27	24	32	DTDILAAFR	71	99.998			Mascot
1407.6678	1407.6981	0.0303	22	13	23	LTYYTPEYETK					Mascot
1407.6678	1407.6981	0.0303	22	13	23	LTYYTPEYETK	63	99.991			Mascot
1465.7546	1465.7893	0.0347	24	138	150	TFQGPPHGIQVER					Mascot
1465.7546	1465.7893	0.0347	24	138	150	TFQGPPHGIQVER	127	100			Mascot
2410.1814	2410.2268	0.0454	19	13	32	LTYYTPEYETKDTDILAAFR					Mascot

38 PREDICTED: small heat shock protein, chloroplastic-like isoform X1 [Cucumis sativus] gi|778675414 20842.3 5.03 10 506 100 453 100

Peptide Information

Calc. Mass	Obsrv. Mass	± da	± ppm	Start Seq.	End Seq.	Sequence	Ion Score	C. I. %	Modification	Rank	Result Type
1008.3752	1008.4065	0.0313	31	157	164	ESEDEEDR					Mascot
1108.527	1108.5588	0.0318	29	120	128	EDDNALYLR					Mascot
1108.527	1108.5588	0.0318	29	120	128	EDDNALYLR	82	100			Mascot
1164.4763	1164.5414	0.0651	56	157	165	ESEDEEDRR					Mascot
1243.7257	1243.7493	0.0236	19	141	151	VSVEQNTLIIK					Mascot
1243.7257	1243.7493	0.0236	19	141	151	VSVEQNTLIIK	74	99.999			Mascot
1602.8738	1602.897	0.0232	14	171	184	LDLPANLYELNSIK					Mascot
1602.8738	1602.897	0.0232	14	171	184	LDLPANLYELNSIK	94	100			Mascot
1693.8181	1693.8523	0.0342	20	115	128	GWDVKEDDNALYLR					Mascot
1757.8282	1757.8767	0.0485	28	69	84	YPGFGGNVFDPFSPTR					Mascot
1757.8282	1757.8767	0.0485	28	69	84	YPGFGGNVFDPFSPTR	110	100			Mascot
1983.915	1984.0088	0.0938	47	120	136	EDDNALYLRMDMPGLSK			Oxidation (M)[10]		Mascot
2028.9563	2029.0087	0.0524	26	67	84	DRYPGFGGNVFDPFSPTR					Mascot

2028.9563	2029.0087	0.0524	26	67	84	DRYPGFGGNVFDPFSPTR	65	99.992			Mascot
2528.2161	2528.3149	0.0988	39	85	106	SLSQVLNLMDQFMEDPFLAASR			Oxidation (M)[9]		Mascot
2544.2109	2544.2766	0.0657	26	85	106	SLSQVLNLMDQFMEDPFLAASR			Oxidation (M)[9,13]		Mascot
2544.2109	2544.2766	0.0657	26	85	106	SLSQVLNLMDQFMEDPFLAASR	30	75.566	Oxidation (M)[9,13]		Mascot

39	PREDICTED: small heat shock protein, chloroplastic-like isoform X2 [Cucumis melo]	gi|659095978	23821.1	6.36	11	492	100	438	100

Peptide Information

Calc. Mass	Obsrv. Mass	± da	± ppm	Start Seq.	End Seq.	Sequence	Ion Score	C. I. %	Modification	Rank	Result Type
860.4584	860.4479	-0.0105	-12	51	57	SLDVDRR					Mascot
864.4608	864.454	-0.0068	-8	1	8	MASSIALR			Oxidation (M)[1]		Mascot
1008.3752	1008.4141	0.0389	39	156	163	ESEDEEDR					Mascot
1108.527	1108.5695	0.0425	38	119	127	EDDNALYLR					Mascot
1108.527	1108.5695	0.0425	38	119	127	EDDNALYLR	80	100			Mascot
1164.4763	1164.5469	0.0706	61	156	164	ESEDEEDRR					Mascot
1243.7257	1243.7634	0.0377	30	140	150	VSVEQNTLIIK					Mascot
1243.7257	1243.7634	0.0377	30	140	150	VSVEQNTLIIK	65	99.993			Mascot
1602.8738	1602.9163	0.0425	27	170	183	LDLPANLYELNSIK					Mascot
1602.8738	1602.9163	0.0425	27	170	183	LDLPANLYELNSIK	111	100			Mascot
1693.8181	1693.8737	0.0556	33	114	127	GWDVKEDDNALYLR					Mascot
1701.7908	1701.8545	0.0637	37	69	83	YPGFGDVFDPFSPTR					Mascot
1701.7908	1701.8545	0.0637	37	69	83	YPGFGDVFDPFSPTR	79	100			Mascot
1972.9188	1972.9835	0.0647	33	67	83	DRYPGFGDVFDPFSPTR					Mascot
1972.9188	1972.9835	0.0647	33	67	83	DRYPGFGDVFDPFSPTR	87	100			Mascot
2544.2109	2544.2954	0.0845	33	84	105	SLSQVLNLMDQFMEDPFLAASR			Oxidation (M)[9,13]		Mascot
2544.2109	2544.2954	0.0845	33	84	105	SLSQVLNLMDQFMEDPFLAASR	16	0	Oxidation (M)[9,13]		Mascot

40

hypothetical protein Csa_3G778410 [Cucumis sativus]	gi|700204052	29807.3	8.32		10	442	100	396	100
**Protein Group**									
PREDICTED: 2-Cys peroxiredoxin BAS1, chloroplastic-like [Cucumis melo]	gi|659084460	30025.4		8.3199996948 2422					
PREDICTED: 2-Cys peroxiredoxin BAS1, chloroplastic-like [Cucumis sativus]	gi|449437450	29807.3		8.3199996948 2422					

Peptide Information

Calc. Mass	Obsrv. Mass	± da	± ppm	Start Seq.	End Seq.	Sequence	Ion Score	C. I. %	Modification	Rank	Result Type
805.4818	805.4807	-0.0011	-1	202	208	GLFIIDK					Mascot
809.4403	809.4319	-0.0084	-10	103	109	LSEYIGK					Mascot
834.4316	834.4313	-0.0003	0	225	231	SVDETKR					Mascot
1058.5154	1058.5884	0.073	69	264	273	GSKEYFSAVA					Mascot
1501.8373	1501.8303	-0.007	-5	188	201	SYGVLIPDQGIALR					Mascot
1501.8373	1501.8303	-0.007	-5	188	201	SYGVLIPDQGIALR	137	100			Mascot
1735.9449	1735.9358	-0.0091	-5	209	224	EGIIQHSTINNLAIGR					Mascot
1735.9449	1735.9358	-0.0091	-5	209	224	EGIIQHSTINNLAIGR	122	100			Mascot
1748.9066	1748.8732	-0.0334	-19	167	183	SGGLGDLQYPLVSDVTK					Mascot
1748.9066	1748.8732	-0.0334	-19	167	183	SGGLGDLQYPLVSDVTK	71	99.998			Mascot
1877.0016	1876.9523	-0.0493	-26	166	183	KSGGLGDLQYPLVSDVTK					Mascot
1877.0016	1876.9523	-0.0493	-26	166	183	KSGGLGDLQYPLVSDVTK	28	63.595			Mascot
2522.4089	2522.4243	0.0154	6	202	224	GLFIIDKEGIIQHSTINNLAIGR					Mascot
2522.4089	2522.4243	0.0154	6	202	224	GLFIIDKEGIIQHSTINNLAIGR	38	96.733			Mascot
2857.3826	2857.3992	0.0166	6	232	256	TLQALQYVQENPDEVCPAGWKPGEK			Carbamidomethyl (C)[16]		Mascot

41

ribulose-1,5-bisphosphate carboxylase/oxygenase large	gi|108951132	51126.8	5.91	7	142	100	128	100

subunit, partial (chloroplast) [Cucumis hirsutus]

Peptide Information

Calc. Mass	Obsrv. Mass	± da	± ppm	Start Seq.	End Seq.	Sequence	Ion Score	C. I. %	Modification	Rank	Result Type
805.4818	805.4688	-0.013	-16	126	132	IPTAYIK					Mascot
1021.5312	1021.5266	-0.0046	-5	19	27	DTDILAAFR					Mascot
1021.5312	1021.5266	-0.0046	-5	19	27	DTDILAAFR	70	99.998			Mascot
1037.4899	1037.495	0.0051	5	337	344	DDFIEKDR					Mascot
1407.6678	1407.6483	-0.0195	-14	8	18	LTYYTPEYETK					Mascot
1407.6678	1407.6483	-0.0195	-14	8	18	LTYYTPEYETK	57	99.959			Mascot
1950.916	1950.9142	-0.0018	-1	223	239	GHYLNATAGTCEEMIKR			Carbamidomethyl (C)[11]		Mascot
2066.0085	2065.9509	-0.0576	-28	306	325	MSGGDHIHAGTVVGKLE GER			Oxidation (M)[1]		Mascot
2189.0947	2188.9033	-0.1914	-87	204	222	FLFCAEAIFKSQAETGEIK			Carbamidomethyl (C)[4]		Mascot

42

Cytosolic class II low molecular weight heat shock protein [Cucumis sativus] gi|700200202 17479.1 5.54 7 347 100 304 100

Protein Group

PREDICTED: 17.9 kDa class II heat shock protein-like [Cucumis sativus] gi|449447155 17479.1 5.5399999618 5303

Peptide Information

Calc. Mass	Obsrv. Mass	± da	± ppm	Start Seq.	End Seq.	Sequence	Ion Score	C. I. %	Modification	Rank	Result Type
800.4876	800.4439	-0.0437	-55	150	156	IVEVKVN					Mascot
974.4975	974.4899	-0.0076	-8	43	52	AMAATPADVK					Mascot
974.4975	974.4899	-0.0076	-8	43	52	AMAATPADVK	53	99.902			Mascot
990.4924	990.4882	-0.0042	-4	43	52	AMAATPADVK			Oxidation (M)[2]		Mascot
990.4924	990.4882	-0.0042	-4	43	52	AMAATPADVK	32	85.627	Oxidation (M)[2]		Mascot
1857.9092	1857.8846	-0.0246	-13	53	68	EYPNSYVFVVDMPGLK					Mascot
1857.9092	1857.8846	-0.0246	-13	53	68	EYPNSYVFVVDMPGLK	86	100			Mascot
1873.9042	1873.8755	-0.0287	-15	53	68	EYPNSYVFVVDMPGLK			Oxidation (M)[12]		Mascot
1873.9042	1873.8755	-0.0287	-15	53	68	EYPNSYVFVVDMPGLK	89	100	Oxidation (M)[12]		Mascot

2198.1299	2198.1211	-0.0088	-4	69	88	VGDIQVQVEDDNVLLISG ER					Mascot
2198.1299	2198.1211	-0.0088	-4	69	88	VGDIQVQVEDDNVLLISG ER	161	100			Mascot
2662.2739	2662.2188	-0.0551	-21	5	28	IMGIDSPIFSTLQHVMDLA DEADK			Oxidation (M)[2]		Mascot
2889.4663	2889.4297	-0.0366	-13	113	139	FVLPENANTDAISAVCQD GVLTVTVQK			Carbamidomethyl (C)[16]		Mascot
3017.5613	3017.5491	-0.0122	-4	112	139	KFVLPENANTDAISAVCQ DGVLTVTVQK			Carbamidomethyl (C)[17]		Mascot

43

PREDICTED: oxygen-evolving enhancer protein 2, chloroplastic [Cucumis sativus]	gi|449460024	28121.2	8.61	15	383	100	291	100

Protein Group

23kDa polypeptide of the oxygen-evolving complex of photosystem II [Cucumis sativus]	gi|6691487	28121.2	8.6099 996566 7725
Oxygen-evolving enhancer protein 2 [Cucumis sativus]	gi|700198352	28121.2	8.6099 996566 7725
RecName: Full=Oxygen-evolving enhancer protein 2, chloroplastic; Short=OEE2; AltName: Full=23 kDa subunit of oxygen evolving system of photosystem II; AltName: Full=23 kDa thylakoid membrane protein; AltName: Full=OEC 23 kDa subunit; AltName: Full=O	gi|11134156	28121.2	8.6099 996566 7725

Peptide Information

Calc. Mass	Obsrv. Mass	± da	± ppm	Start Seq.	End Seq.	Sequence	Ion Score	C. I. %	Modification	Rank	Result Type
807.4611	807.4512	-0.0099	-12	161	167	VDYLLGK					Mascot
849.3585	849.3562	-0.0023	-3	212	220	TADGDEGGK					Mascot
945.5152	945.5113	-0.0039	-4	118	125	EFPGQVLR					Mascot
945.5152	945.5113	-0.0039	-4	118	125	EFPGQVLR	50	99.732			Mascot
1228.6644	1228.5475	-0.1169	-95	19	30	SSSSPRQAALPK					Mascot

1230.6589	1230.6499	-0.009	-7	116	125	EREFPGQVLR					Mascot
1230.6589	1230.6499	-0.009	-7	116	125	EREFPGQVLR					Mascot
1255.5841	1255.5746	-0.0095	-8	150	160	DFGSPEEFLSK					Mascot
1255.5841	1255.5746	-0.0095	-8	150	160	DFGSPEEFLSK	39	96.679			Mascot
1262.6416	1262.6353	-0.0063	-5	202	211	DYYFVSVLTR					Mascot
1262.6416	1262.6353	-0.0063	-5	202	211	DYYFVSVLTR	64	99.989			Mascot
1296.6907	1296.675	-0.0157	-12	221	232	HQLITATVNDGK					Mascot
1299.658	1299.6407	-0.0173	-13	251	263	KFVEGAASSFSVA					Mascot
1563.6963	1563.6785	-0.0178	-11	91	104	SNTDYLPYSGDGFK					Mascot
1563.6963	1563.6785	-0.0178	-11	91	104	SNTDYLPYSGDGFK	104	100			Mascot
1583.7952	1583.7832	-0.012	-8	147	160	SIKDFGSPEEFLSK					Mascot
2127.0312	2127.0012	-0.03	-14	212	232	TADGDEGGKHQLITATVN DGK					Mascot
2285.0569	2285.0171	-0.0398	-17	126	145	YEDNFDSNSNLSVIINPT					Mascot

Project 1\Sample project201(2285.0171 **258** Mascot

						DK					
2413.1519	2413.1145	-0.0374	-15	126	146	YEDNFDSNSNLSVIINPT DKK					Mascot
2413.1519	2413.1145	-0.0374	-15	126	146	YEDNFDSNSNLSVIINPT DKK	35	90.854			Mascot
2720.301	2720.2512	-0.0498	-18	174	201	TASEGGFDPDAVATANIL EATASNVNGK					Mascot

44

ribulose-1,5-bisphosphate carboxylase/oxygenase large subunit, partial (chloroplast) [Cucumis hystrix] gi|111182702 51456.9 6 19 411 100 323 100

Peptide Information

Calc. Mass	Obsrv. Mass	± da	± ppm	Start Seq.	End Seq.	Sequence	Ion Score	C. I. %	Modification	Rank	Result Type
805.4818	805.4727	-0.0091	-11	129	135	IPTAYIK					Mascot
910.4451	910.4388	-0.0063	-7	177	183	AVYECLR			Carbamidomethyl (C)[5]		Mascot
910.4451	910.4388	-0.0063	-7	177	183	AVYECLR	32	84.509	Carbamidomethyl (C)[5]		Mascot
962.4789	962.4694	-0.0095	-10	217	225	SQAETGEIK					Mascot

1021.5312	1021.5197	-0.0115	-11	22	30	DTDILAAFR				Mascot
1021.5312	1021.5197	-0.0115	-11	22	30	DTDILAAFR	62	99.985		Mascot
1154.5636	1154.6003	0.0367	32	293	301	QKNHGMHFR				Mascot
1188.6122	1188.6075	-0.0047	-4	207	216	FLFCAEALFK				Mascot
1245.6337	1245.6146	-0.0191	-15	207	216	FLFCAEALFK			Carbamidomethyl (C)[4]	Mascot
1275.7307	1275.6885	-0.0422	-33	329	339	EITLGFVDLLR				Mascot
1407.6678	1407.6469	-0.0209	-15	11	21	LTYYTPEYETK				Mascot
1407.6678	1407.6469	-0.0209	-15	11	21	LTYYTPEYETK	70	99.997		Mascot
1445.8297	1445.787	-0.0427	-30	154	166	YGRPLLGCTIKPK				Mascot
1451.6219	1451.6426	0.0207	14	191	202	DDENVNSQPFMR				Mascot
1465.7217	1465.7321	0.0104	7	309	323	MSGGDHIHAGTVVGK				Mascot
1465.7546	1465.7321	-0.0225	-15	136	148	TFQGPPHGIQVER	93	100		Mascot
1481.7167	1481.7378	0.0211	14	309	323	MSGGDHIHAGTVVGK			Oxidation (M)[1]	Mascot
1502.8512	1502.8226	-0.0286	-19	154	166	YGRPLLGCTIKPK			Carbamidomethyl (C)[8]	Mascot
1502.8512	1502.8226	-0.0286	-19	154	166	YGRPLLGCTIKPK			Carbamidomethyl (C)[8]	Mascot
1516.7617	1516.7667	0.005	3	205	216	DRFLFCAEALFK			Carbamidomethyl (C)[6]	Mascot
1828.7662	1828.7209	-0.0453	-25	226	241	GHYLNATAGTCEEMMK			Carbamidomethyl (C)[11], Oxidation (M)[14]	Mascot
1844.7612	1844.7142	-0.047	-25	226	241	GHYLNATAGTCEEMMK			Carbamidomethyl (C)[11], Oxidation (M)[14,15]	Mascot
1904.9211	1904.8131	-0.108	-57	436	452	EASKWSPELAAACEVWK				Mascot
2000.8623	2000.8138	-0.0485	-24	226	242	GHYLNATAGTCEEMMKR			Carbamidomethyl (C)[11], Oxidation (M)[14,15]	Mascot
2169.9871	2169.9568	-0.0303	-14	184	202	GGLDFTKDDENVNSQPF MR				Mascot
2185.9819	2185.9392	-0.0427	-20	184	202	GGLDFTKDDENVNSQPF			Oxidation (M)[18]	Mascot

Project 1\Sample project201(2185.9392 **266** Mascot

						MR				
2185.9819	2185.9392	-0.0427	-20	184	202	GGLDFTKDDENVNSQPF MR	68	99.996	Oxidation (M)[18]	Mascot
3010.4187	3010.4055	-0.0132	-4	248	274	ELGAPIVMHDYLTGGFTA NTSLAHYCR			Carbamidomethyl (C)[26], Oxidation (M)[8]	Mascot
3854.8721	3854.8901	0.018	5	31	68	VTPQPGVPPEEAGAAVA AESSTGTWTTVWTDGLT SLDR				Mascot

45

hypothetical protein Csa_7G195260 [Cucumis sativus]	gi|700188884	43082.5	6.19	19	708	100	601	100

Protein Group

PREDICTED: fructose-bisphosphate aldolase, cytoplasmic isozyme 1 [Cucumis sativus]	gi|449444016	43082.5	6.1900 000572 2046

Peptide Information

Calc. Mass	Obsrv. Mass	± da	± ppm	Start Seq.	End Seq.	Sequence	Ion Score	C. I. %	Modification	Rank	Result Type
816.3708	816.3594	-0.0114	-14	176	181	CAQFYK			Carbamidomethyl (C)[1]		Mascot
865.4665	865.4546	-0.0119	-14	54	60	YAEELIK					Mascot
888.5149	888.4905	-0.0244	-27	345	352	ALQQSTLK					Mascot
905.4839	905.4731	-0.0108	-12	364	371	AQAAFLER					Mascot
905.4839	905.4731	-0.0108	-12	364	371	AQAAFLER	64	99.99			Mascot
1164.5677	1164.5123	-0.0554	-48	372	382	CKANSDATLGK			Carbamidomethyl (C)[1]		Mascot
1417.7281	1417.7091	-0.019	-13	86	98	LASISVENVESNR					Mascot
1417.7281	1417.7091	-0.019	-13	86	98	LASISVENVESNR	40	97.676			Mascot
1479.7876	1479.733	-0.0546	-37	243	256	CAAATEIVLAAVYK			Carbamidomethyl (C)[1]		Mascot
1573.8292	1573.8007	-0.0285	-18	85	98	RLASISVENVESNR					Mascot
1587.8352	1587.8457	0.0105	7	2	15	HAWALILPGYSMTK					Mascot
1587.8741	1587.8457	-0.0284	-18	284	298	VAPEVVAEYTVAALR	135	100			Mascot
1743.9752	1743.9365	-0.0387	-22	284	299	VAPEVVAEYTVAALRR					Mascot
1908.8611	1908.812	-0.0491	-26	383	402	YGGGSGGGVASESLYEA GYK					Mascot
2071.9243	2071.8779	-0.0464	-22	383	403	YGGGSGGGVASESLYEA GYKY					Mascot
2071.9243	2071.8779	-0.0464	-22	383	403	YGGGSGGGVASESLYEA GYKY	122	100			Mascot
2095.0779	2095.0432	-0.0347	-17	195	214	IGPSEPSELSIQQNAQGL AR					Mascot
2095.0779	2095.0432	-0.0347	-17	195	214	IGPSEPSELSIQQNAQGL AR	170	100			Mascot
2282.0896	2282.0486	-0.041	-18	153	175	GTVELAGTNGETTTQGF DSLGAR					Mascot
2282.0896	2282.0486	-0.041	-18	153	175	GTVELAGTNGETTTQGF	71	99.998			Mascot

						DSLGAR				
2624.2798	2624.2314	-0.0484	-18	150	175	VDKGTVELAGTNGETTT QGFDSLGAR				Mascot

2798.4871	2798.4294	-0.0577	-21	257	283	ALSDHHVLLEGTLLKPNM VTPGSGSPK				Mascot
2814.4819	2814.4153	-0.0666	-24	257	283	ALSDHHVLLEGTLLKPNM VTPGSGSPK			Oxidation (M)[18]	Mascot
3025.584	3025.5391	-0.0449	-15	300	329	TVPAAVPGVVFLSGGQS EEEATLNLNAINK				Mascot
3147.603	3147.5696	-0.0334	-11	215	242	YAIICQENGLVPIVEPEILT DGPHDINK			Carbamidomethyl (C)[5]	Mascot

46

hypothetical protein Csa_3G168970 [Cucumis sativus]	gi|700202053	53566.9	7.68	23	514	100	387	100
**Protein Group**								
PREDICTED: leghemoglobin reductase [Cucumis sativus]	gi|449459772	53566.9	7.6799 998283 3862					

Peptide Information

Calc. Mass	Obsrv. Mass	± da	± ppm	Start Seq.	End Seq.	Sequence	Ion Score	C. I. %	Modification	Rank	Result Type
807.4359	807.4294	-0.0065	-8	409	415	ELGVAYR					Mascot
969.4611	969.4463	-0.0148	-15	419	426	FPFMANSR					Mascot
980.506	980.493	-0.013	-13	104	112	HAFANHGVK					Mascot
985.456	985.4406	-0.0154	-16	419	426	FPFMANSR			Oxidation (M)[4]		Mascot
1072.5885	1072.5774	-0.0111	-10	189	198	SLPGITIDEK					Mascot
1091.6208	1091.6129	-0.0079	-7	409	418	ELGVAYRVGK					Mascot
1103.6307	1103.6157	-0.015	-14	178	188	SIIIATGSDVK					Mascot
1131.6005	1131.5734	-0.0271	-24	277	288	VVGVDTSGNGVK					Mascot
1149.6151	1149.5917	-0.0234	-20	314	324	TPFTSGLGLEK					Mascot
1459.725	1459.6998	-0.0252	-17	494	506	EAAMATYDKPIHI					Mascot
1480.7036	1480.7084	0.0048	3	481	493	VCHAHPTMSEALK			Carbamidomethyl (C)[2]		Mascot
1496.6985	1496.6671	-0.0314	-21	481	493	VCHAHPTMSEALK			Carbamidomethyl (C)[2], Oxidation (M)[8]		Mascot

	1513.8837	1513.8464	-0.0373	-25	200	214	IISSTGALALTEIPK				Mascot
	1523.7423	1523.7306	-0.0117	-8	91	103	ALLHSSHMYHEAK				Mascot
	1539.7373	1539.705	-0.0323	-21	91	103	ALLHSSHMYHEAK			Oxidation (M)[8]	Mascot
	1594.7418	1594.7166	-0.0252	-16	364	378	AEEDGVACVEFLAGK			Carbamidomethyl (C)[8]	Mascot
	1594.7418	1594.7166	-0.0252	-16	364	378	AEEDGVACVEFLAGK	49	99.759	Carbamidomethyl (C)[8]	Mascot
	1633.8037	1633.7706	-0.0331	-20	75	90	GTLGGTCLNVGCIPSK			Carbamidomethyl (C)[7,12]	Mascot
	1652.8022	1652.7672	-0.035	-21	113	127	FSSVEVDLPAMMAQK				Mascot
	1668.7972	1668.7642	-0.033	-20	113	127	FSSVEVDLPAMMAQK			Oxidation (M)[11]	Mascot
	1669.9847	1669.9542	-0.0305	-18	199	214	RIISSTGALALTEIPK				Mascot
	1684.7921	1684.7463	-0.0458	-27	113	127	FSSVEVDLPAMMAQK			Oxidation (M)[11,12]	Mascot
	1684.7921	1684.7463	-0.0458	-27	113	127	FSSVEVDLPAMMAQK	5	0	Oxidation (M)[11,12]	Mascot
**Project 1\Sample project201(**		1684.7463									**285** Mascot
	1789.9048	1789.8582	-0.0466	-26	74	90	RGTLGGTCLNVGCIPSK			Carbamidomethyl (C)[8,13]	Mascot
	2030.0653	2030.0217	-0.0436	-21	156	175	LISPSEVSVDTIDGGNTV VK				Mascot
	2030.0653	2030.0217	-0.0436	-21	156	175	LISPSEVSVDTIDGGNTV VK	26	43.951		Mascot
	2397.2637	2397.2153	-0.0484	-20	341	363	FATNVPGIYAIGDVIPGPM LAHK			Oxidation (M)[19]	Mascot
	2478.2434	2478.2109	-0.0325	-13	235	257	LGSEVTVVEFASEIVPTM DAEVR				Mascot
	2478.2434	2478.2109	-0.0325	-13	235	257	LGSEVTVVEFASEIVPTM DAEVR	10	0		Mascot
	2494.2383	2494.2063	-0.032	-13	235	257	LGSEVTVVEFASEIVPTM DAEVR			Oxidation (M)[18]	Mascot
	2494.2383	2494.2063	-0.032	-13	235	257	LGSEVTVVEFASEIVPTM DAEVR	60	99.98	Oxidation (M)[18]	Mascot
	2510.1504	2510.2249	0.0745	30	364	386	AEEDGVACVEFLAGKTG HVDYDK			Carbamidomethyl (C)[8]	Mascot
	2513.3093	2513.2825	-0.0268	-11	289	313	LTLEPAAGGDQTTLETDV VLVSAGR				Mascot
	2513.3093	2513.2825	-0.0268	-11	289	313	LTLEPAAGGDQTTLETDV VLVSAGR	196	100		Mascot
	2554.2937	2554.26	-0.0337	-13	379	402	TGHVDYDKVPGVVYTHP EVASVGK				Mascot

2554.2937	2554.26	-0.0337	-13	379	402	TGHVDYDKVPGVVYTHP EVASVGK	52	99.873			Mascot

47

Serine hydroxymethyltransferase [Cucumis sativus]	gi|700193477	57836.6	8.12	30	702	100	512	100

Protein Group

PREDICTED: serine hydroxymethyltransferase, mitochondrial [Cucumis sativus]	gi|449451373	57836.6	8.1199998855 5908

Peptide Information

Calc. Mass	Obsrv. Mass	± da	± ppm	Start Seq.	End Seq.	Sequence	Ion Score	C. I. %	Modification	Rank	Result Type
800.3937	800.3866	-0.0071	-9	239	244	LYDYAR					Mascot
841.4389	841.4309	-0.008	-10	295	301	GAMIFFR					Mascot
857.4338	857.4203	-0.0135	-16	295	301	GAMIFFR			Oxidation (M)[3]		Mascot
862.4893	862.4677	-0.0216	-25	130	136	RALEAFR					Mascot
919.5359	919.5207	-0.0152	-17	220	227	SATLFRPK					Mascot
933.4822	933.4613	-0.0209	-22	433	441	MGTPALTSR					Mascot
937.4625	937.4322	-0.0303	-32	348	355	QATTPEYK					Mascot
949.4771	949.4545	-0.0226	-24	433	441	MGTPALTSR			Oxidation (M)[1]		Mascot
981.4523	981.4293	-0.023	-23	496	503	QDVEEYAK					Mascot
999.453	999.4304	-0.0226	-23	104	112	YSEGYPGAR					Mascot
999.453	999.4304	-0.0226	-23	104	112	YSEGYPGAR	20	0			Mascot
1006.5316	1006.51	-0.0216	-21	356	363	AYQEQVLR					Mascot
1006.5316	1006.51	-0.0216	-21	356	363	AYQEQVLR	37	94.768			Mascot
1041.4888	1041.4667	-0.0221	-21	442	450	GFVEEDFAK					Mascot
1041.4888	1041.4667	-0.0221	-21	442	450	GFVEEDFAK	56	99.938			Mascot
1066.5931	1066.5669	-0.0262	-25	504	512	KFPTIGFEK					Mascot
1066.5931	1066.5669	-0.0262	-25	504	512	KFPTIGFEK	34	90.653			Mascot
1091.6207	1091.6011	-0.0196	-18	228	238	LIVAGASAYAR					Mascot
1091.6207	1091.6011	-0.0196	-18	228	238	LIVAGASAYAR	38	95.506			Mascot
1109.5474	1109.4901	-0.0573	-52	496	504	QDVEEYAKK					Mascot
1135.6074	1135.528	-0.0794	-70	2	11	ALAMAMALRR			Oxidation (M)[4,6]		Mascot
1173.531	1173.5048	-0.0262	-22	313	321	EVLYDYEDK					Mascot
1180.6685	1180.6067	-0.0618	-52	406	416	VLESVHIAANK					Mascot

**Project 1\Sample project201(**		1180.6067									**293**	Mascot
	1336.6743	1336.6366	-0.0377	-28	493	503	NLKQDVEEYAK				Mascot	
	1368.6212	1368.5918	-0.0294	-21	364	375	NCSNFAQSLAEK			Carbamidomethyl (C)[2]	Mascot	
	1569.8054	1569.7697	-0.0357	-23	417	432	NTVPGDVSAMVPGGIR				Mascot	
	1569.8054	1569.7697	-0.0357	-23	417	432	NTVPGDVSAMVPGGIR	52	99.835		Mascot	
	1585.8003	1585.757	-0.0433	-27	417	432	NTVPGDVSAMVPGGIR			Oxidation (M)[10]	Mascot	
	1585.8003	1585.757	-0.0433	-27	417	432	NTVPGDVSAMVPGGIR	88	100	Oxidation (M)[10]	Mascot	
	1660.8403	1660.8091	-0.0312	-19	191	204	ISAVSIFFETMPYR				Mascot	
	1676.8353	1676.793	-0.0423	-25	191	204	ISAVSIFFETMPYR			Oxidation (M)[11]	Mascot	
	1676.8353	1676.793	-0.0423	-25	191	204	ISAVSIFFETMPYR	4	0	Oxidation (M)[11]	Mascot	
	1804.9303	1804.8801	-0.0502	-28	190	204	KISAVSIFFETMPYR			Oxidation (M)[12]	Mascot	
	1816.8236	1816.7786	-0.045	-25	205	219	LDESTGYIDYDQLER				Mascot	
	1816.8236	1816.7786	-0.045	-25	205	219	LDESTGYIDYDQLER	150	100		Mascot	
	2028.076	2028.0143	-0.0617	-30	376	394	GYELVSGGTDNHLVLVNLK				Mascot	
	2028.076	2028.0143	-0.0617	-30	376	394	GYELVSGGTDNHLVLVNLK	84	100		Mascot	
	2040.8678	2040.8157	-0.0521	-26	113	129	YYGGNEYIDMAESLCQK			Carbamidomethyl (C)[15]	Mascot	
	2052.947	2052.9016	-0.0454	-22	476	492	DFLTTMESTPYFQSEIK			Oxidation (M)[6]	Mascot	
	2056.8628	2056.7952	-0.0676	-33	113	129	YYGGNEYIDMAESLCQK			Carbamidomethyl (C)[15], Oxidation (M)[10]	Mascot	
	2278.1311	2278.0754	-0.0557	-24	474	492	LKDFLTTMESTPYFQSEIK				Mascot	
	2291.1238	2291.0686	-0.0552	-24	169	189	IMALDLPHGGHLSHGYQTDTK				Mascot	
	2294.1262	2294.0566	-0.0696	-30	474	492	LKDFLTTMESTPYFQSEIK			Oxidation (M)[8]	Mascot	
	2307.1187	2307.0474	-0.0713	-31	169	189	IMALDLPHGGHLSHGYQTDTK			Oxidation (M)[2]	Mascot	
	2435.2136	2435.1499	-0.0637	-26	169	190	IMALDLPHGGHLSHGYQTDTKK			Oxidation (M)[2]	Mascot	
	2653.4573	2653.4104	-0.0469	-18	322	347	INQAVFPGLQGGPHNHTITGLAVALK				Mascot	
	2770.3638	2770.2722	-0.0916	-33	78	103	GLELIPSENFTSVSVMQAVGSVMTNK			Oxidation (M)[16,23]	Mascot	

48

hypothetical protein Csa_3G889840 [Cucumis sativus]	gi|700205095	40251.8	7.66		19	538	100	430	100
**Protein Group**									
PREDICTED: thylakoid lumenal 29 kDa protein, chloroplastic [Cucumis sativus]	gi|449436992	40251.8		7.6599998474 1211					

Peptide Information

Calc. Mass	Obsrv. Mass	± da	± ppm	Start Seq.	End Seq.	Sequence	Ion Score	C. I. %	Modification	Rank	Result Type
844.4271	844.4218	-0.0053	-6	152	160	SGGPNGSIR					Mascot
896.5087	896.4944	-0.0143	-16	119	126	STLYTAIK					Mascot
910.488	910.4694	-0.0186	-20	111	118	SEFLSSIK					Mascot
917.5203	917.5049	-0.0154	-17	278	286	LSAIGFGPR					Mascot
917.5203	917.5049	-0.0154	-17	278	286	LSAIGFGPR	62	99.987			Mascot
965.5414	965.5253	-0.0161	-17	212	220	STFLASAIR					Mascot
965.5414	965.5253	-0.0161	-17	212	220	STFLASAIR	39	97.385			Mascot
1143.4913	1143.4722	-0.0191	-17	252	262	SDAEAPDPEGR					Mascot
1143.4913	1143.4722	-0.0191	-17	252	262	SDAEAPDPEGR	18	0			Mascot
1174.658	1174.639	-0.019	-16	276	286	EKLSAIGFGPR					Mascot
1194.6477	1194.5807	-0.067	-56	109	118	QRSEFLSSIK					Mascot
1262.666	1262.6411	-0.0249	-20	173	184	LSAAMSLIEEAK					Mascot
1278.661	1278.6152	-0.0458	-36	173	184	LSAAMSLIEEAK			Oxidation (M)[5]		Mascot
1364.6805	1364.6488	-0.0317	-23	161	172	FSSEISRPENAK					Mascot
1364.6805	1364.6488	-0.0317	-23	161	172	FSSEISRPENAK	40	97.554			Mascot
1390.761	1390.725	-0.036	-26	173	185	LSAAMSLIEEAKK					Mascot
1406.756	1406.6808	-0.0752	-53	173	185	LSAAMSLIEEAKK			Oxidation (M)[5]		Mascot
1888.0175	1887.9656	-0.0519	-27	193	211	GGPISYADLIQLAAQSGVK					Mascot
1888.0175	1887.9656	-0.0519	-27	193	211	GGPISYADLIQLAAQSGVK	147	100			Mascot
2056.0332	2055.9746	-0.0586	-29	324	341	ETVSQTDYEVDLITTLTK					Mascot
2060.0334	2059.9802	-0.0532	-26	342	359	ISSLGQQINYEAYSYPVK					Mascot
2060.0334	2059.9802	-0.0532	-26	342	359	ISSLGQQINYEAYSYPVK	123	100			Mascot

2105.0088	2104.9646	-0.0442	-21	229	247	GSLLYSAYGSNGQWGLF					Mascot

Calc. Mass	Obsrv. Mass	± da	± ppm	Start Seq.	End Seq.	Sequence	Ion Score	C. I. %	Modification	Rank	Result Type
						ER					
2188.1284	2188.0696	-0.0588	-27	342	360	ISSLGQQINYEAYSYPVKK					Mascot
2272.207	2272.1265	-0.0805	-35	128	148	NPDIVPSLLTLALNDAVTYDK					Mascot
2299.1665	2299.1179	-0.0486	-21	322	341	SRETVSQTDYEVDLITTLTK					Mascot
2400.302	2400.2385	-0.0635	-26	127	148	KNPDIVPSLLTLALNDAVTYDK					Mascot

49									
	hypothetical protein Csa_4G025100 [Cucumis sativus]	gi|700198022	29887.8	8.96	8	333	100	310	100

Protein Group

PREDICTED: peptide methionine sulfoxide reductase A1-like [Cucumis sativus]	gi|778690317	29887.8	8.9600000381 4697

Peptide Information

Calc. Mass	Obsrv. Mass	± da	± ppm	Start Seq.	End Seq.	Sequence	Ion Score	C. I. %	Modification	Rank	Result Type
831.3777	831.3558	-0.0219	-26	259	265	GCNDPIR			Carbamidomethyl (C)[2]		Mascot
939.4643	939.4312	-0.0331	-35	177	184	HDPTTVNR					Mascot
1137.5283	1137.4954	-0.0329	-29	185	194	QGNDVGTQYR					Mascot
1437.6646	1437.6165	-0.0481	-33	236	246	AEEYHQQYLEK					Mascot
1437.6646	1437.6165	-0.0481	-33	236	246	AEEYHQQYLEK	58	99.966			Mascot
1461.6897	1461.6306	-0.0591	-40	195	206	SGIYFYTPEQEK					Mascot
1461.6897	1461.6306	-0.0591	-40	195	206	SGIYFYTPEQEK	68	99.996			Mascot
1746.7792	1746.7336	-0.0456	-26	163	176	ECSYESLLDAFWAR			Carbamidomethyl (C)[2]		Mascot
1746.7792	1746.7336	-0.0456	-26	163	176	ECSYESLLDAFWAR	25	26.634	Carbamidomethyl (C)[2]		Mascot
1759.865	1759.7848	-0.0802	-46	195	209	SGIYFYTPEQEKAAR					Mascot
3463.5608	3463.5146	-0.0462	-13	127	156	TEVGYTQGHVHNPTYEDVCTGQTYHSEVVR			Carbamidomethyl (C)[19]		Mascot
3463.5608	3463.5146	-0.0462	-13	127	156	TEVGYTQGHVHNPTYEDVCTGQTYHSEVVR	159	100	Carbamidomethyl (C)[19]		Mascot

50									
	hypothetical protein Csa_3G116710 [Cucumis sativus]	gi|700201201	16450.5	6.3	7	308	100	269	100
	**Protein Group**								
	PREDICTED: nucleoside diphosphate kinase [Cucumis melo]	gi|659074723	16401.5	6.3000 001907 3486					
	PREDICTED: nucleoside diphosphate kinase [Cucumis sativus]	gi|449431942	16450.5	6.3000 001907 3486					

Peptide Information

Calc. Mass	Obsrv. Mass	± da	± ppm	Start Seq.	End Seq.	Sequence	Ion Score	C. I. %	Modification	Rank	Result Type
943.5571	943.5392	-0.0179	-19	16	24	GLVGEIISR					Mascot
943.5571	943.5392	-0.0179	-19	16	24	GLVGEIISR	13	0			Mascot
949.4738	949.4574	-0.0164	-17	103	111	GDFAIDVGR					Mascot
949.4738	949.4574	-0.0164	-17	103	111	GDFAIDVGR	51	99.816			Mascot
1384.6815	1384.6587	-0.0228	-16	112	124	NIIHGSDSVESAR					Mascot
1384.6815	1384.6587	-0.0228	-16	112	124	NIIHGSDSVESAR	40	97.591			Mascot
1641.9171	1641.8857	-0.0314	-19	87	102	IIGATKPVDSDVGTIR					Mascot
1641.9171	1641.8857	-0.0314	-19	87	102	IIGATKPVDSDVGTIR	134	100			Mascot
1663.8473	1663.8037	-0.0436	-26	2	15	EQSFIMIKPDGVQR			Oxidation (M)[6]		Mascot
1770.0121	1769.978	-0.0341	-19	86	102	KIIGATKPVDSDVGTIR					Mascot
1770.0121	1769.978	-0.0341	-19	86	102	KIIGATKPVDSDVGTIR	31	81.932			Mascot
1810.8827	1810.8362	-0.0465	-26	1	15	MEQSFIMIKPDGVQR			Oxidation (M)[1,7]		Mascot

51									
	PREDICTED: oxygen-evolving enhancer protein 2, chloroplastic [Cucumis sativus]	gi|449460024	28121.2	8.61	13	396	100	322	100
	**Protein Group**								
	23kDa polypeptide of the oxygen-evolving complex of photosystem II [Cucumis sativus]	gi|6691487	28121.2	8.6099 996566 7725					
	Oxygen-evolving enhancer protein 2 [Cucumis sativus]	gi|700198352	28121.2	8.6099 996566					

			7725
RecName: Full=Oxygen-evolving enhancer protein 2, chloroplastic; Short=OEE2; AltName: Full=23 kDa subunit of oxygen evolving system of photosystem II; AltName: Full=23 kDa thylakoid membrane protein; AltName: Full=OEC 23 kDa subunit; AltName: Full=O	gi|11134156	28121.2	8.6099<br>996566<br>7725

Peptide Information

Calc. Mass	Obsrv. Mass	± da	± ppm	Start Seq.	End Seq.	Sequence	Ion Score	C. I. %	Modification	Rank	Result Type
807.4611	807.4507	-0.0104	-13	161	167	VDYLLGK					Mascot
945.5152	945.5046	-0.0106	-11	118	125	EFPGQVLR					Mascot
945.5152	945.5046	-0.0106	-11	118	125	EFPGQVLR	48	99.638			Mascot
1230.6589	1230.6437	-0.0152	-12	116	125	EREFPGQVLR					Mascot
1255.5841	1255.5636	-0.0205	-16	150	160	DFGSPEEFLSK					Mascot
1255.5841	1255.5636	-0.0205	-16	150	160	DFGSPEEFLSK	45	99.274			Mascot
1262.6416	1262.6271	-0.0145	-11	202	211	DYYFVSVLTR					Mascot
1262.6416	1262.6271	-0.0145	-11	202	211	DYYFVSVLTR	59	99.971			Mascot
1299.658	1299.5592	-0.0988	-76	251	263	KFVEGAASSFSVA					Mascot
1563.6963	1563.6671	-0.0292	-19	91	104	SNTDYLPYSGDGFK					Mascot
1563.6963	1563.6671	-0.0292	-19	91	104	SNTDYLPYSGDGFK	54	99.903			Mascot
1583.7952	1583.7625	-0.0327	-21	147	160	SIKDFGSPEEFLSK					Mascot
2127.0312	2126.9905	-0.0407	-19	212	232	TADGDEGGKHQLITATVNDGK					Mascot
2285.0569	2285.0186	-0.0383	-17	126	145	YEDNFDSNSNLSVIINPTDK					Mascot
2395.1685	2395.1023	-0.0662	-28	2	24	ASTSCFLHHHALTAAARSSSPR					Mascot

Calc. Mass	Obsrv. Mass	± da	± ppm	Start Seq.	End Seq.	Sequence	Ion Score	C. I. %	Modification	Rank	Result Type
2413.1519	2413.1101	-0.0418	-17	126	146	YEDNFDSNSNLSVIINPTDKK					Mascot
2413.1519	2413.1101	-0.0418	-17	126	146	YEDNFDSNSNLSVIINPTDKK	115	100			Mascot
2720.301	2720.251	-0.05	-18	174	201	TASEGGFDPDAVATANILEATASNVNGK					Mascot

52

PREDICTED: LOW QUALITY PROTEIN: ribulose bisphosphate carboxylase large chain, partial [Cucumis melo]	gi|659133948	30149	6.23	10	358	100	315	100

Peptide Information

Calc. Mass	Obsrv. Mass	± da	± ppm	Start Seq.	End Seq.	Sequence	Ion Score	C. I. %	Modification	Rank	Result Type
830.4366	830.4368	0.0002	0	236	242	EGNEIIR					Mascot
914.405	914.3942	-0.0108	-12	102	108	NHGMHFR			Oxidation (M)[4]		Mascot
1059.5615	1059.5522	-0.0093	-9	218	227	VALEACVQAR					Mascot
1116.583	1116.5741	-0.0089	-8	218	227	VALEACVQAR			Carbamidomethyl (C)[6]		Mascot
1116.583	1116.5741	-0.0089	-8	218	227	VALEACVQAR	39	96.423	Carbamidomethyl (C)[6]		Mascot
1154.5636	1154.4771	-0.0865	-75	100	108	QKNHGMHFR					Mascot
1275.7307	1275.7148	-0.0159	-12	136	146	EITLGFVDLLR					Mascot
1275.7307	1275.7148	-0.0159	-12	136	146	EITLGFVDLLR	106	100			Mascot
1459.7402	1459.679	-0.0612	-42	12	23	DRFLFCAEAIFK					Mascot
1465.7217	1465.7052	-0.0165	-11	116	130	MSGGDHIHAGTVVGK					Mascot
1481.7167	1481.6917	-0.025	-17	116	130	MSGGDHIHAGTVVGK			Oxidation (M)[1]		Mascot
1481.7167	1481.6917	-0.025	-17	116	130	MSGGDHIHAGTVVGK	80	100	Oxidation (M)[1]		Mascot
1488.6927	1488.6687	-0.024	-16	260	271	EIKFEFEAMDTI			Oxidation (M)[9]		Mascot
1516.7617	1516.7148	-0.0469	-31	12	23	DRFLFCAEAIFK			Carbamidomethyl (C)[6]		Mascot
1546.736	1546.7124	-0.0236	-15	247	259	WSPELAAACEVWK			Carbamidomethyl (C)[9]		Mascot
1546.736	1546.7124	-0.0236	-15	247	259	WSPELAAACEVWK	91	100	Carbamidomethyl (C)[9]		Mascot
1572.7911	1572.7014	-0.0897	-57	218	231	VALEACVQARNEGR			Carbamidomethyl (C)[6]		Mascot

53

ribulose 1,5-bisphosphate carboxylase/oxygenase large subunit (plastid) [Cucumis hystrix]	gi|590000423	52608.4	6	14	309	100	258	100

Protein Group

RecName: Full=Ribulose bisphosphate carboxylase large chain; Short=RuBisCO large subunit; Flags: Precursor	gi|158518394	52608.4	6
ribulose 1,5-bisphosphate carboxylase/oxygenase large	gi|586598694	52608.4	6

subunit (plastid) [Cucumis hystrix]			
ribulose-1,5-bisphosphate carboxylase/oxygenase large subunit (chloroplast) [Cucumis sativus]	gi|115498311	52608.4	6
ribulose-1,5-bisphosphate carboxylase/oxygenase large subunit (chloroplast) [Cucumis sativus]	gi|115432812	52608.4	6

Peptide Information

Calc. Mass	Obsrv. Mass	± da	± ppm	Start Seq.	End Seq.	Sequence	Ion Score	C. I. %	Modification	Rank	Result Type
830.4366	830.4335	-0.0031	-4	440	446	EGNEIIR					Mascot
914.405	914.3935	-0.0115	-13	306	312	NHGMHFR			Oxidation (M)[4]		Mascot
1023.4741	1023.4537	-0.0204	-20	351	358	DDFVEKDR					Mascot
1059.5615	1059.5474	-0.0141	-13	422	431	VALEACVQAR					Mascot
1116.583	1116.571	-0.012	-11	422	431	VALEACVQAR			Carbamidomethyl (C)[6]		Mascot
1116.583	1116.571	-0.012	-11	422	431	VALEACVQAR	59	99.97	Carbamidomethyl (C)[6]		Mascot
1275.7307	1275.7186	-0.0121	-9	340	350	EITLGFVDLLR					Mascot
1275.7307	1275.7186	-0.0121	-9	340	350	EITLGFVDLLR	114	100			Mascot
1465.7217	1465.7002	-0.0215	-15	320	334	MSGGDHIHAGTVVGK					Mascot
1481.7167	1481.6875	-0.0292	-20	320	334	MSGGDHIHAGTVVGK			Oxidation (M)[1]		Mascot
1481.7167	1481.6875	-0.0292	-20	320	334	MSGGDHIHAGTVVGK	17	0	Oxidation (M)[1]		Mascot
1488.6927	1488.6635	-0.0292	-20	464	475	EIKFEFEAMDTL			Oxidation (M)[9]		Mascot

1510.7571	1510.6758	-0.0813	-54	1	14	MSPQTETKASVGFK					Mascot
1516.7617	1516.7463	-0.0154	-10	216	227	DRFLFCAEAIFK			Carbamidomethyl (C)[6]		Mascot
1546.736	1546.7113	-0.0247	-16	451	463	WSPELAAACEVWK			Carbamidomethyl (C)[9]		Mascot
1546.736	1546.7113	-0.0247	-16	451	463	WSPELAAACEVWK	68	99.997	Carbamidomethyl (C)[9]		Mascot
1572.7911	1572.7007	-0.0904	-57	422	435	VALEACVQARNEGR			Carbamidomethyl (C)[6]		Mascot
1708.8766	1708.8367	-0.0399	-23	147	161	TFQGPPHGIQVERDK					Mascot
2009.0591	2009.0312	-0.0279	-14	340	356	EITLGFVDLLRDDFVEK					Mascot
2066.0085	2065.9717	-0.0368	-18	320	339	MSGGDHIHAGTVVGKLEGER			Oxidation (M)[1]		Mascot

54	PREDICTED: LOW QUALITY PROTEIN: ribulose bisphosphate carboxylase large chain, partial [Cucumis	gi|659133948	30149	6.23	11	224	100	176	100

melo]

Peptide Information

Calc. Mass	Obsrv. Mass	± da	± ppm	Start Seq.	End Seq.	Sequence	Ion Score	C. I. %	Modification	Rank	Result Type
830.4366	830.4388	0.0022	3	236	242	EGNEIIR					Mascot
914.405	914.3876	-0.0174	-19	102	108	NHGMHFR			Oxidation (M)[4]		Mascot
928.4669	928.431	-0.0359	-39	92	99	AMHAVIDR			Oxidation (M)[2]		Mascot
1116.583	1116.5742	-0.0088	-8	218	227	VALEACVQAR			Carbamidomethyl (C)[6]		Mascot
1116.583	1116.5742	-0.0088	-8	218	227	VALEACVQAR	14	0	Carbamidomethyl (C)[6]		Mascot
1118.4711	1118.4564	-0.0147	-13	263	271	FEFEAMDTI			Oxidation (M)[6]		Mascot
1154.5636	1154.4846	-0.079	-68	100	108	QKNHGMHFR					Mascot
1275.7307	1275.7185	-0.0122	-10	136	146	EITLGFVDLLR					Mascot
1275.7307	1275.7185	-0.0122	-10	136	146	EITLGFVDLLR	103	100			Mascot
1459.7402	1459.6874	-0.0528	-36	12	23	DRFLFCAEAIFK					Mascot
1465.7217	1465.6548	-0.0669	-46	116	130	MSGGDHIHAGTVVGK					Mascot
1472.6978	1472.6747	-0.0231	-16	260	271	EIKFEFEAMDTI					Mascot
1481.7167	1481.6863	-0.0304	-21	116	130	MSGGDHIHAGTVVGK			Oxidation (M)[1]		Mascot
1481.7167	1481.6863	-0.0304	-21	116	130	MSGGDHIHAGTVVGK	59	99.969	Oxidation (M)[1]		Mascot
1488.6927	1488.6545	-0.0382	-26	260	271	EIKFEFEAMDTI			Oxidation (M)[9]		Mascot
1546.736	1546.7081	-0.0279	-18	247	259	WSPELAAACEVWK			Carbamidomethyl (C)[9]		Mascot

55	ribulose-1,5-bisphosphate carboxylase/oxygenase large subunit, partial (chloroplast) [Cucumis oreosyce]	gi|111182716	52195.2	6	10	170	100	145	100

Peptide Information

Calc. Mass	Obsrv. Mass	± da	± ppm	Start Seq.	End Seq.	Sequence	Ion Score	C. I. %	Modification	Rank	Result Type
1021.5312	1021.5263	-0.0049	-5	29	37	DTDILAAFR					Mascot
1021.5312	1021.5263	-0.0049	-5	29	37	DTDILAAFR	73	99.999			Mascot
1037.4899	1037.5123	0.0224	22	347	354	DDFIEKDR					Mascot
1059.5615	1059.4625	-0.099	-93	418	427	VALEACVQAR					Mascot
1067.5732	1067.5084	-0.0648	-61	1	10	TETKASVGFK					Mascot
1168.6256	1168.6116	-0.014	-12	292	301	AMHAVIDRQK					Mascot
1407.6678	1407.6484	-0.0194	-14	18	28	LTYYTPEYETK					Mascot
1407.6678	1407.6484	-0.0194	-14	18	28	LTYYTPEYETK	72	99.999			Mascot

Calc. Mass	Obsrv. Mass	± da	± ppm	Start Seq.	End Seq.	Sequence	Ion Score	C. I. %	Modification	Rank	Result Type
1417.8049	1417.6761	-0.1288	-91	131	142	LEDLRIPTAYVK					Mascot
1451.6219	1451.65	0.0281	19	198	209	DDENVNSQPFMR					Mascot
1472.6978	1472.645	-0.0528	-36	460	471	EIKFEFEAMDTL					Mascot
2410.1814	2410.1687	-0.0127	-5	18	37	LTYYTPEYETKDTDILAAFR					Mascot

56

Plastocyanin A, chloroplast [Cucumis sativus]	gi|700204935	17010.6	4.92	4	260	100	242	100

Protein Group

PREDICTED: plastocyanin [Cucumis sativus]	gi|449437172	17010.6	4.92000007629395

Peptide Information

Calc. Mass	Obsrv. Mass	± da	± ppm	Start Seq.	End Seq.	Sequence	Ion Score	C. I. %	Modification	Rank	Result Type
1932.8368	1932.8446	0.0078	4	145	162	GSYSFYCSPHQGAGMVGK			Carbamidomethyl (C)[7]		Mascot
1948.8317	1948.8159	-0.0158	-8	145	162	GSYSFYCSPHQGAGMVGK			Carbamidomethyl (C)[7], Oxidation (M)[15]		Mascot
1948.8317	1948.8159	-0.0158	-8	145	162	GSYSFYCSPHQGAGMVGK	71	99.999	Carbamidomethyl (C)[7], Oxidation (M)[15]		Mascot
2305.0376	2305.0903	0.0527	23	145	166	GSYSFYCSPHQGAGMVGKVTVN			Oxidation (M)[15]		Mascot
2556.2002	2556.2004	0.0002	0	98	121	NNAGFPHNVVFDEDEIPSGVDVGK					Mascot
2556.2002	2556.2004	0.0002	0	98	121	NNAGFPHNVVFDEDEIPSGVDVGK	171	100			Mascot
2621.2651	2621.2334	-0.0317	-12	122	144	ISMDEENLLNAPGEVYEVQLTEK					Mascot

57

Malate dehydrogenase, mitochondrial [Cucumis sativus]	gi|700198438	36180.1	8.52	13	478	100	416	100

Protein Group

PREDICTED: malate dehydrogenase, mitochondrial [Cucumis sativus]	gi|449438883	36180.1	8.5200004577

6367

Peptide Information

Calc. Mass	Obsrv. Mass	± da	± ppm	Start Seq.	End Seq.	Sequence	Ion Score	C. I. %	Modification	Rank	Result Type
1219.7046	1219.7153	0.0107	9	176	186	LFGVTTLDVVR					Mascot
1219.7046	1219.7153	0.0107	9	176	186	LFGVTTLDVVR	59	99.973			Mascot
1258.7075	1258.7091	0.0016	1	329	339	LMPELKASIEK					Mascot
1318.7002	1318.7072	0.007	5	121	132	DDLFNINAGIVK					Mascot
1318.7002	1318.7072	0.007	5	121	132	DDLFNINAGIVK	18	0			Mascot
1347.7996	1347.8098	0.0102	8	175	186	KLFGVTTLDVVR					Mascot
1347.7996	1347.8098	0.0102	8	175	186	KLFGVTTLDVVR	22	0			Mascot
1543.6945	1543.6914	-0.0031	-2	85	98	SEVTGYMGEEQLGK			Oxidation (M)[7]		Mascot
1592.9006	1592.9141	0.0135	8	99	114	ALEGSDVVIIPAGVPR					Mascot
1592.9006	1592.9141	0.0135	8	99	114	ALEGSDVVIIPAGVPR	148	100			Mascot
1601.8354	1601.8765	0.0411	26	11	25	SAVSRSSSSNHLLTR					Mascot
1795.051	1795.026	-0.025	-14	37	55	VAVLGAAGGIGQPLALLM K			Oxidation (M)[18]		Mascot
1818.912	1818.9042	-0.0078	-4	307	323	NGVESVLDLGPLSDFEK					Mascot
1923.146	1923.1169	-0.0291	-15	36	55	KVAVLGAAGGIGQPLALL MK			Oxidation (M)[19]		Mascot
2210.1565	2210.1763	0.0198	9	63	84	LALYDIGATPGVAADVGH VNTR					Mascot
2210.1565	2210.1763	0.0198	9	63	84	LALYDIGATPGVAADVGH VNTR	168	100			Mascot
2274.1257	2274.072	-0.0537	-24	252	274	AGKGSATLSMAYAGALF ADACLK			Carbamidomethyl (C)[21]		Mascot
2290.1206	2290.1763	0.0557	24	252	274	AGKGSATLSMAYAGALF ADACLK			Carbamidomethyl (C)[21], Oxidation (M)[10]		Mascot
2927.4497	2927.4583	0.0086	3	275	301	GLNGVPDVVECSFVQST VTELPFFASK			Carbamidomethyl (C)[11]		Mascot

58

hypothetical protein Csa_2G252020 [Cucumis sativus]	gi|700206734	42867.2	6.38	13	254	100	200	100

Protein Group

PREDICTED: fructose-bisphosphate aldolase 1,	gi|449464838	42867.2	6.3800

chloroplastic [Cucumis sativus] 001144 4092

Peptide Information

Calc. Mass	Obsrv. Mass	± da	± ppm	Start Seq.	End Seq.	Sequence	Ion Score	C. I. %	Modification	Rank	Result Type
871.4996	871.4576	-0.042	-48	26	33	QPSVSVVR					Mascot
873.4577	873.4739	0.0162	19	202	209	EAAWGLAR					Mascot
1027.5895	1027.5959	0.0064	6	357	365	IAQDALIQR					Mascot
1027.5895	1027.5959	0.0064	6	357	365	IAQDALIQR	20	0			Mascot
1098.5327	1098.549	0.0163	15	172	181	AAAYYQQGAR					Mascot
1098.5327	1098.549	0.0163	15	172	181	AAAYYQQGAR	69	99.997			Mascot
1387.7175	1387.7301	0.0126	9	82	94	LASIGLENTEANR					Mascot
1452.8422	1452.838	-0.0042	-3	187	201	TVVSIPNGPSALAVK					Mascot
1543.8187	1543.8274	0.0087	6	81	94	RLASIGLENTEANR					Mascot
1620.8843	1620.8793	-0.005	-3	280	294	ASPQTVAEYTLSLLK					Mascot
1864.0062	1863.9905	-0.0157	-8	278	294	DKASPQTVAEYTLSLLK					Mascot
2363.1653	2363.1663	0.001	0	110	130	YISGAILFEETLYQSTVDGEK					Mascot
2427.1721	2427.1995	0.0274	11	149	171	GLVPLPGSNNESWCQGLDGLASR			Carbamidomethyl (C)[14]		Mascot
2427.1721	2427.1995	0.0274	11	149	171	GLVPLPGSNNESWCQGLDGLASR	111	100	Carbamidomethyl (C)[14]		Mascot
2442.2446	2442.1682	-0.0764	-31	34	56	CHPTAAPSTLIVRAGSYNDELVK					Mascot
3019.5371	3019.5813	0.0442	15	210	237	YAAVSQDNGLVPIVEPEILLDGEHGIDR					Mascot

59

hypothetical protein Csa_2G252020 [Cucumis sativus]	gi|700206734	42867.2	6.38	19	340	100	234	100

Protein Group

PREDICTED: fructose-bisphosphate aldolase 1, chloroplastic [Cucumis sativus] gi|449464838 42867.2 6.3800 001144 4092

Peptide Information

Calc. Mass	Obsrv. Mass	± da	± ppm	Start Seq.	End Seq.	Sequence	Ion Score	C. I. %	Modification	Rank	Result Type
822.4355	822.4471	0.0116	14	238	244	TFEVAQK					Mascot
871.4996	871.4641	-0.0355	-41	26	33	QPSVSVVR					Mascot
873.4577	873.4812	0.0235	27	202	209	EAAWGLAR					Mascot
947.4979	947.5099	0.012	13	339	346	ALQNTCLK			Carbamidomethyl (C)[6]		Mascot
1027.5895	1027.6063	0.0168	16	357	365	IAQDALIQR					Mascot
1027.5895	1027.6063	0.0168	16	357	365	IAQDALIQR	55	99.925			Mascot
1098.5327	1098.5555	0.0228	21	172	181	AAAYYQQGAR					Mascot
1098.5327	1098.5555	0.0228	21	172	181	AAAYYQQGAR	49	99.746			Mascot
1116.6372	1116.5741	-0.0631	-57	366	376	AKSNSLAQLGK					Mascot
1387.7175	1387.7427	0.0252	18	82	94	LASIGLENTEANR					Mascot
1387.7175	1387.7427	0.0252	18	82	94	LASIGLENTEANR	55	99.928			Mascot
1395.7114	1395.7855	0.0741	53	47	59	AGSYNDELVKTAK					Mascot
1452.8422	1452.845	0.0028	2	187	201	TVVSIPNGPSALAVK					Mascot
1466.6614	1466.7114	0.05	34	67	80	GILAMDESNATCGK			Carbamidomethyl (C)[12]		Mascot
1482.6564	1482.6515	-0.0049	-3	67	80	GILAMDESNATCGK			Carbamidomethyl (C)[12], Oxidation (M)[5]		Mascot
1543.8187	1543.8396	0.0209	14	81	94	RLASIGLENTEANR					Mascot
1620.8843	1620.9001	0.0158	10	280	294	ASPQTVAEYTLSLLK					Mascot
1864.0062	1864.0201	0.0139	7	278	294	DKASPQTVAEYTLSLLK					Mascot
2363.1653	2363.1926	0.0273	12	110	130	YISGAILFEETLYQSTVDGEK					Mascot
2370.1506	2370.1514	0.0008	0	149	171	GLVPLPGSNNESWCQGLDGLASR					Mascot
2427.1721	2427.2256	0.0535	22	149	171	GLVPLPGSNNESWCQGLDGLASR			Carbamidomethyl (C)[14]		Mascot
2427.1721	2427.2256	0.0535	22	149	171	GLVPLPGSNNESWCQGLDGLASR	76	100	Carbamidomethyl (C)[14]		Mascot

2434.0027	2434.1807	0.178	73	377	397	YTGEGESEEANQDMYVKGYTY				Mascot
2442.2446	2442.2161	-0.0285	-12	34	56	CHPTAAPSTLIVRAGSYNDELVK				Mascot
2449.9976	2450.168	0.1704	70	377	397	YTGEGESEEANQDMYVKGYTY			Oxidation (M)[14]	Mascot

2499.2661	2499.0991	-0.167	-67	34	56	CHPTAAPSTLIVRAGSYN DELVK			Carbamidomethyl (C)[1]		Mascot
3019.5371	3019.6099	0.0728	24	210	237	YAAVSQDNGLVPIVEPEIL LDGEHGIDR					Mascot

60

Beta-form rubisco activase [Cucumis sativus]	gi|700195391	48292.6	8.19	18	568	100	481	100

Protein Group

PREDICTED: ribulose bisphosphate carboxylase/oxygenase activase 2, chloroplastic-like [Cucumis sativus]	gi|449459892	48292.6	8.1899 995803 833

Peptide Information

Calc. Mass	Obsrv. Mass	± da	± ppm	Start Seq.	End Seq.	Sequence	Ion Score	C. I. %	Modification	Rank	Result Type
895.4156	895.4451	0.0295	33	360	366	VYDDEVR					Mascot
940.4675	940.4932	0.0257	27	311	317	FYWAPTR					Mascot
940.4675	940.4932	0.0257	27	311	317	FYWAPTR	26	43.532			Mascot
1023.5105	1023.5338	0.0233	23	360	367	VYDDEVRK					Mascot
1228.6031	1228.6311	0.028	23	180	189	SFQCELVFAK			Carbamidomethyl (C)[4]		Mascot
1639.7567	1639.7949	0.0382	23	230	244	MSCLFINDLDAGAGR			Carbamidomethyl (C)[3]		Mascot
1655.7517	1655.7866	0.0349	21	230	244	MSCLFINDLDAGAGR			Carbamidomethyl (C)[3], Oxidation (M)[1]		Mascot
1655.7517	1655.7866	0.0349	21	230	244	MSCLFINDLDAGAGR	16	0	Carbamidomethyl (C)[3], Oxidation (M)[1]		Mascot
1773.8728	1773.895	0.0222	13	386	400	EPPPKFEQPTMSLEK			Oxidation (M)[11]		Mascot
1801.9919	1801.9534	-0.0385	-21	40	56	VVNSRVVNPKPSSGSFK					Mascot
1822.9844	1822.8997	-0.0847	-46	1	19	MAASAASVGVVNHAPLS LK					Mascot
1838.9794	1838.97	-0.0094	-5	1	19	MAASAASVGVVNHAPLS LK			Oxidation (M)[1]		Mascot
1865.9314	1865.9716	0.0402	22	401	416	LLEYGGMLVQEQENVK			Oxidation (M)[7]		Mascot
1882.9698	1883.0193	0.0495	26	341	357	LVDTFPGQSIDFFGALR					Mascot
1882.9698	1883.0193	0.0495	26	341	357	LVDTFPGQSIDFFGALR	121	100			Mascot
1890.8828	1890.9282	0.0454	24	78	94	WAGLGTDISDDQQDITR					Mascot
2006.0376	2006.0806	0.043	21	401	417	LLEYGGMLVQEQENVKR					Mascot
2022.0325	2022.0636	0.0311	15	401	417	LLEYGGMLVQEQENVKR			Oxidation (M)[7]		Mascot
2022.0325	2022.0636	0.0311	15	401	417	LLEYGGMLVQEQENVKR	18	0	Oxidation (M)[7]		Mascot

2089.1692	2089.2251	0.0559	27	286	304	VPIIVTGNDFSTLYAPLIR					Mascot
2089.1692	2089.2251	0.0559	27	286	304	VPIIVTGNDFSTLYAPLIR	160	100			Mascot
2309.0464	2309.0735	0.0271	12	128	148	SYDGMDNVLGGLYIAPAF MDK			Oxidation (M)[5,19]		Mascot

2437.1414	2437.1621	0.0207	8	127	148	KSYDGMDNVLGGLYIAP AFMDK			Oxidation (M)[6,20]		Mascot
2476.2314	2476.2988	0.0674	27	418	440	VQLAETYLNEAALGNANE DAITR					Mascot
2476.2314	2476.2988	0.0674	27	418	440	VQLAETYLNEAALGNANE DAITR	139	100			Mascot
2632.3325	2632.4094	0.0769	29	417	440	RVQLAETYLNEAALGNA NEDAITR					Mascot
3342.4866	3342.571	0.0844	25	97	126	GMVDSVFQAPMQAGTH YAVMSSYEYLSQGR			Oxidation (M)[2,11]		Mascot
3358.4814	3358.5923	0.1109	33	97	126	GMVDSVFQAPMQAGTH YAVMSSYEYLSQGR			Oxidation (M)[2,11,20]		Mascot

61

PREDICTED: S-adenosylmethionine synthase 2 [Cucumis sativus]	gi|449472806	43196.7	5.35		24	764	100	605	100

Protein Group

PREDICTED: S-adenosylmethionine synthase 2 [Cucumis sativus]	gi|778728392	43196.7	5.3499 999046 3257
hypothetical protein Csa_7G419610 [Cucumis sativus]	gi|700189832	43196.7	5.3499 999046 3257
hypothetical protein Csa_7G419650 [Cucumis sativus]	gi|700189836	43196.7	5.3499 999046 3257

Peptide Information

Calc. Mass	Obsrv. Mass	± da	± ppm	Start Seq.	End Seq.	Sequence	Ion Score	C. I. %	Modification	Rank	Result Type

873.5152	873.5496	0.0344	39	292	300	SIVASGLAR				Mascot	
919.4778	919.5509	0.0731	80	68	74	IVRDTCR			Carbamidomethyl (C)[6]	Mascot	
979.4744	979.5043	0.0299	31	365	373	TAAYGHFGR				Mascot	
979.4744	979.5043	0.0299	31	365	373	TAAYGHFGR	68	99.997		Mascot	
1141.6113	1141.6421	0.0308	27	228	237	TIFHLNPSGR				Mascot	
1141.6113	1141.6421	0.0308	27	228	237	TIFHLNPSGR	63	99.988		Mascot	
1163.6532	1163.6078	-0.0454	-39	281	291	SGAYIVRQAAK				Mascot	
1296.7773	1296.8051	0.0278	21	328	338	IPDKEILEIVK				Mascot	
1302.7781	1302.7947	0.0166	13	212	222	EHVIKPIIPEK				Mascot	
1400.674	1400.7146	0.0406	29	158	169	NGTCPWLRPDGK			Carbamidomethyl (C)[4]	Mascot	
1453.7548	1453.7942	0.0394	27	238	252	FVIGGPHGDAGLTGR				Mascot	
1453.7548	1453.7942	0.0394	27	238	252	FVIGGPHGDAGLTGR	115	100		Mascot	
1471.7474	1471.7659	0.0185	13	157	169	KNGTCPWLRPDGK				Mascot	
1486.728	1486.7524	0.0244	16	48	60	TNMVMVFGEITTK			Oxidation (M)[3]	Mascot	
1502.723	1502.7617	0.0387	26	48	60	TNMVMVFGEITTK			Oxidation (M)[3,5]	Mascot	
1528.7689	1528.7963	0.0274	18	157	169	KNGTCPWLRPDGK			Carbamidomethyl (C)[5]	Mascot	
**Project 1\Sample project201(**	1528.7963									**397**	Mascot
1581.8496	1581.8647	0.0151	10	238	253	FVIGGPHGDAGLTGRK				Mascot	
1789.9232	1789.9152	-0.008	-4	223	237	YLDEKTIFHLNPSGR				Mascot	
1852.8383	1852.8777	0.0394	21	75	91	NIGFISDDVGLDADNCK			Carbamidomethyl (C)[16]	Mascot	
1852.8383	1852.8777	0.0394	21	75	91	NIGFISDDVGLDADNCK	160	100	Carbamidomethyl (C)[16]	Mascot	
1963.9662	1964.0179	0.0517	26	254	273	IIIDTYGGWGAHGGGAFSGK				Mascot	
2023.0317	2023.0768	0.0451	22	339	355	ENFDFRPGMITINLDLK				Mascot	
2039.0266	2039.0629	0.0363	18	339	355	ENFDFRPGMITINLDLK			Oxidation (M)[9]	Mascot	
2055.9805	2056.0359	0.0554	27	170	187	TQVTVEYYNDNGAMVPVR				Mascot	
2071.9753	2072.0281	0.0528	25	170	187	TQVTVEYYNDNGAMVPVR			Oxidation (M)[14]	Mascot	
2092.061	2092.0745	0.0135	6	253	273	KIIIDTYGGWGAHGGGAFSGK				Mascot	
2334.0588	2334.1108	0.052	22	19	39	LCDQISDAVLDACLAQDPDSK			Carbamidomethyl (C)[2,13]	Mascot	
2417.2573	2417.3259	0.0686	28	92	113	VLVNIEQQSPDIAQGVHGHFTK				Mascot	

2417.2573	2417.3259	0.0686	28	92	113	VLVNIEQQSPDIAQGVHG HFTK	139	100			Mascot
2438.1072	2438.259	0.1518	62	40	60	VACETCSKTNMVMVFGEI TTK			Carbamidomethyl (C)[3,6], Oxidation (M)[11,13]		Mascot
2461.1558	2461.2261	0.0703	29	374	393	DDPDFTWETIKPLEWEKP QS					Mascot
2679.3472	2679.4216	0.0744	28	188	211	VHTVLISTQHDETVTNDEI ATDLK					Mascot
2679.3472	2679.4216	0.0744	28	188	211	VHTVLISTQHDETVTNDEI ATDLK	59	99.968			Mascot
3713.7576	3713.9131	0.1555	42	114	147	RPEEIGAGDQGHMFGYA TDETPELMPLSHVLATK			Oxidation (M)[13]		Mascot

62

ribulose-1,5-bisphosphate carboxylase/oxygenase large subunit, partial (chloroplast) [Cucumis hystrix]	gi|111182702	51456.9	6	25	497	100	336	100

Peptide Information

Calc. Mass	Obsrv. Mass	± da	± ppm	Start Seq.	End Seq.	Sequence	Ion Score	C. I. %	Modification	Rank	Result Type
805.4818	805.5026	0.0208	26	129	135	IPTAYIK					Mascot
830.4366	830.4665	0.0299	36	429	435	EGNEIIR					Mascot
898.41	898.467	0.057	63	295	301	NHGMHFR					Mascot
910.4451	910.4766	0.0315	35	177	183	AVYECLR			Carbamidomethyl (C)[5]		Mascot
914.405	914.4319	0.0269	29	295	301	NHGMHFR			Oxidation (M)[4]		Mascot
914.405	914.4319	0.0269	29	295	301	NHGMHFR	5	0	Oxidation (M)[4]		Mascot
928.4669	928.4924	0.0255	27	285	292	AMHAVIDR			Oxidation (M)[2]		Mascot
962.4789	962.5056	0.0267	28	217	225	SQAETGEIK					Mascot
1021.5312	1021.5573	0.0261	26	22	30	DTDILAAFR					Mascot
1023.4741	1023.5096	0.0355	35	340	347	DDFVEKDR					Mascot
1116.583	1116.621	0.038	34	411	420	VALEACVQAR			Carbamidomethyl (C)[6]		Mascot
1116.583	1116.621	0.038	34	411	420	VALEACVQAR	13	0	Carbamidomethyl (C)[6]		Mascot
1154.5636	1154.5815	0.0179	16	293	301	QKNHGMHFR					Mascot
1170.5586	1170.6721	0.1135	97	293	301	QKNHGMHFR			Oxidation (M)[6]		Mascot
1170.5586	1170.6721	0.1135	97	293	301	QKNHGMHFR			Oxidation (M)[6]		Mascot
1187.6644	1187.6991	0.0347	29	275	284	DNGLLLHIHR					Mascot

1187.6644	1187.6991	0.0347	29	275	284	DNGLLLHIHR	71	99.998		Mascot
1188.6122	1188.6937	0.0815	69	207	216	FLFCAEALFK				Mascot
1245.6433	1245.6847	0.0414	33	429	439	EGNEIIREASK				Mascot
1275.7307	1275.7677	0.037	29	329	339	EITLGFVDLLR				Mascot
1275.7307	1275.7677	0.037	29	329	339	EITLGFVDLLR	77	100		Mascot
1407.6678	1407.7125	0.0447	32	11	21	LTYYTPEYETK				Mascot
1445.8297	1445.818	-0.0117	-8	154	166	YGRPLLGCTIKPK				Mascot
1451.6219	1451.6726	0.0507	35	191	202	DDENVNSQPFMR				Mascot
1465.7546	1465.7966	0.042	29	136	148	TFQGPPHGIQVER				Mascot
1465.7546	1465.7966	0.042	29	136	148	TFQGPPHGIQVER	99	100		Mascot

1481.7167	1481.7809	0.0642	43	309	323	MSGGDHIHAGTVVGK			Oxidation (M)[1]	Mascot
1489.7145	1489.7439	0.0294	20	440	452	WSPELAAACEVWK				Mascot
1502.8512	1502.8892	0.038	25	154	166	YGRPLLGCTIKPK			Carbamidomethyl (C)[8]	Mascot
1502.8512	1502.8892	0.038	25	154	166	YGRPLLGCTIKPK	22	0	Carbamidomethyl (C)[8]	Mascot
1516.7617	1516.879	0.1173	77	205	216	DRFLFCAEALFK			Carbamidomethyl (C)[6]	Mascot
1546.736	1546.7755	0.0395	26	440	452	WSPELAAACEVWK			Carbamidomethyl (C)[9]	Mascot
1844.7612	1844.788	0.0268	15	226	241	GHYLNATAGTCEEMMK			Carbamidomethyl (C)[11], Oxidation (M)[14,15]	Mascot
2000.8623	2000.8981	0.0358	18	226	242	GHYLNATAGTCEEMMKR			Carbamidomethyl (C)[11], Oxidation (M)[14,15]	Mascot
2169.9871	2170.042	0.0549	25	184	202	GGLDFTKDDENVNSQPFMR				Mascot
2185.9819	2186.0325	0.0506	23	184	202	GGLDFTKDDENVNSQPFMR			Oxidation (M)[18]	Mascot
2185.9819	2186.0325	0.0506	23	184	202	GGLDFTKDDENVNSQPFMR	69	99.997	Oxidation (M)[18]	Mascot
3010.4187	3010.5247	0.106	35	248	274	ELGAPIVMHDYLTGGFTANTSLAHYCR			Carbamidomethyl (C)[26], Oxidation (M)[8]	Mascot

63	ribulose-1,5-bisphosphate carboxylase/oxygenase large subunit, partial (chloroplast) [Cucumis hystrix]	gi|111182702	51456.9	6	22	753	100	631	100

Peptide Information

Calc. Mass	Obsrv. Mass	± da	± ppm	Start Seq.	End Seq.	Sequence	Ion Score	C. I. %	Modification	Rank	Result Type
898.41	898.4479	0.0379	42	295	301	NHGMHFR					Mascot
910.4451	910.4821	0.037	41	177	183	AVYECLR			Carbamidomethyl (C)[5]		Mascot
910.4451	910.4821	0.037	41	177	183	AVYECLR	18	0	Carbamidomethyl (C)[5]		Mascot
912.472	912.4963	0.0243	27	285	292	AMHAVIDR					Mascot
914.405	914.4421	0.0371	41	295	301	NHGMHFR			Oxidation (M)[4]		Mascot
928.4669	928.5025	0.0356	38	285	292	AMHAVIDR			Oxidation (M)[2]		Mascot
962.4789	962.5209	0.042	44	217	225	SQAETGEIK					Mascot
1021.5312	1021.5706	0.0394	39	22	30	DTDILAAFR					Mascot
1021.5312	1021.5706	0.0394	39	22	30	DTDILAAFR	90	100			Mascot
1154.5636	1154.6177	0.0541	47	293	301	QKNHGMHFR					Mascot
1187.6644	1187.7162	0.0518	44	275	284	DNGLLLHIHR					Mascot
1188.6122	1188.7025	0.0903	76	207	216	FLFCAEALFK					Mascot
1245.6433	1245.6742	0.0309	25	429	439	EGNEIIREASK					Mascot
1275.7307	1275.7754	0.0447	35	329	339	EITLGFVDLLR					Mascot
1343.6525	1343.7534	0.1009	75	173	183	NYGRAVYECLR					Mascot
1407.6678	1407.7159	0.0481	34	11	21	LTYYTPEYETK					Mascot
1445.8297	1445.826	-0.0037	-3	154	166	YGRPLLGCTIKPK					Mascot
1451.6219	1451.6786	0.0567	39	191	202	DDENVNSQPFMR					Mascot
1465.7546	1465.811	0.0564	38	136	148	TFQGPPHGIQVER					Mascot
1465.7546	1465.811	0.0564	38	136	148	TFQGPPHGIQVER	115	100			Mascot
1502.8512	1502.9044	0.0532	35	154	166	YGRPLLGCTIKPK			Carbamidomethyl (C)[8]		Mascot
1502.8512	1502.9044	0.0532	35	154	166	YGRPLLGCTIKPK	11	0	Carbamidomethyl (C)[8]		Mascot
1812.7714	1812.8298	0.0584	32	226	241	GHYLNATAGTCEEMMK			Carbamidomethyl (C)[11]		Mascot
1828.7662	1828.8209	0.0547	30	226	241	GHYLNATAGTCEEMMK			Carbamidomethyl (C)[11], Oxidation (M)[14]		Mascot
1844.7612	1844.8287	0.0675	37	226	241	GHYLNATAGTCEEMMK			Carbamidomethyl (C)[11], Oxidation (M)[14,15]		Mascot
1904.9211	1904.9332	0.0121	6	436	452	EASKWSPELAAACEVWK					Mascot

1968.8724	1968.991	0.1186	60	226	242	GHYLNATAGTCEEMMKR			Carbamidomethyl (C)[11]		Mascot
1984.8674	1984.9446	0.0772	39	226	242	GHYLNATAGTCEEMMKR			Carbamidomethyl (C)[11], Oxidation (M)[14]		Mascot
2000.8623	2000.92	0.0577	29	226	242	GHYLNATAGTCEEMMKR			Carbamidomethyl (C)[11], Oxidation (M)[14,15]		Mascot
2169.9871	2170.0691	0.082	38	184	202	GGLDFTKDDENVNSQPF					Mascot

Calc. Mass	Obsrv. Mass	± da	± ppm	Start Seq.	End Seq.	Sequence	Ion Score	C. I. %	Modification	Rank	Result Type
						MR					
2169.9871	2170.0691	0.082	38	184	202	GGLDFTKDDENVNSQPFMR	105	100			Mascot
2185.9819	2186.0569	0.075	34	184	202	GGLDFTKDDENVNSQPFMR			Oxidation (M)[18]		Mascot
2410.1814	2410.2683	0.0869	36	11	30	LTYYTPEYETKDTDILAAFR					Mascot
2994.4238	2994.5618	0.138	46	248	274	ELGAPIVMHDYLTGGFTANTSLAHYCR			Carbamidomethyl (C)[26]		Mascot
2994.4238	2994.5618	0.138	46	248	274	ELGAPIVMHDYLTGGFTANTSLAHYCR	111	100	Carbamidomethyl (C)[26]		Mascot
3010.4187	3010.5537	0.135	45	248	274	ELGAPIVMHDYLTGGFTANTSLAHYCR			Carbamidomethyl (C)[26], Oxidation (M)[8]		Mascot
3010.4187	3010.5537	0.135	45	248	274	ELGAPIVMHDYLTGGFTANTSLAHYCR	45	99.369	Carbamidomethyl (C)[26], Oxidation (M)[8]		Mascot
3854.8721	3855.0991	0.227	59	31	68	VTPQPGVPPEEAGAAVAAESSTGTWTTVWTDGLTSLDR					Mascot
3854.8721	3855.0991	0.227	59	31	68	VTPQPGVPPEEAGAAVAAESSTGTWTTVWTDGLTSLDR	182	100			Mascot

64

ribulose-1,5-bisphosphate carboxylase/oxygenase large subunit, partial (chloroplast) [Cucumis hystrix]	gi|111182702	51456.9	6	22	676	100	559	100

Peptide Information

Calc. Mass	Obsrv. Mass	± da	± ppm	Start Seq.	End Seq.	Sequence	Ion Score	C. I. %	Modification	Rank	Result Type
830.4366	830.4578	0.0212	26	429	435	EGNEIIR					Mascot
853.4236	853.4368	0.0132	15	177	183	AVYECLR					Mascot
898.41	898.425	0.015	17	295	301	NHGMHFR					Mascot
910.4451	910.4624	0.0173	19	177	183	AVYECLR			Carbamidomethyl (C)[5]		Mascot
910.4451	910.4624	0.0173	19	177	183	AVYECLR	37	94.759	Carbamidomethyl (C)[5]		Mascot
914.405	914.4193	0.0143	16	295	301	NHGMHFR			Oxidation (M)[4]		Mascot
928.4669	928.4823	0.0154	17	285	292	AMHAVIDR			Oxidation (M)[2]		Mascot

962.4789	962.4952	0.0163	17	217	225	SQAETGEIK				Mascot
1023.4741	1023.4888	0.0147	14	340	347	DDFVEKDR				Mascot
1116.583	1116.6035	0.0205	18	411	420	VALEACVQAR			Carbamidomethyl (C)[6]	Mascot
1116.583	1116.6035	0.0205	18	411	420	VALEACVQAR	40	97.337	Carbamidomethyl (C)[6]	Mascot
1154.5636	1154.5768	0.0132	11	293	301	QKNHGMHFR				Mascot
1170.5586	1170.6598	0.1012	86	293	301	QKNHGMHFR			Oxidation (M)[6]	Mascot
1170.5586	1170.6598	0.1012	86	293	301	QKNHGMHFR			Oxidation (M)[6]	Mascot
1187.6644	1187.6851	0.0207	17	275	284	DNGLLLHIHR				Mascot
1187.6644	1187.6851	0.0207	17	275	284	DNGLLLHIHR	63	99.988		Mascot
1188.6122	1188.6777	0.0655	55	207	216	FLFCAEALFK				Mascot
1245.6433	1245.6448	0.0015	1	429	439	EGNEIIREASK				Mascot
1275.7307	1275.7518	0.0211	17	329	339	EITLGFVDLLR				Mascot
1275.7307	1275.7518	0.0211	17	329	339	EITLGFVDLLR	100	100		Mascot
1451.6219	1451.6488	0.0269	19	191	202	DDENVNSQPFMR				Mascot
1465.7546	1465.7505	-0.0041	-3	136	148	TFQGPPHGIQVER				Mascot
1467.6169	1467.6356	0.0187	13	191	202	DDENVNSQPFMR			Oxidation (M)[11]	Mascot
1467.6169	1467.6356	0.0187	13	191	202	DDENVNSQPFMR	34	89.349	Oxidation (M)[11]	Mascot
1481.7167	1481.6897	-0.027	-18	309	323	MSGGDHIHAGTVVGK			Oxidation (M)[1]	Mascot
1488.6927	1488.7096	0.0169	11	453	464	EIKFEFEAMDTL			Oxidation (M)[9]	Mascot

1489.7145	1489.6837	-0.0308	-21	440	452	WSPELAAACEVWK				Mascot
1546.736	1546.7526	0.0166	11	440	452	WSPELAAACEVWK			Carbamidomethyl (C)[9]	Mascot
1546.736	1546.7526	0.0166	11	440	452	WSPELAAACEVWK	83	100	Carbamidomethyl (C)[9]	Mascot
1812.7714	1812.7892	0.0178	10	226	241	GHYLNATAGTCEEMMK			Carbamidomethyl (C)[11]	Mascot
1828.7662	1828.7725	0.0063	3	226	241	GHYLNATAGTCEEMMK			Carbamidomethyl (C)[11], Oxidation (M)[14]	Mascot
1844.7612	1844.7668	0.0056	3	226	241	GHYLNATAGTCEEMMK			Carbamidomethyl (C)[11], Oxidation (M)[14,15]	Mascot
1904.9211	1904.8506	-0.0705	-37	436	452	EASKWSPELAAACEVWK				Mascot
1984.8674	1984.8883	0.0209	11	226	242	GHYLNATAGTCEEMMKR			Carbamidomethyl (C)[11], Oxidation (M)[14]	Mascot
2000.8623	2000.8718	0.0095	5	226	242	GHYLNATAGTCEEMMKR			Carbamidomethyl (C)[11], Oxidation (M)[14,15]	Mascot
2169.9871	2170.0247	0.0376	17	184	202	GGLDFTKDDENVNSQPFMR				Mascot
2169.9871	2170.0247	0.0376	17	184	202	GGLDFTKDDENVNSQPFMR	47	99.504		Mascot

2185.9819	2186.0059	0.024	11	184	202	GGLDFTKDDENVNSQPFMR			Oxidation (M)[18]		Mascot
2185.9819	2186.0059	0.024	11	184	202	GGLDFTKDDENVNSQPFMR	104	100	Oxidation (M)[18]		Mascot
2994.4238	2994.4961	0.0723	24	248	274	ELGAPIVMHDYLTGGFTANTSLAHYCR			Carbamidomethyl (C)[26]		Mascot
3010.4187	3010.4883	0.0696	23	248	274	ELGAPIVMHDYLTGGFTANTSLAHYCR			Carbamidomethyl (C)[26], Oxidation (M)[8]		Mascot
3010.4187	3010.4883	0.0696	23	248	274	ELGAPIVMHDYLTGGFTANTSLAHYCR	99	100	Carbamidomethyl (C)[26], Oxidation (M)[8]		Mascot

65

Nucleoside diphosphate kinase [Cucumis sativus]	gi|700198251	25963.5	9.18	9	152	100	111	100

Protein Group

PREDICTED: nucleoside diphosphate kinase 3-like [Cucumis sativus]	gi|449462537	25963.5	9.18000030517578

Peptide Information

Calc. Mass	Obsrv. Mass	± da	± ppm	Start Seq.	End Seq.	Sequence	Ion Score	C. I. %	Modification	Rank	Result Type
812.405	812.4135	0.0085	10	135	140	HYHDLK					Mascot
915.5258	915.5367	0.0109	12	190	198	GDLAVVVSR					Mascot
987.5581	987.5961	0.038	38	32	42	AAASAAAVSLR					Mascot
987.5833	987.5961	0.0128	13	103	111	GLISEIISR					Mascot
1033.5571	1033.5293	-0.0278	-27	5	14	ICRSISGAAR					Mascot
1338.6649	1338.6731	0.0082	6	199	211	NIIHGSDGPETAK					Mascot
1344.7634	1344.7819	0.0185	14	91	102	TFIAIKPDGVQR					Mascot
1344.7634	1344.7819	0.0185	14	91	102	TFIAIKPDGVQR	111	100			Mascot
1415.7067	1415.6949	-0.0118	-8	130	140	EFAQKHYHDLK					Mascot
3312.6182	3312.385	-0.2332	-70	141	169	ERPFFNGLCEFLSSGPVIAMVWEGEGVIR			Carbamidomethyl (C)[9], Oxidation (M)[20]		Mascot
3312.6182	3312.385	-0.2332	-70	141	169	ERPFFNGLCEFLSSGPVIAMVWEGEGVIR			Carbamidomethyl (C)[9], Oxidation (M)[20]		Mascot

66									
hypothetical protein Csa_5G609710 [Cucumis sativus]	gi|700196908	20685.2	8.24		14	456	100	355	100

Protein Group

PREDICTED: ribulose bisphosphate carboxylase small chain, chloroplastic [Cucumis sativus]	gi|449434620	20685.2	8.2399 997711 1816
ribulose-1,5-bisphosphate carboxylase small subunit [Cucumis sativus]	gi|379131260	20713.2	8.2399 997711 1816

Peptide Information

Calc. Mass	Obsrv. Mass	± da	± ppm	Start Seq.	End Seq.	Sequence	Ion Score	C. I. %	Modification	Rank	Result Type	
914.4003	914.4161	0.0158	17	117	124	SPGYYDGR	57	99.952			Mascot	
914.4229	914.4161	-0.0068	-7	125	130	YWTMWK					Mascot	
919.4996	919.5142	0.0146	16	160	167	VIGFDNVR					Mascot	
919.4996	919.5142	0.0146	16	160	167	VIGFDNVR	25	16.573			Mascot	
930.4178	930.4258	0.008	9	125	130	YWTMWK			Oxidation (M)[4]		Mascot	
935.4945	935.5127	0.0182	19	33	41	SSAGFPITR					Mascot	
937.5618	937.5733	0.0115	12	62	69	VWPPLGLR					Mascot	
937.5618	937.5733	0.0115	12	62	69	VWPPLGLR	23	0			Mascot	
968.4506	968.4642	0.0136	14	88	94	ECDYLLR			Carbamidomethyl (C)[2]		Mascot	
968.4506	968.4642	0.0136	14	88	94	ECDYLLR	26	32.964	Carbamidomethyl (C)[2]		Mascot	
1010.4941	1010.5062	0.0121	12	152	159	EYPDAFIR					Mascot	
1010.4941	1010.5062	0.0121	12	152	159	EYPDAFIR	60	99.975			Mascot	
1065.6567	1065.6696	0.0129	12	62	70	VWPPLGLRK					Mascot	
1138.5891	1138.6042	0.0151	13	151	159	KEYPDAFIR					Mascot	
1138.5891	1138.6042	0.0151	13	151	159	KEYPDAFIR	62	99.985			Mascot	
1649.8833	1649.9058	0.0225	14	168	181	QVQCISFIAYKPPR					Mascot	
1706.9047	1706.9227	0.018	11	168	181	QVQCISFIAYKPPR			Carbamidomethyl (C)[4]		Mascot	
1706.9047	1706.9227	0.018	11	168	181	QVQCISFIAYKPPR	104	100	Carbamidomethyl (C)[4]		Mascot	
2001.9474	2001.9497	0.0023	1	71	87	FETLSYLPDMSNEQLSK					Mascot	
2130.0422	2130.0544	0.0122	6	70	87	KFETLSYLPDMSNEQLSK					Mascot	
**Project 1\Sample project201(**	2130.0544										**455**	Mascot
2146.0374	2146.0452	0.0078	4	70	87	KFETLSYLPDMSNEQLSK			Oxidation (M)[11]		Mascot	

2282.0679	2282.0896	0.0217	10	131	150	LPMFGCTDSSQVIQEIEE AK			Carbamidomethyl (C)[6]		Mascot
2298.063	2298.0884	0.0254	11	131	150	LPMFGCTDSSQVIQEIEE AK			Carbamidomethyl (C)[6], Oxidation (M)[3]		Mascot
2410.1628	2410.1904	0.0276	11	131	151	LPMFGCTDSSQVIQEIEE AKK			Carbamidomethyl (C)[6]		Mascot
2426.1577	2426.1807	0.023	9	131	151	LPMFGCTDSSQVIQEIEE AKK			Carbamidomethyl (C)[6], Oxidation (M)[3]		Mascot

67

hypothetical protein Csa_5G609710 [Cucumis sativus]	gi|700196908	20685.2	8.24		13	405	100	316	100
**Protein Group**									
PREDICTED: ribulose bisphosphate carboxylase small chain, chloroplastic [Cucumis sativus]	gi|449434620	20685.2		8.2399997711 1816					
ribulose-1,5-bisphosphate carboxylase small subunit [Cucumis sativus]	gi|379131260	20713.2		8.2399997711 1816					

Peptide Information

Calc. Mass	Obsrv. Mass	± da	± ppm	Start Seq.	End Seq.	Sequence	Ion Score	C. I. %	Modification	Rank	Result Type
911.4291	911.4394	0.0103	11	88	94	ECDYLLR					Mascot
914.4003	914.4124	0.0121	13	117	124	SPGYYDGR	57	99.954			Mascot
914.4229	914.4124	-0.0105	-11	125	130	YWTMWK					Mascot
919.4996	919.5109	0.0113	12	160	167	VIGFDNVR					Mascot
919.4996	919.5109	0.0113	12	160	167	VIGFDNVR	42	98.306			Mascot
930.4178	930.4186	0.0008	1	125	130	YWTMWK			Oxidation (M)[4]		Mascot
937.5618	937.5705	0.0087	9	62	69	VWPPLGLR					Mascot
937.5618	937.5705	0.0087	9	62	69	VWPPLGLR	28	64.118			Mascot
968.4506	968.4623	0.0117	12	88	94	ECDYLLR			Carbamidomethyl (C)[2]		Mascot
1010.4941	1010.5022	0.0081	8	152	159	EYPDAFIR					Mascot
1010.4941	1010.5022	0.0081	8	152	159	EYPDAFIR	59	99.969			Mascot
1138.5891	1138.5994	0.0103	9	151	159	KEYPDAFIR					Mascot
1138.5891	1138.5994	0.0103	9	151	159	KEYPDAFIR	40	97.546			Mascot
1706.9047	1706.9176	0.0129	8	168	181	QVQCISFIAYKPPR			Carbamidomethyl (C)[4]		Mascot

2017.9424	2017.9219	-0.0205	-10	71	87	FETLSYLPDMSNEQLSK			Oxidation (M)[10]		Mascot
2130.0422	2130.0283	-0.0139	-7	70	87	KFETLSYLPDMSNEQLSK					Mascot
2134.0791	2133.9973	-0.0818	-38	168	185	QVQCISFIAYKPPRFYSS					Mascot
2146.0374	2146.0295	-0.0079	-4	70	87	KFETLSYLPDMSNEQLSK			Oxidation (M)[11]		Mascot
2146.0374	2146.0295	-0.0079	-4	70	87	KFETLSYLPDMSNEQLSK	91	100	Oxidation (M)[11]		Mascot
2225.0464	2225.1252	0.0788	35	131	150	LPMFGCTDSSQVIQEIEE					Mascot

Project 1\Sample project201(2225.1252 **462** Mascot

						AK					
2298.063	2298.0667	0.0037	2	131	150	LPMFGCTDSSQVIQEIEE AK			Carbamidomethyl (C)[6], Oxidation (M)[3]		Mascot
2426.1577	2426.166	0.0083	3	131	151	LPMFGCTDSSQVIQEIEE AKK			Carbamidomethyl (C)[6], Oxidation (M)[3]		Mascot

68

Catalase isozyme 3 [Cucumis sativus] gi|700192329 57009.2 6.84 24 585 100 453 100

Peptide Information

Calc. Mass	Obsrv. Mass	± da	± ppm	Start Seq.	End Seq.	Sequence	Ion Score	C. I. %	Modification	Rank	Result Type
843.4505	843.4251	-0.0254	-30	486	492	MNVRPNI					Mascot
988.5211	988.5323	0.0112	11	103	110	FSTVIHER					Mascot
988.5211	988.5323	0.0112	11	103	110	FSTVIHER	42	98.487			Mascot
1033.5314	1033.5391	0.0077	7	450	458	FVEALSDPR					Mascot
1052.5272	1052.5367	0.0095	9	164	171	SHIQEPWR					Mascot
1052.5272	1052.5367	0.0095	9	164	171	SHIQEPWR	21	0			Mascot
1058.4902	1058.501	0.0108	10	344	352	SFAYADTQR					Mascot
1058.4902	1058.501	0.0108	10	344	352	SFAYADTQR	47	99.51			Mascot
1093.5194	1093.5327	0.0133	12	234	242	SMLEEEAIR			Oxidation (M)[2]		Mascot
1127.5804	1127.5861	0.0057	5	111	120	GSPETLRDPR					Mascot
1136.6787	1136.6875	0.0088	8	92	102	APGVQTPVIVR					Mascot
1136.6787	1136.6875	0.0088	8	92	102	APGVQTPVIVR	14	0			Mascot
1189.6324	1189.6335	0.0011	1	449	458	RFVEALSDPR					Mascot
1191.5828	1191.6068	0.024	20	147	156	DAMQFPDVIR					Mascot
1202.6239	1202.6251	0.0012	1	405	415	FPMPPNVLSGK			Oxidation (M)[3]		Mascot
1207.5776	1207.5884	0.0108	9	147	156	DAMQFPDVIR			Oxidation (M)[3]		Mascot

1207.5776	1207.5884	0.0108	9	147	156	DAMQFPDVIR	52	99.856	Oxidation (M)[3]		Mascot
1255.559	1255.572	0.013	10	386	395	DEEVNYFPSR					Mascot
1255.559	1255.572	0.013	10	386	395	DEEVNYFPSR	53	99.867			Mascot
1259.6355	1259.63	-0.0055	-4	224	233	FHWKPTCGVK			Carbamidomethyl (C)[7]		Mascot
1523.858	1523.8505	-0.0075	-5	355	368	LGPNYLQLPVNAPK					Mascot
1530.8414	1530.8473	0.0059	4	38	50	GPILLEDYQLIEK					Mascot
1538.7386	1538.761	0.0224	15	465	476	NIWISYWSQADR					Mascot
1542.8615	1542.7561	-0.1054	-68	221	233	LVKFHWKPTCGVK					Mascot
1673.8064	1673.8674	0.061	36	339	352	MLQARSFAYADTQR			Oxidation (M)[1]		Mascot
1709.8317	1709.8138	-0.0179	-10	201	215	HMEGFGVQAYSLINK			Oxidation (M)[2]		Mascot
1817.0181	1816.8385	-0.1796	-99	353	368	HRLGPNYLQLPVNAPK					Mascot

1817.0181	1816.8385	-0.1796	-99	353	368	HRLGPNYLQLPVNAPK					Mascot
1970.976	1970.9966	0.0206	10	130	146	EGNFDIVGNNFPVFFVR					Mascot
1970.976	1970.9966	0.0206	10	130	146	EGNFDIVGNNFPVFFVR	149	100			Mascot
2229.0281	2229.0503	0.0222	10	73	91	GFFEVTHDVSDLTCADFL R			Carbamidomethyl (C)[14]		Mascot
2229.0281	2229.0503	0.0222	10	73	91	GFFEVTHDVSDLTCADFL R	74	100	Carbamidomethyl (C)[14]		Mascot
2233.8975	2233.8721	-0.0254	-11	369	385	CPHHNNHHEGFMNFMH R			Carbamidomethyl (C)[1], Oxidation (M)[12,15]		Mascot
2871.3445	2871.3865	0.042	15	243	268	IGGTNHSHATQDLYESIA AGNFPEWR					Mascot

69

hypothetical protein Csa_4G658590 [Cucumis sativus]	gi|700200341	57052.3	6.8	25	638	100	493	100
**Protein Group**								
PREDICTED: catalase isozyme 1 [Cucumis sativus]	gi|778697155	57052.3	6.8000 001907 3486					

Peptide Information

Calc. Mass	Obsrv. Mass	± da	± ppm	Start Seq.	End Seq.	Sequence	Ion Score	C. I. %	Modification	Rank	Result Type
988.5211	988.5262	0.0051	5	103	110	FSTVIHER					Mascot
1058.5266	1058.5294	0.0028	3	450	458	WVDALSDPR					Mascot

	1069.5175	1069.5258	0.0083	8	164	171	SHIQENWR				Mascot	
	1069.5175	1069.5258	0.0083	8	164	171	SHIQENWR	33	87.286		Mascot	
	1114.5388	1114.5029	-0.0359	-32	397	405	YDPARHAER				Mascot	
	1127.5804	1127.582	0.0016	1	111	120	GSPETLRDPR				Mascot	
	1136.6787	1136.6842	0.0055	5	92	102	APGVQTPVIVR				Mascot	
	1206.6953	1206.7024	0.0071	6	482	492	LASHLNVRPSI				Mascot	
	1212.5784	1212.6333	0.0549	45	282	291	YDFDPLDVTK				Mascot	
	1214.6277	1214.6339	0.0062	5	449	458	RWVDALSDPR				Mascot	
	1219.5702	1219.574	0.0038	3	425	434	ENNFKEPGER				Mascot	
	1226.5988	1226.6064	0.0076	6	406	416	YPHPPAVCTGK			Carbamidomethyl (C)[8]	Mascot	
	1255.559	1255.5686	0.0096	8	387	396	DEEVNYFPSR				Mascot	
	1287.6416	1287.6521	0.0105	8	224	233	FHWRPTCGVK			Carbamidomethyl (C)[7]	Mascot	
	1287.6416	1287.6521	0.0105	8	224	233	FHWRPTCGVK	3	0	Carbamidomethyl (C)[7]	Mascot	
	1495.8268	1495.8201	-0.0067	-4	356	369	LGPNYLQLPANAPK				Mascot	
	1525.7434	1525.7611	0.0177	12	465	476	SIWITYWSQADR				Mascot	
	1525.7434	1525.7611	0.0177	12	465	476	SIWITYWSQADR	34	91.102		Mascot	
	1598.781	1598.7972	0.0162	10	269	281	LFIQTIDPDHEDR				Mascot	
	1620.8745	1620.8832	0.0087	5	292	305	TWPEDILPLQPVGR				Mascot	
	1620.8745	1620.8832	0.0087	5	292	305	TWPEDILPLQPVGR	108	100		Mascot	
	1940.9722	1940.9963	0.0241	12	147	163	DGMKFPDMVHALKPNPK			Oxidation (M)[3]	Mascot	
	1956.967	1956.991	0.024	12	147	163	DGMKFPDMVHALKPNPK			Oxidation (M)[3,8]	Mascot	
**Project 1\Sample project201(**		1956.991									**477**	Mascot
	1984.9917	1985.0031	0.0114	6	130	146	EGNFDLVGNNFPVFFIR				Mascot	
	1984.9917	1985.0031	0.0114	6	130	146	EGNFDLVGNNFPVFFIR	153	100		Mascot	
	2175.8918	2175.8796	-0.0122	-6	370	386	CAHHNNHHEGFMNFMH<br>R			Carbamidomethyl (C)[1]	Mascot	
	2191.887	2191.8774	-0.0096	-4	370	386	CAHHNNHHEGFMNFMH<br>R			Carbamidomethyl (C)[1], Oxidation (M)[12]	Mascot	
	2207.8818	2207.8816	-0.0002	0	370	386	CAHHNNHHEGFMNFMH<br>R			Carbamidomethyl (C)[1], Oxidation (M)[12,15]	Mascot	
	2242.0598	2242.0735	0.0137	6	73	91	GFFEVTHDITNLSCADFL<br>R			Carbamidomethyl (C)[14]	Mascot	
	2242.0598	2242.0735	0.0137	6	73	91	GFFEVTHDITNLSCADFL<br>R	130	100	Carbamidomethyl (C)[14]	Mascot	
	2314.176	2314.1733	-0.0027	-1	224	242	FHWRPTCGVKSLLEEDAI			Carbamidomethyl (C)[7]	Mascot	

						R					
2792.3413	2792.3574	0.0161	6	269	291	LFIQTIDPDHEDRYDFDPL DVTK					Mascot
2792.3413	2792.3574	0.0161	6	269	291	LFIQTIDPDHEDRYDFDPL DVTK	35	92.531			Mascot
2817.2864	2817.3	0.0136	5	243	268	VGGSNHSHATQDLYDSI AAGNYPEWK					Mascot
3501.6685	3501.7175	0.049	14	6	37	HRPSSGYNTPFWTTNSG APVWNNNSSLTVGPR					Mascot

70

Beta-form rubisco activase [Cucumis sativus]	gi|700195391	48292.6	8.19		23	723	100	592	100

Protein Group

PREDICTED: ribulose bisphosphate carboxylase/oxygenase activase 2, chloroplastic-like [Cucumis sativus]	gi|449459892	48292.6	8.1899 995803 833

Peptide Information

Calc. Mass	Obsrv. Mass	± da	± ppm	Start Seq.	End Seq.	Sequence	Ion Score	C. I. %	Modification	Rank	Result Type
809.5244	809.5201	-0.0043	-5	149	155	LVVHITK					Mascot
895.4156	895.4248	0.0092	10	360	366	VYDDEVR					Mascot
940.4675	940.4709	0.0034	4	311	317	FYWAPTR					Mascot
940.4675	940.4709	0.0034	4	311	317	FYWAPTR	33	89.475			Mascot
1023.5105	1023.5042	-0.0063	-6	360	367	VYDDEVRK					Mascot
1152.714	1152.7137	-0.0003	0	165	175	VPLILGLWGGK					Mascot
1209.582	1209.5527	-0.0293	-24	391	400	FEQPTMSLEK					Mascot
1228.6031	1228.603	-0.0001	0	180	189	SFQCELVFAK			Carbamidomethyl (C)[4]		Mascot
1340.6382	1340.6416	0.0034	3	311	320	FYWAPTREDR					Mascot
1639.7567	1639.7626	0.0059	4	230	244	MSCLFINDLDAGAGR			Carbamidomethyl (C)[3]		Mascot
1639.7567	1639.7626	0.0059	4	230	244	MSCLFINDLDAGAGR	93	100	Carbamidomethyl (C)[3]		Mascot
1655.7517	1655.7527	0.001	1	230	244	MSCLFINDLDAGAGR			Carbamidomethyl (C)[3], Oxidation (M)[1]		Mascot
1757.8778	1757.8607	-0.0171	-10	386	400	EPPPKFEQPTMSLEK					Mascot
1801.9919	1801.9326	-0.0593	-33	40	56	VVNSRVVNPKPSSGSFK					Mascot
1838.9794	1838.934	-0.0454	-25	1	19	MAASAASVGVVNHAPLS LK			Oxidation (M)[1]		Mascot

1849.9364	1849.9268	-0.0096	-5	401	416	LLEYGGMLVQEQENVK				Mascot	
1865.9314	1865.9408	0.0094	5	401	416	LLEYGGMLVQEQENVK			Oxidation (M)[7]	Mascot	
1882.9698	1882.9771	0.0073	4	341	357	LVDTFPGQSIDFFGALR				Mascot	
1882.9698	1882.9771	0.0073	4	341	357	LVDTFPGQSIDFFGALR	138	100		Mascot	
1890.8828	1890.8895	0.0067	4	78	94	WAGLGTDISDDQQDITR				Mascot	
2006.0376	2006.0433	0.0057	3	401	417	LLEYGGMLVQEQENVKR				Mascot	
2006.0376	2006.0433	0.0057	3	401	417	LLEYGGMLVQEQENVKR	50	99.797		Mascot	
2022.0325	2022.0352	0.0027	1	401	417	LLEYGGMLVQEQENVKR			Oxidation (M)[7]	Mascot	
**Project 1\Sample project201(**	2022.0352									**486**	Mascot
2089.1692	2089.175	0.0058	3	286	304	VPIIVTGNDFSTLYAPLIR				Mascot	
2089.1692	2089.175	0.0058	3	286	304	VPIIVTGNDFSTLYAPLIR	99	100		Mascot	
2291.0715	2291.0403	-0.0312	-14	190	212	MGITPIMMSAGELESGNA GEPAK				Mascot	
2293.0515	2293.0439	-0.0076	-3	128	148	SYDGMDNVLGGLYIAPAF MDK			Oxidation (M)[5]	Mascot	
2405.1516	2405.1384	-0.0132	-5	127	148	KSYDGMDNVLGGLYIAP AFMDK				Mascot	
2476.2314	2476.2424	0.011	4	418	440	VQLAETYLNEAALGNANE DAITR				Mascot	
2476.2314	2476.2424	0.011	4	418	440	VQLAETYLNEAALGNANE DAITR	139	100		Mascot	
2632.3325	2632.3308	-0.0017	-1	417	440	RVQLAETYLNEAALGNA NEDAITR				Mascot	
3310.4966	3310.5278	0.0312	9	97	126	GMVDSVFQAPMQAGTH YAVMSSYEYLSQGR				Mascot	
3310.4966	3310.5278	0.0312	9	97	126	GMVDSVFQAPMQAGTH YAVMSSYEYLSQGR	40	98.158		Mascot	
3326.4915	3326.5139	0.0224	7	97	126	GMVDSVFQAPMQAGTH YAVMSSYEYLSQGR			Oxidation (M)[2]	Mascot	
3342.4866	3342.4954	0.0088	3	97	126	GMVDSVFQAPMQAGTH YAVMSSYEYLSQGR			Oxidation (M)[2,11]	Mascot	

71	ribulose-1,5-bisphosphate carboxylase/oxygenase large	gi|111182702	51456.9	6	21	756	100	646	100

subunit, partial (chloroplast) [Cucumis hystrix]

Peptide Information

Calc. Mass	Obsrv. Mass	± da	± ppm	Start Seq.	End Seq.	Sequence	Ion Score	C. I. %	Modification	Rank	Result Type
830.4366	830.4485	0.0119	14	429	435	EGNEIIR					Mascot
853.4236	853.4232	-0.0004	0	177	183	AVYECLR					Mascot
898.41	898.4143	0.0043	5	295	301	NHGMHFR					Mascot
910.4451	910.4497	0.0046	5	177	183	AVYECLR			Carbamidomethyl (C)[5]		Mascot
910.4451	910.4497	0.0046	5	177	183	AVYECLR	29	68.85	Carbamidomethyl (C)[5]		Mascot
912.472	912.4608	-0.0112	-12	285	292	AMHAVIDR					Mascot
914.405	914.4139	0.0089	10	295	301	NHGMHFR			Oxidation (M)[4]		Mascot
928.4669	928.4606	-0.0063	-7	285	292	AMHAVIDR			Oxidation (M)[2]		Mascot
962.4789	962.4802	0.0013	1	217	225	SQAETGEIK					Mascot
1023.4741	1023.4772	0.0031	3	340	347	DDFVEKDR					Mascot
1059.5615	1059.549	-0.0125	-12	411	420	VALEACVQAR					Mascot
1116.583	1116.589	0.006	5	411	420	VALEACVQAR			Carbamidomethyl (C)[6]		Mascot
1116.583	1116.589	0.006	5	411	420	VALEACVQAR	23	0	Carbamidomethyl (C)[6]		Mascot
1154.5636	1154.592	0.0284	25	293	301	QKNHGMHFR					Mascot
1170.5586	1170.6447	0.0861	74	293	301	QKNHGMHFR			Oxidation (M)[6]		Mascot
1170.5586	1170.6447	0.0861	74	293	301	QKNHGMHFR			Oxidation (M)[6]		Mascot
1187.6644	1187.6715	0.0071	6	275	284	DNGLLLHIHR					Mascot
1188.6122	1188.6649	0.0527	44	207	216	FLFCAEALFK					Mascot
1245.6337	1245.6309	-0.0028	-2	207	216	FLFCAEALFK			Carbamidomethyl (C)[4]		Mascot
1275.7307	1275.7363	0.0056	4	329	339	EITLGFVDLLR					Mascot
1275.7307	1275.7363	0.0056	4	329	339	EITLGFVDLLR	103	100			Mascot
1451.6219	1451.6287	0.0068	5	191	202	DDENVNSQPFMR					Mascot
1451.6219	1451.6287	0.0068	5	191	202	DDENVNSQPFMR	85	100			Mascot
1465.7217	1465.6659	-0.0558	-38	309	323	MSGGDHIHAGTVVGK					Mascot
1467.6169	1467.6215	0.0046	3	191	202	DDENVNSQPFMR			Oxidation (M)[11]		Mascot
1472.6978	1472.6609	-0.0369	-25	453	464	EIKFEFEAMDTL					Mascot

1481.7167	1481.657	-0.0597	-40	309	323	MSGGDHIHAGTVVGK			Oxidation (M)[1]		Mascot
1488.6927	1488.6704	-0.0223	-15	453	464	EIKFEFEAMDTL			Oxidation (M)[9]		Mascot
1516.7617	1516.7577	-0.004	-3	205	216	DRFLFCAEALFK			Carbamidomethyl (C)[6]		Mascot
1546.736	1546.7327	-0.0033	-2	440	452	WSPELAAACEVWK			Carbamidomethyl (C)[9]		Mascot

1546.736	1546.7327	-0.0033	-2	440	452	WSPELAAACEVWK	74	100	Carbamidomethyl (C)[9]		Mascot
1812.7714	1812.7661	-0.0053	-3	226	241	GHYLNATAGTCEEMMK			Carbamidomethyl (C)[11]		Mascot
1828.7662	1828.7483	-0.0179	-10	226	241	GHYLNATAGTCEEMMK			Carbamidomethyl (C)[11], Oxidation (M)[14]		Mascot
1904.9211	1904.8326	-0.0885	-46	436	452	EASKWSPELAAACEVWK					Mascot
1968.8724	1968.8693	-0.0031	-2	226	242	GHYLNATAGTCEEMMKR			Carbamidomethyl (C)[11]		Mascot
1984.8674	1984.8618	-0.0056	-3	226	242	GHYLNATAGTCEEMMKR			Carbamidomethyl (C)[11], Oxidation (M)[14]		Mascot
2000.8623	2000.8406	-0.0217	-11	226	242	GHYLNATAGTCEEMMKR			Carbamidomethyl (C)[11], Oxidation (M)[14,15]		Mascot
2169.9871	2169.9934	0.0063	3	184	202	GGLDFTKDDENVNSQPFMR					Mascot
2169.9871	2169.9934	0.0063	3	184	202	GGLDFTKDDENVNSQPFMR	144	100			Mascot
2185.9819	2185.9783	-0.0036	-2	184	202	GGLDFTKDDENVNSQPFMR			Oxidation (M)[18]		Mascot
2185.9819	2185.9783	-0.0036	-2	184	202	GGLDFTKDDENVNSQPFMR	15	0	Oxidation (M)[18]		Mascot
2994.4238	2994.4534	0.0296	10	248	274	ELGAPIVMHDYLTGGFTANTSLAHYCR			Carbamidomethyl (C)[26]		Mascot
2994.4238	2994.4534	0.0296	10	248	274	ELGAPIVMHDYLTGGFTANTSLAHYCR	187	100	Carbamidomethyl (C)[26]		Mascot
3010.4187	3010.4431	0.0244	8	248	274	ELGAPIVMHDYLTGGFTANTSLAHYCR			Carbamidomethyl (C)[26], Oxidation (M)[8]		Mascot
3010.4187	3010.4431	0.0244	8	248	274	ELGAPIVMHDYLTGGFTANTSLAHYCR	112	100	Carbamidomethyl (C)[26], Oxidation (M)[8]		Mascot

72	ribulose-1,5-bisphosphate carboxylase/oxygenase large subunit, partial (chloroplast) [Cucumis hystrix]	gi|111182702	51456.9	6	21	586	100	475	100

Peptide Information

Calc. Mass	Obsrv. Mass	± da	± ppm	Start Seq.	End Seq.	Sequence	Ion Score	C. I. %	Modification	Rank	Result Type
830.4366	830.4482	0.0116	14	429	435	EGNEIIR					Mascot
898.41	898.4178	0.0078	9	295	301	NHGMHFR					Mascot
910.4451	910.4515	0.0064	7	177	183	AVYECLR			Carbamidomethyl (C)[5]		Mascot

910.4451	910.4515	0.0064	7	177	183	AVYECLR	9	0	Carbamidomethyl (C)[5]	Mascot
912.472	912.4621	-0.0099	-11	285	292	AMHAVIDR				Mascot
914.405	914.4131	0.0081	9	295	301	NHGMHFR			Oxidation (M)[4]	Mascot
928.4669	928.4742	0.0073	8	285	292	AMHAVIDR			Oxidation (M)[2]	Mascot
962.4789	962.4772	-0.0017	-2	217	225	SQAETGEIK				Mascot
1023.4741	1023.4795	0.0054	5	340	347	DDFVEKDR				Mascot
1059.5615	1059.5374	-0.0241	-23	411	420	VALEACVQAR				Mascot
1116.583	1116.59	0.007	6	411	420	VALEACVQAR			Carbamidomethyl (C)[6]	Mascot
1116.583	1116.59	0.007	6	411	420	VALEACVQAR	37	95.269	Carbamidomethyl (C)[6]	Mascot
1154.5636	1154.5735	0.0099	9	293	301	QKNHGMHFR				Mascot
1170.5586	1170.6476	0.089	76	293	301	QKNHGMHFR			Oxidation (M)[6]	Mascot
1170.5586	1170.6476	0.089	76	293	301	QKNHGMHFR			Oxidation (M)[6]	Mascot
1187.6644	1187.675	0.0106	9	275	284	DNGLLLHIHR				Mascot
1187.6644	1187.675	0.0106	9	275	284	DNGLLLHIHR	39	97.394		Mascot
1188.6122	1188.6692	0.057	48	207	216	FLFCAEALFK				Mascot
1245.6337	1245.6343	0.0006	0	207	216	FLFCAEALFK			Carbamidomethyl (C)[4]	Mascot
1275.7307	1275.7385	0.0078	6	329	339	EITLGFVDLLR				Mascot
1275.7307	1275.7385	0.0078	6	329	339	EITLGFVDLLR	106	100		Mascot
1451.6219	1451.6307	0.0088	6	191	202	DDENVNSQPFMR				Mascot
1451.6219	1451.6307	0.0088	6	191	202	DDENVNSQPFMR	47	99.503		Mascot
1465.7217	1465.6821	-0.0396	-27	309	323	MSGGDHIHAGTVVGK				Mascot
1467.6169	1467.6229	0.006	4	191	202	DDENVNSQPFMR			Oxidation (M)[11]	Mascot
1472.6978	1472.674	-0.0238	-16	453	464	EIKFEFEAMDTL				Mascot

1481.7167	1481.6683	-0.0484	-33	309	323	MSGGDHIHAGTVVGK			Oxidation (M)[1]	Mascot
1488.6927	1488.6864	-0.0063	-4	453	464	EIKFEFEAMDTL			Oxidation (M)[9]	Mascot
1546.736	1546.7357	-0.0003	0	440	452	WSPELAAACEVWK			Carbamidomethyl (C)[9]	Mascot
1812.7714	1812.7677	-0.0037	-2	226	241	GHYLNATAGTCEEMMK			Carbamidomethyl (C)[11]	Mascot
1828.7662	1828.7664	0.0002	0	226	241	GHYLNATAGTCEEMMK			Carbamidomethyl (C)[11], Oxidation (M)[14]	Mascot
1844.7612	1844.7611	-0.0001	0	226	241	GHYLNATAGTCEEMMK			Carbamidomethyl (C)[11], Oxidation (M)[14,15]	Mascot
1904.9211	1904.8201	-0.101	-53	436	452	EASKWSPELAAACEVWK				Mascot
1984.8674	1984.8783	0.0109	5	226	242	GHYLNATAGTCEEMMKR			Carbamidomethyl (C)[11], Oxidation (M)[14]	Mascot
2000.8623	2000.8569	-0.0054	-3	226	242	GHYLNATAGTCEEMMKR			Carbamidomethyl (C)[11], Oxidation (M)[14,15]	Mascot

2009.0591	2009.006	-0.0531	-26	329	345	EITLGFVDLLRDDFVEK					Mascot
2169.9871	2169.9958	0.0087	4	184	202	GGLDFTKDDENVNSQPF MR					Mascot
2169.9871	2169.9958	0.0087	4	184	202	GGLDFTKDDENVNSQPF MR	109	100			Mascot
2185.9819	2185.9844	0.0025	1	184	202	GGLDFTKDDENVNSQPF MR			Oxidation (M)[18]		Mascot
2185.9819	2185.9844	0.0025	1	184	202	GGLDFTKDDENVNSQPF MR	53	99.876	Oxidation (M)[18]		Mascot
2953.3972	2953.4883	0.0911	31	248	274	ELGAPIVMHDYLTGGFTA NTSLAHYCR			Oxidation (M)[8]		Mascot
2994.4238	2994.4575	0.0337	11	248	274	ELGAPIVMHDYLTGGFTA NTSLAHYCR			Carbamidomethyl (C)[26]		Mascot
2994.4238	2994.4575	0.0337	11	248	274	ELGAPIVMHDYLTGGFTA NTSLAHYCR	127	100	Carbamidomethyl (C)[26]		Mascot
3010.4187	3010.4429	0.0242	8	248	274	ELGAPIVMHDYLTGGFTA NTSLAHYCR			Carbamidomethyl (C)[26], Oxidation (M)[8]		Mascot
3010.4187	3010.4429	0.0242	8	248	274	ELGAPIVMHDYLTGGFTA NTSLAHYCR	97	100	Carbamidomethyl (C)[26], Oxidation (M)[8]		Mascot

73

hypothetical protein Csa_6G450370 [Cucumis sativus]	gi|700193028	47714.4	5.48	18	421	100	331	100	

Protein Group

PREDICTED: enolase isoform X1 [Cucumis sativus]	gi|449451102	47714.4	5.4800 000190 7349

Peptide Information

Calc. Mass	Obsrv. Mass	± da	± ppm	Start Seq.	End Seq.	Sequence	Ion Score	C. I. %	Modification	Rank	Result Type
806.4519	806.4554	0.0035	4	416	421	YNQLLR					Mascot
1087.5103	1087.5144	0.0041	4	372	381	AGWGVMASHR			Oxidation (M)[6]		Mascot
1228.6144	1228.6011	-0.0133	-11	189	198	MGVEVYHHLK			Oxidation (M)[1]		Mascot
1510.8477	1510.8141	-0.0336	-22	322	335	VQIVGDDLLVTNPK					Mascot
1564.9058	1564.8541	-0.0517	-33	66	81	AVENVNAIIGPALVGK					Mascot
1573.8433	1573.8282	-0.0151	-10	353	367	VNQIGSVTESIEAVK					Mascot

1804.944	1804.946	0.002	1	35	52	AAVPSGASTGIYEALELR				Mascot	
1804.944	1804.946	0.002	1	35	52	AAVPSGASTGIYEALELR	139	100		Mascot	
1824.9127	1824.9181	0.0054	3	422	438	IEEELGSAAVYAGVNFR				Mascot	
1824.9127	1824.9181	0.0054	3	422	438	IEEELGSAAVYAGVNFR	132	100		Mascot	
1841.9604	1841.9556	-0.0048	-3	17	34	GNPTVEVDIVLSDGSLAR				Mascot	
1841.9604	1841.9556	-0.0048	-3	17	34	GNPTVEVDIVLSDGSLAR	47	99.582		Mascot	
1882.9806	1882.892	-0.0886	-47	168	184	LAMQEFMILPIGASSFK				Mascot	
1901.8512	1901.8439	-0.0073	-4	262	277	TYDLNFKEENNDGSQK				Mascot	
1914.9705	1914.9341	-0.0364	-19	168	184	LAMQEFMILPIGASSFK			Oxidation (M)[3,7]	Mascot	
1914.9705	1914.9341	-0.0364	-19	168	184	LAMQEFMILPIGASSFK	13	0	Oxidation (M)[3,7]	Mascot	
1920.0107	1919.9653	-0.0454	-24	353	370	VNQIGSVTESIEAVKMSK				Mascot	
2252.1294	2252.125	-0.0044	-2	382	403	SGETEDTFIADLSVGLAT GQIK				Mascot	
2324.0425	2324.0298	-0.0127	-5	205	226	YGQDATNVGDEGGFAPN IQENK				Mascot	
2411.1072	2411.0864	-0.0208	-9	239	261	AGYTSQVVIGMDVAASE FYGSDK			Oxidation (M)[11]	Mascot	
2452.1375	2452.1282	-0.0093	-4	204	226	KYGQDATNVGDEGGFAP NIQENK				Mascot	
2480.282	2480.2795	-0.0025	-1	422	444	IEEELGSAAVYAGVNFRK				Mascot	
**Project 1\Sample project201(**	2480.2795									**523**	Mascot
						PVAPY					
2988.321	2988.3196	-0.0014	0	289	313	SFASEYPIVSIEDPFDQD DWEHYAK				Mascot	

74

hypothetical protein Csa_3G740070 [Cucumis sativus]	gi|700203830	30479.5	5.84		9	173	100	133	100
**Protein Group**									
PREDICTED: 29 kDa ribonucleoprotein, chloroplastic [Cucumis sativus]	gi|449440111	30479.5		5.8400 001525 8789					

Peptide Information

Calc. Mass	Obsrv. Mass	± da	± ppm	Start Seq.	End Seq.	Sequence	Ion Score	C. I. %	Modification	Rank	Result Type
865.4666	865.4692	0.0026	3	122	128	VEVIYDK					Mascot
882.4064	882.4147	0.0083	9	175	181	RDDSSFR					Mascot
968.52	968.5192	-0.0008	-1	166	174	VNYGPPPPK					Mascot
968.52	968.5192	-0.0008	-1	166	174	VNYGPPPPK	64	99.992			Mascot
987.5469	987.5349	-0.012	-12	215	223	EKGNVLEAK					Mascot
1124.6171	1124.6162	-0.0009	-1	266	275	VTQAEARPPR					Mascot
1124.6171	1124.6162	-0.0009	-1	266	275	VTQAEARPPR	50	99.798			Mascot
1280.6846	1280.6934	0.0088	7	122	132	VEVIYDKTTGR					Mascot
2865.4783	2865.5095	0.0312	11	95	121	LFVGNLPFTVDSAQLAGL FESAGQVER					Mascot
2865.4783	2865.5095	0.0312	11	95	121	LFVGNLPFTVDSAQLAGL FESAGQVER					Mascot
2980.3782	2980.3889	0.0107	4	135	162	GFGFVTMSTVGEVEAAA QQFNGYELDGR					Mascot
2996.373	2996.3914	0.0184	6	135	162	GFGFVTMSTVGEVEAAA QQFNGYELDGR			Oxidation (M)[7]		Mascot
3368.6394	3368.658	0.0186	6	235	265	GFGFVTYNSAEEVNEAIQ SLDGVDLDGRPIR					Mascot
3368.6394	3368.658	0.0186	6	235	265	GFGFVTYNSAEEVNEAIQ SLDGVDLDGRPIR	20	0			Mascot

75	ribulose-1,5-bisphosphate carboxylase/oxygenase large subunit, partial (chloroplast) [Cucumis hystrix]	gi|111182702	51456.9	6	24	502	100	362	100

Peptide Information

Calc. Mass	Obsrv. Mass	± da	± ppm	Start Seq.	End Seq.	Sequence	Ion Score	C. I. %	Modification	Rank	Result Type
830.4366	830.4509	0.0143	17	429	435	EGNEIIR					Mascot
898.41	898.4214	0.0114	13	295	301	NHGMHFR					Mascot
910.4451	910.4567	0.0116	13	177	183	AVYECLR			Carbamidomethyl (C)[5]		Mascot
912.472	912.4658	-0.0062	-7	285	292	AMHAVIDR					Mascot
914.405	914.4141	0.0091	10	295	301	NHGMHFR			Oxidation (M)[4]		Mascot

914.405	914.4141	0.0091	10	295	301	NHGMHFR			Oxidation (M)[4]	Mascot
928.4669	928.4631	-0.0038	-4	285	292	AMHAVIDR			Oxidation (M)[2]	Mascot
930.4752	930.4199	-0.0553	-59	421	428	NEGRDLAR				Mascot
962.4789	962.4514	-0.0275	-29	217	225	SQAETGEIK				Mascot
1021.5312	1021.5391	0.0079	8	22	30	DTDILAAFR				Mascot
1116.583	1116.5953	0.0123	11	411	420	VALEACVQAR			Carbamidomethyl (C)[6]	Mascot
1116.583	1116.5953	0.0123	11	411	420	VALEACVQAR	46	99.431	Carbamidomethyl (C)[6]	Mascot
1154.5636	1154.5685	0.0049	4	293	301	QKNHGMHFR				Mascot
1170.5586	1170.6501	0.0915	78	293	301	QKNHGMHFR			Oxidation (M)[6]	Mascot
1170.5586	1170.6501	0.0915	78	293	301	QKNHGMHFR			Oxidation (M)[6]	Mascot
1187.6644	1187.6761	0.0117	10	275	284	DNGLLLHIHR				Mascot
1188.6122	1188.6661	0.0539	45	207	216	FLFCAEALFK				Mascot
1275.7307	1275.7424	0.0117	9	329	339	EITLGFVDLLR				Mascot
1275.7307	1275.7424	0.0117	9	329	339	EITLGFVDLLR	73	99.999		Mascot
1407.6678	1407.6605	-0.0073	-5	11	21	LTYYTPEYETK				Mascot
1451.6219	1451.6362	0.0143	10	191	202	DDENVNSQPFMR				Mascot
1451.6219	1451.6362	0.0143	10	191	202	DDENVNSQPFMR	96	100		Mascot
1465.7546	1465.7594	0.0048	3	136	148	TFQGPPHGIQVER				Mascot
1467.6169	1467.7129	0.096	65	191	202	DDENVNSQPFMR			Oxidation (M)[11]	Mascot
1481.7167	1481.7123	-0.0044	-3	309	323	MSGGDHIHAGTVVGK			Oxidation (M)[1]	Mascot
1502.8512	1502.8629	0.0117	8	154	166	YGRPLLGCTIKPK			Carbamidomethyl (C)[8]	Mascot

1546.736	1546.7422	0.0062	4	440	452	WSPELAAACEVWK			Carbamidomethyl (C)[9]	Mascot
1812.7714	1812.7769	0.0055	3	226	241	GHYLNATAGTCEEMMK			Carbamidomethyl (C)[11]	Mascot
1828.7662	1828.7723	0.0061	3	226	241	GHYLNATAGTCEEMMK			Carbamidomethyl (C)[11], Oxidation (M)[14]	Mascot
1904.9211	1904.8501	-0.071	-37	436	452	EASKWSPELAAACEVWK				Mascot
1968.8724	1968.8899	0.0175	9	226	242	GHYLNATAGTCEEMMKR			Carbamidomethyl (C)[11]	Mascot
1984.8674	1984.8619	-0.0055	-3	226	242	GHYLNATAGTCEEMMKR			Carbamidomethyl (C)[11], Oxidation (M)[14]	Mascot
2000.8623	2000.8693	0.007	3	226	242	GHYLNATAGTCEEMMKR			Carbamidomethyl (C)[11], Oxidation (M)[14,15]	Mascot
2169.9871	2170.0059	0.0188	9	184	202	GGLDFTKDDENVNSQPFMR				Mascot
2169.9871	2170.0059	0.0188	9	184	202	GGLDFTKDDENVNSQPFMR	147	100		Mascot
2185.9819	2185.9934	0.0115	5	184	202	GGLDFTKDDENVNSQPF			Oxidation (M)[18]	Mascot

Calc. Mass	Obsrv. Mass	± da	± ppm	Start Seq.	End Seq.	Sequence	Ion Score	C. I. %	Modification	Rank	Result Type
						MR					
2410.1814	2410.176	-0.0054	-2	11	30	LTYYTPEYETKDTDILAAF R					Mascot
2994.4238	2994.4666	0.0428	14	248	274	ELGAPIVMHDYLTGGFTA NTSLAHYCR			Carbamidomethyl (C)[26]		Mascot
3010.4187	3010.4617	0.043	14	248	274	ELGAPIVMHDYLTGGFTA NTSLAHYCR			Carbamidomethyl (C)[26], Oxidation (M)[8]		Mascot

76

hypothetical protein Csa_2G351700 [Cucumis sativus]	gi|700207270	#######	6.62	35	656	100	483	100

Protein Group

PREDICTED: glycine dehydrogenase (decarboxylating), mitochondrial [Cucumis sativus]	gi|449450349	#######	6.6199 998855 5908

Peptide Information

Calc. Mass	Obsrv. Mass	± da	± ppm	Start Seq.	End Seq.	Sequence	Ion Score	C. I. %	Modification	Rank	Result Type
850.4206	850.4391	0.0185	22	1011	1017	FWPSTGR					Mascot
925.5465	925.5627	0.0162	18	534	542	SPIPSGLVR					Mascot
937.4374	937.45	0.0126	13	1018	1025	VDNVYGDR					Mascot
1094.5663	1094.5823	0.016	15	949	957	FCDALISIR			Carbamidomethyl (C)[2]		Mascot
1094.5663	1094.5823	0.016	15	949	957	FCDALISIR	48	99.652	Carbamidomethyl (C)[2]		Mascot
1221.666	1221.6523	-0.0137	-11	862	872	IAILNANYMAK					Mascot
1239.7208	1239.733	0.0122	10	438	450	VHGLAGVFAAGLK					Mascot
1239.7208	1239.733	0.0122	10	438	450	VHGLAGVFAAGLK	109	100			Mascot
1287.6844	1287.7012	0.0168	13	874	883	LENHYPVLFR					Mascot
1287.6844	1287.7012	0.0168	13	874	883	LENHYPVLFR	28	66.115			Mascot
1310.6528	1310.6699	0.0171	13	997	1007	EYAAFPASWLR					Mascot
1310.6528	1310.6699	0.0171	13	997	1007	EYAAFPASWLR	76	100			Mascot
1315.6641	1315.6815	0.0174	13	470	482	VADAHAIADAAYK					Mascot
1367.8158	1367.7507	-0.0651	-48	438	451	VHGLAGVFAAGLKK					Mascot
1443.7855	1443.7999	0.0144	10	873	883	RLENHYPVLFR					Mascot
1443.7855	1443.7999	0.0144	10	873	883	RLENHYPVLFR	27	57.926			Mascot

1498.8588	1498.8792	0.0204	14	375	389	IIGVSVDSSGKPALR				Mascot
1640.8755	1640.8906	0.0151	9	884	898	GVNGTVAHEFIIDLR				Mascot
1721.9109	1721.9115	0.0006	0	452	467	LGTAEVQGLPFFDTVK				Mascot
1838.9042	1838.9171	0.0129	7	782	800	TFCIPHGGGGPGMGPIGVK			Carbamidomethyl (C)[3]	Mascot
1850.0059	1850.02	0.0141	8	451	467	KLGTAEVQGLPFFDTVK				Mascot
1854.899	1854.9006	0.0016	1	782	800	TFCIPHGGGGPGMGPIGVK			Carbamidomethyl (C)[3], Oxidation (M)[13]	Mascot

1949.8953	1949.9097	0.0144	7	572	588	DLSLCHSMIPLGSCTMK			Carbamidomethyl (C)[5,14]	Mascot
1965.8901	1965.8953	0.0052	3	572	588	DLSLCHSMIPLGSCTMK			Carbamidomethyl (C)[5,14], Oxidation (M)[8]	Mascot
2090.0779	2090.1042	0.0263	13	157	174	SYIGMGYYNTFVPPVILR				Mascot
2090.0779	2090.1042	0.0263	13	157	174	SYIGMGYYNTFVPPVILR	21	0		Mascot
2106.073	2106.0925	0.0195	9	157	174	SYIGMGYYNTFVPPVILR			Oxidation (M)[5]	Mascot
2154.0093	2154.0303	0.021	10	101	120	MAEVCGFDSLDSLVDATVPK			Carbamidomethyl (C)[5]	Mascot
2170.0044	2170.0039	-0.0005	0	101	120	MAEVCGFDSLDSLVDATVPK			Carbamidomethyl (C)[5], Oxidation (M)[1]	Mascot
2181.0911	2181.0955	0.0044	2	977	996	GAPHPPSLLMGDAWTKPYSR				Mascot
2197.0859	2197.0962	0.0103	5	977	996	GAPHPPSLLMGDAWTKPYSR			Oxidation (M)[10]	Mascot
2218.0745	2218.0713	-0.0032	-1	245	263	TFVISNNCHPQTIDICVTR			Carbamidomethyl (C)[8]	Mascot
2233.1792	2233.0876	-0.0916	-41	375	395	IIGVSVDSSGKPALRMAMQTR			Oxidation (M)[16]	Mascot
2247.2285	2247.2454	0.0169	8	802	823	HLAPFLPSHPVVPTGGIPAPDK				Mascot
2275.0959	2275.1226	0.0267	12	245	263	TFVISNNCHPQTIDICVTR			Carbamidomethyl (C)[8,16]	Mascot
2275.0959	2275.1226	0.0267	12	245	263	TFVISNNCHPQTIDICVTR	60	99.975	Carbamidomethyl (C)[8,16]	Mascot
2292.0635	2292.0896	0.0261	11	132	151	FDEGLTESQMIEHMQNLAAK				Mascot
2308.0583	2308.0742	0.0159	7	132	151	FDEGLTESQMIEHMQNLAAK			Oxidation (M)[10]	Mascot
2328.1118	2328.135	0.0232	10	347	368	FGVPMGYGGPHAAFLATSQEYK				Mascot

2344.1067	2344.1238	0.0171	7	347	368	FGVPMGYGGPHAAFLAT SQEYK			Oxidation (M)[5]	Mascot	
2375.3235	2375.3367	0.0132	6	801	823	KHLAPFLPSHPVVPTGGI PAPDK				Mascot	
2401.113	2401.1426	0.0296	12	669	691	DVCIIPLSAHGTNPASAA MCGMK			Carbamidomethyl (C)[3,20]	Mascot	
2403.1909	2403.1602	-0.0307	-13	244	263	KTFVISNNCHPQTIDICVT R			Carbamidomethyl (C)[9,17]	Mascot	
2417.1082	2417.1335	0.0253	10	669	691	DVCIIPLSAHGTNPASAA MCGMK			Carbamidomethyl (C)[3,20], Oxidation (M)[19]	Mascot	
2484.2129	2484.2563	0.0434	17	347	369	FGVPMGYGGPHAAFLAT SQEYKR				Mascot	
2597.2783	2597.3169	0.0386	15	543	563	ESPYLTHPIFNTYHTEHE LLR				Mascot	
2597.2783	2597.3169	0.0386	15	543	563	ESPYLTHPIFNTYHTEHE LLR	114	100		Mascot	
2760.2722	2760.3198	0.0476	17	175	197	NIMENPAWYTQYTPYQA EISQGR				Mascot	
2776.2671	2776.3137	0.0466	17	175	197	NIMENPAWYTQYTPYQA EISQGR			Oxidation (M)[3]	Mascot	
2783.3313	2783.3418	0.0105	4	404	429	ATSNICTAQALLANMAAM YAVYHGPK			Carbamidomethyl (C)[6], Oxidation (M)[15]	Mascot	
2831.3445	2831.3728	0.0283	10	283	308	SGDVCGVLVQYPGTEGE VLDYGEFIK			Carbamidomethyl (C)[5]	Mascot	
2955.375	2955.426	0.051	17	717	742	ENLSALMVTYPSTHGVY EEGIDEICK			Carbamidomethyl (C)[25]	Mascot	
3005.6704	3005.7349	0.0645	21	317	346	VVMATDLLALTALKPPGE LGADIVVGSAQR				Mascot	
3021.6653	3021.7175	0.0522	17	317	346	VVMATDLLALTALKPPGE			Oxidation (M)[3]	Mascot	
**Project 1\Sample project201**	3021.7175									**558**	Mascot
						LGADIVVGSAQR					

Plastocyanin A, chloroplast [Cucumis sativus]	gi|700204935	17010.6	4.92	3	410	100	398	100

Protein Group

PREDICTED: plastocyanin [Cucumis sativus]	gi|449437172	17010.6	4.9200000762 9395

Peptide Information

Calc. Mass	Obsrv. Mass	± da	± ppm	Start Seq.	End Seq.	Sequence	Ion Score	C. I. %	Modification	Rank	Result Type
1875.8153	1875.8508	0.0355	19	145	162	GSYSFYCSPHQGAGMVGK					Mascot
1932.8368	1932.8583	0.0215	11	145	162	GSYSFYCSPHQGAGMVGK			Carbamidomethyl (C)[7]		Mascot
1932.8368	1932.8583	0.0215	11	145	162	GSYSFYCSPHQGAGMVGK	99	100	Carbamidomethyl (C)[7]		Mascot
1948.8317	1948.8461	0.0144	7	145	162	GSYSFYCSPHQGAGMVGK			Carbamidomethyl (C)[7], Oxidation (M)[15]		Mascot
1948.8317	1948.8461	0.0144	7	145	162	GSYSFYCSPHQGAGMVGK	100	100	Carbamidomethyl (C)[7], Oxidation (M)[15]		Mascot
2556.2002	2556.2371	0.0369	14	98	121	NNAGFPHNVVFDEDEIPSGVDVGK					Mascot
2556.2002	2556.2371	0.0369	14	98	121	NNAGFPHNVVFDEDEIPSGVDVGK	194	100			Mascot
2621.2651	2621.2896	0.0245	9	122	144	ISMDEENLLNAPGEVYEVQLTEK					Mascot
2621.2651	2621.2896	0.0245	9	122	144	ISMDEENLLNAPGEVYEVQLTEK	95	100			Mascot
2637.26	2637.2729	0.0129	5	122	144	ISMDEENLLNAPGEVYEVQLTEK			Oxidation (M)[3]		Mascot
2637.26	2637.2729	0.0129	5	122	144	ISMDEENLLNAPGEVYEVQLTEK	104	100	Oxidation (M)[3]		Mascot

of 584

of 584

of 584

of 584

of 584

of 584

of 584
