# Supplementary material for: Proteomic analysis of heat stress resistance of cucumber leaves when grafted onto Momordica rootstock
Source: Hortic Res. 2018 Oct 1;5:53. doi: 10.1038/s41438-018-0060-z (PMC6165847; doi:10.1038/s41438-018-0060-z)
Supplement: Supplementary file 2 — (momordica database) detailed match information for each gel spot [file 41438_2018_60_MOESM2_ESM.pdf]

detailed match information for each gel spot（momordica database）

Momordica Database

spot No.	Protein Name	Species	Accession No.	Protein MW	Protein PI	Pep. Count	Protein Score	Protein Score C. I. %	Total Ion Score	Total Ion C. I. %
1	calreticulin [Momordica charantia]		XP_022156394.1	48576.7	4.52	13	185	100	133	100

Peptide Information

Calc. Mass	Obsrv. Mass	± da	ppm	Start Seq.	End Seq.	Sequence	Ion Score	C. I. %	Modification	Rank	Result Type
826.4094	826.403	-0.0064	-8	26	31	VFFEER					Mascot
968.4221	968.5121	0.09	93	62	69	WHGDPNDK					Mascot
1011.4166	1011.5151	0.0985	97	32	39	FDDGWESR					Mascot
1016.4176	1016.4252	0.0076	7	108	116	LDCGGGYMK			Carbamidomethyl (C)[3], Oxidation (M)[8]		Mascot
1032.4633	1032.4504	-0.0129	-12	220	227	KPEDWDDK					Mascot
1068.4956	1068.4968	0.0012	1	70	78	GIQTSEDYR					Mascot
1068.4956	1068.4968	0.0012	1	70	78	GIQTSEDYR	24	69.478			Mascot
1465.7209	1465.7677	0.0468	32	307	318	DDPELYVYPNLK					Mascot
1691.8639	1691.8263	-0.0376	-22	197	211	QTGSLYSDWSLLPPK					Mascot
1819.9589	1819.9305	-0.0284	-16	197	212	QTGSLYSDWSLLPPKK					Mascot
1997.9703	1997.9635	-0.0068	-3	329	346	SGTLFDNVLITDDPEYAK					Mascot
1997.9703	1997.9635	-0.0068	-3	329	346	SGTLFDNVLITDDPEYAK	109	100			Mascot
2343.0308	2343.0522	0.0214	9	127	147	FGGETPYSIMFGPDICGY STK			Carbamidomethyl (C)[16], Oxidation (M)[10]		Mascot
2471.1257	2471.146	0.0203	8	127	148	FGGETPYSIMFGPDICGY STKK			Carbamidomethyl (C)[16], Oxidation (M)[10]		Mascot
3779.8838	3779.9805	0.0967	26	165	196	EVPCETDQLTHVYTFIIRP DATYSILIDNVEK			Carbamidomethyl (C)[4]		Mascot

spot No.	Protein Name	Species	Accession No.	Protein MW	Protein PI	Pep. Count	Protein Score	Protein Score C. I. %	Total Ion Score	Total Ion C. I. %
2	stromal 70 kDa heat shock-related protein, chloroplastic [Momordica charantia]		XP_022154567.1	75179.1	5.26	34	774	100	585	100

Peptide Information

Calc. Mass	Obsrv. Mass	± da	ppm	Start Seq.	End Seq.	Sequence	Ion Score	C. I. %	Modification	Rank	Result Type
869.5091	869.5036	-0.0055	-6	532	539	GVPQIEVK					Mascot
870.5407	870.5431	0.0024	3	229	236	IAGLEVLR					Mascot
884.5676	884.5381	-0.0295	-33	118	125	LVGQIAKR					Mascot
887.4655	887.4686	0.0031	3	170	177	LECPAIGK			Carbamidomethyl (C)[3]		Mascot
902.4829	902.4713	-0.0116	-13	311	318	DEGIDLLK					Mascot
915.4894	915.486	-0.0034	-4	382	389	TPVENSLR					Mascot
935.5309	935.5272	-0.0037	-4	523	531	LDGIPPAPR					Mascot
970.5316	970.5325	0.0009	1	359	366	HIETTITR					Mascot
996.6088	996.6006	-0.0082	-8	410	418	IPAVQELVK					Mascot
1066.578	1066.5394	-0.0386	-36	104	113	TTPSVVAYTK					Mascot
1125.5609	1125.5648	0.0039	3	579	588	MVSEADKFAK					Mascot
1138.6467	1138.6162	-0.0305	-27	616	626	ELGDKVPGPVK					Mascot
1141.5558	1141.5576	0.0018	2	579	588	MVSEADKFAK			Oxidation (M)[1]		Mascot
1156.6685	1156.6686	0.0001	0	380	389	LKTPVENSLR					Mascot
1170.6589	1170.6273	-0.0316	-27	114	124	NGDRLVGQIAK					Mascot
1176.6412	1176.6232	-0.018	-15	300	309	IVDWLAANFK					Mascot
1197.5304	1197.6011	0.0707	59	145	154	KMSEVDEESK			Oxidation (M)[2]		Mascot
1364.7057	1364.7321	0.0264	19	192	203	LVDDASKFLNDK					Mascot
1373.7272	1373.7292	0.002	1	397	409	DIDEVILVGGSTR					Mascot
1373.7272	1373.7292	0.002	1	397	409	DIDEVILVGGSTR	131	100			Mascot
1381.6594	1381.6488	-0.0106	-8	601	612	NQADSVVYQTEK					Mascot
1387.754	1387.7264	-0.0276	-20	321	332	QALQRLTETAEK					Mascot
1396.6414	1396.6608	0.0194	14	369	379	FEELCSDLLDR			Carbamidomethyl (C)[5]		Mascot
1415.7166	1415.7318	0.0152	11	38	51	LGNSSGFSSATFLK					Mascot
1461.7697	1461.7676	-0.0021	-1	178	190	QFAAEEISAQVLR					Mascot
1461.7697	1461.7676	-0.0021	-1	178	190	QFAAEEISAQVLR	129	100			Mascot

1565.6967	1565.7789	0.0822	53	690	704	GPEGDVIDADFTDSK					Mascot
1566.7911	1566.7914	0.0003	0	207	220	AVVTVPAYFNDSQR					Mascot
1566.7911	1566.7914	0.0003	0	207	220	AVVTVPAYFNDSQR	87	100			Mascot
1579.8115	1579.7915	-0.02	-13	126	139	QAVVNPENTFFSVK					Mascot
1579.8115	1579.7915	-0.02	-13	126	139	QAVVNPENTFFSVK	46	99.743			Mascot

1589.8646	1589.767	-0.0976	-61	178	191	QFAAEEISAQVLRK					Mascot
1595.7734	1595.7772	0.0038	2	367	379	AKFEELCSDLLDR			Carbamidomethyl (C)[7]		Mascot
1595.7734	1595.7772	0.0038	2	367	379	AKFEELCSDLLDR	29	88.321	Carbamidomethyl (C)[7]		Mascot
1723.8901	1723.8685	-0.0216	-13	237	252	IINEPTAASLAYGFEK					Mascot
1723.8901	1723.8685	-0.0216	-13	237	252	IINEPTAASLAYGFEK	97	100			Mascot
1735.9126	1735.9193	0.0067	4	126	140	QAVVNPENTFFSVKR					Mascot
1931.9557	1931.9467	-0.009	-5	561	578	QDITITGASTLPSDEVER					Mascot
2060.0505	2060.0542	0.0037	2	560	578	KQDITITGASTLPSDEVER					Mascot
2060.0505	2060.0542	0.0037	2	560	578	KQDITITGASTLPSDEVER	53	99.952			Mascot
2437.1841	2437.2068	0.0227	9	487	509	SEVFSTAADGQTSVEINV LQGER					Mascot
2437.1841	2437.2068	0.0227	9	487	509	SEVFSTAADGQTSVEINV LQGER	13	0			Mascot
3056.5681	3056.6094	0.0413	14	73	103	VVGIDLGTTNSAVAAMEG GKPTIVTNAEGQR					Mascot
3072.563	3072.5989	0.0359	12	73	103	VVGIDLGTTNSAVAAMEG GKPTIVTNAEGQR			Oxidation (M)[16]		Mascot

3

protein disulfide-isomerase-like [Momordica charantia] XP_022141570.1 56222.3 4.85 10 76 99.93 52 99.934

Peptide Information

Calc. Mass	Obsrv. Mass	± da	ppm	Start Seq.	End Seq.	Sequence	Ion Score	C. I. %	Modification	Rank	Result Type
870.4931	870.5341	0.041	47	425	432	DPDVIIAK					Mascot
1037.5011	1037.4669	-0.0342	-33	122	130	SSQDYKGPR					Mascot
1047.4702	1047.4857	0.0155	15	91	99	IDANEESNR					Mascot
1088.5582	1088.5682	0.01	9	141	151	TQSGPASAEIK					Mascot
1088.5582	1088.5682	0.01	9	141	151	TQSGPASAEIK					Mascot
1382.691	1382.5966	-0.0944	-68	457	469	TASGKLVEYSGDR					Mascot
1382.691	1382.5966	-0.0944	-68	457	469	TASGKLVEYSGDR					Mascot
1564.7966	1564.759	-0.0376	-24	470	482	SKEDIINFVETNR					Mascot
1584.7792	1584.7506	-0.0286	-18	411	424	LAPTYEEVGVSYEK					Mascot
1592.7915	1592.8083	0.0168	11	472	484	EDIINFVETNRDK					Mascot
1659.8418	1659.8221	-0.0197	-12	216	228	LFKPFDEQFVDFK					Mascot
1659.8418	1659.8221	-0.0197	-12	216	228	LFKPFDEQFVDFK	52	99.934			Mascot

1821.8728	1821.8781	0.0053	3	271	286	AMFFLNFSSEVADSLK			Oxidation (M)[2]		Mascot

4

ruBisCO large subunit-binding protein subunit alpha [Momordica charantia]	XP_022140442.1	61295.7	5.06	22	380	100	282	100

Peptide Information

Calc. Mass	Obsrv. Mass	± da	ppm	Start Seq.	End Seq.	Sequence	Ion Score	C. I. %	Modification	Rank	Result Type
857.5091	857.5034	-0.0057	-7	482	489	LGADIIQK					Mascot
898.572	898.5627	-0.0093	-10	311	319	GILNVAAIK					Mascot
940.5713	940.5544	-0.0169	-18	274	281	DIIPLLEK					Mascot
1033.5386	1033.4683	-0.0703	-68	22	30	KVNQSQSSR					Mascot
1043.5845	1043.5795	-0.005	-5	91	100	VVNDGVTIAR					Mascot
1052.5007	1052.4911	-0.0096	-9	48	56	EIAFDQSSR					Mascot
1052.5007	1052.4911	-0.0096	-9	48	56	EIAFDQSSR	24	68.678			Mascot
1076.5106	1076.4817	-0.0289	-27	473	481	DKLEDAEEK					Mascot
1181.7001	1181.6901	-0.01	-8	66	77	LANAVGLTLGPR					Mascot
1181.7001	1181.6901	-0.01	-8	66	77	LANAVGLTLGPR	44	99.694			Mascot
1204.6208	1204.6017	-0.0191	-16	80	90	NVVLDEFGSPK					Mascot
1290.6172	1290.6205	0.0033	3	423	434	VGAATETELEDR					Mascot
1372.7318	1372.7136	-0.0182	-13	165	176	TVQGLIEELENK					Mascot
1418.7122	1418.6953	-0.0169	-12	423	435	VGAATETELEDRK					Mascot
1479.7479	1479.7203	-0.0276	-19	240	252	GYISPQFVTNPEK					Mascot
1479.7479	1479.7203	-0.0276	-19	240	252	GYISPQFVTNPEK	86	100			Mascot
1499.6748	1499.6497	-0.0251	-17	394	406	ELAETDSVYDTEK					Mascot
1517.7847	1517.7446	-0.0401	-26	529	542	YENLVEAGVIDPAK					Mascot
1636.7773	1636.7859	0.0086	5	123	139	TNDSAGDGTTTASVLAR					Mascot
1671.88	1671.9009	0.0209	13	475	489	LEDAEEKLGADIIQK					Mascot
1918.9717	1918.9612	-0.0105	-5	370	387	DTTTIIADAASKDEIQAR					Mascot
1918.9717	1918.9612	-0.0105	-5	370	387	DTTTIIADAASKDEIQAR	127	100			Mascot
2225.1013	2225.1047	0.0034	2	1	21	MASANAISSASILCSSQK NLR			Carbamidomethyl (C)[14], Oxidation (M)[1]		Mascot
2250.2957	2250.2429	-0.0528	-23	287	308	APLLIIAEDVTGEALATLV VNK					Mascot
2277.1279	2277.1418	0.0139	6	188	210	AVASISAGNDELIGSMIAD			Oxidation (M)[16]		Mascot

Calc. Mass	Obsrv. Mass	± da	ppm	Start Seq.	End Seq.	Sequence	Ion Score	C. I. %	Modification	Rank	Result Type
						AIDK					
3056.4253	3056.4309	0.0056	2	211	239	VGPDGVLSIESSSSFETT			Oxidation (M)[25]		Mascot

Project 1\Sample proje 3056.4309 of Mascot **76**

VEVEEGMAIDR

5

ribulose-1,5-bisphosphate carboxylase/oxygenase large subunit, partial (chloroplast) [Momordica charantia] ABG24929.1 46428.5 6.31 13 48 58.046

Peptide Information

Calc. Mass	Obsrv. Mass	± da	ppm	Start Seq.	End Seq.	Sequence	Ion Score	C. I. %	Modification	Rank	Result Type
830.4366	830.394	-0.0426	-51	395	401	EGNEIIR					Mascot
853.4236	853.3473	-0.0763	-89	143	149	AVYECLR					Mascot
910.4451	910.4123	-0.0328	-36	143	149	AVYECLR			Carbamidomethyl (C)[5]		Mascot
914.405	914.3726	-0.0324	-35	261	267	NHGMHFR			Oxidation (M)[4]		Mascot
928.4669	928.4238	-0.0431	-46	251	258	AMHAVIDR			Oxidation (M)[2]		Mascot
962.4789	962.4338	-0.0451	-47	183	191	SQAETGEIK					Mascot
1116.583	1116.5524	-0.0306	-27	377	386	VALEACVQAR			Carbamidomethyl (C)[6]		Mascot
1170.5586	1170.6099	0.0513	44	259	267	QKNHGMHFR			Oxidation (M)[6]		Mascot
1187.6644	1187.6294	-0.035	-29	241	250	DNGLLLHIHR					Mascot
1188.6122	1188.63	0.0178	15	173	182	FLFCAEAIFK					Mascot
1245.6337	1245.5878	-0.0459	-37	173	182	FLFCAEAIFK			Carbamidomethyl (C)[4]		Mascot
1309.6947	1309.5863	-0.1084	-83	261	271	NHGMHFRVLAK					Mascot
1465.7546	1465.7258	-0.0288	-20	102	114	TFQGPPHGIQVER					Mascot
1465.7546	1465.7258	-0.0288	-20	102	114	TFQGPPHGIQVER	1	0			Mascot
1489.7145	1489.6053	-0.1092	-73	406	418	WSPELAAACEVWK					Mascot
2185.9819	2185.9419	-0.04	-18	150	168	GGLDFTKDDENVNSQPF MR			Oxidation (M)[18]		Mascot

6

uncharacterized protein LOC111007929 [Momordica charantia] XP_022136172.1 55447.3 9.6 14 47 36.5

Peptide Information

Calc. Mass	Obsrv. Mass	± da	ppm	Start Seq.	End Seq.	Sequence	Ion Score	C. I. %	Modification	Rank	Result Type
818.4618	818.4029	-0.0589	-72	31	38	TPTLSTAK					Mascot

827.3868	827.397	0.0102	12	369	374	TFMSWR					Mascot
830.4196	830.4115	-0.0081	-10	138	143	FPYTFR					Mascot
999.4828	999.4637	-0.0191	-19	369	375	TFMSWRR			Oxidation (M)[3]		Mascot
1005.584	1005.5869	0.0029	3	436	443	QIRVFSQK					Mascot
1118.559	1118.486	-0.073	-65	187	195	TLGKYDHER					Mascot
1138.5276	1138.5627	0.0351	31	244	253	YGHLYGSDAR					Mascot
1308.6399	1308.621	-0.0189	-14	421	432	MVTGCRASDAIK			Carbamidomethyl (C)[5]		Mascot
1316.6635	1316.5376	-0.1259	-96	384	393	YLFNTRDYPK					Mascot
1341.662	1341.6819	0.0199	15	328	337	IFQQIVCYDR			Carbamidomethyl (C)[7]		Mascot
1342.6598	1342.6106	-0.0492	-37	158	168	EVVERDEPDVR					Mascot
1415.6406	1415.6265	-0.0141	-10	456	466	RQCCDVISYSK			Carbamidomethyl (C)[3,4]		Mascot
1445.7305	1445.608	-0.1225	-85	444	455	LELDVEEMKAPR			Oxidation (M)[8]		Mascot
2281.1719	2281.1082	-0.0637	-28	57	77	VSPNAPSLLCSHSTLSPTTRR			Carbamidomethyl (C)[10]		Mascot

7

tulin beta chain-like [Momordica charantia] XP_022151524.1 50078.8 4.73 17 74 99.876

Peptide Information

Calc. Mass	Obsrv. Mass	± da	ppm	Start Seq.	End Seq.	Sequence	Ion Score	C. I. %	Modification	Rank	Result Type
1075.5242	1075.4612	-0.063	-59	310	318	YLTASAMFR			Oxidation (M)[7]		Mascot
1077.5323	1077.4976	-0.0347	-32	155	162	IREEYPDR					Mascot
1139.6936	1139.667	-0.0266	-23	253	262	LAVNLIPFPR					Mascot
1146.5902	1146.5605	-0.0297	-26	242	251	FPGQLNSDLR					Mascot
1231.5776	1231.5532	-0.0244	-20	381	390	VSEQFTAMFR			Oxidation (M)[8]		Mascot
1267.7886	1267.7488	-0.0398	-31	252	262	KLAVNLIPFPR					Mascot
1274.6852	1274.6101	-0.0751	-59	242	252	FPGQLNSDLRK					Mascot
1323.6176	1323.5953	-0.0223	-17	36	46	YQGDSDLQLER					Mascot
1384.7004	1384.6305	-0.0699	-50	163	174	MMLTFSVFPSPK					Mascot
1387.6787	1387.6199	-0.0588	-42	381	391	VSEQFTAMFRR			Oxidation (M)[8]		Mascot
1415.609	1415.6044	-0.0046	-3	298	309	NMMCAADPRHGR			Carbamidomethyl (C)[4]		Mascot
1416.6902	1416.6455	-0.0447	-32	163	174	MMLTFSVFPSPK			Oxidation (M)[1,2]		Mascot
1416.6902	1416.7635	0.0733	52	163	174	MMLTFSVFPSPK			Oxidation (M)[1,2]		Mascot
1431.6039	1431.5978	-0.0061	-4	298	309	NMMCAADPRHGR			Carbamidomethyl (C)[4], Oxidation (M)[2]		Mascot
1445.6478	1445.6139	-0.0339	-23	47	58	INVYYNEASCGR			Carbamidomethyl (C)[10]		Mascot
1638.8462	1638.777	-0.0692	-42	263	276	LHFFMVGFAPLTSR			Oxidation (M)[5]		Mascot

1663.803	1663.7819	-0.0211	-13	63	77	AVLMDLEPGTMDSIR			Oxidation (M)[4]		Mascot
1951.8889	1951.8207	-0.0682	-35	363	379	MASTFIGNSTSIQEMFR			Oxidation (M)[1,15]		Mascot
1972.9974	1972.9542	-0.0432	-22	104	121	GHYTEGAELIDSVLDVVR					Mascot
2809.2776	2809.168	-0.1096	-39	283	306	ALTVPELTQQMWDAKNM MCAADPR			Carbamidomethyl (C)[19], Oxidation (M)[11,17]		Mascot

8

methyl-CpG-binding domain-containing protein 5-like [Momordica charantia]	XP_022155116.1	27429	9.49	11	75	99.904	21	25.581

Peptide Information

Calc. Mass	Obsrv. Mass	± da	ppm	Start Seq.	End Seq.	Sequence	Ion Score	C. I. %	Modification	Rank	Result Type
1094.6317	1094.5308	-0.1009	-92	71	80	AEPLPLNGRK					Mascot
1173.6151	1173.6162	0.0011	1	210	219	VEWVLTDPSK					Mascot
1275.6844	1275.6945	0.0101	8	238	248	EWVGAFQLLGR					Mascot
1292.623	1292.6648	0.0418	32	54	65	TPNNPEPPSPSR					Mascot
1350.5842	1350.6044	0.0202	15	174	186	KDAMSGDGSEEPK					Mascot
1421.6213	1421.6895	0.0682	48	175	188	DAMSGDGSEEPKAK					Mascot
1431.7855	1431.6595	-0.126	-88	237	248	REWVGAFQLLGR					Mascot
1431.7855	1431.6595	-0.126	-88	237	248	REWVGAFQLLGR	21	25.581			Mascot
1448.7241	1448.7078	-0.0163	-11	54	66	TPNNPEPPSPSRR					Mascot
1518.6973	1518.6138	-0.0835	-55	2	15	SASGTSFPDPNWPR					Mascot
1553.8574	1553.7021	-0.1553	###	158	170	IEVLYFLETGTLR					Mascot
1553.8574	1553.7021	-0.1553	###	158	170	IEVLYFLETGTLR					Mascot
1756.8977	1756.9663	0.0686	39	81	98	LPPDAPAPGNGDSPAPR K					Mascot

9

ATP synthase subunit beta, mitochondrial [Momordica charantia]	XP_022150567.1	59910.3	5.84	25	820	100	678	100

Peptide Information

Calc. Mass	Obsrv. Mass	± da	ppm	Start Seq.	End Seq.	Sequence	Ion Score	C. I. %	Modification	Rank	Result Type
866.4003	866.3782	-0.0221	-26	270	276	EGNDLYR					Mascot
975.5622	975.5383	-0.0239	-24	230	240	IGLFGGAGVGK					Mascot

1005.5285	1005.5657	0.0372	37	277	285	EMIESGVIK				Mascot
1021.5234	1021.488	-0.0354	-35	277	285	EMIESGVIK			Oxidation (M)[2]	Mascot
1173.6627	1173.635	-0.0277	-24	217	226	VVDLLAPYQR				Mascot
1173.6627	1173.635	-0.0277	-24	217	226	VVDLLAPYQR	64	99.996		Mascot
1278.6359	1278.6239	-0.012	-9	138	149	TIAMDGTEGLVR			Oxidation (M)[4]	Mascot
1278.6359	1278.7483	0.1124	88	138	149	TIAMDGTEGLVR			Oxidation (M)[4]	Mascot
1367.7529	1367.7256	-0.0273	-20	172	183	IINVIGEPIDER				Mascot
1380.7006	1380.6578	-0.0428	-31	516	528	ESITSFQGVLDGK				Mascot
1382.7135	1382.7172	0.0037	3	18	30	ASSRSPLSNPNPR				Mascot
1390.6863	1390.6575	-0.0288	-21	254	267	AHGGFSVFAGVGER				Mascot
1390.6863	1390.6575	-0.0288	-21	254	267	AHGGFSVFAGVGER	124	100		Mascot
1399.7693	1399.7395	-0.0298	-21	312	324	VGLTGLTVAEHFR				Mascot
1399.7693	1399.7395	-0.0298	-21	312	324	VGLTGLTVAEHFR	102	100		Mascot
1409.8112	1409.7817	-0.0295	-21	153	166	VLNTGSPITVPVGR				Mascot
1409.8112	1409.7817	-0.0295	-21	153	166	VLNTGSPITVPVGR	71	100		Mascot
1457.8396	1457.7526	-0.087	-60	241	253	TVLIMELINNVAK				Mascot
1473.8346	1473.7496	-0.085	-58	241	253	TVLIMELINNVAK			Oxidation (M)[5]	Mascot
1492.7755	1492.7417	-0.0338	-23	341	354	FTQANSEVSALLGR				Mascot
1492.7755	1492.7417	-0.0338	-23	341	354	FTQANSEVSALLGR	35	96.998		Mascot
1621.7462	1621.7523	0.0061	4	295	309	CALVYGQMNEPPGAR			Oxidation (M)[8]	Mascot
1678.7676	1678.718	-0.0496	-30	295	309	CALVYGQMNEPPGAR			Carbamidomethyl (C)[1], Oxidation (M)[8]	Mascot
1678.7676	1678.718	-0.0496	-30	295	309	CALVYGQMNEPPGAR			Carbamidomethyl (C)[1], Oxidation (M)[8]	Mascot
1707.921	1707.8774	-0.0436	-26	123	137	LVLEVAQHLGENMVR				Mascot
1723.916	1723.8597	-0.0563	-33	123	137	LVLEVAQHLGENMVR			Oxidation (M)[13]	Mascot

1723.916	1723.8597	-0.0563	-33	123	137	LVLEVAQHLGENMVR			Oxidation (M)[13]	Mascot
1864.944	1864.9055	-0.0385	-21	325	340	DAEGQDVLLFIDNIFR				Mascot
1864.944	1864.9055	-0.0385	-21	325	340	DAEGQDVLLFIDNIFR	117	100		Mascot
1868.9059	1868.9335	0.0276	15	270	285	EGNDLYREMIESGVIK			Oxidation (M)[9]	Mascot
2013.054	2012.9679	-0.0861	-43	511	528	YVELKESITSFQGVLDGK				Mascot
2061.0498	2060.9963	-0.0535	-26	418	436	QISELGIYPAVDPLDSTSR				Mascot
2172.1548	2172.0808	-0.074	-34	197	216	EAPAFVEQATEQQILVTG IK				Mascot
2186.1453	2186.1045	-0.0408	-19	355	375	IPSAVGYQPTLATDLGGL QER				Mascot

2186.1453	2186.1045	-0.0408	-19	355	375	IPSAVGYQPTLATDLGGL QER	167	100			Mascot
2208.0173	2208.0503	0.033	15	290	309	QADSKCALVYGQMNEPP GAR			Carbamidomethyl (C)[6], Oxidation (M)[13]		Mascot
2688.376	2688.3176	-0.0584	-22	463	486	NLQDIIAILGMDELSEDDK LTVAR			Oxidation (M)[11]		Mascot
3714.8862	3714.8528	-0.0334	-9	382	417	GSITSVQAIYVPADDLTD PAPATTFAHLDATTVLSR					Mascot
3842.9812	3842.9358	-0.0454	-12	381	417	KGSITSVQAIYVPADDLT DPAPATTFAHLDATTVLS R					Mascot

10	ruBisCO large subunit-binding protein subunit beta, chloroplastic [Momordica charantia]	XP_022147736.1	64774	5.68	22	590	100	496	100

Peptide Information

Calc. Mass	Obsrv. Mass	± da	± ppm	Start Seq.	End Seq.	Sequence	Ion Score	C. I. %	Modification	Rank	Result Type
953.4686	953.436	-0.0326	-34	533	540	VLSSDNYR					Mascot
959.5156	959.4839	-0.0317	-33	235	243	GVVTLEEGR					Mascot
1087.6106	1087.5814	-0.0292	-27	374	384	AGKEVLGQASK					Mascot
1159.4758	1159.418	-0.0578	-50	273	281	MSVEYENCK			Carbamidomethyl (C)[8]		Mascot
1182.7094	1182.6649	-0.0445	-38	86	97	LADLVGVTLGPK					Mascot
1229.6848	1229.6051	-0.0797	-65	456	466	LRVEDALNATK					Mascot
1245.5715	1245.507	-0.0645	-52	567	577	CCLEHAASVAK			Carbamidomethyl (C)[1,2]		Mascot
1280.7686	1280.724	-0.0446	-35	169	181	VVAAGANPVLITR					Mascot
1280.7686	1280.724	-0.0446	-35	169	181	VVAAGANPVLITR	76	100			Mascot
1298.7314	1298.6838	-0.0476	-37	294	304	DLINILEEAIR					Mascot
1505.7159	1505.6472	-0.0687	-46	260	272	GYISPYFVTDSEK					Mascot
1505.7159	1505.6472	-0.0687	-46	260	272	GYISPYFVTDSEK	96	100			Mascot
1527.7538	1527.6616	-0.0922	-60	122	135	EVELDDPVENIGAK					Mascot
1540.775	1540.7169	-0.0581	-38	578	590	TFLMSDCVVVEIK			Carbamidomethyl (C)[7]		Mascot
1556.7699	1556.7042	-0.0657	-42	578	590	TFLMSDCVVVEIK			Carbamidomethyl (C)[7], Oxidation (M)[4]		Mascot
1643.8785	1643.8136	-0.0649	-39	467	483	AAVEEGIVVGGGCTLLR					Mascot
1700.9	1700.8472	-0.0528	-31	467	483	AAVEEGIVVGGGCTLLR			Carbamidomethyl (C)[13]		Mascot

Calc. Mass	Obsrv. Mass	± da	ppm	Start Seq.	End Seq.	Sequence	Ion Score	C. I. %	Modification	Rank	Result Type
1700.9	1700.8472	-0.0528	-31	467	483	AAVEEGIVVGGGCTLLR	126	100	Carbamidomethyl (C)[13]		Mascot
1856.9133	1856.8906	-0.0227	-12	273	287	MSVEYENCKLLLVDK			Carbamidomethyl (C)[8], Oxidation (M)[1]		Mascot
1896.0073	1895.8215	-0.1858	-98	122	138	EVELDDPVENIGAKLVR					Mascot
1900.0386	1899.9551	-0.0835	-44	434	452	LSGGVAVIQVGAQTETEL K					Mascot
1904.8848	1904.8483	-0.0365	-19	244	259	SAENFLYVVEGMQFDR					Mascot
1928.9205	1928.7682	-0.1523	-79	1	20	MASSFTAMSSVGTLAAP GSR					Mascot
1948.0022	1947.8252	-0.177	-91	408	424	VAQIKNLIEAADQDYEK					Mascot
2157.1763	2157.0811	-0.0952	-44	434	454	LSGGVAVIQVGAQTETEL KEK					Mascot
2430.2722	2430.1772	-0.095	-39	144	168	TNDLAGDGTTTSVVLAQ					Mascot

Calc. Mass	Obsrv. Mass	± da	ppm	Start Seq.	End Seq.	Sequence	Ion Score	C. I. %	Modification	Rank	Result Type
						GLIAEGVK					
2430.2722	2430.1772	-0.095	-39	144	168	TNDLAGDGTTTSVVLAQ GLIAEGVK	198	100			Mascot
2448.1387	2448.0569	-0.0818	-33	541	563	YGYNAATGNYEDLMAAG IIDPTK					Mascot
2464.1338	2464.0354	-0.0984	-40	541	563	YGYNAATGNYEDLMAAG IIDPTK			Oxidation (M)[14]		Mascot

11

luminal-binding protein 5 [Momordica charantia]	XP_022145904.1	73369.9	5.07	26	593	100	100

Peptide Information

Calc. Mass	Obsrv. Mass	± da	ppm	Start Seq.	End Seq.	Sequence	Ion Score	C. I. %	Modification	Rank	Result Type
943.4957	943.4883	-0.0074	-8	269	275	IMEYFIK					Mascot
959.4907	959.4833	-0.0074	-8	269	275	IMEYFIK			Oxidation (M)[2]		Mascot
965.4938	965.4792	-0.0146	-15	162	170	ETAEAFLGK					Mascot
989.4897	989.4858	-0.0039	-4	542	549	LSQEEIDR					Mascot
998.5013	998.4965	-0.0048	-5	89	97	NQAAVNPER					Mascot
1047.5834	1047.5679	-0.0155	-15	130	138	DGKPYIQVK					Mascot
1169.6637	1169.6622	-0.0015	-1	193	204	DAGIIAGLNVAR					Mascot
1183.647	1183.6432	-0.0038	-3	491	501	FDLTGIPPAPR					Mascot
1183.647	1183.6432	-0.0038	-3	491	501	FDLTGIPPAPR	74	100			Mascot

1224.6293	1224.6139	-0.0154	-13	160	170	MKETAEAFLGK				Mascot
1240.6243	1240.6134	-0.0109	-9	160	170	MKETAEAFLGK			Oxidation (M)[1]	Mascot
1296.6219	1296.6161	-0.0058	-4	334	343	FEELNNDLFR				Mascot
1296.6219	1296.6161	-0.0058	-4	334	343	FEELNNDLFR	85	100		Mascot
1379.6147	1379.6064	-0.0083	-6	572	582	NSLETYTYNMK			Oxidation (M)[10]	Mascot
1453.7686	1453.7145	-0.0541	-37	378	389	VQQLLKDYFDGK				Mascot
1500.8018	1500.7975	-0.0043	-3	361	374	NQIDEIVLVGGSTR				Mascot

1500.8018	1500.7975	-0.0043	-3	361	374	NQIDEIVLVGGSTR	120	100		Mascot
1508.738	1508.7322	-0.0058	-4	69	81	ITPSWVAFTDSER				Mascot
1508.738	1508.7322	-0.0058	-4	69	81	ITPSWVAFTDSER	36	97.596		Mascot
1523.7601	1523.7483	-0.0118	-8	332	343	ARFEELNNDLFR				Mascot
1523.7601	1523.7483	-0.0118	-8	332	343	ARFEELNNDLFR	20	2.949		Mascot
1536.7513	1536.7419	-0.0094	-6	55	68	NGHVEIIANDQGNR				Mascot
1550.8135	1550.7773	-0.0362	-23	146	159	VFSPEEISAMVLTK				Mascot
1554.6628	1554.7314	0.0686	44	550	562	MVQEAEEFAEEDK				Mascot
1566.8083	1566.7618	-0.0465	-30	146	159	VFSPEEISAMVLTK			Oxidation (M)[10]	Mascot
1595.8905	1595.7754	-0.1151	-72	487	501	NLGKFDLTGIPPAPR				Mascot
1659.8953	1659.8585	-0.0368	-22	205	220	IINEPTAAAIAYGLDK				Mascot
1665.8231	1665.8118	-0.0113	-7	174	188	DAVVTVPAYFNDAQR				Mascot
1665.8231	1665.8118	-0.0113	-7	174	188	DAVVTVPAYFNDAQR	44	99.588		Mascot
1680.9027	1680.8186	-0.0841	-50	82	97	LIGEAAKNQAAVNPER				Mascot
1787.9901	1787.9526	-0.0375	-21	205	221	IINEPTAAAIAYGLDKK				Mascot
1875.9634	1875.9462	-0.0172	-9	631	646	ELEAVCNPIITAVYQR			Carbamidomethyl (C)[6]	Mascot
1907.0022	1906.9874	-0.0148	-8	172	188	IKDAVVTVPAYFNDAQR				Mascot
1907.0022	1906.9874	-0.0148	-8	172	188	IKDAVVTVPAYFNDAQR	95	100		Mascot
2427.0471	2427.0098	-0.0373	-15	609	628	DALEWLDDNQSAEKEDYEEK				Mascot
2691.2898	2691.2937	0.0039	1	457	479	SQVFTTYQDQQTTVSIQVFEGER				Mascot

12	elongation factor G-2, chloroplastic [Momordica charantia]	XP_022156454.1	86198	5.48	21	276	100		100

Peptide Information

Calc. Mass	Obsrv. Mass	± da	ppm	Start Seq.	End Seq.	Sequence	Ion Score	C. I. %	Modification	Rank	Result Type
824.4413	824.4523	0.011	13	225	231	LGANFFR					Mascot
885.4828	885.4999	0.0171	19	115	121	ILYYTGR					Mascot
904.5138	904.5259	0.0121	13	622	629	EYIPGVVK					Mascot
978.4891	978.5006	0.0115	12	89	96	SVPLEDYR					Mascot
1086.5361	1086.5156	-0.0205	-19	441	449	LLEMHANSR			Oxidation (M)[4]		Mascot
1134.5902	1134.6261	0.0359	32	88	96	RSVPLEDYR					Mascot
1141.5493	1141.519	-0.0303	-27	216	224	ICFVNKMDR			Oxidation (M)[7]		Mascot
1255.6464	1255.6274	-0.019	-15	97	108	NIGIMAHIDAGK			Oxidation (M)[5]		Mascot
1310.8042	1310.8031	-0.0011	-1	454	467	VALAGDIVALAGLK					Mascot
1416.723	1416.7332	0.0102	7	557	569	VEANVGAPQVNYR					Mascot
1416.723	1416.7332	0.0102	7	557	569	VEANVGAPQVNYR	45	99.679			Mascot
1433.6809	1433.6921	0.0112	8	516	527	LAQEDPSFHFSR					Mascot
1433.6809	1433.6921	0.0112	8	516	527	LAQEDPSFHFSR	9	0			Mascot
1456.6849	1456.6835	-0.0014	-1	57	69	FSSLASSQLCNSR			Carbamidomethyl (C)[10]		Mascot
1463.7238	1463.7335	0.0097	7	585	598	QSGGQGQFADITVR					Mascot
1463.7238	1463.7335	0.0097	7	585	598	QSGGQGQFADITVR	65	99.997			Mascot
1573.8446	1573.8484	0.0038	2	713	727	RGQINSFGDKPGGLK					Mascot
1573.8446	1573.8484	0.0038	2	713	727	RGQINSFGDKPGGLK					Mascot
1591.8187	1591.821	0.0023	1	584	598	KQSGGQGQFADITVR					Mascot
1820.929	1820.9363	0.0073	4	554	569	EFKVEANVGAPQVNYR					Mascot
2068.071	2068.0823	0.0113	5	162	179	INIIDTPGHVDFTLEVER					Mascot
2068.071	2068.0823	0.0113	5	162	179	INIIDTPGHVDFTLEVER	17	0			Mascot
2092.0203	2091.9946	-0.0257	-12	630	649	GLEECMSNGVLAGFPVV DVR					Mascot
2167.1467	2167.1304	-0.0163	-8	728	746	VVDALVPLAEMFQYVSTL R			Oxidation (M)[11]		Mascot
2178.1038	2178.1162	0.0124	6	693	712	VEVVTPEEHLGDVIGDLN SR					Mascot
2178.1038	2178.1162	0.0124	6	693	712	VEVVTPEEHLGDVIGDLN	76	100			Mascot

						SR					
2668.3135	2668.3228	0.0093	3	528	550	DEEINQTVIEGMGELHLEI			Oxidation (M)[12]		Mascot

IVDR

13

transketolase, chloroplastic [Momordica charantia]	XP_022147028.1	80665.8	6.15	18	104	100	99.859

Peptide Information

Calc. Mass	Obsrv. Mass	± da	ppm	Start Seq.	End Seq.	Sequence	Ion Score	C. I. %	Modification	Rank	Result Type
805.4202	805.4151	-0.0051	-6	716	724	FGASAPAGK					Mascot
904.3869	904.3965	0.0096	11	471	477	MYGDFQK			Oxidation (M)[1]		Mascot
996.4686	996.4788	0.0102	10	131	137	NPYWFNR					Mascot
996.4686	996.4788	0.0102	10	131	137	NPYWFNR	15	0			Mascot
1005.5615	1005.5681	0.0066	7	93	101	FLAIDAVEK					Mascot
1138.5123	1138.5247	0.0124	11	298	307	NGNTGYDEIR					Mascot
1193.5433	1193.558	0.0147	12	427	437	YTPESPGDATR					Mascot
1193.5433	1193.558	0.0147	12	427	437	YTPESPGDATR	33	95.894			Mascot
1221.6475	1221.6342	-0.0133	-11	325	336	VTTTIGFGSPNK					Mascot
1359.705	1359.6959	-0.0091	-7	438	449	NLSQQCLNALAK			Carbamidomethyl (C)[6]		Mascot
1361.6808	1361.6808	0	0	337	350	ANSYSVHGSALGAK					Mascot
1432.7795	1432.7849	0.0054	4	728	741	EFGITVEAVVAAAR					Mascot
1467.8054	1467.7821	-0.0233	-16	604	617	LPQLPGTSIEGVEK					Mascot
1689.9534	1689.7874	-0.166	-98	87	101	SINTIRFLAIDAVEK					Mascot
1715.8633	1715.9293	0.066	38	1	16	MASTSSLTLSQALFSR			Oxidation (M)[1]		Mascot
1837.0219	1837.027	0.0051	3	725	741	IYKEFGITVEAVVAAAR					Mascot
2107.1104	2107.0928	-0.0176	-8	450	470	VLPGFLGGSADLASSNM TLLK			Oxidation (M)[17]		Mascot
2142.0291	2142.0383	0.0092	4	357	374	SNLGWPYEPFHVPEDVQ K					Mascot
2299.0845	2299.0981	0.0136	6	565	585	AMPNILMFRPADGNETA GAYK			Oxidation (M)[2,7]		Mascot
3471.7754	3471.845	0.0696	20	178	212	TPGHPENFETPGVEVTT GPLGQGIANAVGLALAEK					Mascot

14

transketolase, chloroplastic [Momordica charantia]	XP_022147028.1	80665.8	6.15	18	247	100	100

Peptide Information

Calc. Mass	Obsrv. Mass	± da	ppm	Start	End	Sequence	Ion	C. I. %	Modification	Rank	Result Type

				Seq.	Seq.		Score			
888.392	888.4042	0.0122	14	471	477	MYGDFQK				Mascot
904.3869	904.393	0.0061	7	471	477	MYGDFQK			Oxidation (M)[1]	Mascot
996.4686	996.4788	0.0102	10	131	137	NPYWFNR				Mascot
996.4686	996.4788	0.0102	10	131	137	NPYWFNR	56	99.974		Mascot
1005.5615	1005.5667	0.0052	5	93	101	FLAIDAVEK				Mascot
1138.5123	1138.5271	0.0148	13	298	307	NGNTGYDEIR				Mascot
1193.5433	1193.5576	0.0143	12	427	437	YTPESPGDATR				Mascot
1193.5433	1193.5576	0.0143	12	427	437	YTPESPGDATR	46	99.75		Mascot
1221.6475	1221.6403	-0.0072	-6	325	336	VTTTIGFGSPNK				Mascot
1359.705	1359.7084	0.0034	3	438	449	NLSQQCLNALAK			Carbamidomethyl (C)[6]	Mascot
1361.6808	1361.6869	0.0061	4	337	350	ANSYSVHGSALGAK				Mascot
1432.7795	1432.7889	0.0094	7	728	741	EFGITVEAVVAAAR				Mascot
1432.7795	1432.7889	0.0094	7	728	741	EFGITVEAVVAAAR	27	81.577		Mascot
1467.8054	1467.7672	-0.0382	-26	604	617	LPQLPGTSIEGVEK				Mascot
1470.7893	1470.7758	-0.0135	-9	286	297	FEGLGWHVIWVK				Mascot
1689.9534	1689.7983	-0.1551	-92	87	101	SINTIRFLAIDAVEK				Mascot
1699.8684	1699.8131	-0.0553	-33	1	16	MASTSSLTLSQALFSR				Mascot
1715.8633	1715.9338	0.0705	41	1	16	MASTSSLTLSQALFSR			Oxidation (M)[1]	Mascot
1837.0219	1837.0209	-0.001	-1	725	741	IYKEFGITVEAVVAAAR				Mascot
2107.1104	2107.1028	-0.0076	-4	450	470	VLPGFLGGSADLASSNM TLLK			Oxidation (M)[17]	Mascot
2142.0291	2142.0459	0.0168	8	357	374	SNLGWPYEPFHVPEDVQ K				Mascot
2142.0291	2142.0459	0.0168	8	357	374	SNLGWPYEPFHVPEDVQ K	61	99.993		Mascot
2299.0845	2299.0908	0.0063	3	565	585	AMPNILMFRPADGNETA GAYK			Oxidation (M)[2,7]	Mascot
3471.7754	3471.8469	0.0715	21	178	212	TPGHPENFETPGVEVTT GPLGQGIANAVGLALAEK				Mascot

15	peptidyl-prolyl cis-trans isomerase CYP38, chloroplastic [Momordica charantia]	XP_022158926.1	48685.7	4.97	14	296	100	100

Peptide Information

Calc. Mass	Obsrv. Mass	± da	ppm	Start Seq.	End Seq.	Sequence	Ion Score	C. I. %	Modification	Rank	Result Type
856.4886	856.5408	0.0522	61	207	214	DAVAPKQK					Mascot
877.4811	877.4879	0.0068	8	249	256	AAVDMKVK			Oxidation (M)[5]		Mascot
955.4843	955.4942	0.0099	10	181	189	EHGSEVLGK					Mascot
1273.7362	1273.6639	-0.0723	-57	169	180	DLIISGLAESKK					Mascot
1295.5837	1295.6027	0.019	15	291	300	HFYDGMEIQR					Mascot
1295.5837	1295.6027	0.019	15	291	300	HFYDGMEIQR	54	99.966			Mascot
1311.5787	1311.5917	0.013	10	291	300	HFYDGMEIQR			Oxidation (M)[6]		Mascot
1311.5787	1311.5917	0.013	10	291	300	HFYDGMEIQR	49	99.894	Oxidation (M)[6]		Mascot
1360.6638	1360.7097	0.0459	34	18	29	SSIPTTHCRSSK			Carbamidomethyl (C)[8]		Mascot
1426.697	1426.7179	0.0209	15	359	371	LPFNAFGTMAMAR					Mascot
1442.6919	1442.707	0.0151	10	359	371	LPFNAFGTMAMAR			Oxidation (M)[9]		Mascot
1442.6919	1442.707	0.0151	10	359	371	LPFNAFGTMAMAR			Oxidation (M)[9]		Mascot
1458.6869	1458.6981	0.0112	8	359	371	LPFNAFGTMAMAR			Oxidation (M)[9,11]		Mascot
1458.8163	1458.6981	-0.1182	-81	166	179	QGKDLIISGLAESK					Mascot
1587.8411	1587.8265	-0.0146	-9	326	339	TRTVPLEIMVDGEK					Mascot
1614.8585	1614.8674	0.0089	6	130	143	EVQKPLEDISESLK					Mascot
1614.8585	1614.8674	0.0089	6	130	143	EVQKPLEDISESLK	66	99.998			Mascot
1729.8684	1729.8837	0.0153	9	340	354	APFYGETLEELGLYK					Mascot
1729.8684	1729.8837	0.0153	9	340	354	APFYGETLEELGLYK	121	100			Mascot
2013.9553	2013.9854	0.0301	15	372	388	EEFDNNSASSQVFWLLK					Mascot
2067.9951	2067.9558	-0.0393	-19	9	26	FCSSLVTSKSSIPTTHCR			Carbamidomethyl (C)[2,17]		Mascot
2563.1836	2563.2151	0.0315	12	301	325	ADGFVVQTGDPEGPAEG FIDPSTEK					Mascot

16

thioredoxin-like 1-2, chloroplastic [Momordica charantia] XP_022137159.1 31050.8 8.76 10 44 0

Peptide Information

Calc. Mass	Obsrv. Mass	± da	ppm	Start Seq.	End Seq.	Sequence	Ion Score	C. I. %	Modification	Rank	Result Type
1129.5605	1129.5035	-0.057	-50	169	177	TMCQALHIR			Carbamidomethyl (C)[3]		Mascot
1145.5554	1145.4626	-0.0928	-81	169	177	TMCQALHIR			Carbamidomethyl (C)[3], Oxidation (M)[2]		Mascot
1401.6865	1401.7827	0.0962	69	192	204	VCSFSCTNATIKK					Mascot
1657.7714	1657.7317	-0.0397	-24	127	141	LAIIDFYSPGCGGCK			Carbamidomethyl (C)[11,14]		Mascot
1772.9541	1772.8865	-0.0676	-38	233	249	LASAGELSLRPPSPYSK					Mascot

1773.88	1773.901	0.021	12	217	232	CSIVPARGLDESELER					Mascot
1786.8212	1786.8859	0.0647	36	187	203	GGEGRVCSFSCTNATIK			Carbamidomethyl (C)[7]		Mascot
1789	1788.8857	-0.1143	-64	78	94	ALNSLTIKAQASICISR					Mascot
1864.8495	1864.948	0.0985	53	25	42	SSGVSGFCSTSISASQFR			Carbamidomethyl (C)[8]		Mascot
1989.0045	1988.9231	-0.0814	-41	162	177	VNFEELKTMCQALHIR			Carbamidomethyl (C)[10]		Mascot
2004.9994	2004.9203	-0.0791	-39	162	177	VNFEELKTMCQALHIR			Carbamidomethyl (C)[10], Oxidation (M)[9]		Mascot
2004.9994	2004.9203	-0.0791	-39	162	177	VNFEELKTMCQALHIR	8	0	Carbamidomethyl (C)[10], Oxidation (M)[9]		Mascot
2127.0938	2127.0317	-0.0621	-29	1	19	MASSLKIGLCVSGLNEYLR			Carbamidomethyl (C)[10], Oxidation (M)[1]		Mascot

17

chromoplast-specific carotenoid-associated protein, chromoplastic [Momordica charantia] XP_022132925.1 35487.3 4.89 3 103 100 100

Peptide Information

Calc. Mass	Obsrv. Mass	± da	ppm	Start Seq.	End Seq.	Sequence	Ion Score	C. I. %	Modification	Rank	Result Type
970.5568	970.5516	-0.0052	-5	275	283	TISSQPPIK					Mascot
1243.5955	1243.5898	-0.0057	-5	106	116	ALVDSFYGTDR					Mascot
1243.5955	1243.5898	-0.0057	-5	106	116	ALVDSFYGTDR	80	100			Mascot
1371.6903	1371.6813	-0.009	-7	105	116	KALVDSFYGTDR					Mascot
1371.6903	1371.6813	-0.009	-7	105	116	KALVDSFYGTDR	18	0			Mascot

18

ACT domain-containing protein ACR11-like isoform X1 [Momordica charantia] XP_022153437.1 31965.5 5.71 8 213 100 100

Protein Group

ACT domain-containing protein ACR11-like isoform X2 [Momordica charantia] XP_022153438.1 31809.4 ##### ##### 3486

Peptide Information

Calc. Mass	Obsrv. Mass	± da	ppm	Start Seq.	End Seq.	Sequence	Ion Score	C. I. %	Modification	Rank	Result Type
808.41	808.4091	-0.0009	-1	261	266	FHVSYR					Mascot
856.5251	856.5237	-0.0014	-2	125	132	NLGLNVVK					Mascot
993.5265	993.4761	-0.0504	-51	261	268	FHVSYRGK					Mascot

1037.4899	1037.4879	-0.002	-2	288	296	RPTTEEASF					Mascot
1037.4899	1037.4879	-0.002	-2	288	296	RPTTEEASF	39	98.81			Mascot
1259.7028	1259.6793	-0.0235	-19	113	124	LGALLDTMNALK					Mascot
1269.6686	1269.6626	-0.006	-5	157	167	VDDPELLEAIR					Mascot
1269.6686	1269.6626	-0.006	-5	157	167	VDDPELLEAIR	38	98.677			Mascot
1275.6978	1275.6821	-0.0157	-12	113	124	LGALLDTMNALK			Oxidation (M)[8]		Mascot
1397.7635	1397.7528	-0.0107	-8	156	167	KVDDPELLEAIR					Mascot
1397.7635	1397.7528	-0.0107	-8	156	167	KVDDPELLEAIR	110	100			Mascot
1760.9298	1760.8669	-0.0629	-36	42	56	TSLALHAKCFCLLQR			Carbamidomethyl (C)[9]		Mascot

19

glycine-rich RNA-binding protein 3, mitochondrial-like [Momordica charantia]	XP_022134612.1	30572.7	4.49	5	109	100		100

Peptide Information

Calc. Mass	Obsrv. Mass	± da	ppm	Start Seq.	End Seq.	Sequence	Ion Score	C. I. %	Modification	Rank	Result Type
851.4006	851.3969	-0.0037	-4	116	122	VNYANDR					Mascot
936.4785	936.4688	-0.0097	-10	66	73	YGEVIEAR					Mascot
936.4785	936.4688	-0.0097	-10	66	73	YGEVIEAR	59	99.99			Mascot
1044.5797	1044.5616	-0.0181	-17	74	82	VIVDRETGR					Mascot
1512.7693	1512.7351	-0.0342	-23	61	73	EAFTKYGEVIEAR					Mascot
2943.3755	2943.3655	-0.01	-3	85	112	GFGFVTYTSSEEASSAIQ ALDGQDLHGR					Mascot
2943.3755	2943.3655	-0.01	-3	85	112	GFGFVTYTSSEEASSAIQ ALDGQDLHGR	37	98.569			Mascot

20

uncharacterized protein LOC111025695 [Momordica charantia]	XP_022159284.1	35471.4	4.9	12	236	100		100

Peptide Information

Calc. Mass	Obsrv. Mass	± da	ppm	Start Seq.	End Seq.	Sequence	Ion Score	C. I. %	Modification	Rank	Result Type
813.508	813.4987	-0.0093	-11	164	170	LTLPLEK					Mascot
935.4833	935.4739	-0.0094	-10	209	216	NDFELGLK					Mascot
1286.674	1286.6376	-0.0364	-28	34	44	VKDFIHDVGEK					Mascot

1310.6199	1310.5938	-0.0261	-20	258	268	DFGSALWDMIR					Mascot
1326.6147	1326.6022	-0.0125	-9	258	268	DFGSALWDMIR			Oxidation (M)[9]		Mascot
1328.7573	1328.7382	-0.0191	-14	152	163	VDLIVDVPVFGR					Mascot
1419.7478	1419.7271	-0.0207	-15	205	216	LENKNDFELGLK					Mascot
1419.7478	1419.7271	-0.0207	-15	205	216	LENKNDFELGLK	53	99.947			Mascot
1443.705	1443.6846	-0.0204	-14	278	290	GNIHVNTPFGEMK					Mascot
1459.6998	1459.6672	-0.0326	-22	278	290	GNIHVNTPFGEMK			Oxidation (M)[12]		Mascot
1459.6998	1459.6672	-0.0326	-22	278	290	GNIHVNTPFGEMK	24	56.658	Oxidation (M)[12]		Mascot
1465.6554	1465.733	0.0776	53	21	33	EDENEGKGGFLDR					Mascot
1569.9364	1569.9191	-0.0173	-11	150	163	IKVDLIVDVPVFGR					Mascot
1705.8174	1705.8015	-0.0159	-9	1	15	MASSDRPEIVERGDK			Oxidation (M)[1]		Mascot
1813.9583	1813.9158	-0.0425	-23	171	186	TGEIPIPYKPDVDIEK					Mascot
1813.9583	1813.9158	-0.0425	-23	171	186	TGEIPIPYKPDVDIEK	106	100			Mascot
2164.0857	2164.1196	0.0339	16	271	290	GTGYTIKGNIHVNTPFGEMK					Mascot

21	ribulose bisphosphate carboxylase/oxygenase activase, chloroplastic [Momordica charantia]	XP_022138899.1	48023.3	6.27	9	442	100	100

Peptide Information

Calc. Mass	Obsrv. Mass	± da	ppm	Start Seq.	End Seq.	Sequence	Ion Score	C. I. %	Modification	Rank	Result Type
895.4156	895.3989	-0.0167	-19	356	362	VYDDEVR					Mascot
940.4675	940.4474	-0.0201	-21	307	313	FYWAPTR					Mascot
940.4675	940.4474	-0.0201	-21	307	313	FYWAPTR	46	99.759			Mascot
1152.714	1152.6809	-0.0331	-29	161	171	VPLILGIWGGK					Mascot
1228.6031	1228.573	-0.0301	-24	176	185	SFQCELVFAK			Carbamidomethyl (C)[4]		Mascot
1228.6031	1228.573	-0.0301	-24	176	185	SFQCELVFAK	63	99.995	Carbamidomethyl (C)[4]		Mascot
1639.7567	1639.714	-0.0427	-26	226	240	MSCLFINDLDAGAGR			Carbamidomethyl (C)[3]		Mascot
1639.7567	1639.714	-0.0427	-26	226	240	MSCLFINDLDAGAGR	48	99.867	Carbamidomethyl (C)[3]		Mascot
1655.7517	1655.696	-0.0557	-34	226	240	MSCLFINDLDAGAGR			Carbamidomethyl (C)[3], Oxidation (M)[1]		Mascot
1882.9698	1882.9327	-0.0371	-20	337	353	LVDTFPGQSIDFFGALR					Mascot
1882.9698	1882.9327	-0.0371	-20	337	353	LVDTFPGQSIDFFGALR	142	100			Mascot
1906.9579	1906.905	-0.0529	-28	397	412	LLEYGNMLVQEQENVK					Mascot
1922.9529	1922.8799	-0.073	-38	397	412	LLEYGNMLVQEQENVK			Oxidation (M)[7]		Mascot
2063.0591	2063.0146	-0.0445	-22	397	413	LLEYGNMLVQEQENVKR					Mascot

Calc. Mass	Obsrv. Mass	± da	ppm	Start Seq.	End Seq.	Sequence	Ion Score	C. I. %	Modification	Rank	Result Type
2079.054	2079.0002	-0.0538	-26	397	413	LLEYGNMLVQEQENVKR			Oxidation (M)[7]		Mascot
2089.1692	2089.1274	-0.0418	-20	282	300	VPIIVTGNDFSTLYAPLIR					Mascot
2089.1692	2089.1274	-0.0418	-20	282	300	VPIIVTGNDFSTLYAPLIR	125	100			Mascot

22

30S ribosomal protein S1, chloroplastic [Momordica charantia]	XP_022138241.1	45213.3	5.33	13	389	100	100

Peptide Information

Calc. Mass	Obsrv. Mass	± da	ppm	Start Seq.	End Seq.	Sequence	Ion Score	C. I. %	Modification	Rank	Result Type
970.5139	970.4961	-0.0178	-18	315	322	VMILSHDR					Mascot
986.5087	986.4674	-0.0413	-42	315	322	VMILSHDR			Oxidation (M)[2]		Mascot
1055.6208	1055.6024	-0.0184	-17	200	210	GGVVAVVEGLR					Mascot
1055.6208	1055.6024	-0.0184	-17	200	210	GGVVAVVEGLR	59	99.987			Mascot
1082.4935	1082.4761	-0.0174	-16	352	360	AEEMAQTFR					Mascot
1098.4884	1098.4653	-0.0231	-21	352	360	AEEMAQTFR			Oxidation (M)[4]		Mascot
1128.5718	1128.558	-0.0138	-12	334	343	LEPTPGDMIR					Mascot
1144.5668	1144.5376	-0.0292	-26	334	343	LEPTPGDMIR			Oxidation (M)[8]		Mascot
1225.5994	1225.5635	-0.0359	-29	2	12	ASMAQQFTGLR			Oxidation (M)[3]		Mascot
1237.5696	1237.549	-0.0206	-17	236	245	FVEVDEEQSR					Mascot
1237.5696	1237.549	-0.0206	-17	236	245	FVEVDEEQSR	45	99.724			Mascot
1256.6667	1256.6422	-0.0245	-19	333	343	KLEPTPGDMIR					Mascot
1272.6617	1272.6329	-0.0288	-23	333	343	KLEPTPGDMIR			Oxidation (M)[9]		Mascot
1280.627	1280.605	-0.022	-17	170	179	SIQYDLAWER					Mascot
1280.627	1280.605	-0.022	-17	170	179	SIQYDLAWER	65	99.997			Mascot
1310.6852	1310.661	-0.0242	-18	138	149	HVEEAGIFPGVR					Mascot
1310.6852	1310.661	-0.0242	-18	138	149	HVEEAGIFPGVR	28	83.552			Mascot
1372.6348	1372.6204	-0.0144	-10	1	12	MASMAQQFTGLR			Oxidation (M)[1,4]		Mascot
1551.8643	1551.8333	-0.031	-20	136	149	IKHVEEAGIFPGVR					Mascot
1578.7727	1578.8	0.0273	17	363	376	IAQAEAMARADMLR			Oxidation (M)[7,12]		Mascot
2188.092	2188.0374	-0.0546	-25	72	91	NAPVEGISFTLEDFHAALEK					Mascot
2188.092	2188.0374	-0.0546	-25	72	91	NAPVEGISFTLEDFHAALEK	145	100			Mascot

23

ribulose bisphosphate carboxylase/oxygenase activase, chloroplastic [Momordica charantia]	XP_022138899.1	48023.3	6.27	11	422	100	100

Peptide Information

Calc. Mass	Obsrv. Mass	± da	ppm	Start Seq.	End Seq.	Sequence	Ion Score	C. I. %	Modification	Rank	Result Type
895.4156	895.3979	-0.0177	-20	356	362	VYDDEVR					Mascot
940.4675	940.4453	-0.0222	-24	307	313	FYWAPTR					Mascot
940.4675	940.4453	-0.0222	-24	307	313	FYWAPTR	26	76.679			Mascot
1152.714	1152.6183	-0.0957	-83	161	171	VPLILGIWGGK					Mascot
1228.6031	1228.5701	-0.033	-27	176	185	SFQCELVFAK			Carbamidomethyl (C)[4]		Mascot
1228.6031	1228.5701	-0.033	-27	176	185	SFQCELVFAK	66	99.998	Carbamidomethyl (C)[4]		Mascot
1328.6456	1328.6256	-0.02	-15	304	313	MEKFYWAPTR					Mascot
1340.6382	1340.6047	-0.0335	-25	307	316	FYWAPTREDR					Mascot
1639.7567	1639.7001	-0.0566	-35	226	240	MSCLFINDLDAGAGR			Carbamidomethyl (C)[3]		Mascot
1882.9698	1882.9253	-0.0445	-24	337	353	LVDTFPGQSIDFFGALR					Mascot
1882.9698	1882.9253	-0.0445	-24	337	353	LVDTFPGQSIDFFGALR	138	100			Mascot
1906.9579	1906.8899	-0.068	-36	397	412	LLEYGNMLVQEQENVK					Mascot
1922.9529	1922.8811	-0.0718	-37	397	412	LLEYGNMLVQEQENVK			Oxidation (M)[7]		Mascot
2063.0591	2063.0088	-0.0503	-24	397	413	LLEYGNMLVQEQENVKR					Mascot
2063.0591	2063.0088	-0.0503	-24	397	413	LLEYGNMLVQEQENVKR	28	86.518			Mascot
2079.054	2078.9939	-0.0601	-29	397	413	LLEYGNMLVQEQENVKR			Oxidation (M)[7]		Mascot
2089.1692	2089.1199	-0.0493	-24	282	300	VPIIVTGNDFSTLYAPLIR					Mascot
2089.1692	2089.1199	-0.0493	-24	282	300	VPIIVTGNDFSTLYAPLIR	136	100			Mascot

24

actin-7 [Momordica charantia]	XP_022132857.1	41682.9	5.31	18	454	100	100

Protein Group

actin-7 [Momordica charantia]	XP_022158718.1	41682.9	#####<br>#####<br>7954

Peptide Information

Calc. Mass	Obsrv. Mass	± da	ppm	Start Seq.	End Seq.	Sequence	Ion Score	C. I. %	Modification	Rank	Result Type
890.4764	890.5403	0.0639	72	209	215	EIVRDMK					Mascot

976.4483	976.4277	-0.0206	-21	21	30	AGFAGDDAPR				Mascot
1144.5602	1144.5256	-0.0346	-30	42	52	HTGVMVGMGQK				Mascot
1160.5552	1160.525	-0.0302	-26	42	52	HTGVMVGMGQK			Oxidation (M)[5]	Mascot
1176.5354	1176.5128	-0.0226	-19	199	208	GYMFTTTAER				Mascot
1176.5354	1176.5128	-0.0226	-19	199	208	GYMFTTTAER	46	99.78		Mascot
1182.5273	1182.5116	-0.0157	-13	53	63	DAYVGDEAQSK				Mascot
1192.5304	1192.5005	-0.0299	-25	199	208	GYMFTTTAER			Oxidation (M)[3]	Mascot
1192.5304	1192.5005	-0.0299	-25	199	208	GYMFTTTAER			Oxidation (M)[3]	Mascot
1198.7056	1198.6801	-0.0255	-21	31	41	AVFPSIVGRPR				Mascot
1198.7056	1198.6801	-0.0255	-21	31	41	AVFPSIVGRPR				Mascot
1445.6655	1445.6359	-0.0296	-20	362	374	GEYDESGPSIVHR				Mascot
1515.7491	1515.7161	-0.033	-22	87	97	IWHHTFYNELR				Mascot
1515.7491	1515.7161	-0.033	-22	87	97	IWHHTFYNELR	71	100		Mascot
1531.8148	1531.7096	-0.1052	-69	180	193	LDLAGRDLTDALMK				Mascot
1547.8098	1547.7078	-0.102	-66	180	193	LDLAGRDLTDALMK			Oxidation (M)[13]	Mascot
1547.8098	1547.7078	-0.102	-66	180	193	LDLAGRDLTDALMK			Oxidation (M)[13]	Mascot
1774.897	1774.8544	-0.0426	-24	241	256	NYELPDGQVITIGAER				Mascot
1774.897	1774.8544	-0.0426	-24	241	256	NYELPDGQVITIGAER	143	100		Mascot
1788.8949	1788.8665	-0.0284	-16	194	208	ILTERGYMFTTTAER				Mascot
1788.8949	1788.8665	-0.0284	-16	194	208	ILTERGYMFTTTAER				Mascot
1855.9324	1855.8711	-0.0613	-33	218	233	LAYVALDYEQELETAK				Mascot
1932.8796	1932.8229	-0.0567	-29	71	86	YPIEHGIVSNWDDMEK				Mascot
1948.8746	1948.8154	-0.0592	-30	71	86	YPIEHGIVSNWDDMEK			Oxidation (M)[14]	Mascot

1954.0645	1954.0081	-0.0564	-29	98	115	VAPEEHPVLLTEAPLNPK				Mascot
1954.0645	1954.0081	-0.0564	-29	98	115	VAPEEHPVLLTEAPLNPK	92	100		Mascot
1975.91	1975.9998	0.0898	45	2	20	ADAEDIQPLVCDNGTGMVK				Mascot
2199.0752	2199.0149	-0.0603	-27	294	314	DLYGNIVLSGGSTMFPGIADR			Oxidation (M)[14]	Mascot
2327.1702	2327.1077	-0.0625	-27	293	314	KDLYGNIVLSGGSTMFPGIADR			Oxidation (M)[15]	Mascot
3151.6423	3151.5981	-0.0442	-14	150	179	TTGIVLDSGDGVSHTVPIYEGYALPHAILR				Mascot

25

sedoheptulose-1,7-bisphosphatase, chloroplastic [Momordica charantia]	XP_022156931.1	42044.2	5.95	20	397	100	100

Peptide Information

Calc. Mass	Obsrv. Mass	± da	ppm	Start Seq.	End Seq.	Sequence	Ion Score	C. I. %	Modification	Rank	Result Type
809.4515	809.4296	-0.0219	-27	279	284	EKYTLR					Mascot
853.4414	853.4294	-0.012	-14	355	362	TQVAYGSK					Mascot
869.3748	869.3666	-0.0082	-9	332	340	AGGYSSDGR					Mascot
886.5356	886.462	-0.0736	-83	47	54	QVPKSSIK					Mascot
903.5046	903.4917	-0.0129	-14	254	261	LFSPGNLR					Mascot
903.5046	903.4917	-0.0129	-14	254	261	LFSPGNLR	45	99.64			Mascot
908.5451	908.5264	-0.0187	-21	217	224	TTYVLALK					Mascot
912.5189	912.5021	-0.0168	-18	272	278	LINYYVK					Mascot
963.4629	963.4214	-0.0415	-43	245	253	ETTEIGEGK					Mascot
1170.5138	1170.527	0.0132	11	90	99	LMMCMGEALR			Oxidation (M)[2]		Mascot
1184.4857	1184.5093	0.0236	20	1	10	METGIACCAR			Carbamidomethyl (C)[7,8], Oxidation (M)[1]		Mascot
1185.5059	1185.483	-0.0229	-19	262	271	ATFDNPDYDK					Mascot
1188.5532	1188.5321	-0.0211	-18	368	377	FEETLYGSSR					Mascot
1188.5532	1188.5321	-0.0211	-18	368	377	FEETLYGSSR	40	99.003			Mascot
1202.5037	1202.5466	0.0429	36	90	99	LMMCMGEALR			Oxidation (M)[2,3,5]		Mascot
1251.658	1251.627	-0.031	-25	302	313	GIFTNVTSPSTK					Mascot
1394.7638	1394.6627	-0.1011	-72	34	46	SLKASSLFGESLR					Mascot
1478.7057	1478.6755	-0.0302	-20	202	216	GSDQVAAAMGVYGPR	127	100			Mascot
1494.7006	1494.661	-0.0396	-26	202	216	GSDQVAAAMGVYGPR			Oxidation (M)[9]		Mascot
1494.7006	1494.661	-0.0396	-26	202	216	GSDQVAAAMGVYGPR	90	100	Oxidation (M)[9]		Mascot
1510.7458	1510.6575	-0.0883	-58	68	80	CEIGDSLEVFLTK			Carbamidomethyl (C)[1]		Mascot
1574.9193	1574.866	-0.0533	-34	318	331	LLFEVAPLGFLVEK					Mascot
1633.8618	1633.7942	-0.0676	-41	285	299	YTGGMVPDVNQIIVK					Mascot
1649.8568	1649.8019	-0.0549	-33	285	299	YTGGMVPDVNQIIVK			Oxidation (M)[5]		Mascot
1649.8568	1649.8019	-0.0549	-33	285	299	YTGGMVPDVNQIIVK	81	100	Oxidation (M)[5]		Mascot
2079.0071	2078.9524	-0.0547	-26	262	278	ATFDNPDYDKLINYYVK					Mascot

2079.0071 2078.9524 -0.0547 -26 262 278 ATFDNPDYDKLINYYVK Mascot

2717.1853	2717.1184	-0.0669	-25	107	132	TASCGGTACVNSFGDEQ LAVDMLADK			Carbamidomethyl (C)[4,9]		Mascot
2733.1802	2733.1086	-0.0716	-26	107	132	TASCGGTACVNSFGDEQ LAVDMLADK			Carbamidomethyl (C)[4,9], Oxidation (M)[22]		Mascot

26

oxygen-evolving enhancer protein 1, chloroplastic [Momordica charantia]	XP_022133288.1	34923.9	5.91	10	271	100	100

Peptide Information

Calc. Mass	Obsrv. Mass	± da	ppm	Start Seq.	End Seq.	Sequence	Ion Score	C. I. %	Modification	Rank	Result Type
850.4305	850.4222	-0.0083	-10	238	245	GSSFLDPK					Mascot
964.5866	964.5781	-0.0085	-9	209	216	VPFLFTIK					Mascot
1080.5573	1080.5433	-0.014	-13	92	100	LTFDEIQSK					Mascot
1235.6227	1235.5857	-0.037	-30	273	284	ENIKNASSSTGK					Mascot
1236.6583	1236.6439	-0.0144	-12	91	100	RLTFDEIQSK					Mascot
1236.6583	1236.6439	-0.0144	-12	91	100	RLTFDEIQSK	9	0			Mascot
1479.7762	1479.703	-0.0732	-49	23	36	TGTSQLRSSQTVSK					Mascot
1562.7559	1562.7378	-0.0181	-12	248	264	GGSTGYDNAVALPAGGR					Mascot
1562.7559	1562.7378	-0.0181	-12	248	264	GGSTGYDNAVALPAGGR	93	100			Mascot
1614.8269	1614.7666	-0.0603	-37	47	60	LTCSLHSDLKDVAR			Carbamidomethyl (C)[3]		Mascot
1760.8813	1760.8611	-0.0202	-11	192	208	DGIDYAAVTVQLPGGER					Mascot
1760.8813	1760.8611	-0.0202	-11	192	208	DGIDYAAVTVQLPGGER	131	100			Mascot
2254.1125	2254.0854	-0.0271	-12	167	187	LTYTLDEIEGPFEVGADG SIK					Mascot

27

oxygen-evolving enhancer protein 1, chloroplastic [Momordica charantia]	XP_022133288.1	34923.9	5.91	9	330	100	100

Peptide Information

Calc. Mass	Obsrv. Mass	± da	ppm	Start Seq.	End Seq.	Sequence	Ion Score	C. I. %	Modification	Rank	Result Type
964.5866	964.571	-0.0156	-16	209	216	VPFLFTIK					Mascot
964.5866	964.571	-0.0156	-16	209	216	VPFLFTIK	64	99.996			Mascot
1080.5573	1080.5437	-0.0136	-13	92	100	LTFDEIQSK					Mascot

1236.6583	1236.6438	-0.0145	-12	91	100	RLTFDEIQSK					Mascot
1236.6583	1236.6438	-0.0145	-12	91	100	RLTFDEIQSK	11	0			Mascot
1562.7559	1562.734	-0.0219	-14	248	264	GGSTGYDNAVALPAGGR					Mascot
1562.7559	1562.734	-0.0219	-14	248	264	GGSTGYDNAVALPAGGR	53	99.949			Mascot
1614.8269	1614.7528	-0.0741	-46	47	60	LTCSLHSDLKDVAR			Carbamidomethyl (C)[3]		Mascot
1614.8269	1614.7528	-0.0741	-46	47	60	LTCSLHSDLKDVAR			Carbamidomethyl (C)[3]		Mascot
1759.861	1759.8458	-0.0152	-9	147	162	AEGVNKNSPPEFQNTK					Mascot
1760.8813	1760.8563	-0.025	-14	192	208	DGIDYAAVTVQLPGGER					Mascot
1760.8813	1760.8563	-0.025	-14	192	208	DGIDYAAVTVQLPGGER	151	100			Mascot
2254.1125	2254.0757	-0.0368	-16	167	187	LTYTLDEIEGPFEVGADG SIK					Mascot
2254.1125	2254.0757	-0.0368	-16	167	187	LTYTLDEIEGPFEVGADG SIK	20	10.131			Mascot
2294.1299	2294.0752	-0.0547	-24	188	208	FEEKDGIDYAAVTVQLPG GER					Mascot

28	oxygen-evolving enhancer protein 1, chloroplastic [Momordica charantia]	XP_022133288.1	34923.9	5.91	11	558	100	100

Peptide Information

Calc. Mass	Obsrv. Mass	± da	ppm	Start Seq.	End Seq.	Sequence	Ion Score	C. I. %	Modification	Rank	Result Type
850.4305	850.427	-0.0035	-4	238	245	GSSFLDPK					Mascot
964.5866	964.5657	-0.0209	-22	209	216	VPFLFTIK					Mascot
964.5866	964.5657	-0.0209	-22	209	216	VPFLFTIK	58	99.985			Mascot
1080.5573	1080.5377	-0.0196	-18	92	100	LTFDEIQSK					Mascot
1235.6227	1235.6096	-0.0131	-11	273	284	ENIKNASSSTGK					Mascot
1236.6583	1236.6378	-0.0205	-17	91	100	RLTFDEIQSK					Mascot
1236.6583	1236.6378	-0.0205	-17	91	100	RLTFDEIQSK	35	97.149			Mascot
1562.7559	1562.7268	-0.0291	-19	248	264	GGSTGYDNAVALPAGGR					Mascot
1562.7559	1562.7268	-0.0291	-19	248	264	GGSTGYDNAVALPAGGR	166	100			Mascot
1614.8269	1614.7509	-0.076	-47	47	60	LTCSLHSDLKDVAR			Carbamidomethyl (C)[3]		Mascot
1709.8817	1709.7924	-0.0893	-52	30	46	SSQTVSKAFGIEATGAR					Mascot
1760.8813	1760.8469	-0.0344	-20	192	208	DGIDYAAVTVQLPGGER					Mascot
1760.8813	1760.8469	-0.0344	-20	192	208	DGIDYAAVTVQLPGGER	157	100			Mascot
2254.1125	2254.0601	-0.0524	-23	167	187	LTYTLDEIEGPFEVGADG					Mascot

						SIK					
2254.1125	2254.0601	-0.0524	-23	167	187	LTYTLDEIEGPFEVGADG	97	100			Mascot
						SIK					
2294.1299	2294.0693	-0.0606	-26	188	208	FEEKDGIDYAAVTVQLPG					Mascot
						GER					

29

protein MET1, chloroplastic [Momordica charantia] XP_022153815.1 36556.4 5.67 12 284 100 100

Peptide Information

Calc. Mass	Obsrv. Mass	± da	ppm	Start Seq.	End Seq.	Sequence	Ion Score	C. I. %	Modification	Rank	Result Type
800.3971	800.3944	-0.0027	-3	149	154	TMYTIR			Oxidation (M)[2]		Mascot
802.4166	802.4094	-0.0072	-9	214	219	RENDLR					Mascot
881.425	881.4208	-0.0042	-5	230	236	YEEALEK					Mascot
937.499	937.4852	-0.0138	-15	120	128	TGLFTVGDK					Mascot
1084.5569	1084.6017	0.0448	41	149	156	TMYTIRQR			Oxidation (M)[2]		Mascot
1084.5569	1084.6017	0.0448	41	149	156	TMYTIRQR			Oxidation (M)[2]		Mascot
1126.5264	1126.5122	-0.0142	-13	171	180	TDSFGELTEK					Mascot
1724.849	1724.8094	-0.0396	-23	310	324	FDESFINENAINAIK					Mascot
1724.849	1724.8094	-0.0396	-23	310	324	FDESFINENAINAIK	84	100			Mascot
1820.8773	1820.832	-0.0453	-25	101	119	GRDGGTYIDAIAPGGSAD					Mascot
						K					
1880.9501	1880.9214	-0.0287	-15	309	324	RFDESFINENAINAIK					Mascot
1880.9501	1880.9214	-0.0287	-15	309	324	RFDESFINENAINAIK	82	100			Mascot
2039.0081	2038.9867	-0.0214	-10	269	287	AGLSALEDALEAGFEDFK					Mascot
						R					
2039.0081	2038.9867	-0.0214	-10	269	287	AGLSALEDALEAGFEDFK	74	100			Mascot
						R					
2138.0918	2138.0703	-0.0215	-10	129	148	VIATSAVFGTEIWPAAEY					Mascot
						GR					
2177.0972	2177.0271	-0.0701	-32	290	308	TDPDLSTLRTSEEFEPLV					Mascot
						K					

30

protease Do-like 1, chloroplastic [Momordica charantia] XP_022134109.1 46827.8 6.67 12 253 100 100

Peptide Information

Calc. Mass	Obsrv. Mass	± da	ppm	Start Seq.	End Seq.	Sequence	Ion Score	C. I. %	Modification	Rank	Result Type
815.4985	815.4949	-0.0036	-4	415	421	VTVEVLR					Mascot
855.541	855.5117	-0.0293	-34	369	376	AGLLPTKR					Mascot
996.6564	996.6498	-0.0066	-7	332	340	VTRPILGIK					Mascot
996.6564	996.6498	-0.0066	-7	332	340	VTRPILGIK					Mascot
1024.5059	1024.4945	-0.0114	-11	396	404	VTNGSDLYR					Mascot
1145.6161	1145.6091	-0.007	-6	114	123	LQTDELATVR					Mascot
1214.7103	1214.6774	-0.0329	-27	411	421	VGDKVTVEVLR					Mascot
1273.7111	1273.703	-0.0081	-6	113	123	KLQTDELATVR					Mascot
1273.7111	1273.703	-0.0081	-6	113	123	KLQTDELATVR	21	33.913			Mascot
1423.7441	1423.7336	-0.0105	-7	165	176	DGHIVTNYHVIR					Mascot
1423.7441	1423.7336	-0.0105	-7	165	176	DGHIVTNYHVIR	67	99.998			Mascot
1776.0742	1776.0607	-0.0135	-8	216	232	LRPIPVGISADLLVGQK					Mascot
1776.0742	1776.0607	-0.0135	-8	216	232	LRPIPVGISADLLVGQK	110	100			Mascot
2037.9797	2038.0183	0.0386	19	422	439	GDHMEKIPVTLEPKPDES			Oxidation (M)[4]		Mascot
2385.2925	2385.281	-0.0115	-5	233	255	VFAIGNPFGLDHTLTTGVI SGLR					Mascot
2385.2925	2385.281	-0.0115	-5	233	255	VFAIGNPFGLDHTLTTGVI SGLR	13	0			Mascot
2736.4204	2736.3965	-0.0239	-9	341	368	FAPDQSVEQLGVSGVLV LDAPANGPAGK					Mascot

31

fructose-bisphosphate aldolase 1, chloroplastic [Momordica charantia]	XP_022146084.1	42808.1	6.86	14	309	100		100

Peptide Information

Calc. Mass	Obsrv. Mass	± da	ppm	Start Seq.	End Seq.	Sequence	Ion Score	C. I. %	Modification	Rank	Result Type
811.5036	811.5006	-0.003	-4	138	145	QGIVPGIK					Mascot
822.4355	822.437	0.0015	2	238	244	TFEVAQK					Mascot
855.5046	855.4503	-0.0543	-63	26	33	QPAVSVVR					Mascot
873.4577	873.4637	0.006	7	202	209	EAAWGLAR					Mascot
901.5101	901.4515	-0.0586	-65	368	376	ANSLAQLGK					Mascot
947.4979	947.4932	-0.0047	-5	339	346	ALQNTCLK			Carbamidomethyl (C)[6]		Mascot

1098.5327	1098.5315	-0.0012	-1	172	181	AAAYYQQGAR					Mascot
1098.5327	1098.5315	-0.0012	-1	172	181	AAAYYQQGAR	56	99.979			Mascot
1387.7175	1387.7139	-0.0036	-3	82	94	LASIGLENTEANR					Mascot
1387.7175	1387.7139	-0.0036	-3	82	94	LASIGLENTEANR	55	99.975			Mascot
1452.8422	1452.8348	-0.0074	-5	187	201	TVVSIPNGPSALAVK					Mascot
1466.6614	1466.6731	0.0117	8	67	80	GILAMDESNATCGK			Carbamidomethyl (C)[12]		Mascot
1482.6564	1482.6487	-0.0077	-5	67	80	GILAMDESNATCGK			Carbamidomethyl (C)[12], Oxidation (M)[5]		Mascot
1543.8187	1543.808	-0.0107	-7	81	94	RLASIGLENTEANR					Mascot
2363.1653	2363.1538	-0.0115	-5	110	130	YISGAILFEETLYQSTVDGEK					Mascot
2427.1721	2427.1748	0.0027	1	149	171	GLVPLPGSNNESWCQGLDGLASR			Carbamidomethyl (C)[14]		Mascot
2427.1721	2427.1748	0.0027	1	149	171	GLVPLPGSNNESWCQGLDGLASR	139	100	Carbamidomethyl (C)[14]		Mascot
2444.2239	2444.1499	-0.074	-30	34	56	CHPTAAPSTLTVRAGSYADELVK			Carbamidomethyl (C)[1]		Mascot

32

fruit protein pKIWI502 [Momordica charantia] XP_022150511.1 32744.1 7.77 8 172 100 100

Peptide Information

Calc. Mass	Obsrv. Mass	± da	ppm	Start Seq.	End Seq.	Sequence	Ion Score	C. I. %	Modification	Rank	Result Type
948.5261	948.5255	-0.0006	-1	98	105	AGQYLQLR					Mascot
948.5261	948.5255	-0.0006	-1	98	105	AGQYLQLR	52	99.952			Mascot
1197.5422	1197.5375	-0.0047	-4	224	233	FEEWESSGVK					Mascot
1199.6168	1199.5265	-0.0903	-75	204	213	SDVRLYYGAR					Mascot
1260.6617	1260.6299	-0.0318	-25	151	162	GDVVQLSQVMGK					Mascot
1276.6566	1276.676	0.0194	15	151	162	GDVVQLSQVMGK			Oxidation (M)[10]		Mascot
1388.7567	1388.7371	-0.0196	-14	150	162	KGDVVQLSQVMGK					Mascot
1404.7516	1404.728	-0.0236	-17	150	162	KGDVVQLSQVMGK			Oxidation (M)[11]		Mascot
1475.7775	1475.767	-0.0105	-7	136	149	SVEGSIAELLCGLK	92	100	Carbamidomethyl (C)[11]		Mascot
1907.0273	1906.9608	-0.0665	-35	174	191	DYPTVLIFATGSGISPIR					Mascot
1961.8699	1961.8623	-0.0076	-4	218	233	MAYQDRFEEWESSGVK					Mascot

33

20 kDa chaperonin, chloroplastic [Momordica charantia]	XP_022147534.1	26725.3	8.76	11	218	100	100

Peptide Information

Calc. Mass	Obsrv. Mass	± da	ppm	Start Seq.	End Seq.	Sequence	Ion Score	C. I. %	Modification	Rank	Result Type
828.3886	828.396	0.0074	9	229	235	YAGNEFK					Mascot
846.4567	846.4637	0.007	8	75	81	IKEAEEK					Mascot
894.5043	894.4775	-0.0268	-30	45	52	SYTGLVVR					Mascot
952.5098	952.5049	-0.0049	-5	121	129	TGAQVVYSK					Mascot
970.4952	970.5006	0.0054	6	161	168	DLQPLNDR					Mascot
970.4952	970.5006	0.0054	6	161	168	DLQPLNDR	52	99.938			Mascot
1009.4949	1009.4996	0.0047	5	238	246	DGSDYIALR					Mascot
1009.4949	1009.4996	0.0047	5	238	246	DGSDYIALR	60	99.991			Mascot
1149.6263	1149.6267	0.0004	0	61	70	YTSIKPLGDR					Mascot
1149.6263	1149.6267	0.0004	0	61	70	YTSIKPLGDR	52	99.943			Mascot
1532.7327	1532.7244	-0.0083	-5	147	160	EDDIVGILETDDAK					Mascot
1887.0698	1887.0472	-0.0226	-12	53	70	AATVVAPKYTSIKPLGDR					Mascot
1920.0226	1919.9419	-0.0807	-42	130	146	YAGTELEFNGSKHLILK					Mascot
2484.21	2484.2114	0.0014	1	147	168	EDDIVGILETDDAKDLQPL NDR					Mascot

34

ascorbate peroxidase [Momordica charantia]	AGJ72851.1	27407.8	5.43	14	652	100	100

Peptide Information

Calc. Mass	Obsrv. Mass	± da	ppm	Start Seq.	End Seq.	Sequence	Ion Score	C. I. %	Modification	Rank	Result Type
917.4695	917.5082	0.0387	42	31	38	NCAPLMLR					Mascot
923.4404	923.4523	0.0119	13	53	61	TGGPFGTMR					Mascot
923.4404	923.4523	0.0119	13	53	61	TGGPFGTMR					Mascot
939.4353	939.4443	0.009	10	53	61	TGGPFGTMR			Oxidation (M)[8]		Mascot
939.4353	939.4443	0.009	10	53	61	TGGPFGTMR			Oxidation (M)[8]		Mascot
974.491	974.5038	0.0128	13	31	38	NCAPLMLR			Carbamidomethyl (C)[2]		Mascot
990.4859	990.4941	0.0082	8	31	38	NCAPLMLR			Carbamidomethyl (C)[2], Oxidation (M)[6]		Mascot
1073.5837	1073.5892	0.0055	5	199	208	EGLLQLASDK					Mascot
1250.6012	1250.6146	0.0134	11	120	130	EDKPEPPPEGR					Mascot
1250.6012	1250.6146	0.0134	11	120	130	EDKPEPPPEGR	70	100			Mascot

Calc. Mass	Obsrv. Mass	± da	ppm	Start Seq.	End Seq.	Sequence	Ion Score	C. I. %	Modification	Rank	Result Type
1287.6467	1287.6566	0.0099	8	188	198	SYFTELLTGEK					Mascot
1287.6467	1287.6566	0.0099	8	188	198	SYFTELLTGEK	60	99.99			Mascot
1309.6859	1309.6185	-0.0674	-51	131	142	LPDATKGSDHLR					Mascot
1401.6355	1401.6403	0.0048	3	4	14	CYPVVSEEYQK			Carbamidomethyl (C)[1]		Mascot
1566.7369	1566.8794	0.1425	91	39	52	LAWHSAGTYCKDSK					Mascot
1583.9155	1583.9269	0.0114	7	209	222	ALLSDPVFRPLVEK					Mascot
1583.9155	1583.9269	0.0114	7	209	222	ALLSDPVFRPLVEK	79	100			Mascot
1605.824	1605.8998	0.0758	47	25	38	GFIADKNCAPLMLR			Carbamidomethyl (C)[8]		Mascot
1636.8402	1636.8525	0.0123	8	64	79	SELAHGANNGLDIAVR					Mascot
1808.8855	1808.8916	0.0061	3	172	187	SGFEGPWTTNPLIFDK					Mascot
1808.8855	1808.8916	0.0061	3	172	187	SGFEGPWTTNPLIFDK	95	100			Mascot
2061.8826	2061.8958	0.0132	6	223	240	YAADEDAFFADYAEAHQK					Mascot
2061.8826	2061.8958	0.0132	6	223	240	YAADEDAFFADYAEAHQK	144	100			Mascot
2552.2451	2552.2847	0.0396	16	143	166	DVFYTMGLSDQDIVALSGGHTLGR					Mascot
2568.24	2568.2671	0.0271	11	143	166	DVFYTMGLSDQDIVALSGGHTLGR			Oxidation (M)[6]		Mascot
2568.24	2568.2671	0.0271	11	143	166	DVFYTMGLSDQDIVALSGGHTLGR	118	100	Oxidation (M)[6]		Mascot

35	triosephosphate isomerase, chloroplastic [Momordica charantia]	XP_022146193.1	32588.9	6.9	14	371	100	100

Peptide Information

Calc. Mass	Obsrv. Mass	± da	ppm	Start Seq.	End Seq.	Sequence	Ion Score	C. I. %	Modification	Rank	Result Type
954.4832	954.4999	0.0167	17	57	64	FFVGGNWK					Mascot
970.4662	970.4959	0.0297	31	47	56	GVFTMAGSGK			Oxidation (M)[5]		Mascot
1096.5898	1096.6113	0.0215	20	141	149	WVILGHSER					Mascot
1096.5898	1096.6113	0.0215	20	141	149	WVILGHSER	70	99.999			Mascot
1285.6246	1285.6433	0.0187	15	190	199	TFDVCFQQLK			Carbamidomethyl (C)[5]		Mascot

1361.7059	1361.7128	0.0069	5	110	121	IEISAQNSWVSK					Mascot
1371.6903	1371.7061	0.0158	12	151	162	HIIGEDDQFIGK					Mascot
1420.7319	1420.7584	0.0265	19	275	288	EDIDGFLVGGASLK					Mascot
1435.7428	1435.7616	0.0188	13	122	135	GGAFTGEISVEQLK					Mascot
1435.7428	1435.7616	0.0188	13	122	135	GGAFTGEISVEQLK	105	100			Mascot
1457.6995	1457.7501	0.0506	35	57	69	FFVGGNWKCNGTK					Mascot
1499.7853	1499.7675	-0.0178	-12	151	163	HIIGEDDQFIGKK					Mascot
1527.7915	1527.8002	0.0087	6	150	162	RHIIGEDDQFIGK					Mascot
1548.8268	1548.8469	0.0201	13	274	288	KEDIDGFLVGGASLK					Mascot
1548.8268	1548.8469	0.0201	13	274	288	KEDIDGFLVGGASLK	126	100			Mascot
1619.8785	1619.8754	-0.0031	-2	1	16	MAVVSTSLASQLSAVR					Mascot
1619.8785	1619.8754	-0.0031	-2	1	16	MAVVSTSLASQLSAVR					Mascot
1695.837	1695.8441	0.0071	4	257	273	IIYGGSVNGSNCAELAK					Mascot

36

ribulose-1,5-bisphosphate carboxylase/oxygenase large subunit, partial (chloroplast) [Momordica charantia]	AFH05588.1	18792.6	5.92	7	304	100	100

Peptide Information

Calc. Mass	Obsrv. Mass	± da	ppm	Start Seq.	End Seq.	Sequence	Ion Score	C. I. %	Modification	Rank	Result Type
922.4992	922.5403	0.0411	45	162	170	LGLSAKNYG					Mascot
1021.5312	1021.5552	0.024	23	17	25	DTDILAAFR					Mascot
1021.5312	1021.5552	0.024	23	17	25	DTDILAAFR	77	100			Mascot
1407.6678	1407.6924	0.0246	17	6	16	LTYYTPEYETK					Mascot
1407.6678	1407.6924	0.0246	17	6	16	LTYYTPEYETK	82	100			Mascot
1465.7546	1465.786	0.0314	21	131	143	TFQGPPHGIQVER					Mascot
1465.7546	1465.786	0.0314	21	131	143	TFQGPPHGIQVER	107	100			Mascot
1502.8512	1502.7584	-0.0928	-62	149	161	YGRPLLGCTIKPK			Carbamidomethyl (C)[8]		Mascot
2410.1814	2410.2292	0.0478	20	6	25	LTYYTPEYETKDTDILAAF R					Mascot
3854.8721	3855.0376	0.1655	43	26	63	VTPQPGVPPEEAGAAVA AESSTGTWTTVWTDGLT SLDR					Mascot

37

ribulose-1,5-bisphosphate carboxylase/oxygenase large subunit, partial (chloroplast) [Momordica charantia]	AFH05590.1	18059.3	5.5	5	282	100	100

Peptide Information

Calc. Mass	Obsrv. Mass	± da	ppm	Start Seq.	End Seq.	Sequence	Ion Score	C. I. %	Modification	Rank	Result Type
1021.5312	1021.5586	0.0274	27	17	25	DTDILAAFR					Mascot
1021.5312	1021.5586	0.0274	27	17	25	DTDILAAFR	71	100			Mascot
1407.6678	1407.6981	0.0303	22	6	16	LTYYTPEYETK					Mascot
1407.6678	1407.6981	0.0303	22	6	16	LTYYTPEYETK	63	99.996			Mascot
1427.8257	1427.7454	-0.0803	-56	119	130	LEDLRIPPAYIK					Mascot
1465.7546	1465.7893	0.0347	24	131	143	TFQGPPHGIQVER					Mascot
1465.7546	1465.7893	0.0347	24	131	143	TFQGPPHGIQVER	127	100			Mascot
2410.1814	2410.2268	0.0454	19	6	25	LTYYTPEYETKDTDILAAFR					Mascot

38

small heat shock protein, chloroplastic-like [Momordica charantia]	XP_022154035.1	13673.9	5.05	3	76	99.924	99.996

Peptide Information

Calc. Mass	Obsrv. Mass	± da	ppm	Start Seq.	End Seq.	Sequence	Ion Score	C. I. %	Modification	Rank	Result Type
1108.527	1108.5588	0.0318	29	28	36	EDNDALYLR					Mascot
1108.527	1108.5588	0.0318	29	28	36	EDNDALYLR	63	99.996			Mascot
1657.7349	1657.8198	0.0849	51	1	14	MDQFMEDPFLAASR					Mascot
1673.7299	1673.681	-0.0489	-29	1	14	MDQFMEDPFLAASR			Oxidation (M)[1]		Mascot
1983.915	1984.0088	0.0938	47	28	44	EDNDALYLRMDMPGLSK			Oxidation (M)[10]		Mascot

39

small heat shock protein, chloroplastic-like [Momordica charantia]	XP_022154035.1	13673.9	5.05	1	67	99.421	99.996

Peptide Information

Calc. Mass	Obsrv. Mass	± da	ppm	Start Seq.	End Seq.	Sequence	Ion Score	C. I. %	Modification	Rank	Result Type
1108.527	1108.5695	0.0425	38	28	36	EDNDALYLR					Mascot

1108.527	1108.5695	0.0425	38	28	36	EDNDALYLR	63	99.996			Mascot

40								
	2-Cys peroxiredoxin BAS1, chloroplastic [Momordica charantia]	XP_022144676.1	29551.3	7.66	10	442	100	100

Peptide Information

Calc. Mass	Obsrv. Mass	± da	ppm	Start Seq.	End Seq.	Sequence	Ion Score	C. I. %	Modification	Rank	Result Type
805.4818	805.4807	-0.0011	-1	200	206	GLFIIDK					Mascot
809.4403	809.4319	-0.0084	-10	101	107	LSEYIGK					Mascot
834.4316	834.4313	-0.0003	0	223	229	SVDETKR					Mascot
1501.8373	1501.8303	-0.007	-5	186	199	SYGVLIPDQGIALR					Mascot
1501.8373	1501.8303	-0.007	-5	186	199	SYGVLIPDQGIALR	137	100			Mascot
1620.8857	1620.7526	-0.1331	-82	40	54	SFVGLRSSFLPSAPR					Mascot
1735.9449	1735.9358	-0.0091	-5	207	222	EGIIQHSTINNLAIGR					Mascot
1735.9449	1735.9358	-0.0091	-5	207	222	EGIIQHSTINNLAIGR	122	100			Mascot
1748.9066	1748.8732	-0.0334	-19	165	181	SGGLGDLQYPLVSDVTK					Mascot
1748.9066	1748.8732	-0.0334	-19	165	181	SGGLGDLQYPLVSDVTK	71	100			Mascot
1877.0016	1876.9523	-0.0493	-26	164	181	KSGGLGDLQYPLVSDVT K					Mascot
1877.0016	1876.9523	-0.0493	-26	164	181	KSGGLGDLQYPLVSDVT K	28	85.031			Mascot
2522.4089	2522.4243	0.0154	6	200	222	GLFIIDKEGIIQHSTINNLAI GR					Mascot
2522.4089	2522.4243	0.0154	6	200	222	GLFIIDKEGIIQHSTINNLAI GR	38	98.657			Mascot
2857.3826	2857.3992	0.0166	6	230	254	TLQALQYVQENPDEVCP AGWKPGEK			Carbamidomethyl (C)[16]		Mascot

41								
	ribulose-1,5-bisphosphate carboxylase/oxygenase large subunit, partial (chloroplast) [Momordica charantia]	CCD31477.1	5340.7	6.07	2	140	100	100

Protein Group

ribulose-1,5-bisphosphate carboxylase/oxygenase large	CCD31475.1	5340.7	#####

subunit, partial (chloroplast) [Momordica charantia]			##### 6138
ribulose-1,5-bisphosphate carboxylase/oxygenase large subunit, partial (chloroplast) [Momordica charantia]	CCD31473.1	5340.7	##### ##### 6138
ribulose-1,5-bisphosphate carboxylase/oxygenase large subunit, partial (chloroplast) [Momordica charantia]	CCD31471.1	5340.7	##### ##### 6138
ribulose-1,5-bisphosphate carboxylase/oxygenase large subunit, partial (chloroplast) [Momordica charantia]	CCD31479.1	5340.7	##### ##### 6138

Peptide Information

Calc. Mass	Obsrv. Mass	± da	ppm	Start Seq.	End Seq.	Sequence	Ion Score	C. I. %	Modification	Rank	Result Type
1021.5312	1021.5266	-0.0046	-5	33	41	DTDILAAFR					Mascot
1021.5312	1021.5266	-0.0046	-5	33	41	DTDILAAFR	70	100			Mascot
1407.6678	1407.6483	-0.0195	-14	22	32	LTYYTPEYETK					Mascot
1407.6678	1407.6483	-0.0195	-14	22	32	LTYYTPEYETK	57	99.983			Mascot

42	LOW QUALITY PROTEIN: 17.9 kDa class II heat shock protein-like [Momordica charantia]	XP_022135183.1	18615.8	5.89	6	280	100	100

Peptide Information

Calc. Mass	Obsrv. Mass	± da	ppm	Start Seq.	End Seq.	Sequence	Ion Score	C. I. %	Modification	Rank	Result Type
800.4876	800.4439	-0.0437	-55	161	167	IVEVKVN					Mascot
1004.5081	1004.575	0.0669	67	51	60	AMAATPADIK			Oxidation (M)[2]		Mascot
1004.5081	1004.575	0.0669	67	51	60	AMAATPADIK			Oxidation (M)[2]		Mascot
1857.9092	1857.8846	-0.0246	-13	61	76	EYPNSYVFVVDMPGLK					Mascot
1857.9092	1857.8846	-0.0246	-13	61	76	EYPNSYVFVVDMPGLK	86	100			Mascot
1873.9042	1873.8755	-0.0287	-15	61	76	EYPNSYVFVVDMPGLK			Oxidation (M)[12]		Mascot
1873.9042	1873.8755	-0.0287	-15	61	76	EYPNSYVFVVDMPGLK	89	100	Oxidation (M)[12]		Mascot
2198.1663	2198.1211	-0.0452	-21	77	96	VGDIKVQVEDDNVLLISGER					Mascot

Calc. Mass	Obsrv. Mass	± da	ppm	Start Seq.	End Seq.	Sequence	Ion Score	C. I. %	Modification	Rank	Result Type
2198.1663	2198.1211	-0.0452	-21	77	96	VGDIKVQVEDDNVLLISGER	161	100			Mascot
2889.4663	2889.4297	-0.0366	-13	124	150	FVLPENANTDAISAVCQDGVLTVTVQK			Carbamidomethyl (C)[16]		Mascot
3017.5613	3017.5491	-0.0122	-4	123	150	KFVLPENANTDAISAVCQDGVLTVTVQK			Carbamidomethyl (C)[17]		Mascot

43

oxygen-evolving enhancer protein 2, chloroplastic [Momordica charantia]	XP_022136521.1	28154.3	8.61	7	110	100	100

Peptide Information

Calc. Mass	Obsrv. Mass	± da	ppm	Start Seq.	End Seq.	Sequence	Ion Score	C. I. %	Modification	Rank	Result Type
807.4611	807.4512	-0.0099	-12	163	169	VDYLLGK					Mascot
849.3585	849.3562	-0.0023	-3	214	222	TADGDEGGK					Mascot
945.5152	945.5113	-0.0039	-4	120	127	EFPGQVLR					Mascot
945.5152	945.5113	-0.0039	-4	120	127	EFPGQVLR	50	99.898			Mascot
1230.6589	1230.6499	-0.009	-7	118	127	EREFPGQVLR					Mascot
1230.6589	1230.6499	-0.009	-7	118	127	EREFPGQVLR					Mascot
1299.658	1299.6407	-0.0173	-13	253	265	KFVEGAASSFSVA					Mascot
2285.0569	2285.0171	-0.0398	-17	128	147	YEDNFDSNSNLSVIINPTDK					Mascot
2413.1519	2413.1145	-0.0374	-15	128	148	YEDNFDSNSNLSVIINPTDKK					Mascot
2413.1519	2413.1145	-0.0374	-15	128	148	YEDNFDSNSNLSVIINPTDKK	35	96.521			Mascot

44

ribulose-1,5-bisphosphate carboxylase/oxygenase large subunit, partial (chloroplast) [Momordica charantia]	AFH05588.1	18792.6	5.92	5	249	100	100

Protein Group

ribulose-1,5-bisphosphate carboxylase/oxygenase large subunit, partial (chloroplast) [Momordica charantia]	AFH05589.1	19019.8	##### ##### 0835
ribulose-1,5-bisphosphate carboxylase/oxygenase large	AFH05590.1	18059.3	5.5

subunit, partial (chloroplast) [Momordica charantia]

Peptide Information

Calc. Mass	Obsrv. Mass	± da	ppm	Start Seq.	End Seq.	Sequence	Ion Score	C. I. %	Modification	Rank	Result Type
1021.5312	1021.5197	-0.0115	-11	17	25	DTDILAAFR					Mascot
1021.5312	1021.5197	-0.0115	-11	17	25	DTDILAAFR	62	99.994			Mascot
1407.6678	1407.6469	-0.0209	-15	6	16	LTYYTPEYETK					Mascot
1407.6678	1407.6469	-0.0209	-15	6	16	LTYYTPEYETK	70	99.999			Mascot
1445.8297	1445.787	-0.0427	-30	149	161	YGRPLLGCTIKPK					Mascot
1465.7546	1465.7321	-0.0225	-15	131	143	TFQGPPHGIQVER					Mascot
1465.7546	1465.7321	-0.0225	-15	131	143	TFQGPPHGIQVER	93	100			Mascot
1502.8512	1502.8226	-0.0286	-19	149	161	YGRPLLGCTIKPK			Carbamidomethyl (C)[8]		Mascot
1502.8512	1502.8226	-0.0286	-19	149	161	YGRPLLGCTIKPK			Carbamidomethyl (C)[8]		Mascot
3854.8721	3854.8901	0.018	5	26	63	VTPQPGVPPEEAGAAVA AESSTGTWTTVWTDGLT SLDR					Mascot

45

fructose-bisphosphate aldolase, cytoplasmic isozyme 1 [Momordica charantia] XP_022139399.1 37974.9 6.38 7 155 100 100

Peptide Information

Calc. Mass	Obsrv. Mass	± da	ppm	Start Seq.	End Seq.	Sequence	Ion Score	C. I. %	Modification	Rank	Result Type
816.3708	816.3594	-0.0114	-14	130	135	CQQFYK					Mascot
888.5149	888.4905	-0.0244	-27	299	306	ALQQSTLK					Mascot
1164.6259	1164.5123	-0.1136	-98	8	17	YADELIKNAK					Mascot
1479.7876	1479.733	-0.0546	-37	197	210	CAAATEIVLAAVYK			Carbamidomethyl (C)[1]		Mascot
1587.8741	1587.8457	-0.0284	-18	238	252	VAPEVVAEYTVAALR					Mascot
1587.8741	1587.8457	-0.0284	-18	238	252	VAPEVVAEYTVAALR	135	100			Mascot
1743.9752	1743.9365	-0.0387	-22	238	253	VAPEVVAEYTVAALRR					Mascot
2798.4871	2798.4294	-0.0577	-21	211	237	ALSDHHVLLEGTLLKPNM VTPGSGSPK					Mascot
2814.4819	2814.4153	-0.0666	-24	211	237	ALSDHHVLLEGTLLKPNM VTPGSGSPK			Oxidation (M)[18]		Mascot

46

leghemoglobin reductase [Momordica charantia] XP_022137754.1 53565.9 7.68 12 144 100 100

Peptide Information

Calc. Mass	Obsrv. Mass	± da	ppm	Start Seq.	End Seq.	Sequence	Ion Score	C. I. %	Modification	Rank	Result Type
969.4611	969.4463	-0.0148	-15	420	427	FPFMANSR					Mascot
980.506	980.493	-0.013	-13	105	113	HAFANHGVK					Mascot
985.456	985.4406	-0.0154	-16	420	427	FPFMANSR			Oxidation (M)[4]		Mascot
1072.5027	1072.5774	0.0747	70	1	9	MAMSSFARR			Oxidation (M)[1]		Mascot
1459.725	1459.6998	-0.0252	-17	495	507	EAAMATYDKPIHI					Mascot
1480.7036	1480.7084	0.0048	3	482	494	VCHAHPTMSEALK			Carbamidomethyl (C)[2]		Mascot
1496.6985	1496.6671	-0.0314	-21	482	494	VCHAHPTMSEALK			Carbamidomethyl (C)[2], Oxidation (M)[8]		Mascot
1523.7423	1523.7306	-0.0117	-8	92	104	ALLHSSHMYHEAK					Mascot
1539.7373	1539.705	-0.0323	-21	92	104	ALLHSSHMYHEAK			Oxidation (M)[8]		Mascot
1594.7418	1594.7166	-0.0252	-16	365	379	AEEDGVACVEFIAGK			Carbamidomethyl (C)[8]		Mascot
1594.7418	1594.7166	-0.0252	-16	365	379	AEEDGVACVEFIAGK	49	99.904	Carbamidomethyl (C)[8]		Mascot
1633.8037	1633.7706	-0.0331	-20	76	91	GTLGGTCLNVGCIPSK			Carbamidomethyl (C)[7,12]		Mascot
1652.8022	1652.7672	-0.035	-21	114	128	FSSVEVDLPAMMAQK					Mascot
1668.7972	1668.7642	-0.033	-20	114	128	FSSVEVDLPAMMAQK			Oxidation (M)[11]		Mascot
1684.7921	1684.7463	-0.0458	-27	114	128	FSSVEVDLPAMMAQK			Oxidation (M)[11,12]		Mascot
1684.7921	1684.7463	-0.0458	-27	114	128	FSSVEVDLPAMMAQK	5	0	Oxidation (M)[11,12]		Mascot
1789.9048	1789.8582	-0.0466	-26	75	91	RGTLGGTCLNVGCIPSK			Carbamidomethyl (C)[8,13]		Mascot
2510.1504	2510.2249	0.0745	30	365	387	AEEDGVACVEFIAGKTGH VDYDK			Carbamidomethyl (C)[8]		Mascot
2554.2937	2554.26	-0.0337	-13	380	403	TGHVDYDKVPGVVYTHP EVASVGK					Mascot
2554.2937	2554.26	-0.0337	-13	380	403	TGHVDYDKVPGVVYTHP EVASVGK	52	99.949			Mascot

47

serine hydroxymethyltransferase 2, mitochondrial [Momordica charantia] XP_022131877.1 56947.3 8.59 17 408 100 100

Peptide Information

Calc. Mass	Obsrv. Mass	± da	ppm	Start Seq.	End Seq.	Sequence	Ion Score	C. I. %	Modification	Rank	Result Type
800.3937	800.3866	-0.0071	-9	234	239	LYDYAR					Mascot

841.4389	841.4309	-0.008	-10	290	296	GAMIFFR				Mascot
857.4338	857.4203	-0.0135	-16	290	296	GAMIFFR			Oxidation (M)[3]	Mascot
933.4822	933.4613	-0.0209	-22	428	436	MGTPALTSR				Mascot
937.4625	937.4322	-0.0303	-32	343	350	QATTPEYK				Mascot
949.4771	949.4545	-0.0226	-24	428	436	MGTPALTSR			Oxidation (M)[1]	Mascot
999.453	999.4304	-0.0226	-23	99	107	YSEGYPGAR				Mascot
999.453	999.4304	-0.0226	-23	99	107	YSEGYPGAR	20	7.841		Mascot
1066.5569	1066.5669	0.01	9	499	507	QFPTIGFEK				Mascot
1066.5569	1066.5669	0.01	9	499	507	QFPTIGFEK	34	96.373		Mascot
1091.6207	1091.6011	-0.0196	-18	223	233	LIVAGASAYAR				Mascot
1091.6207	1091.6011	-0.0196	-18	223	233	LIVAGASAYAR	38	98.256		Mascot
1180.6685	1180.6067	-0.0618	-52	401	411	VLESVHIAANK				Mascot
1358.6111	1358.6456	0.0345	25	306	316	GQEVLYDYEDK				Mascot
1569.8054	1569.7697	-0.0357	-23	412	427	NTVPGDVSAMVPGGIR				Mascot
1569.8054	1569.7697	-0.0357	-23	412	427	NTVPGDVSAMVPGGIR	52	99.936		Mascot
1585.8003	1585.757	-0.0433	-27	412	427	NTVPGDVSAMVPGGIR			Oxidation (M)[10]	Mascot
1585.8003	1585.757	-0.0433	-27	412	427	NTVPGDVSAMVPGGIR	88	100	Oxidation (M)[10]	Mascot
1660.8403	1660.8091	-0.0312	-19	186	199	ISAVSIFFETMPYR				Mascot
1676.8353	1676.793	-0.0423	-25	186	199	ISAVSIFFETMPYR			Oxidation (M)[11]	Mascot
1676.8353	1676.793	-0.0423	-25	186	199	ISAVSIFFETMPYR	4	0	Oxidation (M)[11]	Mascot
1804.9303	1804.8801	-0.0502	-28	185	199	KISAVSIFFETMPYR			Oxidation (M)[12]	Mascot
1816.8236	1816.7786	-0.045	-25	200	214	LDESTGYIDYDQLER				Mascot
1816.8236	1816.7786	-0.045	-25	200	214	LDESTGYIDYDQLER	150	100		Mascot
2040.8678	2040.8157	-0.0521	-26	108	124	YYGGNEYIDMAESLCQK			Carbamidomethyl (C)[15]	Mascot
2056.8628	2056.7952	-0.0676	-33	108	124	YYGGNEYIDMAESLCQK			Carbamidomethyl (C)[15], Oxidation (M)[10]	Mascot

2291.1238	2291.0686	-0.0552	-24	164	184	IMALDLPHGGHLSHGYQ TDTK				Mascot
2307.1187	2307.0474	-0.0713	-31	164	184	IMALDLPHGGHLSHGYQ TDTK			Oxidation (M)[2]	Mascot
2316.0925	2316.0647	-0.0278	-12	343	362	QATTPEYKAYQEQVLSN CSR				Mascot
2435.2136	2435.1499	-0.0637	-26	164	185	IMALDLPHGGHLSHGYQ TDTKK			Oxidation (M)[2]	Mascot

48								
thylakoid lumenal 29 kDa protein, chloroplastic isoform X2 [Momordica charantia]	XP_022133457.1	30739.7	5.68	8	88	99.996		99.984

Peptide Information

Calc. Mass	Obsrv. Mass	± da	ppm	Start Seq.	End Seq.	Sequence	Ion Score	C. I. %	Modification	Rank	Result Type
844.4271	844.4218	-0.0053	-6	64	72	SGGPNGSIR					Mascot
965.5414	965.5253	-0.0161	-17	124	132	STFLASAIR					Mascot
965.5414	965.5253	-0.0161	-17	124	132	STFLASAIR	39	98.941			Mascot
1143.4913	1143.4722	-0.0191	-17	164	174	SDAEAPDPEGR					Mascot
1143.4913	1143.4722	-0.0191	-17	164	174	SDAEAPDPEGR	18	0			Mascot
2056.0332	2055.9746	-0.0586	-29	236	253	ETVSQTDYEVDLITTLTK					Mascot
2074.0491	2073.9966	-0.0525	-25	254	271	ISSLGQQINYEAYTYPVK					Mascot
2105.0088	2104.9646	-0.0442	-21	141	159	GNLLYTAYGSSGQWGLFDR					Mascot
2171.1025	2170.958	-0.1445	-67	2	20	VSSHQMVEPAYAADLIQRR					Mascot
2299.1665	2299.1179	-0.0486	-21	234	253	SRETVSQTDYEVDLITTLTK					Mascot

49								
peptide methionine sulfoxide reductase A1-like [Momordica charantia]	XP_022141289.1	29239.6	8.87	6	170	100		100

Peptide Information

Calc. Mass	Obsrv. Mass	± da	ppm	Start Seq.	End Seq.	Sequence	Ion Score	C. I. %	Modification	Rank	Result Type
831.3777	831.3558	-0.0219	-26	254	260	GCNDPIR			Carbamidomethyl (C)[2]		Mascot
1137.5283	1137.4954	-0.0329	-29	180	189	QGNDVGTQYR					Mascot
1437.6646	1437.6165	-0.0481	-33	231	241	AEEYHQQYLEK					Mascot
1437.6646	1437.6165	-0.0481	-33	231	241	AEEYHQQYLEK	58	99.987			Mascot
1461.6897	1461.6306	-0.0591	-40	190	201	SGIYFYTPEQEK					Mascot
1461.6897	1461.6306	-0.0591	-40	190	201	SGIYFYTPEQEK	68	99.999			Mascot
1746.7792	1746.7336	-0.0456	-26	158	171	ECSYESLLDAFWAR			Carbamidomethyl (C)[2]		Mascot
1746.7792	1746.7336	-0.0456	-26	158	171	ECSYESLLDAFWAR	25	71.76	Carbamidomethyl (C)[2]		Mascot
1759.865	1759.7848	-0.0802	-46	190	204	SGIYFYTPEQEKAAR					Mascot

50

nucleoside diphosphate kinase [Momordica charantia] XP_022152788.1 16378.5 6.31 5 127 100 100

Peptide Information

Calc. Mass	Obsrv. Mass	± da	ppm	Start Seq.	End Seq.	Sequence	Ion Score	C. I. %	Modification	Rank	Result Type
943.5571	943.5392	-0.0179	-19	16	24	GLVGEIISR					Mascot
943.5571	943.5392	-0.0179	-19	16	24	GLVGEIISR	13	0			Mascot
949.4738	949.4574	-0.0164	-17	103	111	GDFAIDVGR					Mascot
949.4738	949.4574	-0.0164	-17	103	111	GDFAIDVGR	51	99.927			Mascot
1384.6815	1384.6587	-0.0228	-16	112	124	NIIHGSDSVESAR					Mascot
1384.6815	1384.6587	-0.0228	-16	112	124	NIIHGSDSVESAR	40	99.041			Mascot
1663.8473	1663.8037	-0.0436	-26	2	15	EQSFIMIKPDGVQR			Oxidation (M)[6]		Mascot
1810.8827	1810.8362	-0.0465	-26	1	15	MEQSFIMIKPDGVQR			Oxidation (M)[1,7]		Mascot

51

oxygen-evolving enhancer protein 2, chloroplastic [Momordica charantia] XP_022136521.1 28154.3 8.61 7 189 100 100

Peptide Information

Calc. Mass	Obsrv. Mass	± da	ppm	Start Seq.	End Seq.	Sequence	Ion Score	C. I. %	Modification	Rank	Result Type
807.4611	807.4507	-0.0104	-13	163	169	VDYLLGK					Mascot
945.5152	945.5046	-0.0106	-11	120	127	EFPGQVLR					Mascot
945.5152	945.5046	-0.0106	-11	120	127	EFPGQVLR	48	99.858			Mascot
1230.6589	1230.6437	-0.0152	-12	118	127	EREFPGQVLR					Mascot
1299.658	1299.5592	-0.0988	-76	253	265	KFVEGAASSFSVA					Mascot
2154.0332	2154.0205	-0.0127	-6	1	20	MASTACFLHHHALTTAAAR			Carbamidomethyl (C)[6], Oxidation (M)[1]		Mascot
2285.0569	2285.0186	-0.0383	-17	128	147	YEDNFDSNSNLSVIINPTDK					Mascot
2413.1519	2413.1101	-0.0418	-17	128	148	YEDNFDSNSNLSVIINPTDKK					Mascot
2413.1519	2413.1101	-0.0418	-17	128	148	YEDNFDSNSNLSVIINPTDKK	115	100			Mascot

52

ribulose-1,5-bisphosphate carboxylase/oxygenase large subunit, partial (chloroplast) [Momordica charantia]	ABG24929.1	46428.5	6.31	8	149	100	100

Peptide Information

Calc. Mass	Obsrv. Mass	± da	ppm	Start Seq.	End Seq.	Sequence	Ion Score	C. I. %	Modification	Rank	Result Type
830.4366	830.4368	0.0002	0	395	401	EGNEIIR					Mascot
914.405	914.3942	-0.0108	-12	261	267	NHGMHFR			Oxidation (M)[4]		Mascot
1059.5615	1059.5522	-0.0093	-9	377	386	VALEACVQAR					Mascot
1116.583	1116.5741	-0.0089	-8	377	386	VALEACVQAR			Carbamidomethyl (C)[6]		Mascot
1116.583	1116.5741	-0.0089	-8	377	386	VALEACVQAR	39	98.598	Carbamidomethyl (C)[6]		Mascot
1154.5636	1154.4771	-0.0865	-75	259	267	QKNHGMHFR					Mascot
1459.7402	1459.679	-0.0612	-42	171	182	DRFLFCAEAIFK					Mascot
1465.7546	1465.7052	-0.0494	-34	102	114	TFQGPPHGIQVER					Mascot
1516.7617	1516.7148	-0.0469	-31	171	182	DRFLFCAEAIFK			Carbamidomethyl (C)[6]		Mascot
1546.736	1546.7124	-0.0236	-15	406	418	WSPELAAACEVWK			Carbamidomethyl (C)[9]		Mascot
1546.736	1546.7124	-0.0236	-15	406	418	WSPELAAACEVWK	91	100	Carbamidomethyl (C)[9]		Mascot
1572.7911	1572.7014	-0.0897	-57	377	390	VALEACVQARNEGR			Carbamidomethyl (C)[6]		Mascot

53

ribulose-1,5-bisphosphate carboxylase/oxygenase large subunit, partial (chloroplast) [Momordica charantia]	ABG24929.1	46428.5	6.31	8	147	100	100

Peptide Information

Calc. Mass	Obsrv. Mass	± da	ppm	Start Seq.	End Seq.	Sequence	Ion Score	C. I. %	Modification	Rank	Result Type
830.4366	830.4335	-0.0031	-4	395	401	EGNEIIR					Mascot
914.405	914.3935	-0.0115	-13	261	267	NHGMHFR			Oxidation (M)[4]		Mascot
1059.5615	1059.5474	-0.0141	-13	377	386	VALEACVQAR					Mascot
1116.583	1116.571	-0.012	-11	377	386	VALEACVQAR			Carbamidomethyl (C)[6]		Mascot
1116.583	1116.571	-0.012	-11	377	386	VALEACVQAR	59	99.988	Carbamidomethyl (C)[6]		Mascot
1465.7546	1465.7002	-0.0544	-37	102	114	TFQGPPHGIQVER					Mascot
1516.7617	1516.7463	-0.0154	-10	171	182	DRFLFCAEAIFK			Carbamidomethyl (C)[6]		Mascot
1546.736	1546.7113	-0.0247	-16	406	418	WSPELAAACEVWK			Carbamidomethyl (C)[9]		Mascot
1546.736	1546.7113	-0.0247	-16	406	418	WSPELAAACEVWK	68	99.999	Carbamidomethyl (C)[9]		Mascot

1572.7911	1572.7007	-0.0904	-57	377	390	VALEACVQARNEGR			Carbamidomethyl (C)[6]		Mascot
1708.8766	1708.8367	-0.0399	-23	102	116	TFQGPPHGIQVERDK					Mascot

54	LOW QUALITY PROTEIN: DNA polymerase alpha catalytic subunit-like [Momordica charantia]	XP_022156119.1	138074.1	8.62	26	44	0	0

Peptide Information

Calc. Mass	Obsrv. Mass	± da	ppm	Start Seq.	End Seq.	Sequence	Ion Score	C. I. %	Modification	Rank	Result Type
828.4039	828.3303	-0.0736	-89	###	1065	FWSNFK					Mascot
830.4804	830.4388	-0.0416	-50	###	1142	KEILCPK					Mascot
844.4709	844.4529	-0.018	-21	166	173	IGAMPISR					Mascot
1020.5295	1020.5178	-0.0117	-11	817	824	RDWCLLSK					Mascot
1023.5655	1023.4637	-0.1018	-99	791	799	YAAVKLQCK					Mascot
1094.5188	1094.4932	-0.0256	-23	522	530	FEVTVDCPK			Carbamidomethyl (C)[7]		Mascot
1116.5466	1116.5742	0.0276	25	274	283	NEPVCTLNAR					Mascot
1116.5466	1116.5742	0.0276	25	274	283	NEPVCTLNAR					Mascot
1156.549	1156.459	-0.09	-78	###	1231	DIAVSLHMAPC					Mascot
1289.7576	1289.7039	-0.0537	-42	861	871	GQVALEKYIIR					Mascot
1289.7576	1289.7039	-0.0537	-42	861	871	GQVALEKYIIR	9	0			Mascot
1297.6747	1297.6954	0.0207	16	###	1058	QERPVVDEPTK					Mascot
1334.7467	1334.6622	-0.0845	-63	578	589	AKIPYSFPLNGK					Mascot
1334.7467	1334.6622	-0.0845	-63	578	589	AKIPYSFPLNGK					Mascot
1417.8022	1417.6803	-0.1219	-86	886	897	NQPHVQVAQRLK					Mascot
1424.6808	1424.6697	-0.0111	-8	###	1097	GRMTCGMISNQVK					Mascot
1470.7444	1470.6843	-0.0601	-41	###	1231	LKDIAVSLHMAPC			Carbamidomethyl (C)[13], Oxidation (M)[10]		Mascot
1481.7021	1481.6863	-0.0158	-11	###	1097	GRMTCGMISNQVK			Carbamidomethyl (C)[5]		Mascot
1481.7021	1481.6863	-0.0158	-11	###	1097	GRMTCGMISNQVK			Carbamidomethyl (C)[5]		Mascot
1487.6559	1487.6879	0.032	22	###	1070	FWSNFKCPNCK			Carbamidomethyl (C)[7,10]		Mascot
1507.692	1507.703	0.011	7	686	699	LTANSMYGCLGFSK			Oxidation (M)[6]		Mascot
1520.7964	1520.6775	-0.1189	-78	###	1026	YLDCKPLVLTCPR					Mascot
1548.7185	1548.7129	-0.0056	-4	686	699	LTANSMYGCLGFSK			Carbamidomethyl (C)[9]		Mascot
1564.7135	1564.7035	-0.01	-6	686	699	LTANSMYGCLGFSK			Carbamidomethyl (C)[9], Oxidation (M)[6]		Mascot
1578.7404	1578.6908	-0.0496	-31	###	1149	EILCPKYPQCDGR			Carbamidomethyl (C)[4]		Mascot
1578.7404	1578.6908	-0.0496	-31	###	1149	EILCPKYPQCDGR			Carbamidomethyl (C)[4]		Mascot

1604.7924	1604.7188	-0.0736	-46	355	369	VKAGDPYHSCCVVVK					Mascot
1626.7404	1626.6609	-0.0795	-49	###	1174	QICYFCHVLDTER					Mascot
1661.8138	1661.7035	-0.1103	-66	355	369	VKAGDPYHSCCVVVK			Carbamidomethyl (C)[10]		Mascot
1792.9878	1792.8347	-0.1531	-85	425	440	LLDLNVSTFSMTPVKK					Mascot
1804.9044	1804.8606	-0.0438	-24	562	577	LNVNEIVSASVICCQR			Carbamidomethyl (C)[13]		Mascot
1820.9939	1820.8716	-0.1223	-67	424	439	RLLDLNVSTFSMTPVK					Mascot
1941.0276	1940.9089	-0.1187	-61	590	604	QRPPTFWNPILERMR					Mascot
2020.915	2020.9706	0.0556	28	357	373	AGDPYHSCCVVVKNMQ R			Carbamidomethyl (C)[8,9]		Mascot
2238.969	2239.0847	0.1157	52	###	1120	FIAKYYHGLMMCDEETC K			Carbamidomethyl (C)[12]		Mascot

55

ribulose-1,5-bisphosphate carboxylase/oxygenase large subunit, partial (chloroplast) [Momordica charantia]	CCD31477.1	5340.7	6.07	4	177	100		100

Protein Group

ribulose-1,5-bisphosphate carboxylase/oxygenase large subunit, partial (chloroplast) [Momordica charantia]	CCD31475.1	5340.7	##### ##### 6138
ribulose-1,5-bisphosphate carboxylase/oxygenase large subunit, partial (chloroplast) [Momordica charantia]	CCD31473.1	5340.7	##### ##### 6138
ribulose-1,5-bisphosphate carboxylase/oxygenase large subunit, partial (chloroplast) [Momordica charantia]	CCD31471.1	5340.7	##### ##### 6138
ribulose-1,5-bisphosphate carboxylase/oxygenase large subunit, partial (chloroplast) [Momordica charantia]	CCD31479.1	5340.7	##### ##### 6138

Peptide Information

Calc. Mass	Obsrv. Mass	± da	ppm	Start Seq.	End Seq.	Sequence	Ion Score	C. I. %	Modification	Rank	Result Type
1021.5312	1021.5263	-0.0049	-5	33	41	DTDILAAFR					Mascot
1021.5312	1021.5263	-0.0049	-5	33	41	DTDILAAFR	73	100			Mascot

Calc. Mass	Obsrv. Mass	± da	ppm	Start Seq.	End Seq.	Sequence	Ion Score	C. I. %	Modification	Rank	Result Type
1407.6678	1407.6484	-0.0194	-14	22	32	LTYYTPEYETK					Mascot
1407.6678	1407.6484	-0.0194	-14	22	32	LTYYTPEYETK	72	100			Mascot
1699.9014	1699.8051	-0.0963	-57	33	48	DTDILAAFRVTPQPGV					Mascot
2410.1814	2410.1687	-0.0127	-5	22	41	LTYYTPEYETKDTDILAAFR					Mascot

56

plastocyanin, partial [Momordica charantia] XP_022137655.1 11734.7 4.26 3 87 99.995 100

Peptide Information

Calc. Mass	Obsrv. Mass	± da	ppm	Start Seq.	End Seq.	Sequence	Ion Score	C. I. %	Modification	Rank	Result Type
1932.8368	1932.8446	0.0078	4	92	109	GSYSFYCSPHQGAGMVGK			Carbamidomethyl (C)[7]		Mascot
1948.8317	1948.8159	-0.0158	-8	92	109	GSYSFYCSPHQGAGMVGK			Carbamidomethyl (C)[7], Oxidation (M)[15]		Mascot
1948.8317	1948.8159	-0.0158	-8	92	109	GSYSFYCSPHQGAGMVGK	71	100	Carbamidomethyl (C)[7], Oxidation (M)[15]		Mascot
2305.0376	2305.0903	0.0527	23	92	113	GSYSFYCSPHQGAGMVGKVTVN			Oxidation (M)[15]		Mascot
2685.3408	2685.3298	-0.011	-4	19	44	LGGDDGSLAFVPNDFSISSGDKIVFK					Mascot

57

malate dehydrogenase, mitochondrial [Momordica charantia] XP_022142854.1 35421.8 8.71 9 283 100 100

Peptide Information

Calc. Mass	Obsrv. Mass	± da	ppm	Start Seq.	End Seq.	Sequence	Ion Score	C. I. %	Modification	Rank	Result Type
1219.7046	1219.7153	0.0107	9	170	180	LFGVTTLDVVR					Mascot
1219.7046	1219.7153	0.0107	9	170	180	LFGVTTLDVVR	59	99.989			Mascot
1318.7002	1318.7072	0.007	5	115	126	DDLFNINAGIVK					Mascot
1318.7002	1318.7072	0.007	5	115	126	DDLFNINAGIVK	18	0			Mascot
1347.7996	1347.8098	0.0102	8	169	180	KLFGVTTLDVVR					Mascot
1347.7996	1347.8098	0.0102	8	169	180	KLFGVTTLDVVR	22	48.875			Mascot
1795.051	1795.026	-0.025	-14	31	49	VAVLGAAGGIGQPLSLLM					Mascot

Calc. Mass	Obsrv. Mass	± da	ppm	Start Seq.	End Seq.	Sequence	Ion Score	C. I. %	Modification	Rank	Result Type
						K					
1818.912	1818.9042	-0.0078	-4	301	317	NGVESVLDLGPLSDFEK					Mascot
1923.146	1923.1169	-0.0291	-15	30	49	KVAVLGAAGGIGQPLSLLMK					Mascot
2175.1843	2175.3503	0.166	76	115	134	DDLFNINAGIVKSLCIAIAK			Carbamidomethyl (C)[15]		Mascot
2210.1565	2210.1763	0.0198	9	57	78	LALYDIAGTPGVAADVGHVNTR					Mascot
2210.1565	2210.1763	0.0198	9	57	78	LALYDIAGTPGVAADVGHVNTR	150	100			Mascot
2274.1257	2274.072	-0.0537	-24	246	268	AGKGSATLSMAYAGALFADACLK			Carbamidomethyl (C)[21]		Mascot
2290.1206	2290.1763	0.0557	24	246	268	AGKGSATLSMAYAGALFADACLK			Carbamidomethyl (C)[21], Oxidation (M)[10]		Mascot

58

fructose-bisphosphate aldolase 1, chloroplastic [Momordica charantia]	XP_022146084.1	42808.1	6.86	8	202	100	100

Peptide Information

Calc. Mass	Obsrv. Mass	± da	ppm	Start Seq.	End Seq.	Sequence	Ion Score	C. I. %	Modification	Rank	Result Type
855.5046	855.4545	-0.0501	-59	26	33	QPAVSVVR					Mascot
873.4577	873.4739	0.0162	19	202	209	EAAWGLAR					Mascot
1098.5327	1098.549	0.0163	15	172	181	AAAYYQQGAR					Mascot
1098.5327	1098.549	0.0163	15	172	181	AAAYYQQGAR	69	99.999			Mascot
1387.7175	1387.7301	0.0126	9	82	94	LASIGLENTEANR					Mascot
1452.8422	1452.838	-0.0042	-3	187	201	TVVSIPNGPSALAVK					Mascot
1543.8187	1543.8274	0.0087	6	81	94	RLASIGLENTEANR					Mascot
2363.1653	2363.1663	0.001	0	110	130	YISGAILFEETLYQSTVDGEK					Mascot
2427.1721	2427.1995	0.0274	11	149	171	GLVPLPGSNNESWCQGLDGLASR			Carbamidomethyl (C)[14]		Mascot
2427.1721	2427.1995	0.0274	11	149	171	GLVPLPGSNNESWCQGLDGLASR	111	100	Carbamidomethyl (C)[14]		Mascot

59

fructose-bisphosphate aldolase 1, chloroplastic [Momordica charantia]	XP_022146084.1	42808.1	6.86	12	225	100	100

Peptide Information

Calc. Mass	Obsrv. Mass	± da	ppm	Start Seq.	End Seq.	Sequence	Ion Score	C. I. %	Modification	Rank	Result Type
822.4355	822.4471	0.0116	14	238	244	TFEVAQK					Mascot
855.5046	855.4651	-0.0395	-46	26	33	QPAVSVVR					Mascot
873.4577	873.4812	0.0235	27	202	209	EAAWGLAR					Mascot
901.5101	901.4691	-0.041	-45	368	376	ANSLAQLGK					Mascot
947.4979	947.5099	0.012	13	339	346	ALQNTCLK			Carbamidomethyl (C)[6]		Mascot
1098.5327	1098.5555	0.0228	21	172	181	AAAYYQQGAR					Mascot
1098.5327	1098.5555	0.0228	21	172	181	AAAYYQQGAR	49	99.898			Mascot
1387.7175	1387.7427	0.0252	18	82	94	LASIGLENTEANR					Mascot
1387.7175	1387.7427	0.0252	18	82	94	LASIGLENTEANR	55	99.971			Mascot
1452.8422	1452.845	0.0028	2	187	201	TVVSIPNGPSALAVK					Mascot
1466.6614	1466.7114	0.05	34	67	80	GILAMDESNATCGK			Carbamidomethyl (C)[12]		Mascot
1482.6564	1482.6515	-0.0049	-3	67	80	GILAMDESNATCGK			Carbamidomethyl (C)[12], Oxidation (M)[5]		Mascot
1543.8187	1543.8396	0.0209	14	81	94	RLASIGLENTEANR					Mascot
2363.1653	2363.1926	0.0273	12	110	130	YISGAILFEETLYQSTVDG EK					Mascot
2370.1506	2370.1514	0.0008	0	149	171	GLVPLPGSNNESWCQGL DGLASR					Mascot
2427.1721	2427.2256	0.0535	22	149	171	GLVPLPGSNNESWCQGL DGLASR			Carbamidomethyl (C)[14]		Mascot
2427.1721	2427.2256	0.0535	22	149	171	GLVPLPGSNNESWCQGL DGLASR	76	100	Carbamidomethyl (C)[14]		Mascot

60

ribulose bisphosphate carboxylase/oxygenase activase, chloroplastic [Momordica charantia]	XP_022138899.1	48023.3	6.27	9	342	100	100

Peptide Information

Calc. Mass	Obsrv. Mass	± da	ppm	Start Seq.	End Seq.	Sequence	Ion Score	C. I. %	Modification	Rank	Result Type
895.4156	895.4451	0.0295	33	356	362	VYDDEVR					Mascot
940.4675	940.4932	0.0257	27	307	313	FYWAPTR					Mascot
940.4675	940.4932	0.0257	27	307	313	FYWAPTR	26	77.394			Mascot

1192.6395	1192.562	-0.0775	-65	387	396	FEQPKMTLAK					Mascot
1228.642	1228.6311	-0.0109	-9	326	336	TDNVPVEDIVK					Mascot
1639.7567	1639.7949	0.0382	23	226	240	MSCLFINDLDAGAGR			Carbamidomethyl (C)[3]		Mascot
1655.7517	1655.7866	0.0349	21	226	240	MSCLFINDLDAGAGR			Carbamidomethyl (C)[3], Oxidation (M)[1]		Mascot
1655.7517	1655.7866	0.0349	21	226	240	MSCLFINDLDAGAGR	16	0	Carbamidomethyl (C)[3], Oxidation (M)[1]		Mascot
1882.9698	1883.0193	0.0495	26	337	353	LVDTFPGQSIDFFGALR					Mascot
1882.9698	1883.0193	0.0495	26	337	353	LVDTFPGQSIDFFGALR	121	100			Mascot
1906.9579	1906.9425	-0.0154	-8	397	412	LLEYGNMLVQEQENVK					Mascot
1922.9529	1922.9109	-0.042	-22	397	412	LLEYGNMLVQEQENVK			Oxidation (M)[7]		Mascot
2089.1692	2089.2251	0.0559	27	282	300	VPIIVTGNDFSTLYAPLIR					Mascot
2089.1692	2089.2251	0.0559	27	282	300	VPIIVTGNDFSTLYAPLIR	160	100			Mascot
2309.0459	2309.0735	0.0276	12	186	208	MGISPIMMSAGELESGNA GEPAK			Oxidation (M)[1,7]		Mascot

61

S-adenosylmethionine synthase 2 [Momordica charantia]	XP_022150092.1	43024.6	5.5	18	484	100	100

Protein Group

S-adenosylmethionine synthase 2 [Momordica charantia]	XP_022150098.1	43024.6	5.5

Peptide Information

Calc. Mass	Obsrv. Mass	± da	ppm	Start Seq.	End Seq.	Sequence	Ion Score	C. I. %	Modification	Rank	Result Type
873.5152	873.5496	0.0344	39	292	300	SIVASGLAR					Mascot
919.4778	919.5509	0.0731	80	68	74	IVRDTCR			Carbamidomethyl (C)[6]		Mascot
979.4744	979.5043	0.0299	31	365	373	TAAYGHFGR					Mascot
979.4744	979.5043	0.0299	31	365	373	TAAYGHFGR	68	99.999			Mascot
1141.6113	1141.6421	0.0308	27	228	237	TIFHLNPSGR					Mascot
1141.6113	1141.6421	0.0308	27	228	237	TIFHLNPSGR	63	99.995			Mascot
1163.6532	1163.6078	-0.0454	-39	281	291	SGAYIVRQAAK					Mascot
1400.674	1400.7146	0.0406	29	158	169	NGTCPWLRPDGK			Carbamidomethyl (C)[4]		Mascot
1453.7548	1453.7942	0.0394	27	238	252	FVIGGPHGDAGLTGR					Mascot
1453.7548	1453.7942	0.0394	27	238	252	FVIGGPHGDAGLTGR	115	100			Mascot
1471.7474	1471.7659	0.0185	13	157	169	KNGTCPWLRPDGK					Mascot
1486.728	1486.7524	0.0244	16	48	60	TNMVMVFGEITTK			Oxidation (M)[3]		Mascot

1502.723	1502.7617	0.0387	26	48	60	TNMVMVFGEITTK			Oxidation (M)[3,5]		Mascot
1528.7689	1528.7963	0.0274	18	157	169	KNGTCPWLRPDGK			Carbamidomethyl (C)[5]		Mascot
1581.8496	1581.8647	0.0151	10	238	253	FVIGGPHGDAGLTGRK					Mascot
1789.9232	1789.9152	-0.008	-4	223	237	YLDEKTIFHLNPSGR					Mascot
1963.9662	1964.0179	0.0517	26	254	273	IIIDTYGGWGAHGGGAFS GK					Mascot
2023.0317	2023.0768	0.0451	22	339	355	ENFDFRPGMITINLDLK					Mascot
2039.0266	2039.0629	0.0363	18	339	355	ENFDFRPGMITINLDLK			Oxidation (M)[9]		Mascot
2092.061	2092.0745	0.0135	6	253	273	KIIIDTYGGWGAHGGGAF SGK					Mascot
2334.0588	2334.1108	0.052	22	19	39	LCDQISDAVLDACLAQDP DSK			Carbamidomethyl (C)[2,13]		Mascot
2417.2573	2417.3259	0.0686	28	92	113	VLVNIEQQSPDIAQGVHG HFTK					Mascot
2417.2573	2417.3259	0.0686	28	92	113	VLVNIEQQSPDIAQGVHG	139	100			Mascot

						HFTK					
2438.1072	2438.259	0.1518	62	40	60	VACETCSKTNMVMVFGEI TTK			Carbamidomethyl (C)[3,6], Oxidation (M)[11,13]		Mascot
3713.7576	3713.9131	0.1555	42	114	147	RPEEIGAGDQGHMFGYA TDETPELMPLSHVLATK			Oxidation (M)[13]		Mascot

62

ribulose-1,5-bisphosphate carboxylase/oxygenase large subunit, partial (chloroplast) [Momordica charantia]	ABG24929.1	46428.5	6.31	18	368	100	100

Peptide Information

Calc. Mass	Obsrv. Mass	± da	ppm	Start Seq.	End Seq.	Sequence	Ion Score	C. I. %	Modification	Rank	Result Type
801.4869	801.4897	0.0028	3	95	101	IPPAYIK					Mascot
830.4366	830.4665	0.0299	36	395	401	EGNEIIR					Mascot
898.41	898.467	0.057	63	261	267	NHGMHFR					Mascot
910.4451	910.4766	0.0315	35	143	149	AVYECLR			Carbamidomethyl (C)[5]		Mascot
914.405	914.4319	0.0269	29	261	267	NHGMHFR			Oxidation (M)[4]		Mascot
914.405	914.4319	0.0269	29	261	267	NHGMHFR	5	0	Oxidation (M)[4]		Mascot

928.4669	928.4924	0.0255	27	251	258	AMHAVIDR			Oxidation (M)[2]		Mascot
962.4789	962.5056	0.0267	28	183	191	SQAETGEIK					Mascot
1116.583	1116.621	0.038	34	377	386	VALEACVQAR			Carbamidomethyl (C)[6]		Mascot
1116.583	1116.621	0.038	34	377	386	VALEACVQAR	13	0	Carbamidomethyl (C)[6]		Mascot
1154.5636	1154.5815	0.0179	16	259	267	QKNHGMHFR					Mascot
1170.5586	1170.6721	0.1135	97	259	267	QKNHGMHFR			Oxidation (M)[6]		Mascot
1170.5586	1170.6721	0.1135	97	259	267	QKNHGMHFR			Oxidation (M)[6]		Mascot
1187.6644	1187.6991	0.0347	29	241	250	DNGLLLHIHR					Mascot
1187.6644	1187.6991	0.0347	29	241	250	DNGLLLHIHR	71	100			Mascot
1188.6122	1188.6937	0.0815	69	173	182	FLFCAEAIFK					Mascot
1245.6433	1245.6847	0.0414	33	395	405	EGNEIIREASK					Mascot
1445.8297	1445.818	-0.0117	-8	120	132	YGRPLLGCTIKPK					Mascot
1447.7653	1447.7808	0.0155	11	275	289	LSGGDHIHAGTVVGK					Mascot
1451.6219	1451.6726	0.0507	35	157	168	DDENVNSQPFMR					Mascot
1465.7546	1465.7966	0.042	29	102	114	TFQGPPHGIQVER					Mascot
1465.7546	1465.7966	0.042	29	102	114	TFQGPPHGIQVER	99	100			Mascot
1489.7145	1489.7439	0.0294	20	406	418	WSPELAAACEVWK					Mascot
1502.8512	1502.8892	0.038	25	120	132	YGRPLLGCTIKPK			Carbamidomethyl (C)[8]		Mascot
1502.8512	1502.8892	0.038	25	120	132	YGRPLLGCTIKPK	22	52.458	Carbamidomethyl (C)[8]		Mascot
1516.7617	1516.879	0.1173	77	171	182	DRFLFCAEAIFK			Carbamidomethyl (C)[6]		Mascot

1546.736	1546.7755	0.0395	26	406	418	WSPELAAACEVWK			Carbamidomethyl (C)[9]		Mascot
2169.9871	2170.042	0.0549	25	150	168	GGLDFTKDDENVNSQPFMR					Mascot
2185.9819	2186.0325	0.0506	23	150	168	GGLDFTKDDENVNSQPFMR			Oxidation (M)[18]		Mascot
2185.9819	2186.0325	0.0506	23	150	168	GGLDFTKDDENVNSQPFMR	69	100	Oxidation (M)[18]		Mascot

63

ribulose-1,5-bisphosphate carboxylase/oxygenase large subunit, partial (chloroplast) [Momordica charantia]	AFH05590.1	18059.3	5.5	6	431	100	100

Peptide Information

Calc. Mass	Obsrv. Mass	± da	ppm	Start	End	Sequence	Ion	C. I. %	Modification	Rank	Result Type

				Seq.	Seq.		Score				
1021.5312	1021.5706	0.0394	39	17	25	DTDILAAFR					Mascot
1021.5312	1021.5706	0.0394	39	17	25	DTDILAAFR	90	100			Mascot
1407.6678	1407.7159	0.0481	34	6	16	LTYYTPEYETK					Mascot
1445.8297	1445.826	-0.0037	-3	149	161	YGRPLLGCTIKPK					Mascot
1465.7546	1465.811	0.0564	38	131	143	TFQGPPHGIQVER					Mascot
1465.7546	1465.811	0.0564	38	131	143	TFQGPPHGIQVER	115	100			Mascot
1502.8512	1502.9044	0.0532	35	149	161	YGRPLLGCTIKPK			Carbamidomethyl (C)[8]		Mascot
1502.8512	1502.9044	0.0532	35	149	161	YGRPLLGCTIKPK	11	0	Carbamidomethyl (C)[8]		Mascot
2410.1814	2410.2683	0.0869	36	6	25	LTYYTPEYETKDTDILAAF R					Mascot
3854.8721	3855.0991	0.227	59	26	63	VTPQPGVPPEEAGAAVA AESSTGTWTTVWTDGLT SLDR					Mascot
3854.8721	3855.0991	0.227	59	26	63	VTPQPGVPPEEAGAAVA AESSTGTWTTVWTDGLT SLDR	182	100			Mascot

64	ribulose-1,5-bisphosphate carboxylase/oxygenase large subunit, partial (chloroplast) [Momordica charantia]	ABG24929.1	46428.5	6.31	15	426	100	100

Peptide Information

Calc. Mass	Obsrv. Mass	± da	ppm	Start Seq.	End Seq.	Sequence	Ion Score	C. I. %	Modification	Rank	Result Type
830.4366	830.4578	0.0212	26	395	401	EGNEIIR					Mascot
853.4236	853.4368	0.0132	15	143	149	AVYECLR					Mascot
898.41	898.425	0.015	17	261	267	NHGMHFR					Mascot
910.4451	910.4624	0.0173	19	143	149	AVYECLR			Carbamidomethyl (C)[5]		Mascot
910.4451	910.4624	0.0173	19	143	149	AVYECLR	37	97.995	Carbamidomethyl (C)[5]		Mascot
914.405	914.4193	0.0143	16	261	267	NHGMHFR			Oxidation (M)[4]		Mascot
928.4669	928.4823	0.0154	17	251	258	AMHAVIDR			Oxidation (M)[2]		Mascot
962.4789	962.4952	0.0163	17	183	191	SQAETGEIK					Mascot
1116.583	1116.6035	0.0205	18	377	386	VALEACVQAR			Carbamidomethyl (C)[6]		Mascot
1116.583	1116.6035	0.0205	18	377	386	VALEACVQAR	40	98.981	Carbamidomethyl (C)[6]		Mascot
1154.5636	1154.5768	0.0132	11	259	267	QKNHGMHFR					Mascot
1170.5586	1170.6598	0.1012	86	259	267	QKNHGMHFR			Oxidation (M)[6]		Mascot
1170.5586	1170.6598	0.1012	86	259	267	QKNHGMHFR			Oxidation (M)[6]		Mascot

1187.6644	1187.6851	0.0207	17	241	250	DNGLLLHIHR					Mascot
1187.6644	1187.6851	0.0207	17	241	250	DNGLLLHIHR	63	99.995			Mascot
1188.6122	1188.6777	0.0655	55	173	182	FLFCAEAIFK					Mascot
1245.6433	1245.6448	0.0015	1	395	405	EGNEIIREASK					Mascot
1451.6219	1451.6488	0.0269	19	157	168	DDENVNSQPFMR					Mascot
1465.7546	1465.7505	-0.0041	-3	102	114	TFQGPPHGIQVER					Mascot
1467.6169	1467.6356	0.0187	13	157	168	DDENVNSQPFMR			Oxidation (M)[11]		Mascot
1467.6169	1467.6356	0.0187	13	157	168	DDENVNSQPFMR	34	95.925	Oxidation (M)[11]		Mascot
1489.7145	1489.6837	-0.0308	-21	406	418	WSPELAAACEVWK					Mascot
1546.736	1546.7526	0.0166	11	406	418	WSPELAAACEVWK			Carbamidomethyl (C)[9]		Mascot
1546.736	1546.7526	0.0166	11	406	418	WSPELAAACEVWK	83	100	Carbamidomethyl (C)[9]		Mascot
1904.9211	1904.8506	-0.0705	-37	402	418	EASKWSPELAAACEVWK					Mascot
2169.9871	2170.0247	0.0376	17	150	168	GGLDFTKDDENVNSQPF MR					Mascot

2169.9871	2170.0247	0.0376	17	150	168	GGLDFTKDDENVNSQPF MR	47	99.81			Mascot
2185.9819	2186.0059	0.024	11	150	168	GGLDFTKDDENVNSQPF MR			Oxidation (M)[18]		Mascot
2185.9819	2186.0059	0.024	11	150	168	GGLDFTKDDENVNSQPF MR	104	100	Oxidation (M)[18]		Mascot

65

nucleoside diphosphate kinase 3-like [Momordica charantia] XP_022136350.1 25999.4 9.16 5 126 100 100

Peptide Information

Calc. Mass	Obsrv. Mass	± da	ppm	Start Seq.	End Seq.	Sequence	Ion Score	C. I. %	Modification	Rank	Result Type
812.405	812.4135	0.0085	10	135	140	HYHDLK					Mascot
1300.7332	1300.7234	-0.0098	-8	32	44	AVASAAAVSLRER					Mascot
1338.6649	1338.6731	0.0082	6	199	211	NIIHGSDGPETAK					Mascot
1344.7634	1344.7819	0.0185	14	91	102	TFIAIKPDGVQR					Mascot
1344.7634	1344.7819	0.0185	14	91	102	TFIAIKPDGVQR	111	100			Mascot
1415.7067	1415.6949	-0.0118	-8	130	140	EFAQKHYHDLK					Mascot

66

ribulose bisphosphate carboxylase small chain, chloroplastic-like [Momordica charantia]	XP_022150468.1	20525.3	8.97	4	69	99.617	99.974

Peptide Information

Calc. Mass	Obsrv. Mass	± da	ppm	Start Seq.	End Seq.	Sequence	Ion Score	C. I. %	Modification	Rank	Result Type
914.4003	914.4161	0.0158	17	117	124	SPGYYDGR	57	99.981			Mascot
914.4229	914.4161	-0.0068	-7	125	130	YWTMWK					Mascot
919.4996	919.5142	0.0146	16	34	42	SAAAFPVTR					Mascot
919.4996	919.5142	0.0146	16	34	42	SAAAFPVTR					Mascot
930.4178	930.4258	0.008	9	125	130	YWTMWK			Oxidation (M)[4]		Mascot
982.4992	982.4714	-0.0278	-28	152	159	EYPSAFIR					Mascot

67

ribulose bisphosphate carboxylase small chain, chloroplastic-like [Momordica charantia]	XP_022150468.1	20525.3	8.97	4	70	99.689	99.976

Peptide Information

Calc. Mass	Obsrv. Mass	± da	ppm	Start Seq.	End Seq.	Sequence	Ion Score	C. I. %	Modification	Rank	Result Type
914.4003	914.4124	0.0121	13	117	124	SPGYYDGR	57	99.982			Mascot
914.4229	914.4124	-0.0105	-11	125	130	YWTMWK					Mascot
919.4996	919.5109	0.0113	12	34	42	SAAAFPVTR					Mascot
919.4996	919.5109	0.0113	12	34	42	SAAAFPVTR					Mascot
930.4178	930.4186	0.0008	1	125	130	YWTMWK			Oxidation (M)[4]		Mascot
2239.0642	2239.1184	0.0542	24	95	112	SGWIPCIEFELEHGFVYR			Carbamidomethyl (C)[6]		Mascot

68

serine hydroxymethyltransferase 2, mitochondrial [Momordica charantia]	XP_022131877.1	56947.3	8.59	13	170	100	100

Peptide Information

Calc. Mass	Obsrv. Mass	± da	ppm	Start Seq.	End Seq.	Sequence	Ion Score	C. I. %	Modification	Rank	Result Type

800.3937	800.4091	0.0154	19	234	239	LYDYAR					Mascot
857.4338	857.4462	0.0124	14	290	296	GAMIFFR			Oxidation (M)[3]		Mascot
999.453	999.4618	0.0088	9	99	107	YSEGYPGAR					Mascot
1066.5569	1066.5725	0.0156	15	499	507	QFPTIGFEK					Mascot
1091.6207	1091.6329	0.0122	11	223	233	LIVAGASAYAR					Mascot
1140.5354	1140.5896	0.0542	48	471	480	DFVATMQSNK					Mascot
1154.5729	1154.4988	-0.0741	-64	446	455	VAEFFDEAVK					Mascot
1585.8003	1585.7981	-0.0022	-1	412	427	NTVPGDVSAMVPGGIR			Oxidation (M)[10]		Mascot
1585.8003	1585.7981	-0.0022	-1	412	427	NTVPGDVSAMVPGGIR	17	0	Oxidation (M)[10]		Mascot
1676.8353	1676.8373	0.002	1	186	199	ISAVSIFFETMPYR			Oxidation (M)[11]		Mascot
1804.9303	1804.9166	-0.0137	-8	185	199	KISAVSIFFETMPYR			Oxidation (M)[12]		Mascot
1816.8236	1816.8385	0.0149	8	200	214	LDESTGYIDYDQLER					Mascot
1816.8236	1816.8385	0.0149	8	200	214	LDESTGYIDYDQLER	115	100			Mascot
2056.8628	2056.8684	0.0056	3	108	124	YYGGNEYIDMAESLCQK			Carbamidomethyl (C)[15], Oxidation (M)[10]		Mascot
2307.1187	2307.1077	-0.011	-5	164	184	IMALDLPHGGHLSHGYQ TDTK			Oxidation (M)[2]		Mascot

69

catalase isozyme 1 [Momordica charantia] XP_022132848.1 56846.2 6.84 14 378 100 100

Peptide Information

Calc. Mass	Obsrv. Mass	± da	ppm	Start Seq.	End Seq.	Sequence	Ion Score	C. I. %	Modification	Rank	Result Type
988.5211	988.5262	0.0051	5	103	110	FSTVIHER					Mascot
1069.5175	1069.5258	0.0083	8	164	171	SHIQENWR					Mascot
1069.5175	1069.5258	0.0083	8	164	171	SHIQENWR	33	94.874			Mascot
1127.5804	1127.582	0.0016	1	111	120	GSPETLRDPR					Mascot
1136.6787	1136.6842	0.0055	5	92	102	APGVQTPVIVR					Mascot
1226.5988	1226.6064	0.0076	6	406	416	YPHPPAVCTGK			Carbamidomethyl (C)[8]		Mascot
1255.559	1255.5686	0.0096	8	387	396	DEEVNYFPSR					Mascot
1495.8268	1495.8201	-0.0067	-4	356	369	LGPNYLQLPANAPK					Mascot
1525.826	1525.7611	-0.0649	-43	38	50	GPILLEDYHLVEK					Mascot
1525.826	1525.7611	-0.0649	-43	38	50	GPILLEDYHLVEK	33	95.083			Mascot
1620.8745	1620.8832	0.0087	5	292	305	TWPEDILPLQPVGR					Mascot
1620.8745	1620.8832	0.0087	5	292	305	TWPEDILPLQPVGR	108	100			Mascot
1940.9722	1940.9963	0.0241	12	147	163	DGMKFPDMVHALKPNPK			Oxidation (M)[3]		Mascot
1956.967	1956.991	0.024	12	147	163	DGMKFPDMVHALKPNPK			Oxidation (M)[3,8]		Mascot

1984.9917	1985.0031	0.0114	6	130	146	EGNFDLVGNNFPVFFIR					Mascot
1984.9917	1985.0031	0.0114	6	130	146	EGNFDLVGNNFPVFFIR	153	100			Mascot
2175.8918	2175.8796	-0.0122	-6	370	386	CAHHNNHHEGFMNFMHR			Carbamidomethyl (C)[1]		Mascot
2191.887	2191.8774	-0.0096	-4	370	386	CAHHNNHHEGFMNFMHR			Carbamidomethyl (C)[1], Oxidation (M)[12]		Mascot
2207.8818	2207.8816	-0.0002	0	370	386	CAHHNNHHEGFMNFMHR			Carbamidomethyl (C)[1], Oxidation (M)[12,15		Mascot
2242.1868	2242.0735	-0.1133	-51	38	56	GPILLEDYHLVEKLANFDR					Mascot
2242.1868	2242.0735	-0.1133	-51	38	56	GPILLEDYHLVEKLANFDR					Mascot
2817.2864	2817.3	0.0136	5	243	268	VGGSNHSHATQDLYDSIAAGNYPEWK					Mascot

70

ribulose bisphosphate carboxylase/oxygenase activase, chloroplastic [Momordica charantia]	XP_022138899.1	48023.3	6.27	11	391	100	100

Peptide Information

Calc. Mass	Obsrv. Mass	± da	ppm	Start Seq.	End Seq.	Sequence	Ion Score	C. I. %	Modification	Rank	Result Type
809.5243	809.5201	-0.0042	-5	145	151	LVIHISK					Mascot
895.4156	895.4248	0.0092	10	356	362	VYDDEVR					Mascot
940.4675	940.4709	0.0034	4	307	313	FYWAPTR					Mascot
940.4675	940.4709	0.0034	4	307	313	FYWAPTR	33	95.691			Mascot
1152.714	1152.7137	-0.0003	0	161	171	VPLILGIWGGK					Mascot
1228.6031	1228.603	-0.0001	0	176	185	SFQCELVFAK			Carbamidomethyl (C)[4]		Mascot
1340.6382	1340.6416	0.0034	3	307	316	FYWAPTREDR					Mascot
1639.7567	1639.7626	0.0059	4	226	240	MSCLFINDLDAGAGR			Carbamidomethyl (C)[3]		Mascot
1639.7567	1639.7626	0.0059	4	226	240	MSCLFINDLDAGAGR	93	100	Carbamidomethyl (C)[3]		Mascot
1655.7517	1655.7527	0.001	1	226	240	MSCLFINDLDAGAGR			Carbamidomethyl (C)[3], Oxidation (M)[1]		Mascot
1882.9698	1882.9771	0.0073	4	337	353	LVDTFPGQSIDFFGALR					Mascot
1882.9698	1882.9771	0.0073	4	337	353	LVDTFPGQSIDFFGALR	138	100			Mascot
1906.9579	1906.9238	-0.0341	-18	397	412	LLEYGNMLVQEQENVK					Mascot
2089.1692	2089.175	0.0058	3	282	300	VPIIVTGNDFSTLYAPLIR					Mascot

2089.1692	2089.175	0.0058	3	282	300	VPIIVTGNDFSTLYAPLIR	99	100			Mascot
2293.0508	2293.0439	-0.0069	-3	186	208	MGISPIMMSAGELESGNA GEPAK			Oxidation (M)[1]		Mascot

71

ribulose-1,5-bisphosphate carboxylase/oxygenase large subunit, partial (chloroplast) [Momordica charantia]	ABG24929.1	46428.5	6.31	15	422	100	100

Peptide Information

Calc. Mass	Obsrv. Mass	± da	ppm	Start Seq.	End Seq.	Sequence	Ion Score	C. I. %	Modification	Rank	Result Type
830.4366	830.4485	0.0119	14	395	401	EGNEIIR					Mascot
853.4236	853.4232	-0.0004	0	143	149	AVYECLR					Mascot
898.41	898.4143	0.0043	5	261	267	NHGMHFR					Mascot
910.4451	910.4497	0.0046	5	143	149	AVYECLR			Carbamidomethyl (C)[5]		Mascot
910.4451	910.4497	0.0046	5	143	149	AVYECLR	29	87.902	Carbamidomethyl (C)[5]		Mascot
912.472	912.4608	-0.0112	-12	251	258	AMHAVIDR					Mascot
914.405	914.4139	0.0089	10	261	267	NHGMHFR			Oxidation (M)[4]		Mascot
928.4669	928.4606	-0.0063	-7	251	258	AMHAVIDR			Oxidation (M)[2]		Mascot
962.4789	962.4802	0.0013	1	183	191	SQAETGEIK					Mascot
1059.5615	1059.549	-0.0125	-12	377	386	VALEACVQAR					Mascot
1116.583	1116.589	0.006	5	377	386	VALEACVQAR			Carbamidomethyl (C)[6]		Mascot
1116.583	1116.589	0.006	5	377	386	VALEACVQAR	23	59.29	Carbamidomethyl (C)[6]		Mascot
1154.5636	1154.592	0.0284	25	259	267	QKNHGMHFR					Mascot
1170.5586	1170.6447	0.0861	74	259	267	QKNHGMHFR			Oxidation (M)[6]		Mascot
1170.5586	1170.6447	0.0861	74	259	267	QKNHGMHFR			Oxidation (M)[6]		Mascot
1187.6644	1187.6715	0.0071	6	241	250	DNGLLLHIHR					Mascot
1188.6122	1188.6649	0.0527	44	173	182	FLFCAEAIFK					Mascot
1245.6337	1245.6309	-0.0028	-2	173	182	FLFCAEAIFK			Carbamidomethyl (C)[4]		Mascot
1451.6219	1451.6287	0.0068	5	157	168	DDENVNSQPFMR					Mascot
1451.6219	1451.6287	0.0068	5	157	168	DDENVNSQPFMR	85	100			Mascot
1465.7546	1465.6659	-0.0887	-61	102	114	TFQGPPHGIQVER					Mascot
1467.6169	1467.6215	0.0046	3	157	168	DDENVNSQPFMR			Oxidation (M)[11]		Mascot
1516.7617	1516.7577	-0.004	-3	171	182	DRFLFCAEAIFK			Carbamidomethyl (C)[6]		Mascot
1546.736	1546.7327	-0.0033	-2	406	418	WSPELAAACEVWK			Carbamidomethyl (C)[9]		Mascot
1546.736	1546.7327	-0.0033	-2	406	418	WSPELAAACEVWK	74	100	Carbamidomethyl (C)[9]		Mascot
1904.9211	1904.8326	-0.0885	-46	402	418	EASKWSPELAAACEVWK					Mascot

	2169.9871	2169.9934	0.0063	3	150	168	GGLDFTKDDENVNSQPF MR					Mascot
	2169.9871	2169.9934	0.0063	3	150	168	GGLDFTKDDENVNSQPF MR	144	100			Mascot
	2185.9819	2185.9783	-0.0036	-2	150	168	GGLDFTKDDENVNSQPF MR			Oxidation (M)[18]		Mascot
	2185.9819	2185.9783	-0.0036	-2	150	168	GGLDFTKDDENVNSQPF MR	15	0	Oxidation (M)[18]		Mascot

72

ribulose-1,5-bisphosphate carboxylase/oxygenase large subunit, partial (chloroplast) [Momordica charantia]	ABG24929.1	46428.5	6.31	14	300	100		100

Peptide Information

Calc. Mass	Obsrv. Mass	± da	ppm	Start Seq.	End Seq.	Sequence	Ion Score	C. I. %	Modification	Rank	Result Type
830.4366	830.4482	0.0116	14	395	401	EGNEIIR					Mascot
898.41	898.4178	0.0078	9	261	267	NHGMHFR					Mascot
910.4451	910.4515	0.0064	7	143	149	AVYECLR			Carbamidomethyl (C)[5]		Mascot
910.4451	910.4515	0.0064	7	143	149	AVYECLR	9	0	Carbamidomethyl (C)[5]		Mascot
912.472	912.4621	-0.0099	-11	251	258	AMHAVIDR					Mascot
914.405	914.4131	0.0081	9	261	267	NHGMHFR			Oxidation (M)[4]		Mascot
928.4669	928.4742	0.0073	8	251	258	AMHAVIDR			Oxidation (M)[2]		Mascot
962.4789	962.4772	-0.0017	-2	183	191	SQAETGEIK					Mascot
1059.5615	1059.5374	-0.0241	-23	377	386	VALEACVQAR					Mascot
1116.583	1116.59	0.007	6	377	386	VALEACVQAR			Carbamidomethyl (C)[6]		Mascot
1116.583	1116.59	0.007	6	377	386	VALEACVQAR	37	98.177	Carbamidomethyl (C)[6]		Mascot
1154.5636	1154.5735	0.0099	9	259	267	QKNHGMHFR					Mascot
1170.5586	1170.6476	0.089	76	259	267	QKNHGMHFR			Oxidation (M)[6]		Mascot
1170.5586	1170.6476	0.089	76	259	267	QKNHGMHFR			Oxidation (M)[6]		Mascot
1187.6644	1187.675	0.0106	9	241	250	DNGLLLHIHR					Mascot
1187.6644	1187.675	0.0106	9	241	250	DNGLLLHIHR	39	98.996			Mascot
1188.6122	1188.6692	0.057	48	173	182	FLFCAEAIFK					Mascot
1245.6337	1245.6343	0.0006	0	173	182	FLFCAEAIFK			Carbamidomethyl (C)[4]		Mascot

1451.6219	1451.6307	0.0088	6	157	168	DDENVNSQPFMR					Mascot
1451.6219	1451.6307	0.0088	6	157	168	DDENVNSQPFMR	47	99.809			Mascot
1465.7546	1465.6821	-0.0725	-49	102	114	TFQGPPHGIQVER					Mascot
1467.6169	1467.6229	0.006	4	157	168	DDENVNSQPFMR			Oxidation (M)[11]		Mascot
1546.736	1546.7357	-0.0003	0	406	418	WSPELAAACEVWK			Carbamidomethyl (C)[9]		Mascot
1904.9211	1904.8201	-0.101	-53	402	418	EASKWSPELAAACEVWK					Mascot
2169.9871	2169.9958	0.0087	4	150	168	GGLDFTKDDENVNSQPFMR					Mascot
2169.9871	2169.9958	0.0087	4	150	168	GGLDFTKDDENVNSQPFMR	109	100			Mascot

2185.9819	2185.9844	0.0025	1	150	168	GGLDFTKDDENVNSQPFMR			Oxidation (M)[18]		Mascot
2185.9819	2185.9844	0.0025	1	150	168	GGLDFTKDDENVNSQPFMR	53	99.952	Oxidation (M)[18]		Mascot

73

enolase [Momordica charantia]	XP_022143756.1	47768.5	5.84	16	211	100	100

Peptide Information

Calc. Mass	Obsrv. Mass	± da	ppm	tart Seq.	End Seq.	Sequence	Ion Score	C. I. %	Modification	Rank	Result Type
806.4519	806.4554	0.0035	4	416	421	YNQLLR					Mascot
1087.5103	1087.5144	0.0041	4	372	381	AGWGVMASHR			Oxidation (M)[6]		Mascot
1228.6144	1228.6011	-0.0133	-11	189	198	MGVEVYHHLK			Oxidation (M)[1]		Mascot
1510.8477	1510.8141	-0.0336	-22	322	335	VQIVGDDLLVTNPK					Mascot
1564.9058	1564.8541	-0.0517	-33	66	81	AVENVNAIIGPALVGK					Mascot
1573.8433	1573.8282	-0.0151	-10	353	367	VNQIGSVTESIEAVK					Mascot
1804.944	1804.946	0.002	1	35	52	AAVPSGASTGIYEALELR					Mascot
1804.944	1804.946	0.002	1	35	52	AAVPSGASTGIYEALELR	139	100			Mascot
1810.9333	1810.8239	-0.1094	-60	422	438	IEEELGSAAVYAGAKFR					Mascot
1855.9761	1855.9393	-0.0368	-20	17	34	GNPTVEVDIVLSDGTLAR					Mascot
1868.965	1868.9108	-0.0542	-29	168	184	LAMQEFMILPVGASSFK					Mascot
1884.9598	1884.9525	-0.0073	-4	168	184	LAMQEFMILPVGASSFK			Oxidation (M)[3]		Mascot
1920.0107	1919.9653	-0.0454	-24	353	370	VNQIGSVTESIEAVKMSK					Mascot
2252.1294	2252.125	-0.0044	-2	382	403	SGETEDTFIADLSVGLAT					Mascot

Calc. Mass	Obsrv. Mass	± da	ppm	Start Seq.	End Seq.	Sequence	Ion Score	C. I. %	Modification	Rank	Result Type
						GQIK					
2324.0425	2324.0298	-0.0127	-5	205	226	YGQDATNVGDEGGFAPN IQENK					Mascot
2376.1648	2376.1472	-0.0176	-7	168	188	LAMQEFMILPVGASSFKE AMK			Oxidation (M)[3,7,20]		Mascot
2452.1375	2452.1282	-0.0093	-4	204	226	KYGQDATNVGDEGGFAP NIQENK					Mascot
2988.321	2988.3196	-0.0014	0	289	313	SFASEYPIVSIEDPFDQD DWEHYAK					Mascot

74

29 kDa ribonucleoprotein A, chloroplastic-like [Momordica charantia] XP_022144753.1 30075.1 5.58 5 126 100 100

Peptide Information

Calc. Mass	Obsrv. Mass	± da	ppm	Start Seq.	End Seq.	Sequence	Ion Score	C. I. %	Modification	Rank	Result Type
865.4666	865.4692	0.0026	3	121	127	VEVIYDK					Mascot
968.52	968.5192	-0.0008	-1	165	173	VNYGPPPPK					Mascot
968.52	968.5192	-0.0008	-1	165	173	VNYGPPPPK	64	99.997			Mascot
1120.5131	1120.5601	0.047	42	184	193	NASNFSNPNR					Mascot
1124.6171	1124.6162	-0.0009	-1	265	274	VTQAEARPPR					Mascot
1124.6171	1124.612	-0.0009	-1	265	274	VTQAEARPPR	50	99.919			Mascot
1280.6846	1280.6934	0.0088	7	121	131	VEVIYDKTTGR					Mascot

75

ribulose-1,5-bisphosphate carboxylase/oxygenase large subunit, partial (chloroplast) [Momordica charantia] ABG24929.1 46428.5 6.31 16 361 100 100

Peptide Information

Calc. Mass	Obsrv. Mass	± da	ppm	Start Seq.	End Seq.	Sequence	Ion Score	C. I. %	Modification	Rank	Result Type
830.4366	830.4509	0.0143	17	395	401	EGNEIIR					Mascot
898.41	898.4214	0.0114	13	261	267	NHGMHFR					Mascot
910.4451	910.4567	0.0116	13	143	149	AVYECLR			Carbamidomethyl (C)[5]		Mascot
912.472	912.4658	-0.0062	-7	251	258	AMHAVIDR					Mascot

914.405	914.4141	0.0091	10	261	267	NHGMHFR			Oxidation (M)[4]		Mascot
914.405	914.4141	0.0091	10	261	267	NHGMHFR			Oxidation (M)[4]		Mascot
928.4669	928.4631	-0.0038	-4	251	258	AMHAVIDR			Oxidation (M)[2]		Mascot
930.4752	930.4199	-0.0553	-59	387	394	NEGRDLAR					Mascot
962.4789	962.4514	-0.0275	-29	183	191	SQAETGEIK					Mascot
1116.583	1116.5953	0.0123	11	377	386	VALEACVQAR			Carbamidomethyl (C)[6]		Mascot
1116.583	1116.5953	0.0123	11	377	386	VALEACVQAR	46	99.776	Carbamidomethyl (C)[6]		Mascot
1154.5636	1154.5685	0.0049	4	259	267	QKNHGMHFR					Mascot
1170.5586	1170.6501	0.0915	78	259	267	QKNHGMHFR			Oxidation (M)[6]		Mascot
1170.5586	1170.6501	0.0915	78	259	267	QKNHGMHFR			Oxidation (M)[6]		Mascot
1187.6644	1187.6761	0.0117	10	241	250	DNGLLLHIHR					Mascot
1188.6122	1188.6661	0.0539	45	173	182	FLFCAEAIFK					Mascot
1451.6219	1451.6362	0.0143	10	157	168	DDENVNSQPFMR					Mascot
1451.6219	1451.6362	0.0143	10	157	168	DDENVNSQPFMR	96	100			Mascot
1465.7546	1465.7594	0.0048	3	102	114	TFQGPPHGIQVER					Mascot
1467.6169	1467.7129	0.096	65	157	168	DDENVNSQPFMR			Oxidation (M)[11]		Mascot
1502.8512	1502.8629	0.0117	8	120	132	YGRPLLGCTIKPK			Carbamidomethyl (C)[8]		Mascot
1546.736	1546.7422	0.0062	4	406	418	WSPELAAACEVWK			Carbamidomethyl (C)[9]		Mascot
1904.9211	1904.8501	-0.071	-37	402	418	EASKWSPELAAACEVWK					Mascot
2169.9871	2170.0059	0.0188	9	150	168	GGLDFTKDDENVNSQPF MR					Mascot
2169.9871	2170.0059	0.0188	9	150	168	GGLDFTKDDENVNSQPF MR	147	100			Mascot
2185.9819	2185.9934	0.0115	5	150	168	GGLDFTKDDENVNSQPF			Oxidation (M)[18]		Mascot

MR

76

glycine dehydrogenase (decarboxylating), mitochondrial [Momordica charantia] XP_022132037.1 113402.2 6.88 23 287 100 100

Peptide Information

Calc. Mass	Obsrv. Mass	± da	ppm	Start Seq.	End Seq.	Sequence	Ion Score	C. I. %	Modification	Rank	Result Type
925.5326	925.5627	0.0301	33	15	22	HLVSASRR					Mascot
937.4374	937.45	0.0126	13	###	1028	VDNVYGDR					Mascot

1094.5663	1094.5823	0.016	15	952	960	FCDALISIR			Carbamidomethyl (C)[2]	Mascot
1094.5663	1094.5823	0.016	15	952	960	FCDALISIR	48	99.858	Carbamidomethyl (C)[2]	Mascot
1221.666	1221.6523	-0.0137	-11	865	875	IAILNANYMAK				Mascot
1239.7208	1239.733	0.0122	10	441	453	VHGLAGAFAVGLK				Mascot
1239.7208	1239.733	0.0122	10	441	453	VHGLAGAFAVGLK	72	100		Mascot
1310.6528	1310.6699	0.0171	13	###	1010	EYAAFPASWLR				Mascot
1310.6528	1310.6699	0.0171	13	###	1010	EYAAFPASWLR	76	100		Mascot
1367.8158	1367.7507	-0.0651	-48	441	454	VHGLAGAFAVGLKK				Mascot
1498.8588	1498.8792	0.0204	14	378	392	IIGVSVDSSGKPALR				Mascot
1640.8755	1640.8906	0.0151	9	887	901	GVNGTVAHEFIIDLR				Mascot
1838.9042	1838.9171	0.0129	7	785	803	TFCIPHGGGGPGMGPIG VK			Carbamidomethyl (C)[3]	Mascot
1854.899	1854.9006	0.0016	1	785	803	TFCIPHGGGGPGMGPIG VK			Carbamidomethyl (C)[3], Oxidation (M)[13]	Mascot
1949.8953	1949.9097	0.0144	7	575	591	DLSLCHSMIPLGSCTMK			Carbamidomethyl (C)[5,14]	Mascot
1965.8901	1965.8953	0.0052	3	575	591	DLSLCHSMIPLGSCTMK			Carbamidomethyl (C)[5,14], Oxidation (M)[8]	Mascot
2090.0779	2090.1042	0.0263	13	160	177	SYIGMGYYNTFVPPVILR				Mascot
2090.0779	2090.1042	0.0263	13	160	177	SYIGMGYYNTFVPPVILR	21	26.345		Mascot
2106.073	2106.0925	0.0195	9	160	177	SYIGMGYYNTFVPPVILR			Oxidation (M)[5]	Mascot
2233.1792	2233.0876	-0.0916	-41	378	398	IIGVSVDSSGKPALRMAM QTR			Oxidation (M)[16]	Mascot
2292.1218	2292.0896	-0.0322	-14	571	591	LQSKDLSLCHSMIPLGSC TMK				Mascot
2308.0583	2308.0742	0.0159	7	135	154	FDEGLTESQMIEHMQNL ASK				Mascot
2309.25	2309.0757	-0.1743	-75	523	545	AVPFTAASLAPEVENAIP SGLVR				Mascot
2328.1118	2328.135	0.0232	10	350	371	FGVPMGYGGPHAAFLAT SQEYK				Mascot
2344.1067	2344.1238	0.0171	7	350	371	FGVPMGYGGPHAAFLAT SQEYK			Oxidation (M)[5]	Mascot
2417.2065	2417.1335	-0.073	-30	247	266	KTFIISNNCHPQTIDICVTR			Carbamidomethyl (C)[9,17]	Mascot
2484.2129	2484.2563	0.0434	17	350	372	FGVPMGYGGPHAAFLAT SQEYKR				Mascot

2760.3074	2760.3198	0.0124	4	286	311	SGDVCGVLVQYPGTEGE VLDYGEFVK					Mascot
2776.2671	2776.3137	0.0466	17	178	200	NIMENPAWYTQYTPYQA EISQGR			Oxidation (M)[3]		Mascot
2783.3313	2783.3418	0.0105	4	407	432	ATSNICTAQALLANMAAM YAVYHGPK			Carbamidomethyl (C)[6], Oxidation (M)[15]		Mascot
2955.375	2955.426	0.051	17	720	745	ENLSALMVTYPSTHGVY EEGIDEICK			Carbamidomethyl (C)[25]		Mascot

77

plastocyanin, partial [Momordica charantia]	XP_022137655.1	11734.7	4.26	2	110	100	100

Peptide Information

Calc. Mass	Obsrv. Mass	± da	ppm	Start Seq.	End Seq.	Sequence	Ion Score	C. I. %	Modification	Rank	Result Type
1875.8153	1875.8508	0.0355	19	92	109	GSYSFYCSPHQGAGMV GK					Mascot
1932.8368	1932.8583	0.0215	11	92	109	GSYSFYCSPHQGAGMV GK			Carbamidomethyl (C)[7]		Mascot
1932.8368	1932.8583	0.0215	11	92	109	GSYSFYCSPHQGAGMV GK	99	100	Carbamidomethyl (C)[7]		Mascot
1948.8317	1948.8461	0.0144	7	92	109	GSYSFYCSPHQGAGMV GK			Carbamidomethyl (C)[7], Oxidation (M)[15]		Mascot
1948.8317	1948.8461	0.0144	7	92	109	GSYSFYCSPHQGAGMV GK	100	100	Carbamidomethyl (C)[7], Oxidation (M)[15]		Mascot
2685.3408	2685.3296	-0.0112	-4	19	44	LGGDDGSLAFVPNDFSIS SGDKIVFK					Mascot
