# Supplementary material for: Proteomic analysis of heat stress resistance of cucumber leaves when grafted onto Momordica rootstock
Source: Hortic Res. 2018 Oct 1;5:53. doi: 10.1038/s41438-018-0060-z (PMC6165847; doi:10.1038/s41438-018-0060-z)
Supplement: Supplementary file 3 — Primers used for qRT-PCR assays [file 41438_2018_60_MOESM3_ESM.docx]

**Table S1.** Primers used for qRT-PCR assays.

| Gene name | Forward primer | Reverse primer | |  |
| --- | --- | --- | --- | --- |
| *RbcL* | 5′-AGCCTGTTGCTGGAGAAG-3′ | | 5′-AGGGCGACCATACTTGTT-3′ | |
| *RbcS* | 5′-GCCTCAAATCTTCCGCTGGT-3′ | | 5′-AATCCGCTTCCGATGTCGAAT-3′ | |
| *psbO* | 5′-GCGGCTACCCTTATGCAAC-3′ | | 5′-ATCTTAGCAGCATCGGCAAAC-3′ | |
| *PBGD* | 5′-TCATGGCGTTGGTCTCATAAG-3′ | | 5′-ACAGGCAATCCCAATAGCTCC-3′ | |
| *petC* | 5′-GGCGTCTTCCACTCTATCTTC-3′ | | 5′-AGCGTTTTATCCTTCTCGACT-3′ | |
| *Gsa* | 5′-CGGCTTGCTCGTGCTT-3′ | | 5′-CTGGTTCTTTCAATCGCTTTA-3′ | |
| *Actin* | 5′-CCGTTCTGTCCCTCTACGCTAGTG-3′ | | 5′-GGAACTGCTCTTTGCAGTCTCGAG-3′ | |
