# Supplementary material for: Proteomic analysis of heat stress resistance of cucumber leaves when grafted onto Momordica rootstock
Source: Hortic Res. 2018 Oct 1;5:53. doi: 10.1038/s41438-018-0060-z (PMC6165847; doi:10.1038/s41438-018-0060-z)
Supplement: Supplementary file 4 — 2-DE and Western Blot [file 41438_2018_60_MOESM4_ESM.pptx]

## Slide 1
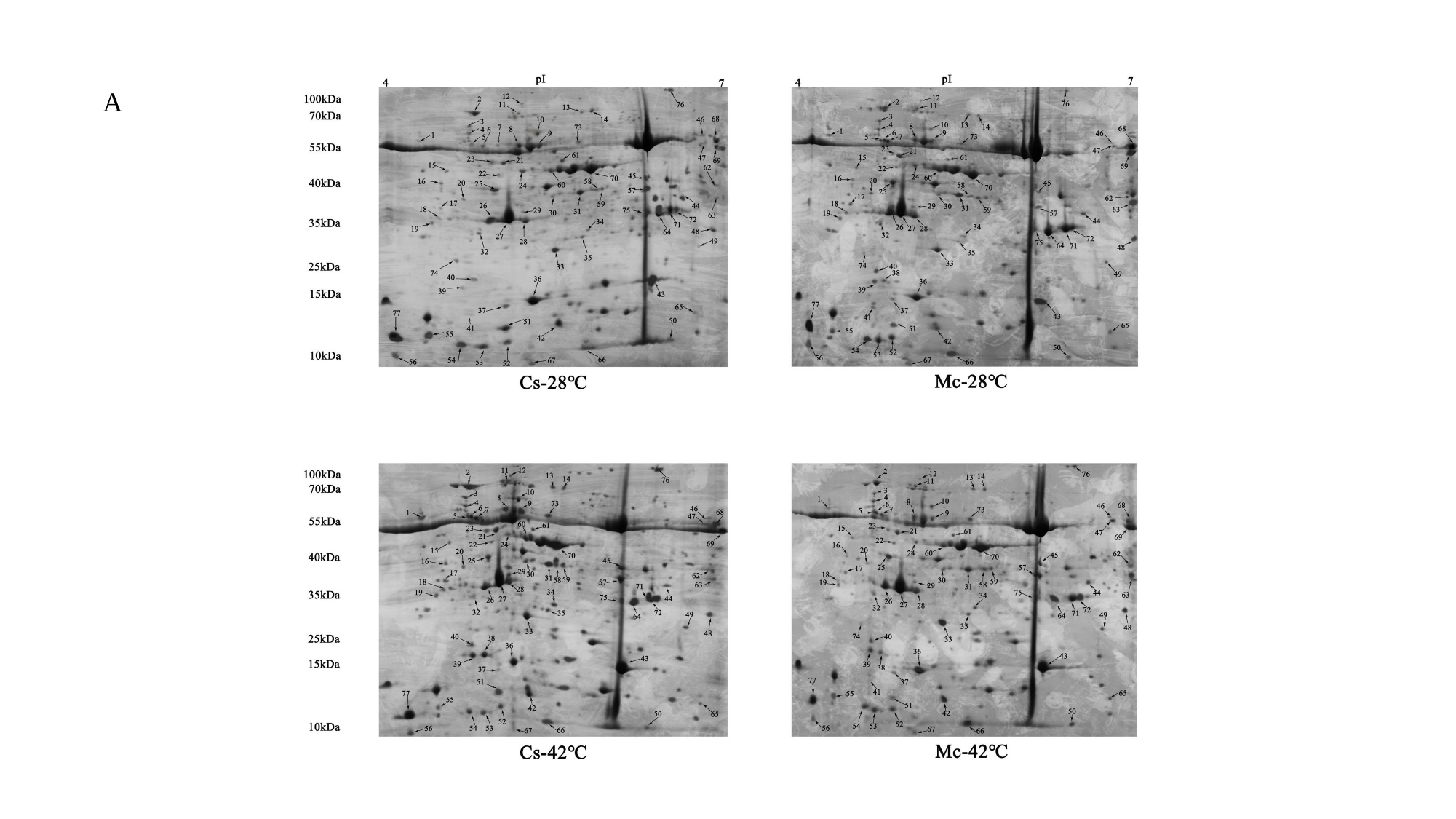

A

## Slide 2
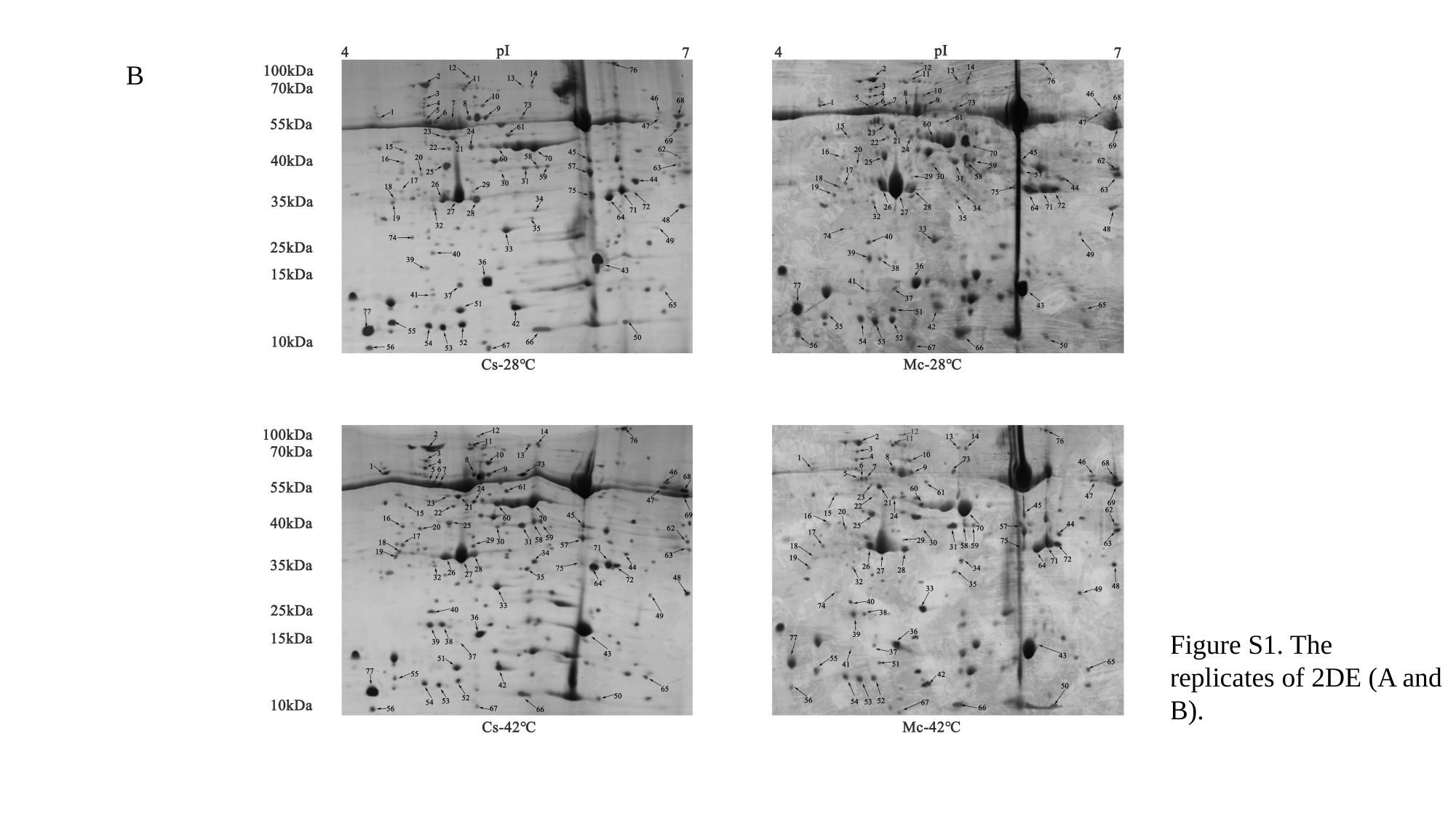

B
Figure S1. The replicates of 2DE (A and B).

## Slide 3
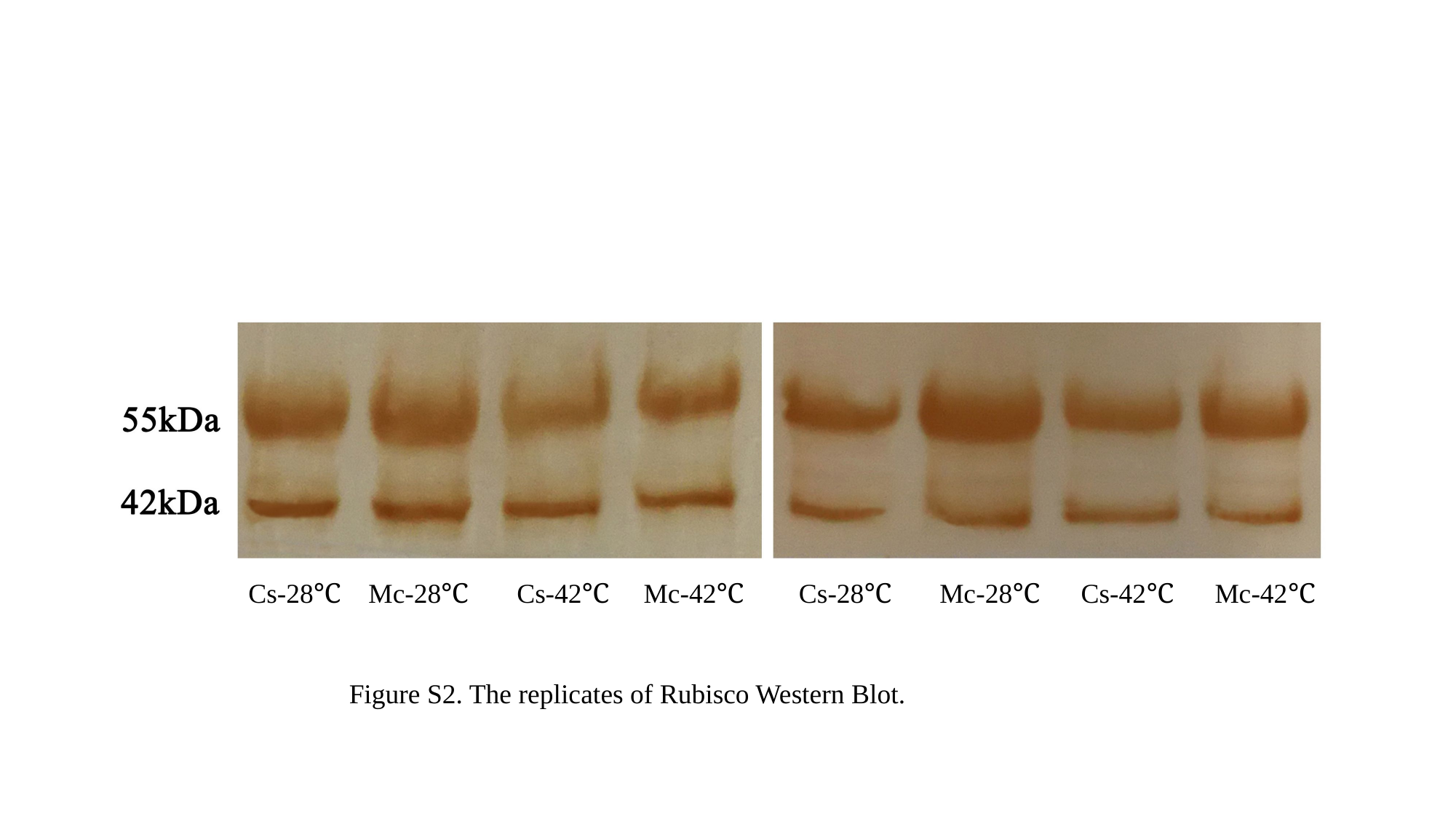

Cs-28℃ Mc-28℃ Cs-42℃ Mc-42℃ Cs-28℃ Mc-28℃ Cs-42℃ Mc-42℃
Figure S2. The replicates of Rubisco Western Blot.

## Slide 4
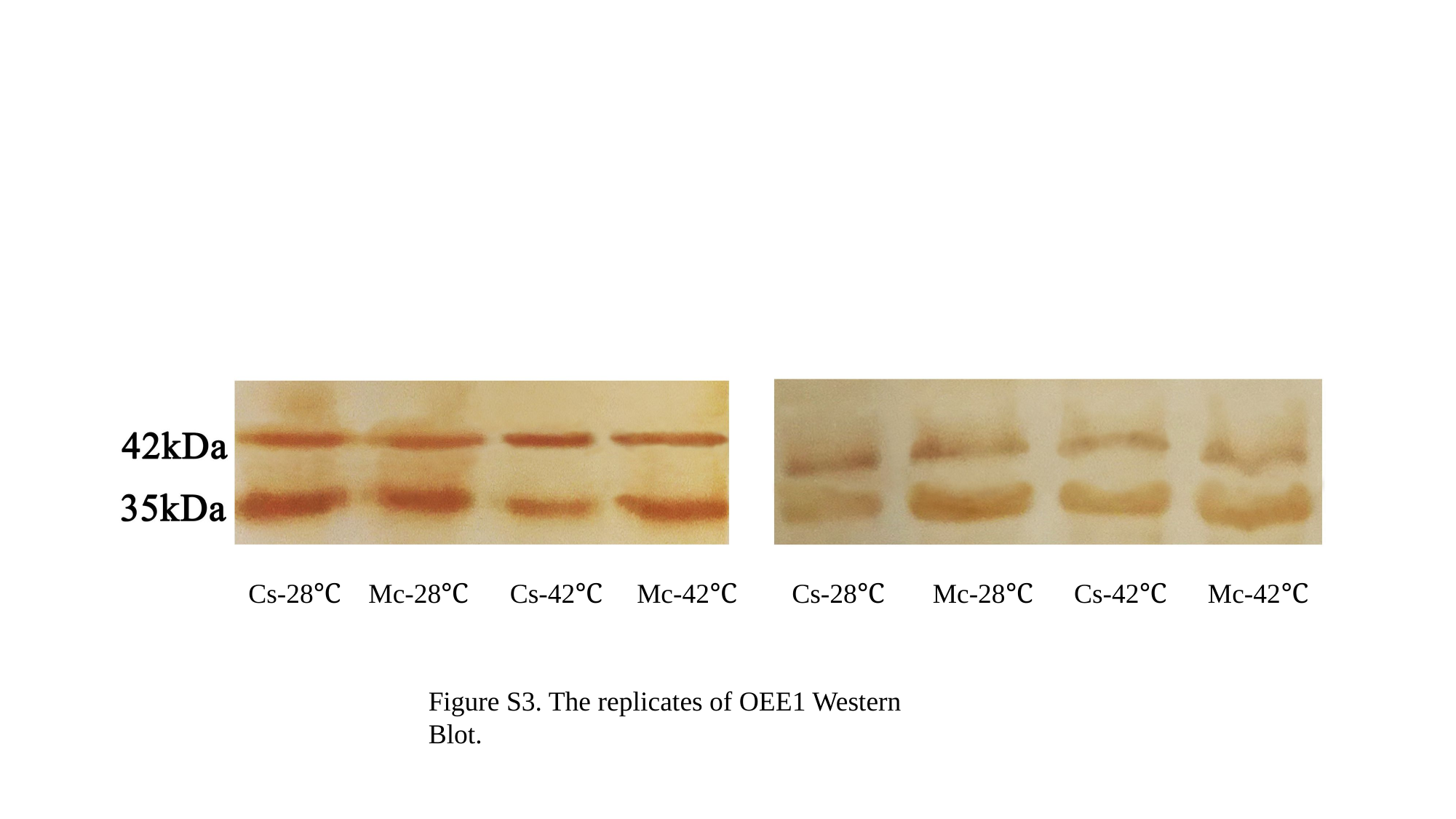

Cs-28℃ Mc-28℃ Cs-42℃ Mc-42℃ Cs-28℃ Mc-28℃ Cs-42℃ Mc-42℃
Figure S3. The replicates of OEE1 Western Blot.
